# Research Poster Abstracts

**DOI:** 10.1080/24740527.2020.1765649

**Published:** 2020-05-15

## Opioid Craving Mediates the Association between Opioid Withdrawal Symptoms and Prescription Opioid Misuse

Alice Bruneau^a,*^, Leah Frimerman^a^, Amanda Sirois^b^, Katherine Scott^a^, Jordi Perez^c^, Yoram Shir^c^ and Marc O. Martel^d^

^a^Department of Experimental Medicine, McGill University, Montreal, Québec, Canada; ^b^Department of Dentistry, McGill University, Montreal, Québec, Canada; ^c^Department of Anesthesia, McGill University, Montreal, Québec, Canada; ^d^Faculty of Dentistry & Department of Anesthesia, McGill University, Montreal, Québec, Canada

**CONTACT** Alice Bruneau alice.bruneau@mail.mcgill.ca

© 2020 The Author(s). Published with license by Taylor & Francis Group, LLC.

This is an Open Access article distributed under the terms of the Creative Commons Attribution License (http://creativecommons.org/licenses/by/4.0/), which permits unrestricted use, distribution, and reproduction in any medium, provided the original work is properly cited.

**Introduction/Aim**: Prescription opioid misuse is observed in up to 20–30% of chronic non-cancer pain (CNCP) patients on long-term opioid therapy. In previous research, factors such as negative affect and opioid craving have emerged as strong determinants of opioid misuse. The contribution of opioid withdrawal symptoms to opioid misuse, however, has remained largely unexplored. The first objective of this study was to examine the association between opioid withdrawal symptoms and opioid misuse among CNCP patients prescribed opioid therapy. We also examined the factors associated with opioid withdrawal symptoms.

**Methods**: In this longitudinal diary study, patients (n = 103) provided reports of pain intensity, negative affect, catastrophizing, opioid withdrawal symptoms, and opioid craving for 14 consecutive days. Prescription opioid misuse was also assessed.

**Results**: A marginally significant association was found between opioid withdrawal symptoms and opioid misuse (r =.19, *p* = .05). Multilevel analyses revealed that higher daily levels of pain, negative affect, and catastrophizing were associated with heightened opioid withdrawal symptoms (all p’s <.05). Opioid withdrawal symptoms were also associated with opioid craving (*p* <.05), and a follow-up analysis indicated that craving mediated the association between opioid withdrawal symptoms and opioid misuse (*p* <.01).

**Discussion/Conclusions**: This study provides new insights into the association between opioid withdrawal symptoms and opioid misuse. Our findings suggest that opioid withdrawal symptoms might lead to opioid misuse indirectly through elevations in opioid craving. Our findings have implications for interventions designed to prevent and/or reduce opioid misuse in patients with pain.

## Pain Intensity versus Unpleasantness: Experimental Pain Sensitivity Differs as a Function of Pain Catastrophizing, Chronic Pain and Sex

Elana R. Abelson^a,*^, Emily A. Beckmann^a^, Hadas Nahman-Averbuch^b^, Christopher D. King^b^, Robert C. Coghill^b^ and Kristen E. Jastrowski Mano^a^

^a^Department of Psychology, University of Cincinnati, Cincinnati, Ohio, USA; ^b^Behavioral Medicine & Clinical Psychology, Cincinnati Children’s Hospital Medical Center, Cincinnati, Ohio, USA

**CONTACT** Elana R. Abelson abelsoea@mail.uc.edu

© 2020 The Author(s). Published with license by Taylor & Francis Group, LLC.

This is an Open Access article distributed under the terms of the Creative Commons Attribution License (http://creativecommons.org/licenses/by/4.0/), which permits unrestricted use, distribution, and reproduction in any medium, provided the original work is properly cited.

**Introduction/Aim**: Pain catastrophizing and clinical pain are often associated with greater experimental pain sensitivity. Previous work has also demonstrated that healthy women display lower pain thresholds than men. However, little work has differentiated between pain intensity versus unpleasantness. The aim of this study was to examine experimental pain responses as a function of sex, experience with chronic pain and pain catastrophizing.

**Methods**: Emerging adults (*N* = 85, *M_age_ = *18.9, 51.2% female) rated pain intensity (0 = no pain, 10 = worst pain imaginable) and unpleasantness (0 to 10) using a computerized visual analogue scale (VAS) during a thermal psychophysical task. A TSA-II stimulator was used to deliver heat stimuli (less than or equal to 49°C). Participants also completed chronic pain history and catastrophizing questionnaires.

**Results**: Men *without* chronic pain had significantly higher *intensity* ratings than women without chronic pain, *t*(51) = 2.13, *p* = .038; however, men *with* chronic pain had *lower* intensity ratings than women with chronic pain. Among those reporting low pain catastrophizing, men with chronic pain had significantly lower *intensity* ratings than men without chronic pain; women with chronic pain had higher *unpleasantness* ratings than women without chronic pain.

Overall, women without chronic pain had the *lowest* intensity and unpleasantness ratings; men without chronic pain had the *highest* intensity and unpleasantness ratings.

**Discussion/Conclusions**: Results suggest complex interactions among sex, chronic pain status, and pain catastrophizing in relation to experimental pain responses. Furthermore, pain intensity and unpleasantness appear to capture unique aspects of the pain experience.

## Documenting Patient Experiences in a Community Interdisciplinary Pain Management Centre: A Qualitative Study at the Pain and Wellness Centre (PWC)

S. Fatima Lakha^a,*^, Alex Mailis^b^ and Angela Mailis^b,c,d^

^a^Research Department, Pain and Wellness Centre, Vaughan, Ontario, Canada; ^b^Department of Physical Medicine, Pain and Wellness Centre, Vaughan, Ontario, Canada; ^c^Department of Medicine, Division of Physical Medicine, University of Toronto, Vaughan, Toronto, Canada; ^d^Toronto Rehab Institute, University Health Network, Toronto, Ontario, Canada

**CONTACT** S.Fatima Lakha sfatima.lakha@utoronto.ca

© 2020 The Author(s). Published with license by Taylor & Francis Group, LLC.

This is an Open Access article distributed under the terms of the Creative Commons Attribution License (http://creativecommons.org/licenses/by/4.0/), which permits unrestricted use, distribution, and reproduction in any medium, provided the original work is properly cited.

**Introduction/Aim**: Understanding patients’ experiences in the environment they are treated is vital in order to improve care and management. The objective of this project is to describe chronic pain patients’ perceived impact of participation in a community interdisciplinary pain program (IDP). By sharing the experiences of these patients, we will inform ongoing quality improvement efforts and apprise others who are engaged in similar chronic pain programs.

**Methods**: Retrospective data were collected from 25 patients treated in the PWC IDP who provided written testimonials and/or google reviews between October 2016 and August 2019. Analysis was performed in a) descriptive demographic and pain data; and b) qualitative data extracted from patients’ written testimonials. The thematic analysis approach was used for the interpretation of qualitative data.

**Results**: Male/female ratio was 1:2.5 (*p* < .001) with mean age 48 ± 18 yrs. Regarding the participants 55% (*p* < .05) were married and 40% had completed college, university or post-graduate studies. The average duration of pain at time of IDP admission was 5 ± 5 years; 75% had two or more sites of pain. Coding of testimonials revealed a number of themes that were usefully organized in domains and (themes): a) patients’ experiences (pain management tools, interdisciplinary pain approach, patient center-care, expectations) and b) previous treatment vs interdisciplinary program care (physician-patient interaction, use of health resources, accurate diagnosis). Detailed results will be presented.

**Discussion/Conclusions**: Patient opinions are core for improving care. The results of the study will educate pain management teams in their pursue of patient-centered management strategies.

## Development of a Short-form Pain Resilience Scale

P. Maxwell Slepian^a,b,*^, Brett Ankawi^c^, Douglas J. French^d^, R. Thomas Evans^d^, Joel Katz http://orcid.org/0000-0002-8686-447X^a,b,e^ and Christopher R. France^f^

^a^Department of Anesthesia and Pain Medicine, Toronto General Hospital, Toronto, Ontario, Canada; ^b^Department of Psychology, York University, Toronto, Ontario, Canada; ^c^VA Connecticut Healthcare System, Pain Research, Informatics, Multi-morbidities, and Education Center, West Haven, Connecticut, USA; ^d^Atlantic Pain Clinic, Moncton, New Brunswick, Canada; ^e^Department of Anesthesia, University of Toronto, Toronto, Ontario, Canada; ^f^Department of Psychology, Ohio University, Athens, Ohio, USA

**CONTACT** P. Maxwell Slepian maxwell.slepian@uhn.ca

© 2020 The Author(s). Published with license by Taylor & Francis Group, LLC.

This is an Open Access article distributed under the terms of the Creative Commons Attribution License (http://creativecommons.org/licenses/by/4.0/), which permits unrestricted use, distribution, and reproduction in any medium, provided the original work is properly cited.

**Introduction/Aim**: The Pain Resilience Scale (PRS) is a validated 14-item measure capturing adaptive regulation of cognitions and emotions and continued behavioral engagement despite pain. The PRS has predicted psychological adjustment to pain and treatment outcomes; however, the clinical utility of the scale may be improved by the validation of a short-form that is easier to use in clinical settings.

**Methods**: Confirmatory factor analyses on 8-, 6-, and 4-item versions of the PRS were conducted in a sample of individuals with chronic pain who were recruited from Amazon’s Mechanical Turk (n = 1519, 54% female). Convergent validity with pain-related psychosocial measures (e.g. pain catastrophizing, pain-related fear, pain self-efficacy) was examined in the same sample. Predictive validity was examined in a sample of patients (n = 149, 51% female) undergoing an interdisciplinary functional restoration program for chronic pain.

**Results**: The 6-item (RMSEA = 0.027, 95%CI: 0.006, 0.046) was a superior fit compared to the 8- or 4-item PRS. The six–item PRS was highly correlated to the full measure (r = 0.97), demonstrated expected convergent validity with other pain-related psychosocial measures and predicted improvement in self-reported physical and mental health during the course of treatment, *p’s* < 0.05.

**Discussion/Conclusions**: The validity of a 6-item version of the PRS was supported by CFA and was associated with other psychosocial measures and treatment outcomes. Use of a shortened version of the PRS may help reduce patient burden and improve clinical utility of the scale.

## Predictors of Parents’ Insensitive Behaviors in Response to Infant Vaccination Pain

Shaylea Badovinac^a,*^, Hannah Gennis^a^, Rebecca Pillai Riddell^a^ and Hartley Garfield^b^

^a^Clinical-Developmental Psychology, York University, Toronto, Ontario, Canada; ^b^Pediatrics, University of Toronto, Toronto, Ontario, Canada

**CONTACT** Shaylea Badovinac sdbadov@yorku.ca

© 2020 The Author(s). Published with license by Taylor & Francis Group, LLC.

This is an Open Access article distributed under the terms of the Creative Commons Attribution License (http://creativecommons.org/licenses/by/4.0/), which permits unrestricted use, distribution, and reproduction in any medium, provided the original work is properly cited.

**Introduction/Aim**: Previous studies have shown strong relationships between infant pain-related distress and parents’ use of insensitive behaviors post-vaccination.^1,2^ The present study explored predictors of parents’ insensitive behavior in response to infant pain-related distress, focusing on parent psychopathology, parenting stress, pre-needle worry, and pre-needle physiological arousal, as well as infant pre-needle distress.

**Methods**: The study included parent-infant dyads (*n *= 56) from the 12-month wave of an ongoing longitudinal study (OUCH Cardio Cohort). Dyads were videotaped and connected to equipment that recorded their heart rate during infants’ routine vaccinations. Parent insensitive behaviors were coded for three minutes post-needle^2018^.^2^ Parents rated pre-needle worry on a Likert scale immediately before the needle. Parent psychopathology (PSI)^3^ and parenting stress (BSI) ^4^ were self-reported. Parent heart rate was averaged over the 30-second epoch pre-needle using MindWare software (HRV Analysis 3.1.3.). Infant pre-needle pain-related distress was coded for 15 seconds pre-needle using FLACC.^5^ A forced-entry regression model was used.

**Results**: The regression model accounted for a significant proportion of variance in parents’ insensitive behaviors (R^2^ =.35, *p *<.001). Infant pre-needle pain-related distress emerged as the only significant predictor of parent insensitive behaviors (β = 0.55, *p *<.001).

**Discussion/Conclusions**: Infants’ pre-needle distress uniquely predicted parents’ use of insensitive behaviors post-needle. Future studies should explore how different parent factors (e.g., psychopathology, stress) interact with infant pre-needle distress to predict parents’ use of insensitive behaviorsfm post-needle.

References1.Badovinac
S, Gennis
H, Riddell
RP, Garfield
H, Greenberg
S. Understanding the relative contributions of sensitive and insensitive parent behaviors on infant vaccination pain. Children. 2018;5(6):80. doi:10.3390/children5060080.PMC6025307299121772.Pillai Riddell
R, Gennis
H, Tablon
P, Greenberg
S, Garfield
H. Developing a measure of distress-promoting parent behaviors during infant vaccination: assessing reliability and validity. Can J Pain. 2018;2:135–144. doi:10.1080/24740527.2018.1471325.PMC8730610350053733.Abidin
RR. Parenting stress index.
4th ed. Lutz, FL: PAR; 2012.4.Derogatis
LR.
Brief symptom inventory (BSI)-18: administration, scoring and procedures manual. Minneapolis, MN: NCS Pearson; 2001.5.Merkel
F, Voepel
SI, Lewis
T, Shayevitz
JR, Malviya
S. The FLACC: a behavioral scale for scoring postoperative pain in young children. Pediatr. Nurs. 1997;23:293–297.9220806

## Sensitivity to Pain Traumatization is Associated with Slower Resolution of Acute Pain after Cardiothoracic Surgery

P. Maxwell Slepian^a,b,*^, Hance Clarke^a,c^, Aliza Weinrib^a,b^, Dorothy Wong^a^, George Djaiani^a,c^, Marcelo Cypel^d,e^, Vivek Rao^d,e^, Ze’Ev Seltzer^f^ and Joel Katz http://orcid.org/0000-0002-8686-447X^a,b,c^

^a^Department of Anesthesia and Pain Medicine, Toronto General Hospita, Toronto, Ontario, Canada; ^b^Department of Psychology, York University, Toronto, Ontario, Canada; ^c^Department of Anesthesia, University of Toronto, Toronto, Ontario, Canada; ^d^Department of Surgery, Toronto General Hospital, Toronto, Ontario, Canada; ^e^Department of Surgery, University of Toronto, Toronto, Ontario, Canada; ^f^Faculties of Dentistry and Medicine, University of Toronto, Toronto, Ontario, Canada

**CONTACT** P. Maxwell Slepian maxwell.slepian@uhn.ca

© 2020 The Author(s). Published with license by Taylor & Francis Group, LLC.

This is an Open Access article distributed under the terms of the Creative Commons Attribution License (http://creativecommons.org/licenses/by/4.0/), which permits unrestricted use, distribution, and reproduction in any medium, provided the original work is properly cited.

**Introduction/Aim**: The speed at which pain resolves after surgery is an important consideration in postoperative pain management. The current study examined the influence of psychosocial variables on day-to-day changes in pain intensity during hospitalization after major cardiothoracic surgery.

**Methods**: A prospective cohort of patients undergoing major cardiothoracic surgery (N = 949, 34.6% female) completed the Sensitivity to Pain Traumatization Scale (SPTS) and Mindful Attention and Awareness Scale (MAAS) prior to surgery. Maximum pain ratings (11-point NRS) for each day were recorded while the patient was in the hospital after surgery. Growth curve modeling was used to examine the pattern of changes in pain ratings over time and predictors of these changes. Age, sex, and ongoing pre-operative chronic pain were included as time-invariant covariates. Daily opioid use (in mg morphine equivalent, MME/day) was included as a time varying covariate.

**Results**: Unconditional growth curve analyses identified a logarithmic growth curve as better fitting than linear, quadratic, or cubic curves, ΔAIC/BIC > 10. On average, pain intensity decreased over time, b = −2.69, *p* < .001. Conditional analyses supported inclusion of psychosocial predictors in the model. Controlling for age, sex, and in-hospital morphine use, higher preoperative SPTS scores were associated with slower reduction in pain over time, b = 0.04, *p* = .007. Preoperative MAAS was unrelated to postoperative pain, *p* > .05.

**Discussion/Conclusions**: The results highlight psychosocial risk factors related to the dynamic experience of in-hospital postoperative pain. Targeted perioperative psychosocial interventions may speed up acute postoperative pain resolution.

## The Known Unknowns: A Scoping Review of Literature on Family Physicians’ Gaps in Knowledge and Skills for Managing Chronic Pain

Mihal Esterlis http://orcid.org/0000-0002-1249-0671^a,*^, Virginia McEwen^b^ and Anuj Bhatia^c,d^

^a^Ryerson University, Toronto, Ontario, Canada; ^b^Northern Ontario School of Medicine, Thunder Bay, Ontario, Canada; ^c^Department of Anesthesia and Pain Management; ^d^Toronto Western Hospital, University Health Network, Toronto, Ontario, Canada

**CONTACT** Mihal Esterlis michelleesterlis@gmail.com

© 2020 The Author(s). Published with license by Taylor & Francis Group, LLC.

This is an Open Access article distributed under the terms of the Creative Commons Attribution License (http://creativecommons.org/licenses/by/4.0/), which permits unrestricted use, distribution, and reproduction in any medium, provided the original work is properly cited.

**Introduction/Aim**: Primary care providers (PCPs), who provide the bulk of care for patients with chronic pain, often report knowledge gaps, limited resources, and difficult patient encounters while managing chronic pain. This scoping review seeks to evaluate the extent of PCPs’ identified knowledge/skill gaps in chronic pain management.

**Methods**: A broad literature search was conducted on PubMed and the gray literature for relevant articles in the area of knowledge and skill gaps of PCP for managing chronic pain, with multiple search term derivatives of both concepts. The initial search yielded 175 articles which were then screened for relevance, yielding 16 studies for inclusion.

**Results**: The 16 articles included in this review reflected a variety of study designs, settings, and methods (Table 1). Consistent themes emerged with respect to knowledge gaps in assessment, diagnosis, treatment, and allied health roles in chronic pain. A general lack of confidence in approaching discontinuation of high dose or ineffective opioid regimes, professional isolation and managing complex pain patients with limited access to pain specialists were also reported.

**Discussion/Conclusions**: Studies summarized in this review demonstrate commonalities that will be useful in creating a targeted, useful and concise curriculum for primary care providers. Findings from these studies will be used to generate a survey to elucidate priority topics for PCPs caring for chronic pain patients. The current scoping review synthesizes information from clinical studies that can inform us in creating a Pain Day for PCPs that will maximize learning and ultimately benefit chronic pain patients.

**Table 1.** A summary of types of study methodologies that assessed primary care providers’ deficits in knowledge and skills for chronic pain management.

Data collection method	Number
Survey	7
Focus group interviews	5
Reports of case-based discussion	1
Reports of workshops on chronic pain	1
RCT evaluating case-based e-mail discussion against no discussion	1
Pre-post intervention studies	2

## Biopsychosocial Characteristics and Healthcare Utilization in Veterans Seeking Care at an Interprofessional Chronic Pain Clinic

Etienne J. Bisson http://orcid.org/0000-0002-0649-3550^a,b,c,d^, Scott Duggan^a,d^, Mary Anne Good^a^, Mona Sawhney http://orcid.org/0000-0001-5399-1715^a,e^ and Rosemary Wilson http://orcid.org/0000-0003-3262-243X^a,d,e,*^

^a^Chronic Pain Clinic, Kingston Health Sciences Centre-Hotel Dieu Hospital Site, Kingston, Ontario, Canada; ^b^Centre for Neuroscience Studies, Queen’s University, Kingston, Ontario, Canada; ^c^School of Rehabilitation Therapy, Queen’s University, Kingston, Ontario, Canada; ^d^Department of Anesthesiology and Perioperative Medicine, Queen’s University, Kingston, Ontario, Canada; ^e^School of Nursing, Queen’s University, Kingston, Ontario, Canada

**CONTACT** Etienne Bisson etienne.bisson@kingstonhsc.ca

© 2020 The Author(s). Published with license by Taylor & Francis Group, LLC.

This is an Open Access article distributed under the terms of the Creative Commons Attribution License (http://creativecommons.org/licenses/by/4.0/), which permits unrestricted use, distribution, and reproduction in any medium, provided the original work is properly cited.

**Introduction/Aim**: The purpose of this study was to identify if there were differences in biopsychosocial characteristics and health care utilization between veterans and adult civilians with chronic pain seeking care at an interprofessional chronic pain clinic.

**Methods**: Patient-reported baseline data between November 2017 and October 2019 were extracted from the Kingston Health Sciences Center Chronic Pain Registry. Veterans were identified and age/sex-matched with civilians randomly selected (1:2 case-control ratio). Descriptive and comparative statistics were completed for several biopsychosocial measures (e.g. pain severity, pain catastrophizing, fear of movement, suicidal ideation, depression, quality of life) and healthcare utilization.

**Results**: Veterans (n = 47) were 79% male with a mean age of 57 ± 14. Compared to age/sex matched civilians (n = 94), more veterans reported having suicidal ideation (23% vs 8.6%). The biopsychosocial measures examined were not clinically different between groups. Veterans utilized more community allied health care such as chiropractor, massage therapist, physiotherapist and psychologist compared to civilians (mean of 25.0 ± 38.0 vs 4.74 ± 8.18 visits in the previous year).

**Discussion/Conclusions**: Despite heavier utilization of community allied health care by veterans, our results show that this population present similar biopsychosocial characteristics but higher suicidal ideation than the civilian population at enrollment into an interprofessional chronic pain clinic. Findings may reflect that siloed multidisciplinary care for pain management in veterans has no additional benefits. More research is warranted to examine how veterans may benefit from an interprofessional chronic pain program that is evidenced based.

## Pain Flashbacks and Their Relationship to Comorbid Posttraumatic Stress Disorder and Chronic Pain: A Review

Larah Maunder^a,*^, Julia Moreau^a^, Joel Katz http://orcid.org/0000-0002-8686-447X^b^ and Tim Salomons^a^

^a^Psychology, Queen’s University, Kingston, Ontario, Canada; ^b^Psychology, York University, Toronto, Ontario, Canada

**CONTACT** Larah Maunder larah.maunder@queensu.ca

© 2020 The Author(s). Published with license by Taylor & Francis Group, LLC.

This is an Open Access article distributed under the terms of the Creative Commons Attribution License (http://creativecommons.org/licenses/by/4.0/), which permits unrestricted use, distribution, and reproduction in any medium, provided the original work is properly cited.

**Introduction/Aim**: There is a high co-occurrence of posttraumatic stress disorder (PTSD) and chronic pain, yet the mechanisms behind this relationship are poorly understood. This has led to poor treatment outcomes for patients. Beyond occurring together, it is possible that PTSD and chronic pain might interact and aggravate each other through a common traumatic symptom: “pain flashbacks,” a phenomenon in which individuals report reexperiencing the physical pain they felt during a traumatic injury.

**Methods**: This review summarizes the empirical literature on pain flashbacks, including their prevalence and a theoretical analysis of their mechanistic role in exacerbating and maintaining chronic pain and PTSD. Neurobiological and psychological hypotheses are presented to explain the etiology and maintenance of pain flashbacks. A discussion of the meaning of pain flashbacks is presented, emphasizing how individuals attribute their current pain to the pain experienced during the traumatic exposure.

**Discussion/Conclusions**: Important candidates for the etiology of pain flashbacks include anxiety sensitivity, fear and avoidance behaviors, and the encoding of pain memories at the somatosensory level. Pain flashback prevalence rates documented in the recent literature (49%) are likely an overestimate due to the inclusion of clinical samples and use of a screening tool to determine symptom presence. Future studies will require semi-structured interviews sampled from more diverse populations to determine prevalence. We conclude with recommendations for future research, and discuss treatment implications for individuals who experience pain flashbacks as part of their chronic pain and PTSD symptom profile. This may include psychotherapy, such as interoceptive exposure, targeting fear and avoidance symptoms.

## Beta-arrestin2-mediated Nuclear Signaling in Inflammatory Pain

Ahmed Hassan^a,*^, Mircea Iftinca^a^, Daniel Young^a^, Robyn Flynn^a^, Francina Agosti^a^, Manon Defaye^a^, Nasser Abdullah^a^, Morley Hollenberg^a^, Antoine Dufour^a^ and Christophe Altier^a^

^a^Physiology and Pharmacology, University of Calgary, Calgary, Alberta, Canada

**CONTACT** Ahmed Hassan ahmed.hassan1@ucalgary.ca

© 2020 The Author(s). Published with license by Taylor & Francis Group, LLC.

This is an Open Access article distributed under the terms of the Creative Commons Attribution License (http://creativecommons.org/licenses/by/4.0/), which permits unrestricted use, distribution, and reproduction in any medium, provided the original work is properly cited.

**Introduction/Aim**: Transient receptor potential vanilloid type 1 (TRPV1) are nonselective cation channels that detect chemical and physical painful stimuli. Our lab has showed that TRPV1 activation induced the shuttling of b-arrestin2 (ß-arr2) to the nucleus. The Aim of this study is to determine the role of ß-arr2-mediated signal transduction in the nucleus with regard to pain sensation.

**Methods**: Nuclear proteins interacting with the WT ß-arr2-YFP or the nucleus sequestered ß-arr2-L395Q-YFP mutant were identified by immunoprecipitation followed by mass spectrometry in transfected HEK293 cells. Protein-protein interactions were validated using co-immunoprecipitation and immunocytochemistry in HEK and sensory dorsal root ganglia neurons.

**Results**: We identified 559 proteins that we categorized in 7 distinct functional groups: 1. Ribosomal biogenesis, 2. Gene transcription, 3. Pre-mRNA processing, 4. Cell division, 5. DNA damage/repair, 6. nucleocytoplasmic transport machinery and 7. Miscellaneous functions. A comparative/quantitative analysis between WT and mutant identified TCOF1 and RNA Polymerase 1, proteins that are involved in ribosomal biogenesis. Association was confirmed using co-immunoprecipitation and immunocytochemistry from HEK and DRGs.

**Discussion/Conclusions**: Interactions of ß-arr2 with proteins involved in ribosomal biogenesis suggest the involvement of ß-arr2 in regulating protein synthesis in response to noxious stimuli in sensory neurons. The control of mRNA translation by ß-arr2 in DRG neurons could contribute to long-term neuronal plasticity that can lead to the transition from acute to chronic pain states in inflammation.

## Skin-resident Dendritic Cells Control Sensory Neuron Activation through the CCL22-CCR4 Axis in Postoperative Pain

Jaqueline Raymondi Silva^a^, Mircea Iftinca^b^, Courtney Bannerman^c^, Isaac F Gomes^d^, Thiago M. Cunha^d^, Ian Gilron^a^, Christophe Altier^b^, Nader Ghasemlou^a,*^

^a^Anesthesiology and Perioperative Medicine, Queen’s University, Kingston, Ontario, Canada; ^b^Physiology and Pharmacology, University of Calgary, Calgary, Alberta, Canada; ^c^Biomedical and Molecular Sciences, Queen’s University, Kingston, Ontario, Canada; ^d^Pharmacology, University of Sao Paulo, Ribeirao Preto, São Paulo, Brazil

**CONTACT** Jaqueline Raymondi Silva jrs5@queensu.ca

© 2020 The Author(s). Published with license by Taylor & Francis Group, LLC.

This is an Open Access article distributed under the terms of the Creative Commons Attribution License (http://creativecommons.org/licenses/by/4.0/), which permits unrestricted use, distribution, and reproduction in any medium, provided the original work is properly cited.

**Introduction/Aim**: Inflammatory pain occurs as a result of interactions between the immune and nervous systems, which includes the activation of tissue-resident immune cells in the site of injury. We therefore sought to examine the role of dendritic cells and its mediators CCL17 and CCL22 in the development of inflammatory pain.

**Methods**: Male C57BL/6J mice were used for all experiments. Plantar incisional wound was used to model pain. Behavior was assessed using the von Frey assay, Hargreaves and acetone tests. The antagonist C 021 was used to block CCR4 or knockout mice were used to assess loss of receptor function. γδ T cell-null and CD11cDTR mice were also used to assess whether CCR4+ cells contribute to pain outcomes.

**Results**: CCL17/22 are upregulated after tissue injury by dendritic cells. Both chemokines elicit mechanical and thermal hypersensitivity when administered subcutaneously, a response abrogated by pharmacological blockade of CCR4 using the antagonist C 021. Calcium imaging of dissociated sensory neurons from naïve and postoperative mice showed that CCL22, but not CCL17, was able to directly activate neurons; electrophysiological recordings demonstrated that sensory neurons are sensitized to CCL22 after injury. These responses were blocked using C 021. Finally, our data show that acute post-surgical pain is reduced in mice lacking CCR4, wildtype animals treated with CCR4 antagonist, as well as transgenic mice depleted of dendritic cells.

**Discussion/Conclusions**: These results suggest a role for the CCL22:CCR4 axis in the genesis of inflammatory pain via direct communication between dendritic cells and sensory neurons, opening new therapeutic avenues for its control.

## The Effects of Numerical Anchoring and Pain Relevance of Viewed Images on Pain Ratings

Rebecca E. Lewinson http://orcid.org/0000-0001-7157-2784^a,*^ and Joel D. Katz http://orcid.org/0000-0002-8686-447X^a^

^a^Department of Psychology, York University, Toronto, Ontario, Canada

**CONTACT** Rebecca Lewinson lewinson@yorku.ca

© 2020 The Author(s). Published with license by Taylor & Francis Group, LLC.

This is an Open Access article distributed under the terms of the Creative Commons Attribution License (http://creativecommons.org/licenses/by/4.0/), which permits unrestricted use, distribution, and reproduction in any medium, provided the original work is properly cited.

**Introduction/Aim**: Pain is a subjective experience, and as such, it is important to determine the factors that influence how pain is interpreted by others, as these pain inferences can impact how and when a patient receives treatment. Numeric anchoring is a cognitive bias whereby exposure to a numeric quantity influences subsequent judgments involving other quantities. Anchoring effects have previously been shown to influence how a patient’s pain intensity is perceived. This study aims to better understand the variables involved in pain inferences by using pain-relevant and pain-irrelevant images in conjunction with numeric anchors to induce the anchoring effect.

**Methods**: 590 participants read a vignette describing a patient with chronic pain before being randomized to one of six groups (2 × 3 design). Participants viewed a pain-relevant (car crash) or pain-irrelevant (house) image which displayed a scrolling header containing either a high number (NEWS 98), a low number (NEWS 2) or no number (NEWS). Participants were then asked to estimate the patient’s pain intensity on a 0–100 numeric rating scale.

**Results**: There were no significant differences between groups in pain intensity ratings *H*(5) = 5.198, *p* = .392. Neither the photos, *H*(1) = 2.355, *p* = .125, nor the numerical anchors, *H*(2) = 0.088, *p* = .957 influenced subsequent pain ratings.

**Discussion/Conclusions**: Pain ratings were not influenced by prior exposure to a numerical anchor (high, low, none) or the relevance of an image to pain (relevant, irrelevant). Future studies are needed to better understand the anchoring effect and the factors that influence pain inferences.

## Parent and Child Catastrophizing about Each Other’s Pain in Parents with Chronic Pain and Their Children

Kristen S. Higgins^a,*^, Christine T. Chambers^b^, Natalie O. Rosen^c^, Simon Sherry^d^, Somayyeh Mohammadi^e,f^, Mary E. Lynch^g^, Marsha Campbell-Yeo http://orcid.org/0000-0001-6645-2809^h^ and Alexander J. Clark^g^

^a^Department of Psychology & Neuroscience, Dalhousie University and Centre for Pediatric Pain Research, IWK Health Centre, Halifax, Nova Scotia, Canada; ^b^Departments of Pediatrics and Psychology & Neuroscience, Dalhousie University and Centre for Pediatric Pain Research, IWK Health Centre, Halifax, Nova Scotia, Canada; ^c^Department of Psychology & Neuroscience, Dalhousie Universityand Obstetrics & Gynecology, IWK Health Centre, Halifax, Nova Scotia, Canada; ^d^Department of Psychology & Neuroscience, Dalhousie University, Halifax, Nova Scotia, Canada; ^e^Centre for Pediatric Pain Research, IWK Health Centre, Halifax, Nova Scotia, Canada; ^f^Department of Occupational Science and Occupational Therapy, University of British Columbia, Vancouver, British Columbia, Canada; ^g^Department of Anesthesia, Pain Management & Perioperative Care, Dalhousie Universityand Pain Management Unit, QEII Health Sciences Centre, Halifax, Nova Scotia, Canada; ^h^School of Nursing, Dalhousie Universityand Centre for Pediatric Pain Research, IWK Health Centre, Halifax, Nova Scotia, Canada

**CONTACT** Kristen S. Higgins Kristen.higgins@dal.ca

© 2020 The Author(s). Published with license by Taylor & Francis Group, LLC.

This is an Open Access article distributed under the terms of the Creative Commons Attribution-NonCommercial License (http://creativecommons.org/licenses/by-nc/4.0/), which permits unrestricted non-commercial use, distribution, and reproduction in any medium, provided the original work is properly cited.

**Introduction/Aim**: Research has established that children’s catastrophizing about their own pain is associated with poorer child pain outcomes. For children of parents with chronic pain, catastrophizing about parental pain might uniquely predict child pain due to increased exposure to parents’ chronic pain and disability. This study examined dyadic associations between child and parent catastrophizing about one another’s pain and child pain during the cold pressor task (CPT).

**Methods**: As part of a larger project, 72 parents with chronic pain and one of their children (ages 8–15) completed questionnaires measuring trait catastrophizing about their own and each other’s pain. Children completed the CPT and parents and children rated the child’s worst pain intensity during the task. Analyses were guided by the Actor-Partner Interdependence Model.

**Results**: Higher levels of child and parent catastrophizing about their own pain were associated with greater catastrophizing about one another’s pain (b_child_ = 0.63, *SE *= 0.10, *p* < .001; b_parent_ = 0.49, *SE *= 0.08, *p* < .001). Greater parent catastrophizing about their own pain was associated with greater child catastrophizing about parent pain (b = 0.20, *SE *= 0.10, *p* < .05). Higher child catastrophizing about parent pain was associated with greater self- (b = 0.05, *SE *= 0.03, *p* < .05) and parent-reported (b = 0.05, *SE *= 0.02, *p* < .05) child CPT pain intensity, beyond associations with catastrophizing about their own pain. Higher parent catastrophizing about child pain was associated with lower levels of child self-reported (b = −0.08, *SE *= 0.03, *p* < .05), but not parent-reported (b = 0.04, *SE *= 0.03, *p* > .05), CPT pain intensity.

**Discussion/Conclusions**: Child catastrophizing about parental chronic pain may be a vulnerability factor associated with poorer child pain outcomes and should be considered in future studies and clinical practice.

## Investigating Chronic Pain Management Among Emerging Adults

Rachel Ellingson^a^, Etienne J. Bisson^b^, Rosemary Wilson http://orcid.org/0000-0003-3262-243X^a,c^ and Catherine Goldie^a,*^

^a^School of Nursing, Queen’s University, Kingston, Ontario, Canada; ^b^Chronic Pain Clinic, Kingston Health Sciences Centre-Hotel Dieu Hospital Site, Kingston, Ontario, Canada; ^c^KHSC Chronic Pain Clinic, Kingston, Ontario, Canada

**CONTACT** Rachel Ellingson 17rt6@queensu.ca

© 2020 The Author(s). Published with license by Taylor & Francis Group, LLC.

This is an Open Access article distributed under the terms of the Creative Commons Attribution License (http://creativecommons.org/licenses/by/4.0/), which permits unrestricted use, distribution, and reproduction in any medium, provided the original work is properly cited.

**Introduction/Aim**: Emerging adults (EA) with chronic pain (CP) struggle to achieve age-specific expectations due to physical limitations resulting from their pain. Those accessing CP services are frequently viewed as synonymous with the general adult population (ages 18–64), despite significant variation in the relationship they have with their pain condition and societal pressures to achieve stable employment, financial independence and establish social relationships. This study investigated characteristics of EA accessing a specialized CP clinic and describes interventions offered and utilized by this group.

**Methods**: A retrospective chart review was conducted between 2017–18, 41 EA’s (aged 18–29) and 41 middle aged adults (MA’s) (aged 30–64) receiving care from a CP clinic in Southeastern Ontario were examined over six months. Groups were matched on sex and number of pain sites. Demographic and pain characteristics, interventions, referrals, and clinic utilization were examined using bivariate and multivariate analysis.

**Results**: MA’s reported higher pain severity scores, *t*(80) = − 2.15, *p* = .035. EA’s more frequently received referrals for additional consultation and/or diagnostic investigations (*X*^2^ (1, N = 82) = 4.97, *p* = .026) and were more likely to have at least one psychology visit (*X*^2 ^= 7.29, *p* = .007). Multivariate analysis identified EA’s with higher patient health questionnaire scores for depression were more likely to see a psychologist (OR 1.23, 95%CI 1.014–1.492).

**Discussion/Conclusions**: Findings of this study inform our understanding of characteristics of EA’s who utilize CP services and which services are offered and utilized by them. Further research is needed to better understand the efficacy of treatments offered and satisfaction with pain management approaches.

## Positive, but Not Negative, Pain-related Expectancies Mediate the Relationship between Pain Catastrophizing and Long-term Pain

Catherine Paré^a,*^, Junie S. Carrière^b^ and Michael J.L. Sullivan^a^

^a^Psychology, McGill University, Montréal, Québec, Canada; ^b^Anaesthesiology, Brigham and Women’s Hospital Pain Management Center, Harvard Medical School, Chestnut Hill, Massachusetts, USA

**CONTACT** Catherine Paré catherine.pare2@mail.mcgill.ca

© 2020 The Author(s). Published with license by Taylor & Francis Group, LLC.

This is an Open Access article distributed under the terms of the Creative Commons Attribution License (http://creativecommons.org/licenses/by/4.0/), which permits unrestricted use, distribution, and reproduction in any medium, provided the original work is properly cited.

**Introduction/Aim**: Pain catastrophizing has been broadly defined as an excessive negative cognitive orientation toward painful stimuli. Implicit in this definition is that negative outcome expectancies might be the vehicle through which catastrophizing might contribute to chronic pain. Considerable research has shown that “less positive” outcome expectancies are prospectively related to problematic recovery. However, the prognostic value of negative outcome expectancies has yet to be investigated. This study examined the role of negative and positive outcome expectancies as mediators of the relationship between catastrophizing and pain experience.

**Methods**: 159 individuals with work-related musculoskeletal injuries were recruited from one of 6 primary care physiotherapy clinics in the greater Montreal region.

**Results**: Our study replicated previous research findings that pain catastrophizing predicts long-term pain severity. Our findings revealed that lower positive expectancies, but not higher negative expectancies, partially mediated the relationship between pain catastrophizing and long-term pain.

**Discussion/Conclusions**: Conceptual frameworks have emphasized negative outcome expectancies as a central feature of pain catastrophizing. Although the results of the present study revealed a significant correlation between pain catastrophizing and negative outcome expectancies, negative outcome expectancies did not mediate the relation between pain catastrophizing and long-term pain. The findings suggest that positive outcome expectancies might serve a preventive function in relation to long-term pain. Efforts to reduce the incidence of chronic pain might benefit more from techniques aimed at increasing positive outcome expectancies than techniques aimed at decreasing negative outcome expectancies.

## The Immediate Effect of Combined Lidocaine and Ketamine Infusion on Neuropathic Pain: A 5-year Retrospective Observational Study

Ramin Safakish^a,*^, Shadi Babazadeh^a^ and Tina Emadi^a^

^a^Allevio Pain Management, Toronto, Ontario, Canada

**CONTACT** Ramin Safakish Ramin.Safakish@AllevioClinic.com

© 2020 Allevio Pain Management. Published with license by Taylor & Francis Group, LLC.

This is an Open Access article distributed under the terms of the Creative Commons Attribution License (http://creativecommons.org/licenses/by/4.0/), which permits unrestricted use, distribution, and reproduction in any medium, provided the original work is properly cited.

**Introduction/Aim**: Chronic pain is the leading cause of disability worldwide and costs our healthcare systems more than any other practice area. This study observes the relationship between an infused dose of lidocaine and ketamine, and the extent of immediate pain relief in patients with neuropathic pain (including diabetic neuropathy), fibromyalgia, and headache.

This is a retrospective cohort review, conducted in a single outpatient center.

This project has been conditionally approved by Veritas IRB Inc.

**Methods**: 670 subjects (508 females, 162 males) were treated at Allevio Pain Management by Dr. Safakish from March 2013 to May 2017, were included. In total, we analyzed 670 patients and 3,741 infusions.

**Results**: The mean age was 53.2 ± 13.4, mean weight (kg) was 80.7 ± 20.3. Half of the patients had neuropathic pain (51.8%), followed by fibromyalgia (40.9%), complex regional pain syndrome (CRPS) (6.2%), and chronic headache (1.0%).

The median number of infusions was 3. 38% of patients received more than five infusions for their condition. Univariable analysis showed that only lidocaine and ketamine dosages had a statistically significant impact on pain improvement.

**Conclusions**: We have shown that in outpatients with chronic neuropathic pain, the immediate analgesic effects of intravenous lidocaine and ketamine are impressive. The impact of lidocaine dosage (mg/kg) on pain relief showed borderline significance, while an increase in ketamine dosage (mg) was still associated with clinically meaningful pain relief.

## Parent Perceptions of the Use of Electroencephalogram for Neonatal Pain Measurement: Findings from a Randomized Controlled Trial

Britney Benoit^a,*^, Margot Latimer^b^, Aaron Newman^c^, Ruth Martin-Misener^b^ and Marsha Campbell-Yeo^b^

^a^Rankin School of Nursing, St. Francis Xavier University, Antigonish, Nova Scotia, Canada; ^b^School of Nursing, Dalhousie University, Halifax, Nova Scotia, Canada; ^c^Department of Psychology & Neuroscience, Dalhousie University, Halifax, Nova Scotia, Canada

**CONTACT** Britney Benoit bbenoit@stfx.ca

© 2020 The Author(s). Published with license by Taylor & Francis Group, LLC.

This is an Open Access article distributed under the terms of the Creative Commons Attribution License (http://creativecommons.org/licenses/by/4.0/), which permits unrestricted use, distribution, and reproduction in any medium, provided the original work is properly cited.

**Introduction/Aim**: Measurement of infant pain using neurophysiological imaging methods is an emergent area of research, yet, no studies have examined parent perceptions of the use of this technology. This paper reports on parent perceptions of 1) the use of neonatal electroencephalogram (EEG) for pain measurement, and 2) the use of breastfeeding and 24% oral sucrose for neonatal pain management during heel lance.

**Methods**: A sample of mothers (*n* = 36) who consented to their newborns taking part in a randomized controlled trial examining the influence of breastfeeding and 24% oral sucrose on pain-related EEG activity completed a study-specific questionnaire.

**Results**: Mothers reported positive perceptions of the use of EEG for pain measurement, with the majority reporting they felt good or very good (*n* = 33, 91.7%) about their baby wearing the EEG net. Six mothers (16.7%) indicated that they had concerns about their newborn’s comfort and safety prior to EEG application. All (*n* = 36) mothers perceived that breastfeeding and 24% oral sucrose provided adequate pain relief.

**Discussion/Conclusions**: In this sample of mothers who agreed to enroll their infant in an EEG study, there were positive perceptions regarding the use of EEG and the pain-reducing efficacy of the interventions. Studies using EEG for pain measurement must prioritize newborn comfort and safety. Examination of the perceptions of parents who declined to have their infant take part in the EEG study is needed to inform engagement of representative samples in future research.

## The Effect of Prolotherapy for Joint Pain on Pain Control & Quality of Life Improvement - 6 Years Retrospective Observational Study

Imrat Sohanpal^a,*^, Ramin Safakish^a^, Shadi Babazadeh^a^

^a^Allevio Pain Management, Toronto, Ontario, Canada

**CONTACT** Imrat Sohanpal Imrat.Sohanpal@AllevioClinic.com

© 2020 The Author(s). Published with license by Taylor & Francis Group, LLC.

This is an Open Access article distributed under the terms of the Creative Commons Attribution License (http://creativecommons.org/licenses/by/4.0/), which permits unrestricted use, distribution, and reproduction in any medium, provided the original work is properly cited.

**Introduction/Aim**: Extra-articular elements such as ligamentous injuries and enthesopathy are likely the most frequent sources of joint dysfunction and potential hypermobility.

Prolotherapy is not a new treatment in medicine.

**Methods**: This is a retrospective single center observational study of 266 patients who had been diagnosed with joint dysfunction and had prolotherapy from 2013–2018 in Allevio Pain Management.

This project has been conditionally approved by Veritas IRB Inc.

**Results**: A total of 246 patients (186 females, 60 males) that underwent 2–7 prolotherapy sessions were recruited for this study. The mean age was 54.2 years. According to our data, at the time of prolotherapy, 156 (65.5%) of patients were in working age From those 156 patients, 99 (63.4%) returned to work after intervention. 68 patients reported usage of narcotics before treatment (dosage 0.18– 27 mg) a significant reduction in narcotic use after intervention was observed (*p*-value<0.001).

With respect to non-narcotic pain drugs, 84 (34.1%) patients reported a decrease in use, 49.2% reported no change

A total of 178 patients (72.3%) had a reduction of 50% or more in at least one BPI item, and 100 patients (40.6%) had a reduction of 50% or more in all BPI items, which was statistically significant.

Using chiropractor or osteopath services which is common in SI joint dysfunction did not show any significant improvement.

**Discussion/Conclusions**: This study had shown a statistically and clinically significant improvement in BPI, reduction in narcotic consumption, and return to work after treatment.

## Role of Neuronal FAM150b in CFA-induced Inflammatory Pain

Nasser Abdullah^a,*^, Vinícius M. Gadotti^a^, Francina Agosti^a^, Manon Defaye^a^, Mircea Iftinca^a^, Gerald Zamponi^a^ and Christophe Altier^a^

^a^Department of Physiology and Pharmacology, University of Calgary, Calgary, Alberta, Canada

**CONTACT** Nassar Abdullah nasser.abdullah@ucalgary.ca

© 2020 University of Calgary. Published with license by Taylor & Francis Group, LLC.

This is an Open Access article distributed under the terms of the Creative Commons Attribution License (http://creativecommons.org/licenses/by/4.0/), which permits unrestricted use, distribution, and reproduction in any medium, provided the original work is properly cited.

**Introduction/Aim**:FAM150b was recently identified as an endogenous ligand of the orphan anaplastic lymphoma kinase receptor (ALK). The expression of ALK is confined to the developing nervous system as well as in innate immune cells where it is involved in proinflammatory responses to infections. Moreover, ALK mutations have been identified in cancers including neuroblastomas, which account for 10% of pediatric cancers. We found that expression of FAM150b was increased in TRPV1+ nociceptors during CFA inflammation, suggesting a role of neuronal FAM150b-ALK signaling in the control of inflammatory pain.

**Methods**: Transgenic mice that express a GFP conjugated to the TRPV1 channel were used to isolate TRPV1+ neurons following CFA induced inflammation by FACS followed by GeneChip microarray analysis. Inhibition of ALK using crizotinib (i.t.) or lorlatinib (i.t. and i.g.) was tested in the CFA and the formalin models of inflammatory pain. Thermal hyperalgesia post CFA injection was measured using the Hargreaves test. Nociceptive behaviors (paw licking and flinching) were scored in response to formalin.

**Results**: Fam150b is upregulated in nociceptors during inflammation. Acute ALK inhibition by crizotinib or lorlatinib reduced CFA-induced thermal hyperalgesia as well as formalin-induced inflammatory pain.

**Discussion/Conclusions**: FAM150b released from TRPV1+ nociceptors promote thermal hyperalgesia during CFA inflammation. Blocking the ALK receptor caused anti-nociceptive effects. Future experiments will determine where FAM150b is acting in the pain pathway and whether it modulates neurons or glial cells in the spinal cord. Identifying the cellular targets of FAM150b could lead to further insights into the role played by nociceptors in pain, inflammation and cancer.

## Caregiver Psychological Distress is Related to Toddler’s Physiological Responses to Pain

Miranda G. DiLorenzo^a,*^, Jordana Waxman^a^, Rebecca Pillai Riddell http://orcid.org/0000-0003-3990-3680^a^, Daniel Flanders^b^, Eitan Weinberg^b^ and Hartley Garfield^c^

^a^Psychology, York University, Toronto, Ontario, Canada; ^b^Kindercare Pediatrics, Toronto, Ontario, Canada; ^c^Pediatrics, University of Toronto, Toronto, Ontario, Canada

**CONTACT** Miranda G. DiLorenzo mgdilo@yorku.ca

© 2020 The Author(s). Published with license by Taylor & Francis Group, LLC.

This is an Open Access article distributed under the terms of the Creative Commons Attribution License (http://creativecommons.org/licenses/by/4.0/), which permits unrestricted use, distribution, and reproduction in any medium, provided the original work is properly cited.

**Introduction/Aim**: To examine the relationships between caregiver psychological distress and the physiological pain-related distress response of their toddlers during 12-, and 18-month vaccinations.

**Methods**: Caregiver-toddler dyads were followed as part of an ongoing longitudinal study at 12-, 18-, and 24-month vaccinations. Only data from 12- (*n* = 112) and 18-months (*n* = 81) will be reported in the current investigation. Toddler heart rate (HR) and heart rate variability (HRV) were analyzed during sequential 30-second epochs (30 seconds before the needle, immediately after the needle, 1-minute post-needle, and 2-minutes post-needle). Caregiver psychological distress was measured according to different subscales (Somatization, Obsessive-Compulsive, Interpersonal Sensitivity, Depression, Anxiety, Hostility, Phobic, Paranoid and Psychoticism) on the Brief Symptoms Inventory (BSI; Derogatis, 2000).

**Results**: There were no significant correlations between toddler cardiac indicators and BSI subscale scores at 12-months of age. At 18-months, negative correlations emerged between caregiver hostility and toddler heart rate across baseline and post-needle epochs (*r* = −.26 to −.32, *p* <.05). Significant positive correlations were also found between caregiver hostility and toddler HRV across post-needle time points at 18-months (*r* =.26 –.29, *p* <.05).

**Discussion**: Our findings suggest that caregivers who report more hostility have children who display a pattern of hyporesponsive (lower heart rate and higher heart rate variability) in the vaccination context. Null findings at the 12-month vaccination suggest that impact of parent psychological distress on toddler physiological reactivity and regulation in the vaccination context may not manifest until after 12 months of age.

## Kinesiophobia and Activity Limitation after Multidisciplinary Rehabilitation in Patients with Chronic Pain: Further Examination of the Fear-avoidance Model, a Replication

Eleni G. Hapidou^a,*^ and Myranda Rocha^a^

^a^Psychology, Neuroscience and Behavior, McMaster University, Hamilton, Ontario, Canada

**CONTACT** Eleni G. Hapidou hapidou@hhsc.ca

© 2020 The Author(s). Published with license by Taylor & Francis Group, LLC.

This is an Open Access article distributed under the terms of the Creative Commons Attribution License (http://creativecommons.org/licenses/by/4.0/), which permits unrestricted use, distribution, and reproduction in any medium, provided the original work is properly cited.

**Introduction/Aim**: To examine changes in kinesiophobia in relation to activity limitation after a multidisciplinary rehabilitation program in patients with heterogeneous chronic pain.

**Methods**: This is a prospective cohort study of 100 patients who completed an interdisciplinary chronic pain management program. Data from baseline (T_0_), after four weeks of rehabilitation (T_1_), and at 6-months follow-up (T_2_) will be analyzed. Outcome measures are the Tampa Scale for kinesiophobia (TSK-11) (Hapidou, O’Brien, Pierrynowski, de las Heras, Patel & Patla, 2012), and the Patient Disability Index (PDI) (Pollard, 1984). The relationship between kinesiophobia and activity limitation will be explored with regards to subgroups with high, medium and low baseline TSK scores, and for those patients who did or did not reach the smallest detectable change (SDC) in TSK. This is a partial replication of a study by Bergsten, Lundberg, Lindberg and Elfving (2012).

**Results**: Based on common findings in our Program, we expect kinesiophobia to decrease from baseline to after rehabilitation, and to stay relatively stable at the 6-months follow-up point. The PDI is also expected to follow a similar pattern.

**Discussion/Conclusions**: If the predictions are true, individuals with chronic pain can consider multidisciplinary rehabilitation programs to decrease kinesiophobia and activity limitations and for these changes to persist after the end of the Program. These results can also be useful to therapists in clinical settings who provide rehabilitation and pain management therapies.

## The Effects of Music Attributes on Pain Perception

Darius Valevicius^a,*^, Anna Bendas^b^, David Greenberg^c^ and Mathieu Roy^b^

^a^Department of Cognitive Science, McGill University, Montreal, Quebec, Canada; ^b^Department of Psychology, McGill University, Montreal, Quebec, Canada; ^c^Department of Psychology, Bar-Ilan University, Ramat-Gan, Tel Aviv, Israel

**CONTACT** Darius Valevicius darius.valevicius@mail.mcgill.ca

© 2020 The Author(s). Published with license by Taylor & Francis Group, LLC.

This is an Open Access article distributed under the terms of the Creative Commons Attribution License (http://creativecommons.org/licenses/by/4.0/), which permits unrestricted use, distribution, and reproduction in any medium, provided the original work is properly cited.

Music has been shown to reduce pain in a variety of contexts, including chronic pain, surgical operations, and experimental research. However, music is incredibly diverse, and we do not yet know which types or styles contribute more than others to the reduction of pain. Previous research has failed to differentiate music into different categories, in part because an appropriate musical typology has not been available. To answer this question, we adopted the “Arousal, Valence, Depth” model of music attributes, which locates songs along three psychological dimensions. We presented a set of musical excerpts to 60 healthy participants concurrently with painful thermal stimulations and showed that low arousal and low valence significantly reduced pain. High depth also reduced pain in subjects with a “systematizing” cognitive style. This research is a first step in uncovering the mechanisms of music-induced analgesia and may inform clinics and therapists as to the best music to use for alleviating pain.

**Introduction/Aim**: To test the effectiveness of music in reducing pain based on three dimensions: Arousal (stimulating-calming), valence (positive-negative emotion), and depth (complex-simple).

**Methods**: 60 healthy participants participated in the study. First, 28 thermal stimulations were delivered via a TSA-II Medoc thermode to determine their thermal pain response curve. This was used to compute a temperature corresponding to a subjective pain rating of 50/100, which was used throughout the rest of the experiment. Subjects were then presented with 45 clips of 40 seconds in length concurrently with 8-second thermal stimulations, which co-terminated with the clips. 27 clips were songs varying along the arousal, valence, and depth dimensions, 9 clips were controls consisting of scrambled music, and 9 were silent controls. For each trial, patients rated the amount of pain they experienced in response to the stimulation.

**Results**: Arousal was positively related to pain (β = 1.14, SE = 0.48, t = 2.37, df = 60, *p* < .05) as was valence (β = 0.79, SE = 0.36, t = 2.21, df = 379, *p* < .05), meaning that low arousal and low valence music performed better in reducing pain. Depth was significantly moderated by systematizing (β = − 0.96, SE = 0.36, t = − 2.67, df = 1375, *p* < .01), such that high systematizers experienced less pain in response to high depth music.

**Discussion/Conclusions**: The results indicate that music may reduce pain via multiple pathways. Low arousal in music might result in a state of mental or physiological relaxation, which could help reduce the sensation of pain. The effect of low valence (i.e. sad or melancholic) music is more difficult to explain, but it may be due to a mood-congruence effect or the emotionally soothing property that is anecdotally characteristic of sad music. High depth may reduce pain through cognitive engagement or distraction, which may be particularly salient for people with a systematizing cognitive style who are more prone to attend to the mechanics and structural relationships of stimuli.

## Regulation of Inflammatory Pain by Neuronal Stimulator of Interferon Genes (STING)

Francina Agosti^a,*^, Siavash Zarezadeh^a^, Nasser Abdullah^a^, Manon Defaye^a^, Mircea Iftinca^a^, Frank Jirik^b^ and Christophe Altier^a^

^a^Department of Physiology & Pharmacology, University of Calgary, Calgary, Alberta, Canada; ^b^Department of Biochemistry & Molecular Biology, University of Calgary, Calgary, Alberta, Canada

**CONTACT** Francina Agosti francina.agosti@ucalgary.ca

© 2020 The Author(s). Published with license by Taylor & Francis Group, LLC.

This is an Open Access article distributed under the terms of the Creative Commons Attribution License (http://creativecommons.org/licenses/by/4.0/), which permits unrestricted use, distribution, and reproduction in any medium, provided the original work is properly cited.

**Introduction/Aim**: Sensory nociceptors that express TRPV1 channel respond to noxious heat/chemical stimuli to produce pain sensation. Recent findings reported that TRPV1+ neurons respond to bacterial infection in the skin, gut or lungs, and modulate host immune response. STING is an innate immune signaling platform that responds to viral/bacterial infections. Using a model of paw infection, we found an upregulation of STING in TRPV1+ neurons.

**Methods**: TRPV1-GFP C57BL/6 mice were used to conduct FACS-sorting of TRPV1+ neurons followed by GeneChip analysis after CFA paw injection. Measure of type I INF was conducted on cultured DRG neurons by ELISA assay, and on DRG by qPCR. Immunostaining of STING was conducted in DRG tissue sections after CFA injections. Measure of STING protein expression was done by WB analysis from DRGs.

**Results**: We found that STING is co-expressed with TRPV1 in DRG neurons, and its expression increases upon CFA infection. We observed that type I INF RNA was increased in DRGs post CFA injection. Moreover, we found that cultured DRG neurons release INFβ in response to STING activation by synthetic agonists (DMXAA and ADUS-100).

**Discussion/Conclusions**: This work uncovers the functional regulation of STING in sensory DRG neurons. Our future experiments will focus on deciphering the role of neuronal STING in TRPV1+ neuronal activity and increased pain sensitivity associated with bacterial infection. We will asses inflammatory response and pain using full and conditional (TRPV1+ neurons) STING KO, as well as TRPV1+ neuron-depleted mice. Our work will highlight a novel role of STING in inflammatory pain induced by bacterial infection.

## Facilitators and Barriers to Use of Parent-targeted Interventions in Vaccination Pain Management of Infants

Shokoufeh Modanloo^a,*^ and Denise Harrison http://orcid.org/0000-0001-7549-7742^b^

^a^School of Nursing, University of Ottawa, Ottawa, Ontario, Canada; ^b^School of Nursing, & Children’s Hospital of Eastern Ontario (CHEO), University of Ottawa, Ottawa, Ontario, Canada

**CONTACT** Shokoufeh Modanloo Smoda044@uottawa.ca

© 2020 The Author(s). Published with license by Taylor & Francis Group, LLC.

This is an Open Access article distributed under the terms of the Creative Commons Attribution License (http://creativecommons.org/licenses/by/4.0/), which permits unrestricted use, distribution, and reproduction in any medium, provided the original work is properly cited.

**Introduction/Aim**: Clinical practice guidelines recommend breastfeeding, secure upright holding and sucrose for infants during vaccination. However, these pain management strategies are inconsistently used during vaccination. This may be partly due to lack of parents’ knowledge or confidence in using these strategies.

**Methods**: As part of an online pilot randomized controlled trial (RCT) in which parent-targeted pain management information was shared with parents of infants prior to their scheduled vaccination, an online survey (15 Likert scale and open-ended questions) was sent to 151 participants to investigate facilitators and barriers to use of pain management strategies subsequent to the vaccination. All data were collected electronically using REDCap. SPSS version 23.0 and thematic analysis was used to perform all descriptive and inferential analyses.

**Results**: 89 participants (59%) completed the survey. Most parents reported having enough information about pain management (80, 90%), and felt confident to use (78, 89%) and comfortable to communicate their choice to clinicians (75, 84%). However, the following barriers were identified: lack of support from clinicians, individual discomfort, infant’s discomfort, and uncomfortable clinical setting, where breastfeeding, upright holding or sucrose was not supported. Facilitators identified were access to a comfortable environment, supportive clinicians, education, sufficient time, available sucrose, and partner support. Solutions included: being confident and advocating for their infant, having a clinical setting which facilitated parents to comfort their infants, empowering parents, and having clinicians’ support.

**Discussion/Conclusions**: This study contributes to implementation science by determining facilitators and barriers of using effective pain management strategies during vaccination of infants.

## Comparative Efficacy of Pharmacologic Interventions for the Prevention of Chronic Postsurgical Pain. A Systematic Review and Network Meta-analysis

Claire Allen^a,*^, Andrew M. Walker^a^, Zahra A. Premji^b^, Marie-Eve Beauchemin-Turcotte^a^, Jenny Wong^a^, Sonya Soh^a^, Geoffrey S. Hawboldt^a^, Kelly Shinkaruk^a^ and David P. Archer^a^

^a^Cumming School of Medicine, University of Calgary, Calgary, Alberta, Canada; ^b^Haskayne School of Business, Libraries and Cultural Resources, University of Calgary, Calgary, Alberta, Canada

**CONTACT** Claire Allen claire.allen@ahs.ca

© 2020 The Author(s). Published with license by Taylor & Francis Group, LLC.

This is an Open Access article distributed under the terms of the Creative Commons Attribution-NonCommercial License (http://creativecommons.org/licenses/by-nc/4.0/), which permits unrestricted non-commercial use, distribution, and reproduction in any medium, provided the original work is properly cited.

**Introduction/Aim**: Optimal perioperative pain management is an important public health goal with 10% to 50% of patients reporting chronic postsurgical pain (CPSP). With few head-to-head trials of CPSP preventive strategies, ranking is difficult. Through a systematic review, pairwise and network meta-analyses (NMA) we aimed to rank interventions for efficacy and adverse effects. Our goals are to inform the selection of preventative measures in perioperative practice and guide future investigations.

**Methods**: We searched Cochrane Central Registry of Controlled Trials, MEDLINE, Embase, ClinicalTrials.gov, and WHO ICTRP for double-blind, randomized controlled trials of CPSP prevention in adults. We assessed risk of bias and confidence level in the evidence. Using meta-regression, we evaluated potential effect modifiers. Primary outcomes were treatment effect and potential harm. We used group-level data and estimated risk ratio with random effects.

**Figure 1.**
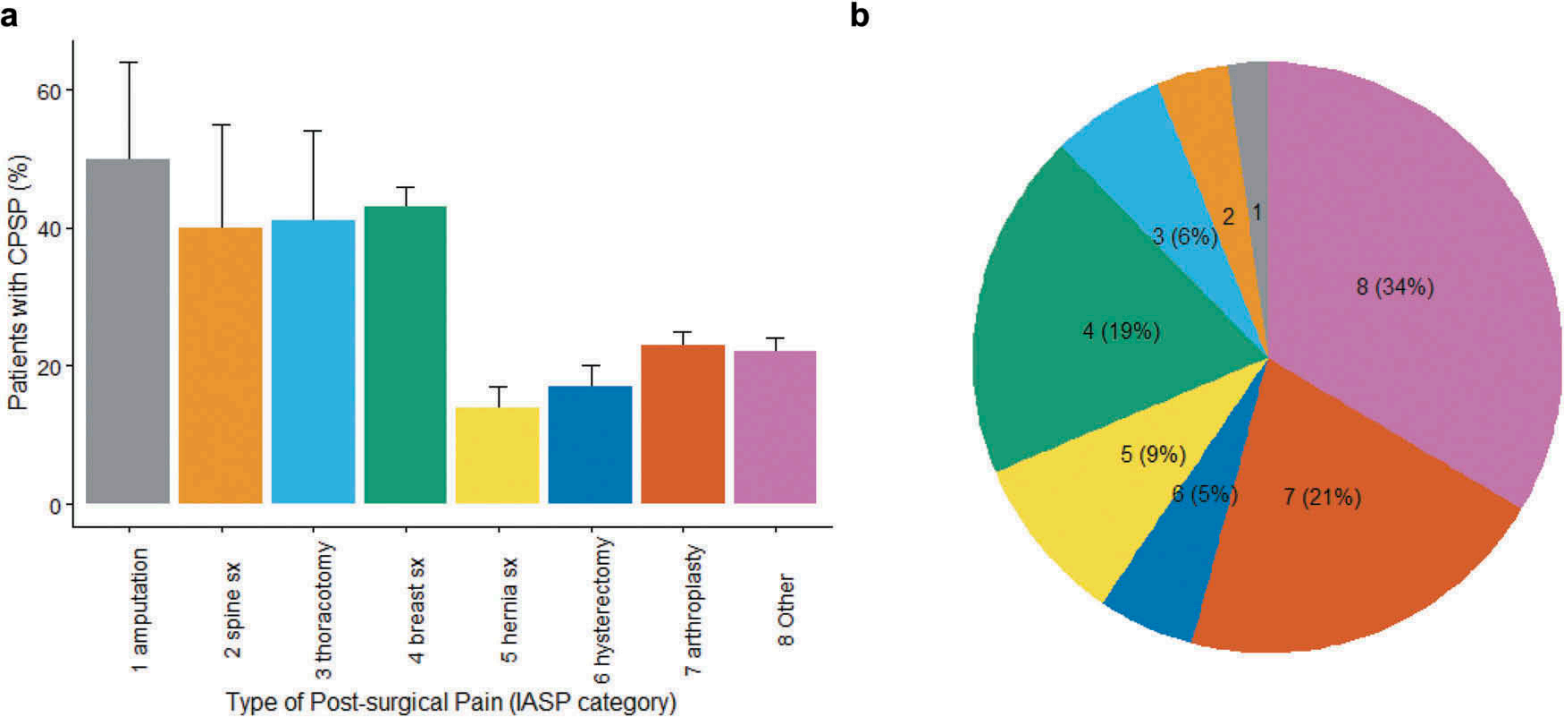
A. Incidence of CPSP according to type of surgery. We grouped categories 1-4 as high risk and 5-8 as low risk. B. The proportion (%) of randomized patients in the included studies according to IASP category. A majority of patients (69%) were in the low risk group.

**Figure 2.**
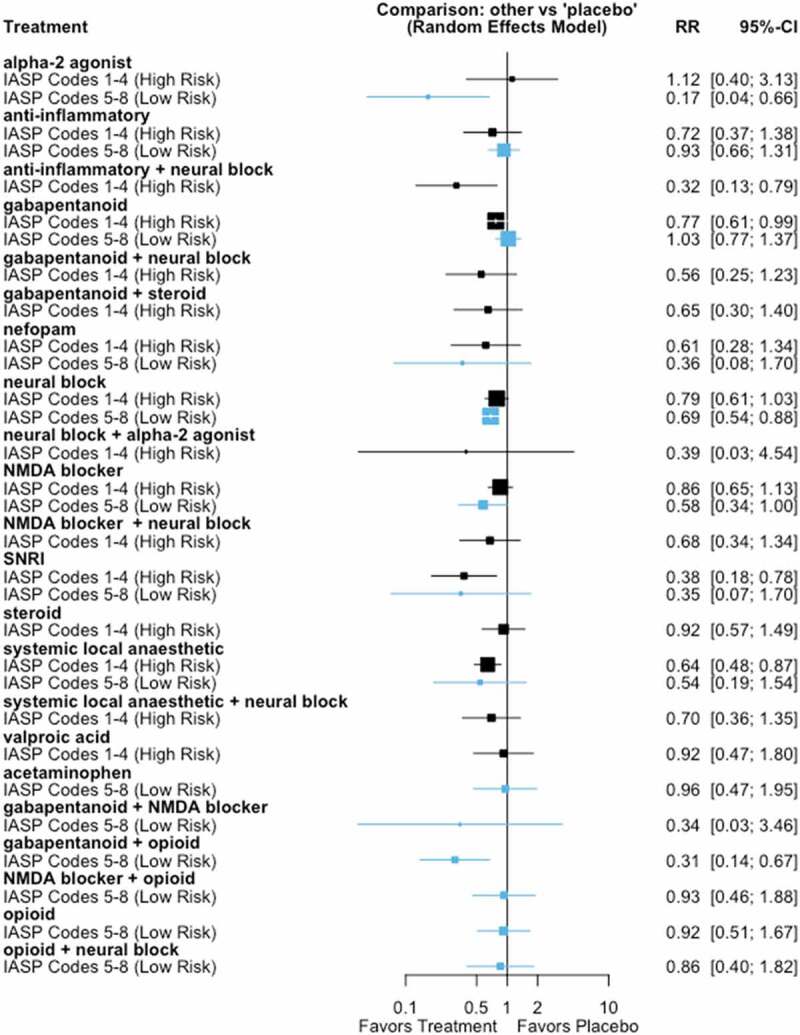
Forest plot showing the risk of CPSP of interventions, relative to placebo in high risk (black) and low risk (blue) surgical categories. The area of each estimate square is proportional to the inverse variance weighting of that comparison in the meta-analysis.

**Figure 3.**
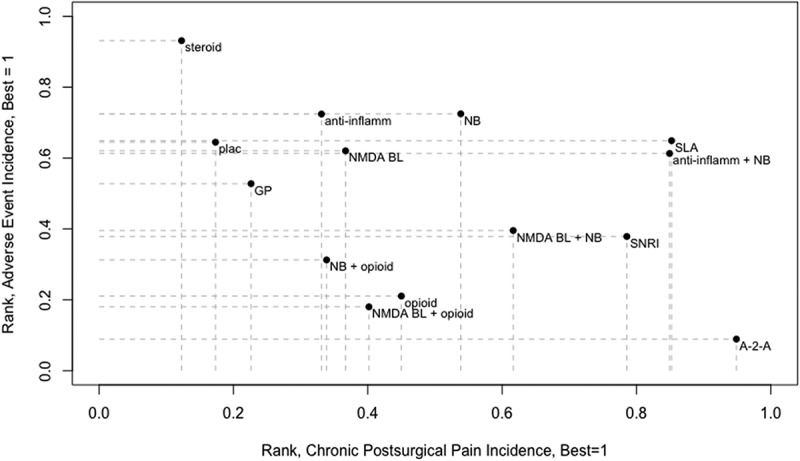
Relative ranking of efficacy and drug-related adverse effects across all categories of CPSP risk. Abbreviations: A-2-A = α-2 receptor agonist; anti-inflamm = anti-inflammatory drug; NB = neural block; NMDA BL = NMDA receptor blocker; SNRI = serotonin noradrenaline reuptake inhibitor, GP = gabapentanoids; plac = placebo.

**Results**: We included 102 studies with 13,416 participants. Here we report preliminary results. CPSP incidence in placebo patients varied according to IASP coding (Fig. 1). In high-risk patients, effective interventions were anti–inflammatories+neural block (NNT = 4), SNRI (NNT = 3), and systemic local anesthetic (NNT = 7). For low-risk patients, effective interventions were alpha-2 receptor agonists (NNT = 6), gabapentanoids + NMDA receptor block (NNT = 5), and neural block (NNT = 15) (Fig. 2). Our ranking for comparative efficacy and adverse effects is in Fig. 3.

**Discussion/Conclusions**: Interventions with “acute post-operative pain benefits” had the greatest effect in reducing CPSP risk. Our ranking suggests that “de-afferentation” with neural block may be important. A preplanned living NMA comparing top-ranking interventions may address under-powered studies with low precision in the estimates.

## Influence of Acute Stress on Interoceptive Accuracy in Preschoolers

Kim Opdensteinen^a,*^, Luca Schaan^a^, Anna Pohl^b^, André Schulz^c^ and Tanja Hechler^a^

^a^Department of Clinical Psychology and Psychotherapy for Children and Adolescents, University of Trier, Trier, Germany; ^b^Department of Psychology, University of Cologne, Cologne, Germany; ^c^Clinical Psychophysiology Laboratory, University of Luxembourg, Esch-sur-Alzette, Luxembourg

**CONTACT** Kim Opdensteinen opdensteinen@uni-trier.de

© 2020 The Author(s). Published with license by Taylor & Francis Group, LLC.

This is an Open Access article distributed under the terms of the Creative Commons Attribution License (http://creativecommons.org/licenses/by/4.0/), which permits unrestricted use, distribution, and reproduction in any medium, provided the original work is properly cited.

**Introduction/Aim**: Altered interoception becomes particularly interesting due to its links to chronic pain (Di Lernia et al., 2016), but little is known on how altered interoception might develop, and how it contributes to chronic pain development. Stress-induced dysregulation of the HPA axis functioning is currently discussed as a potential candidate. However, developmental research into the effects of stress on interoception and their role for chronic pain is lacking.

**Methods**: Therefore, 25 healthy preschool-aged children (15 boys, 4–6 years) completed the modified Jumping Jack Paradigm (JJP; Schaan et al., 2019) to assess interoceptive accuracy (IA) before and after a validated acute laboratory stress task (*n* = 12) or a matched control task (*n* = 13) (Roos et al., 2017). IA, heartrate and cortisol responses were assessed.

**Results**: Comparing the stress and control group, acute stress induction led to a significant increase in heartrate (*F*_1;22_ = 6.0, *p* = .023, *pη2 *= 0.215) and in cortisol response (*F*_2.7;62.5_ = 2.77, *p* = .054). Only in the stress group, cortisol response after the matching task was negatively related to IA (*r*_12_ = −0.62, *p* = .032).

**Discussion/Conclusions**: The present pilot study constitutes a first step to address the salient research question how the reactivity of the physiological stress axes affects IA in healthy preschool children. Reduced IA after stress induction might be due to difficulties in shifting attention between the adjustments of internal sensations (i.e. heart rate) and external cues (i.e. rating scale with different sized circles as a proxy for heart rate). A next step will be the comparison of interoceptive processes between healthy and preschool children with early life pain.

## Expected Beneficial Treatments Identified by Women Prior to Entering an Interdisciplinary Chronic Pelvic Pain Program

Magali Robert^a,*^, Vishal Varshney^b^, Maryam Nasr-Esfahani^c^, John Jarrell^c^ and Caroline Lachance^d^

^a^Department of Obstetrics & Gynecology and Department of Anaesthesiology, Perioperative and Pain Medicine, Cumming School of Medicine, University of Calgary, Calgary, AB, Canada; ^b^Department of Anesthesiology, Perioperative and Pain Medicine, University of Calgary, Calgary, AB, Canada; ^c^Department of Obstetrics and Gynecology, University of Calgary, Calgary, AB, Canada; ^d^Department of Obstetrics and Gynecology, Université de Montreal, Montreal, PQ, Canada

**CONTACT** Magali Robert Magali.robert@AHS.ca

© 2020 The Author(s). Published with license by Taylor & Francis Group, LLC.

This is an Open Access article distributed under the terms of the Creative Commons Attribution License (http://creativecommons.org/licenses/by/4.0/), which permits unrestricted use, distribution, and reproduction in any medium, provided the original work is properly cited.

**Introduction/Aim**: The aim of the study was to characterize the treatments that women entering an interdisciplinary chronic pelvic pain program think will be beneficial for their care.

**Methods**: This is a cohort study of women who were engaged in a one year interdisciplinary chronic pelvic pain program in Calgary in April 2019. Women are required to have seen a general gynecologist prior to referral. All women are asked what treatments they believe will be beneficial as part of their intake questionnaire. Up to three treatments can be reported per patient

**Results**: 98 women were enrolled. The average age was 39.4, range (21–80) with 9.7 years, range (1–49) of pain. The EQ5D health scale was 48.6 range (10 − 90) (n = 87) and PDI, 38 range (0–64) (n = 97). Most women had at least one surgery: 1.8 range (0–9) (n = 93). Parity ranged from 0–4: average 1(n = 89).

Overall 2 women did not answer the question and 32 women (33%) did not know which treatment(s) would be beneficial while 5 women felt they needed investigations to find a cause. A total of 118 treatment choices were listed in the remaining women

A rehabilitation approach was identified in 30 women (31%); 15% believed surgery was required and 13% identified a psychological intervention as beneficial. Medical management (surgery, medication, intervention etc.) was 40% of all treatments required.

**Discussion/Conclusions**: Most women entering an interdisciplinary chronic pelvic pain do not know what treatments they would benefit from. Identified treatments in descending order included: medical/interventional, rehabilitation and psychological interventions.

## Kinematic and Stability Changes in Women Using a Sacroiliac Belt for Pelvic Girdle Pain: A Case-control Study

Magali Robert^a,*^, Sasa Cigoja^b^, Louise-Helene Gagnon^c^

^a^Cumming School of Medicine, Department of Obstetrics & Gynecology and Department of Anaesthesia, Perioperative and Pain Medicine, University of Calgary, Calgary, Alberta, Canada; ^b^Faculty of Kinesiology, University of Calgary, Calgary, Alberta, Canada; ^c^Department of Obstetrics and Gynecology, University of Toronto, Toronto, Ontario, Canada

**CONTACT** Magali Robert Magali.robert@AHS.ca Cumming School of Medicine, Department of Obstetrics & Gynecology and Department of Anaesthesia, Perioperative, University of Calgary, Calgary, AB

© 2020 The Author(s). Published with license by Taylor & Francis Group, LLC.

This is an Open Access article distributed under the terms of the Creative Commons Attribution License (http://creativecommons.org/licenses/by/4.0/), which permits unrestricted use, distribution, and reproduction in any medium, provided the original work is properly cited.

**Introduction/Aim**: To compare the change in stability during standing with application of the sacroiliac belt in women with pelvic girdle pain (PGP) and those without.

To evaluate the kinematic changes during standing and the stance phase of walking with application of the sacroiliac belt in women with PGP and those without.

**Methods**: Five women with PGP were matched to women without pain. Stability was measured with 95% confidence ellipse of center of pressure during standing with the belt off and on. Kinematic studies were performed during standing and the stance phase of walking with and without the belt. Descriptive statistics were used. Wilcoxon signed rank test was used to compare belt conditions and groups.

**Results**:The CoP data showed improved stability in all women with application of the belt (p = .005). No differences between belt conditions were seen during the stance phase of walking. 8 of 10 women showed internal rotation of the femur with standing following application of the belt (p = .040). During the stance phase of walking, all women showed internal rotation (p = .005) and abduction (p = .005) of the femur with belt use.

**Discussion/Conclusions**: Similar stability and kinematic changes were seen in women with and without PGP making it impossible to distinguish between the two groups when looking at these parameters.

## Belief of Cause of Pain in Women Entering an Interdisciplinary Chronic Pelvic Pain Program

Magali Robert^a,*^, Vishal Varshney^b^, Maryam Nasr-Esfahani^c^, John Jarrell^c^ and Caroline Lachance^d^

^a^Department of Obstetrics & Gynecology and Department of Anaesthesia, Perioperative, and Pain Medicine, Cumming School of Medicine, University of Calgary, Calgary, Alberta, Canada; ^b^Department of Anesthesiology, Perioperative and Pain Medicine, University of Calgary, Calgary, Alberta, Canada; ^c^Department of Obstetrics and Gynecology, University of Calgary, Calgary, Alberta, Canada; ^d^Department of Obstetrics and Gynecology, Université De Montreal, Montreal, Quebec, Canada

**CONTACT** Magali Robert Magali.robert@AHS.ca

© 2020 The Author(s). Published with license by Taylor & Francis Group, LLC.

This is an Open Access article distributed under the terms of the Creative Commons Attribution License (http://creativecommons.org/licenses/by/4.0/), which permits unrestricted use, distribution, and reproduction in any medium, provided the original work is properly cited.

**Introduction/Aim**: The aim of the study is to characterize the beliefs of causes of pain in women entering an interdisciplinary chronic pelvic pain program.

**Methods**: This is a cohort study of women who were engaged in a one-year interdisciplinary chronic pelvic pain program in Calgary in April 2019. Women are required to have seen a general gynecologist prior to referral. All women are asked: “What do you believe is the cause of the pain?” as part of their intake questionnaire.

**Results**: 98 women were enrolled. The average age was 39.4, range (21–80) with 9.7 years, range (1–49) of pain. The EQ5D health scale was 48.6 range (10–90) (n = 87) and PDI, 38 range (0–64) (n = 97). Most women had at least one surgery: 1.8 range (0–9) (n = 93). Parity ranged from 0–4: average 1(n = 89).

A total of 137 possible causes were listed in the 98 women. 98 women answered at least one cause, 51 at least two causes, 22 three causes, 3 four causes.

The most common reason women gave for their pain was endometriosis (17% of answers or 23% of women); followed by muscular problems (13% of answers or 18% of women) and neurological (12% of answers and 13% of women). Thirteen women (13% of population) did not know the cause of their pain.

Gynecological reasons were listed as 53/137 of possible etiologies (39%). Over 25 different causes of pelvic pain were identified.

**Discussion/Conclusions**: Women present with diverse beliefs of their cause of pelvic pain. Only 39% are considered of gynecological origin.

## Screening and Diagnostic Tools for Complex Regional Pain Syndrome: A Systematic Review

Giulia Mesaroli^a,*^, Amos Hundert^b^, Kathryn Birnie^c^, Fiona Campbell^d^ and Jennifer Stinson^e^

^a^Department of Rehabilitation, The Hospital for Sick Children and Department of Physical Therapy, Univeristy of Toronto, Toronto, Ontario, Canada; ^b^Child Health Evaluative Sciences, The Hospital for Sick Children, Toronto, Ontario, Canada; ^c^Department of Anesthesiology, Perioperative and Pain Medicine, and the Alberta Children’s Hospital, University of Calgary, Calgary, Alberta, Canada; ^d^Department of Anesthesia and Pain Medicine, The Hospital for Sick Children, University of Toronto, Toronto, Ontario, Canada; ^e^Child Health Evaluative Sciences, The Hospital for Sick Children, Lawrence S. Bloomberg Faculty of Nursing, University of Toronto, Toronto, Ontario, Canada

**CONTACT** Giulia Mesaroli giulia.mesaroli@sickkids.ca

© 2020 The Author(s). Published with license by Taylor & Francis Group, LLC.

This is an Open Access article distributed under the terms of the Creative Commons Attribution License (http://creativecommons.org/licenses/by/4.0/), which permits unrestricted use, distribution, and reproduction in any medium, provided the original work is properly cited.

**Introduction/Aim**: Complex regional pain syndrome (CRPS) is a severely painful condition that presents with a constellation of symptoms. Our understanding of the pathophysiology of CRPS has evolved over time, as has the diagnostic criteria. Our primary objective was to identify screening and diagnostic tools for CRPS and summarize the feasibility, measurement properties and study quality. A secondary objective was to identify screening and diagnostic tools used for CRPS in pediatric populations (0– 21 years of age).

**Methods**: A systematic review of English articles in electronic databases (PsycINFO, MEDLINE, Embase, CINAHL, CENTRAL, and Web of Science was conducted with the aid of a librarian on November 21, 2018. Studies were included if the tool was a screening or diagnostic tool, the tool included self-report or physical examination, and the primary objective of the study was to evaluate the measurement properties or feasibility. For each study, data was extracted for quality indicators using a QUADAS-2 tool.

**Results**: No screening tools were identified. Four diagnostic tools were identified and met Cohen’s criteria for well establishment (Veldman’s Criteria, IASP Criteria, Budapest Criteria and the Budapest Research Criteria). There are no well-established diagnostic tools for CRPS in youth.

**Discussion/Conclusions**: As there are no extant screening tools for CRPS, all people with suspected disease should undergo rapid diagnostic assessment by a clinician. For adults, the Budapest Criteria are the preferred diagnostic tool. Future research is recommended to develop a diagnostic tool for pediatric populations and screening tools for both pediatric and adults.

## Anti-allodynic Effects of Delta-9-Tetrahydrocannabinol (THC) and Cannabidiol (CBD) in Neuropathic Rats Tolerant to Morphine

Leora Pearl-Dowler^a,*^, Luca Posa^b^, Alexandra Teggin^c^ and Gabriella Gobbi^b^

^a^Department of Psychiatry and Integrated Program in Neuroscience, McGill University, Montreal, Quebec, Canada; ^b^Department of Psychiatry and Alan Edwards Centre for Research on Pain, McGill University, Montreal, Quebec, Canada; ^c^Department of Psychiatry, McGill University, Montreal, Quebec, Canada

**CONTACT** Leora Pearl-Dowler leora.pearl-dowler@mail.mcgill.ca

© 2020 The Author(s). Published with license by Taylor & Francis Group, LLC.

This is an Open Access article distributed under the terms of the Creative Commons Attribution License (http://creativecommons.org/licenses/by/4.0/), which permits unrestricted use, distribution, and reproduction in any medium, provided the original work is properly cited.

**Introduction/Aim**: Neuropathic pain (NP) is a chronic health disorder caused by damage to the nervous system. NP is characterized by persistent pain that is often treated with opioids, which produce tolerance and dependence. There is thus a need to find alternative drugs for patients who have NP and are tolerant to morphine. We explored the hypothesis that the cannabinoids Delta-9-Tetrahydrocannabinol (THC) and Cannabidiol (CBD) might be used as analgesics following opioid tolerance.

**Methods**: Wistar rats underwent spared nerve injury and were treated with morphine (5 mg/kg s.c. twice daily) or vehicle for 7 days. They were assessed for tolerance to morphine using von-Frey filaments and tolerant rats were then treated with THC (2.5 mg/kg i.p.) or CBD (20 mg/kg i.p.). In-vivo electrophysiological recording of ON cells in the rostral ventral medulla were recorded after injection of THC (10ug) or CBD (1ug) into the periaqueductal gray in tolerant rats to determine the responses of these cells.

**Results**: A significant progressive reduction in the anti-allodynic effect of morphine was observed over the 7 days of treatment. THC maintained its anti-allodynic effect in morphine-tolerant (AUC = 19.48 ± 0.71) compared with non-tolerant rats (AUC = 20.82 ± 0.34, p=n.s.). Furthermore, THC produced a modest reduction in the firing rate of ON cells in morphine-tolerant (33.7%), compared to non-tolerant rats (45.7%). Instead, preliminary results showed that the antiallodynic effects of CBD were attenuated in morphine-tolerant animals.

**Discussion/Conclusions**: Overall, these data suggest that THC, but not CBD, may be an effective analgesic to manage neuropathic pain in morphine-tolerant subjects.

## Pain, Neuropathy, and Impaired Mobility are Associated with Reduced Taxane Dose in Women Undergoing Breast Cancer Chemotherapy

Lye-Ann Robichaud^a,*^, Marianne Bouvrette^b^, Maud Bouffard^c^, Lucia Gagliese^d^, Robert H. Dworkin^e^, Jennifer Gewandter^e^, Julie Lemieux^f^, Josée Savard^a^, Michèle Aubin^b^, Philip Jackson^a^, Anne Dionne^g^, Bruno Gagnon^b^, Pierre Gagnon^h^, Sophie Lauzier http://orcid.org/0000-0002-7746-9208^g^, Cindy Shobbrook^i^, David Warr^j^ and Lynn R. Gauthier^b,c^

^a^School of Psychology, Université Laval, Quebec City, Quebec, Canada; ^b^Faculty of Medicine, Université Laval, Quebec City, Quebec, Canada; ^c^Oncology Division, Research Centre of the CHU de Quebec, Quebec City, Quebec, Canada; ^d^School of Kinesiology and Health Science, York University, Toronto, Ontario, Canada; ^e^School of Medicine and Dentistry, University of Rochester Medical Center, Rochester, New York, USA; ^f^Centre des maladies du sein Deschênes-Fabia, Hôpital du Saint-Sacrement, CHU de Quebec, Quebec City, Quebec, Canada; ^g^Faculty of Pharmacy, Université Laval, Quebec City, Quebec, Canada; ^h^Department of Psychiatry and Neurosciences, Université Laval, Quebec City, Quebec, Canada; ^i^Faculty of Nursing, University of Toronto, Toronto, Ontatio, Canada; ^j^Department of Medical Oncology, Princess Margaret Cancer Centre, Toronto, Ontario, Canada

**CONTACT** Lynn R Gauthier lynn.gauthier@crchudequebec.ulaval.ca

© 2020 The Author(s). Published with license by Taylor & Francis Group, LLC.

This is an Open Access article distributed under the terms of the Creative Commons Attribution License (http://creativecommons.org/licenses/by/4.0/), which permits unrestricted use, distribution, and reproduction in any medium, provided the original work is properly cited.

**Introduction/Aim**: Chemotherapy-induced peripheral neuropathy (CIPN) is associated with dose reductions or early discontinuation (DR/ED) in 2%–40% of women undergoing taxane-based treatment for breast cancer (BC), potentially affecting survival. Understanding factors associated with CIPN-related-DR/ED could improve treatment decision-making. We compared CIPN, pain, quality of life (QOL) and psychosocial wellbeing; balance/mobility; and sensation (ST) and pain threshold (PT) between women who received taxane-as-prescribed (TAP) versus DR/ED.

**Methods**: 121 women with BC completed CIPN (FACT-Taxane), pain intensity and qualities (Short-Form McGill Pain Questionnaire-2[SF-MPQ-2]), QOL, fatigue, and psychosocial wellbeing measures, the Timed-Up-and-Go (TUG), and foot and hand thermal, vibration, and touch ST/PT prior to chemotherapy and after the last taxane cycle. Group 1 (G1;n = 74[60.7%]) received TAP. Group 2 (G2;n = 36[29.5%]) had neuropathy-related-DR/ED. Group 3 (G3;n = 11[9.0%]) had DR/ED for other reasons. DR/ED reasons were abstracted from medical charts. Chi-square and mixed ANOVA characterized the sample and change over time.

**Results**: Groups did not differ on age, neoadjuvant vs. adjuvant treatment, or comorbidities (*p* > .05). More G2 received paclitaxel than docetaxel (80%vs.20%; *p* = .001) compared to G1 (50.1% vs.49.3%,NS) and G3 (18.1%vs.81.8%,NS). There were group X time interactions on FACT-Taxane and SF-MPQ-2-Neuropathic (*p* ≤ 0.001; G2 > G1, *p* < .001), and group main effects for foot and hand cold PT (*p* < .05; G2 < G1) and TUG (*p* = .05, G2 > G1). There were no interactions or group main effects on QOL, fatigue, and psychosocial wellbeing.

**Discussion/Conclusions**: Compared to TAP, neuropathy-related-DR/ED was associated with a greater increase in CIPN and neuropathic pain, lower cold PT, and longer TUG, suggesting balance/mobility impairment. Further research characterizing CIPN-related-DR/ED could improve decision-making and QOL.

## Knowledge and Beliefs of the Medical Use of Cannabis among Surgical Nurses: Understanding Current Needs and Exploring Knowledge Enhancement Strategies

Salima S. J. Ladak^a,*^, Jiao Jiang^b^, Arlene Buzon-Tan^a^, Susan Walker^a^, Kari Van Camp^c^, Simone Charles^c^, Diana Tamir^a^ and Hance Clarke http://orcid.org/0000-0003-4975-3823^a^

^a^Anesthesia and Pain Management, University Health Network, Toronto, Ontario, Canada; ^b^Anesthesia Department, Sunnybrook Health Science Centre, Toronto, Ontario, Canada; ^c^Lawrence S. Bloomberg Faculty of Nursing, University of Toronto, Toronto, Ontario, Canada

**CONTACT** Salima S. J. Ladak salima.ladak@uhn.ca

© 2020 The Author(s). Published with license by Taylor & Francis Group, LLC.

This is an Open Access article distributed under the terms of the Creative Commons Attribution License (http://creativecommons.org/licenses/by/4.0/), which permits unrestricted use, distribution, and reproduction in any medium, provided the original work is properly cited.

This prospective observational study was primarily designed to understand nurses’ knowledge of the medical use of cannabis in the acute post-operative setting, as well as their beliefs regarding cannabis for medical use. The secondary aim was to understand what influenced these knowledge levels and beliefs. 230 registered nurses aged 20–70 years working in a quaternary care academic health science center participated. Desired knowledge levels across all topics pertaining to medical cannabis use, such as therapeutic indications, potential risks, mechanism of action, routes of administration, and dosing strategies were higher than current knowledge (paired t-test, *p* < .001). Nurses’ current levels of knowledge were rated lowest in the areas pertaining to Health Canada’s Marijuana for Medical Access Regulations as well as dosing and creating effective treatment plans for patients using medical cannabis. On average, nurses provided neutral responses to questions pertaining to whether (1) they would be comfortable discussing cannabis for medical use (2) protection from legal liability would improve this level of comfort, (3) they believed patients could die from cannabis overuse, 4) cannabis is a strong analgesic that can be used to treat acute pain, and (5) additional education would improve their confidence to have discussions with or treat patients already using medical cannabis. Clear gaps were identified by RNs describing current versus desired knowledge levels related to the use of medical cannabis, as well as beliefs about the effects of cannabis. Ward-based educational updates, workshops and small group learning opportunities were the most favored educational interventions.

**Introduction/Aim**: This study was primarily designed to understand nurses’ knowledge levels of the medical use of cannabis in the acute post-operative setting, as well as their personal beliefs regarding cannabis for medical use. The secondary aim was to understand factors that influenced these knowledge levels and beliefs. This poster will illustrate knowledge gaps that were identified and provide recommendations for future educational interventions.

**Methods**: A prospective observational study of surgical nurses (N = 230; aged 20–70 years) in a multi-center quaternary care academic health science center was conducted. The main outcome measures were composite knowledge and belief scores, and predictors that influenced these measures.

**Results**: Desired knowledge across all topics pertaining to medical cannabis use, such as therapeutic indications, potential risks, mechanism of action, routes of administration, and dosing strategies were found to be significantly higher than current knowledge; all paired t-test *p*-values < 0.001. Nurses’ current levels of knowledge were rated lowest in the areas pertaining to Health Canada’s Marijuana for Medical Access Regulations as well as dosing and creating effective treatment plans for patients using medical cannabis. On average, nurses provided neutral responses to five attitude questions pertaining to whether (1) they would be comfortable discussing cannabis for medical use with their patients, (2) protection from legal liability would improve this level of comfort, (3) they believed patients could die from cannabis overuse, 4) cannabis is a strong analgesic that can be used to treat acute pain, and (5) additional education would improve their confidence to have discussions with patients or treat patients already using medical cannabis

**Discussion/Conclusions**: Clear gaps were identified by RNs describing their current versus desired knowledge levels related to the use of medical cannabis, as well as beliefs about the effects of cannabis. Future educational interventions should address these gaps to enable RNs to improve their professional capacity to better address patient and family questions. Ward-based educational updates, workshops and small group learning opportunities were the most favored educational interventions.

## Co-Existing Primary Pain Sites in Children and Adolescents are Associated with Depressive and Anxiety Symptoms and Reduced Quality of Life

Paulina Podgorny^a,*^, Alfred K. Yeung^b^, Madison Kennedy^c^, Cara Nania^c^ and Melanie Noel http://orcid.org/0000-0003-3752-8055^d^

^a^Department of Pediatrics, University of Calgary, Calgary, AB, Canada; ^b^Department of Pediatrics, Section of Gastroenterology, Hepatology, and Nutrition, University of Calgary, Calgary, AB, Canada; ^c^Department of Psychology, University of Calgary, Calgary, AB, Canada; ^d^Department of Psychology, Alberta Children’s Hospital Research Institute (Behaviour and the Developing Brain Theme), Hotchkiss Brain Institute, Mathison Centre for Mental Health Research and Education, University of Calgary, Calgary, AB, Canada

**CONTACT** Paulina Podgorny pcpodgor@ucalgary.ca

© 2020 The Author(s). Published with license by Taylor & Francis Group, LLC.

This is an Open Access article distributed under the terms of the Creative Commons Attribution License (http://creativecommons.org/licenses/by/4.0/), which permits unrestricted use, distribution, and reproduction in any medium, provided the original work is properly cited.

**Introduction/Aim**: Pediatric chronic pain is often categorized into three primary sites: abdominal, headache, and musculoskeletal. Studies have demonstrated increased lifetime risk for depressive and anxiety disorders in children and adolescents with chronic pain. These studies have focused primarily on singular pain sites. The Vi Riddell Pain Program was developed to assess chronic pain of various types in youth for up to 3 years. Our goal was to examine the impact of co-existing multiple primary pain sites on the quality of life and mental health of children enrolled in this program.

**Methods**: A retrospective analysis of 38 8- to 17-year-olds (73.7% female) attending an outpatient multidisciplinary chronic pain program was performed. Patients completed self-report scales for anxiety, depression (PROMIS), and quality of life (PedsQL). Bi-variate correlations were done between average pain scores and mental health, and QoL subscales.

**Results**: Children with abdominal pain had chronic pain in other sites, most commonly headache (50%). In our cohort, 44.7% of children reported pain that was “always present” and 42% indicated an intensity ≥8/10 in the last week. This group’s average pain rating correlated with higher rates of depressive symptoms (r = 0.340, *p* < .05), anxiety (r = 0.358, *p* < .05), and decreased QoL (r = −0.445, *p* < .01).

**Discussion/Conclusions**: Our findings suggest the symptoms experienced by children with abdominal pain and another chronic pain site are associated with a current reduction in quality of life and increased rates of internalizing mental health disorders. Longitudinal studies to investigate the association between specific pain sites and pain amplification in childhood may be warranted.

## Role of Excitatory Serotonin Receptor, 5HT_2A_, and Estrogen Fluctuations in Trigeminal Pain Signaling

Sukhbir Kaur^a,*^, Hanna McDonald^b^, Taylor Hickman^a^, Dayna L. Averitt^a^

^a^Department of Biology, Texas Woman’s University, Denton, Texas, USA; ^b^Department of Medical Science, University of North Texas Health Science Center, Fort Worth, Texas, USA

**CONTACT** Sukhbir Kaur slulla@twu.edu

© 2020 The Author(s). Published with license by Taylor & Francis Group, LLC.

This is an Open Access article distributed under the terms of the Creative Commons Attribution License (http://creativecommons.org/licenses/by/4.0/), which permits unrestricted use, distribution, and reproduction in any medium, provided the original work is properly cited.

**Introduction/Aim**: Craniofacial disorders involving trigeminal sensory neurons disproportionately affect women. A subset of these neurons expresses transient receptor potential vanilloid 1 ion channels (TRPV1). TRPV1 is activated by capsaicin, and can be sensitized by proinflammatory mediators, including serotonin(5HT), causing an enhanced Ca^+2^influx and calcitonin-gene related-peptide (CGRP) release. Studies in males report that trigeminal neurons co-express 5HT_2A_ receptors and TRPV1, providing an anatomical substrate for sensitization. Interestingly, we found that peripheral 5HT evokes greater orofacial pain behaviors in proestrus and estrus females. Here, we hypothesized that estrogen fluctuations exacerbate serotonergic sensitization of capsaicin-evoked pain via 5HT_2A_ receptor.

**Methods**: Adult male, cycling and ovariectomized female Sprague-Dawley rats (n = 160) received one vibrissal pad injection of 1.5 µg or 3 µg 5HT+1 µg/50 μl capsaicin, or capsaicin only. Number of vibrissal pad forelimb swipes were counted as nocifensive behavior. Another group received M100907 (5HT_2A_ antagonist; 2 nM/100 μl) 15 min before 5HT-evoked nocifensive behavior testing. Also, trigeminal ganglia cultures were treated with M100907 followed by 5HT+capsaicin stimulation and CGRP release was quantified by ELISA. Data were analyzed by ANOVA and Tukey’s posthoc analysis. Currently, we are performing in-situ hybridization using oligo-probes against 5HT_2A_ and TRPV1 mRNA.

**Results**: Capsaicin-evoked nocifensive behaviors were significantly exacerbated by 3 µg 5HT in estrus females and males, whereas by 1.5 µg 5HT in proestrus females. Our preliminary data indicates that blocking peripheral 5HT_2A_ receptors reduces 5HT effects on nocifensive behaviors in both males and females, and also reduces *in vitro* capsaicin-evoked CGRP release.

**Discussion/Conclusions**: Estrogen modulates pronociceptive effects of 5HT on trigeminal sensory neurons via excitatory 5HT_2A_ receptor.

## Pain Acceptance Predicts Reduced Pain Catastrophizing and Pain-related Disability One Year after Major Surgery

Muhammad Abid Azam http://orcid.org/0000-0002-6016-1274^a,*^, Aliza Weinrib^a^, Ze’ev Seltzer^b^, Marcelo Cypel^c^, Vivek Rao^d^, Elham ElSherif^a^, Dorothy Wong^a^, Hance Clarke^a^ and Joel Katz^a^

^a^Department of Anesthesia and Pain Management, Pain Research Unit, Toronto General Hospital, University Health Network, Toronto, Ontario, Canada; ^b^Center for the Study of Pain, University of Toronto, Ontario; ^c^Department of Surgery, University Health Network, Toronto, Ontario; ^d^Peter Munk Cardiac Center, Toronto General Hospital, Toronto, Ontario

**CONTACT** Muhammad Abid Azam abidazam@yorku.ca

© 2020 The Author(s). Published with license by Taylor & Francis Group, LLC.

This is an Open Access article distributed under the terms of the Creative Commons Attribution License (http://creativecommons.org/licenses/by/4.0/), which permits unrestricted use, distribution, and reproduction in any medium, provided the original work is properly cited.

**Introduction/Aim**: Catastrophizing and disability are primary targets of cognitive-behavioral treatment for chronic pain. According to acceptance and commitment therapy, increased pain acceptance protects against the deleterious effects of pain catastrophizing and pain-related disability. The aim of this study was to further support this rationale by examining whether pain acceptance predicts lower pain catastrophizing and disability 12 months after cardiothoracic surgery in patients who develop persistent post-surgical pain (PPSP).

**Methods**: PPSP was reported by n **= **111 (8.2%) of patients 12 months after surgery (mean age = 56.1 years; 48.6% female; mean NRS pain = 3.36, SD = 2.57). Pain acceptance at 3 months was measured using the Chronic Pain Acceptance Questionnaire (CPAQ). Pain disability and catastrophizing at 12-months were measured using the Pain Disability Index (PDI) and Pain Catastrophizing Scale (PCS), respectively. Correlational and multiple regression analyses were conducted to examine relationships between CPAQ, PCS, and PDI scores.

**Results**: Pain acceptance was a significant inverse predictor of pain disability (*r*(60) = −0.59, *r^2^ *= 0.35, *p* < .001) and pain catastrophizing (*r*(65) = −0.54, *r^2^ *= 0.29, *p* < .001). In separate multiple regression models, 3-month pain acceptance was the only significant predictor of pain disability [*F*(4, 48) = 8.68, *p* < .001, R^2^ = 0.42; (*B *= −0.38, *p* < .001)] and pain catastrophizing [*F*(4, 51) = 5.41, *p* = .001, R^2^ = 0.30; (*B *= −0.53, *p* < .001)] at 12-months after controlling for sex, pre-surgery chronic pain, and mean pain intensity at 12-months.

**Discussion/Conclusions**: For patients who develop PPSP, treatments emphasizing pain acceptance may reduce catastrophizing and disability in the long-term by fostering greater re-engagement with valued life activities.

## Improvements in Youth Functional Outcomes following Physical Therapy within Intensive Pain Rehabilitation

Allison McPeak^a,*^, Amanda de Chastelain^b^, Nivez Rasic^c^ and Melanie Noel http://orcid.org/0000-0003-3752-8055^d^

^a^Cumming School of Medicine, University of Calgary, Calgary, Alberta, Canada; ^b^Physical Therapist, Alberta Children’s Hospital, The Vi Riddell Children’s Pain & Rehabilitation Centre, Calgary, Alberta, Canada; ^c^Department of Anesthesia, University of Calgary, Calgary, Alberta, Canada; ^d^University of Calgary, Department of Psychology, Alberta Children’s Hospital Research Institute (Behaviour and the Developing Brain Theme), Hotchkiss Brain Institute, Mathison Centre for Mental Health Research and Education, Calgary, Alberta, Canada

**CONTACT** Allison McPeak aemcpeak@ucalgary.ca

© 2020 The Author(s). Published with license by Taylor & Francis Group, LLC.

This is an Open Access article distributed under the terms of the Creative Commons Attribution License (http://creativecommons.org/licenses/by/4.0/), which permits unrestricted use, distribution, and reproduction in any medium, provided the original work is properly cited.

**Introduction/Aim**: Chronic pain in youth is a growing epidemic that adversely affects psychosocial and functional domains. The Intensive Pain Rehabilitation Program (IPRP) at the Alberta Children’s Hospital attempts to alter these adverse effects via multidisciplinary rehabilitation through a 3-to-6-week, day-treatment model. This program focuses on improving pain self-management skills utilizing interventions from psychologists, family and art therapists, nurses, anesthesiologists, and physical and occupational therapists. Research into the role of physical activity in chronic pain rehabilitation has been limited and largely conducted in adult populations. However, favorable results have been found related to pain severity and physical functioning, which is closely linked to pain-related fear. The present study examined change in self-reported pain interference, fear of pain, and functional disability along with preliminary physical therapy data following youth participation in IPRP.

**Methods**: Thirty-eight adolescents completed measures of pain interference, fear of pain, and functional disability at baseline (pre-IPRP) and discharge (post-IPRP). Preliminary physical therapy data (n = 11) examined included the Six Minute Walk Test (6MWT), an objective assessment of functional exercise capacity. Paired sample T-tests were used to examine change between baseline and discharge of the IPRP program.

**Results**: Between baseline and discharge youth reported less pain interference (*p* = .002), fear of pain (*p* = .001), and disability (*p* = .003). The preliminary physical therapy data illustrated an improvement in the 6MWT (*p* = .046) following physical therapy within IPRP.

**Discussion/Conclusions**: Findings show that youth attending IPRP report less pain interference, fear of pain, and disability. In addition, youth are experiencing objective improvements in their functional exercise capacity.

## Is It Worth It? Is an Intensive Interdisciplinary Pain Treatment Program Making a Difference in Youth with Pain-related Disability?

Karen Hurtubise http://orcid.org/0000-0003-3441-7106^a,*^, Astrid Brousselle^b^, Melanie Noel http://orcid.org/0000-0003-3752-8055^c^, Nivez Rasic^d^ and Chantal Camden http://orcid.org/0000-0002-5503-3403^a,e^

^a^Faculté de Médecine et Sciences de la santé, Université de Sherbrooke, Sherbrooke, Quebec, Canada; ^b^School of Public Administration, University of Victoria, Victoria, British Columbia, Canada; ^c^Department of Psychology, University of Calgary, Calgary, Alberta, Canada; ^d^Department of Anesthesia & Pain Medicine, Foothills Hospital, Calgary, Alberta, Canada; ^e^Centre de Recherche du Centre Hospitalier Universitaire de Sherbrooke, Sherbrooke, Quebec, Canada

**CONTACT** Karen Hurtubise karen.hurtubise@usherbrooke.ca

© 2020 The Author(s). Published with license by Taylor & Francis Group, LLC.

This is an Open Access article distributed under the terms of the Creative Commons Attribution License (http://creativecommons.org/licenses/by/4.0/), which permits unrestricted use, distribution, and reproduction in any medium, provided the original work is properly cited.

**Introduction/Aim**: Multimodal treatment (MMT) and day-hospital intensive interdisciplinary pain treatment (IIPT) are common interventions prescribed to youth with pain-related disability. Rarely have these two models been compared. Our study aimed to evaluate and compare the outcomes and perceived impacts of IIPT and MMT provided at the same facility.

**Methods**: A mixed method convergent design was used. Forty-four IIPT and 138 MMT youth completed the PROMIS Pain Interference for Youth and Children and the PedsQL 4.0 Generic Core Scale at admission, 3 and 12-months post-program. Sixteen IIPT and eight MMT youth and parents then agreed to be interviewed. Questionnaire data was analyzed using longitudinal mixed modeling, while themes were generated from interview transcripts and compared.

**Results**: The IIPT group demonstrated significant improvement at 3-months and 12-months on the PROMIS Pain Interference. Significant improvements in the PedsQL were noted in both groups across timepoints, with a trend toward more improvement in the MMT group. Themes demonstrated that IIPT youth and parents were confident and empowerment in managing the pain, as compared to those in the MMT. However, IIPT youth described more academic and social repercussions upon their return from the program, than those enrolled in MMT, where academic and social supports were perceived to be intact.

**Discussion/Conclusions**: Comparing IIPT and MMT specialized pain treatments underscores the positive outcomes experienced by youth in both groups, and highlighted youth most likely to benefit from each program. Discussing these program differences is crucial to facilitating informed family treatment choices.

## Medication Literacy for Youth with Chronic Pain

Sehjal Bhargava^a,*^, Casey McMahon^b^, Loren Regier^c^ and Krista Baerg^b^

^a^College of Medicine, University of Saskatchewan, Saskatoon, Saskatchewan, Canada; ^b^Department of Pediatrics, University of Saskatchewan, Saskatoon, Saskatchewan, Canada; ^c^RxFiles, University of Saskatchewan, Saskatoon, Saskatchewan, Canada

**CONTACT** Sehjal Bhargava srb158@mail.usask.ca

© 2020 The Author(s). Published with license by Taylor & Francis Group, LLC.

This is an Open Access article distributed under the terms of the Creative Commons Attribution-NonCommercial License (http://creativecommons.org/licenses/by-nc/4.0/), which permits unrestricted non-commercial use, distribution, and reproduction in any medium, provided the original work is properly cited.

**Introduction/Aim**: Chronic pain affects 1 in 5 youth, and often requires complex multimodal management strategies. Resources on pharmacological management of chronic pain are lacking for youth. Improved health literacy, developed through patient education has been linked to positive patient outcomes. To increase pediatric chronic pain medication literacy, we propose the development of a pain medication resource for youth living with chronic pain, informed by youth with chronic pain.

**Methods**: The survey was distributed via Canadian community pediatricians, pain clinic distribution lists and social media. Youth, their caregivers, and healthcare providers were surveyed about information youth need to inform their decisions about chronic pain medication. Youth were surveyed about where they currently acquire information. Data was coded and analyzed.

**Results**: Sixty-eight participants completed the survey, 36 healthcare providers, 22 youth, and 10 caregivers. Information on drug side effects, medication alternatives, dosing, effects of long-term drug use and the role of medication in managing chronic pain were top areas of interest among participants. Primary sources of health information for youth are their doctor, the internet, and pharmacists. Youth indicated they would utilize an interactive resource such as a website or app to access information about chronic pain, medications, and to connect with other youth living with chronic pain.

**Discussion/Conclusions**: Medication side effects are of top concern when choosing a medication. A resource for youth containing reliable health and medication information would help empower youth to advocate for themselves and make educated health decisions, resulting in improved disease management and health outcomes.

## Psychosocial and Mechanism-based Pain Assessment in Pediatric Patients with Chronic Back Pain: A Cluster Analysis

Don Daniel Ocay^a,*^, Allison Loewen^b^, Shajenth Premachandran^a^, Pablo M. Ingelmo^c^, Neil Saran^d^, Jean A. Ouellet^d^ and Catherine E. Ferland^c^

^a^Experimental Surgery, McGill University, Montreal, Quebec, Canada; ^b^Medicine, McGill University, Montreal, Quebec, Canada; ^c^Anesthesia, McGill University, Montreal, Quebec, Canada; ^d^Pediatric Orthopedics, McGill University, Montreal, Quebec, Canada

**CONTACT** Don Daniel Ocay don.ocay@mail.mcgill.ca

© 2020 The Author(s). Published with license by Taylor & Francis Group, LLC.

This is an Open Access article distributed under the terms of the Creative Commons Attribution License (http://creativecommons.org/licenses/by/4.0/), which permits unrestricted use, distribution, and reproduction in any medium, provided the original work is properly cited.

**Introduction/Aim**: Psychophysical characteristics of patients with chronic back pain have been shown to be heterogeneous. Identifying subgroups with different clinical profiles may inform tailored management and improve outcomes. The objective of this study was to identify clinical profiles of children and adolescents that have similar psychophysical characteristics of chronic back pain.

**Methods**: One hundred and ninety-eight patients with chronic back pain were recruited for the study. Pain assessment was mainly conducted in the form of an interview and with the use of validated pain-related questionnaires assessing their psychosocial factors and quality of life. All patients underwent mechanical and thermal quantitative sensory tests assessing detection and pain thresholds, and conditioned pain modulation efficacy.

**Results**: Hierarchal clustering partitioned our patients into three clusters. Cluster 1 represented 45.5% of the patients and was characterized by a higher pain threshold and lower psychosocial factors. Cluster 2, representing 35.4% of the cohort was considered to have low psychosocial factors and low pain thresholds. And finally, Cluster 3, 19.2% of patients had increased psychosocial factors and low pain thresholds.

**Discussion/Conclusions**: This study identified clinical profiles of children and adolescents experiencing chronic back pain based on specific physical and psychosocial characteristics highlighting that chronic pain treatment should address underlying nociceptive and non-nociceptive mechanisms.

## Optogenetic Manipulation of Synaptic Plasticity in Single Spinal Dorsal Horn Neurons

Erika K. Harding^a,*^, Samuel W. Fung^a,*^ and Robert P. Bonin^a^

^a^Department of Pharmaceutical Sciences, University of Toronto, Toronto, Ontario, Canada

**CONTACT** Erika K. Harding e.harding@mail.utoronto.ca

^*^Co-first authors

© 2020 The Author(s). Published with license by Taylor & Francis Group, LLC.

This is an Open Access article distributed under the terms of the Creative Commons Attribution-NonCommercial License (http://creativecommons.org/licenses/by-nc/4.0/), which permits unrestricted non-commercial use, distribution, and reproduction in any medium, provided the original work is properly cited.

**Introduction/Aim**: Pathological pain is one of the most common causes of debilitation worldwide and is induced by perturbations within the nociceptive signaling pathway. The spinal cord superficial dorsal horn (SDH) is a crucial component along this pathway and exhibits increased excitability during pathological pain. One process thought to govern this increased excitability is synaptic plasticity, in the form of long-term potentiation (LTP) of SDH neurons. However, the SDH is comprised of a poorly characterized, heterogeneous neuronal population. Therefore, we combined patch-clamp electrophysiology with optogenetics in single SDH neurons to determine which neuronal populations are capable of undergoing LTP.

**Methods**: We obtained acute L4-L6 parasagittal slices from adult TRPV1-ChR2 transgenic mice and recorded from individual SDH neurons using patch-clamp electrophysiology. We then excited SDH neurons using selective optogenetic stimulation of TRPV1+central terminals via patterned light stimulation with a 488 nm LED, and measured resultant optogenetic excitatory postsynaptic potentials (oEPSPs).

**Results**: Selective optogenetic stimulation of TRPV1+central terminals produced oEPSPs in 12/12 (100%) SDH neurons. For each neuron, LED intensity was optimized to produce oEPSPs resembling spontaneous EPSPs. An optical stimulation paradigm (2 Hz for 2 minutes) was then used to induce SDH neuron synaptic plasticity and produced LTP in 4/8 (50%) neurons. We then grouped SDH neurons by firing type and morphology to determine potential cell-type differences in their capacity to undergo LTP.

**Discussion/Conclusions**: Our findings demonstrate the capability of individual SDH neurons to undergo long-term potentiation, concomitantly defining the neuronal populations contributing to increased excitability observed in pathological pain conditions.

## Working Memory Predicts Academic Performance above and beyond Pain Intensity in Adolescents with Chronic Musculoskeletal Pain

Emily A. Beckmann^a,*^, Lauren M. Fussner^b^, Sara E. Williams^c^, Anne Lynch-Jordan^c^, Susmita Kashikar-Zuck^c^ and Kristen E. Jastrowski Mano^a^

^a^Department of Psychology, University of Cincinnati, Cincinnati, Ohio, USA; ^b^Yale Child Study Center, Yale School of Medicine, New Haven, Connecticut, USA; ^c^Cincinnati Children’s Hospital Medical Center, Behavioral Medicine & Clinical Psychology, Cincinnati, Ohio, USA

**CONTACT** Emily A. Beckmann beckmaea@mail.uc.edu

© 2020 The Author(s). Published with license by Taylor & Francis Group, LLC.

This is an Open Access article distributed under the terms of the Creative Commons Attribution License (http://creativecommons.org/licenses/by/4.0/), which permits unrestricted use, distribution, and reproduction in any medium, provided the original work is properly cited.

**Introduction/Aim**: Executive functioning deficits (e.g., difficulties in working memory) are often reported by chronic pain patients and relate to functional disability. Among youth, chronic pain is associated with notable impairment in school-related functioning, particularly in the realm of academic performance (i.e. declining grades). Despite the link between working memory and school functioning, this specific relationship has not been investigated in pediatric chronic pain. Therefore, the aim of this study was to investigate the extent to which working memory interferes with academic functioning among adolescents with chronic musculoskeletal (MSK) pain.

**Methods**: Adolescents with chronic MSK pain (*N* = 30, *M_age_ = *14.9, 66.7% female) completed questionnaires assessing executive functioning (Behavior Rating Inventory of Executive Function-2 [BRIEF-2]), average pain intensity (visual analog scale), and academic performance (current grades).

**Results**: To test the hypothesis that working memory accounts for variance in academic performance above and beyond pain intensity, a hierarchical regression analysis was conducted. Academic performance was regressed onto pain intensity in the first step and academic performance was regressed onto working memory in the second step. Results indicated that working memory accounted for an additional 22% of the variance in academic performance above and beyond that of pain intensity [*Δr2 *=.22, *b* =.48, *t*(26) = 2.79, *p* =.01].

**Discussion/Conclusions**: Results suggest that working memory is associated with academic performance above and beyond pain intensity among adolescents with chronic pain. Understanding how specific domains of EF relate to academic performance could inform school-based EF interventions for youth with chronic pain.

## Most Common Non-lethal Adverse Effects Associated with Chronic Pain Treatment: A Cross-sectional Web-based Study in the Quebec Adult Population

Mamadou Diallo^a,*^, Véronique Gagnon^a^, M. Gabrielle Pagé http://orcid.org/0000-0002-7742-2717^b,c^, Line Guénette http://orcid.org/0000-0001-9769-7550^d^, Lucie Blais^e^ and Anaïs Lacasse http://orcid.org/0000-0002-3992-5145^a^

^a^Département des sciences de la santé, Université du Québec en Abitibi-Témiscamingue (UQAT), Rouyn-Noranda, Québec, Canada; ^b^Centre de recherche du Centre hospitalier de l’Université de Montréal (CRCHUM), Montreal, Quebec, Canada; ^c^Département d’anesthésiologie et de médecine de la douleur, Faculté de médecine, Université de Montréal, Montréal, Québec, Canada; ^d^Faculté de pharmacie, Université Laval, Québec, Québec, Canada; ^e^Faculté de pharmacie, Université de Montréal, Montréal, Québec, Canada

**CONTACT** Mamadou Diallo mamadoualiou.diallo@uqat.ca

© 2020 The Author(s). Published with license by Taylor & Francis Group, LLC.

This is an Open Access article distributed under the terms of the Creative Commons Attribution License (http://creativecommons.org/licenses/by/4.0/), which permits unrestricted use, distribution, and reproduction in any medium, provided the original work is properly cited.

**Introduction/Aim**: For many individuals, adverse effects are as important as symptom reduction when deciding whether to use a treatment. In the context where chronic pain (CP) treatment is characterized by off-label prescribing, polypharmacy and multimorbidity, results of premarketing studies should always be complemented by real-world evidence. This study aimed to document the most frequent side effects associated with CP treatment in the context of real-world practice.

**Methods**: Between June and October 2019, a web-based cross-sectional study was conducted in a community sample of adults suffering from CP (Quebec, Canada). Participants completed a standardized checklist about presence and severity (mild, moderate, severe) of 19 side effects that could be associated with their pain medication.

**Results**: Of the 1569 participants who completed the questionnaire about strategies to ease their pain, 93.4% reported using medications (26.6% prescribed medications only, 13.6% over-the-counter medications only, 53.1% both, 6.6% none). Among these participants, fatigue (77.1%), dry mouth (66.3%), drowsiness (64.2%), decreased libido (61.1%), and impaired memory (58.4%) were the most frequently reported side effects. When looking at side effects reported to be severe, the most common were fatigue (27.6%), decreased libido (23.9%), dry mouth (16.9%), insomnia (15.7%), weight gain (14.8%), and constipation (13.6%).

**Discussion/Conclusion**: Our results could help establish priorities in terms of support that could be offered to CP sufferers. Using multivariate analysis, next steps will include the identification of socioeconomic (e.g., sex, gender) and clinical factors (e.g., pain characteristics, use of nonpharmacological treatments, access to care) associated with such side effects.

## Chronic Pain Status Predicts Working Memory Performance among Emerging Adults

Emily A. Beckmann^a,*^, Elana Abelson^a^, Hadas Nahman-Averbuch^b^, Christopher D. King^b^, Robert C. Coghill^b^ and Kristen E. Jastrowski Mano^a^

^a^Department of Psychology, University of Cincinnati, Cincinnati, Ohio, USA; ^b^Behavioral Medicine & Clinical Psychology, Cincinnati Children’s Hospital Medical Center, Cincinnati, Ohio, USA

**CONTACT** Emily A. Beckmann beckmaea@mail.uc.edu

© 2020 The Author(s). Published with license by Taylor & Francis Group, LLC.

This is an Open Access article distributed under the terms of the Creative Commons Attribution License (http://creativecommons.org/licenses/by/4.0/), which permits unrestricted use, distribution, and reproduction in any medium, provided the original work is properly cited.

**Introduction/Aim**: Chronic pain (CP) patients often report cognitive concerns, such as perceived deficits in working memory (WM). WM involves the cognitive maintenance and manipulation of information for short periods of time and is essential for daily functioning. WM has typically been assessed using self-report questionnaires in clinical CP samples or with experimental pain and WM tasks in healthy samples. A key gap in knowledge exists regarding whether individuals with CP show WM deficits on performance-based neuropsychological measures. Therefore, the aim of this study was to determine if CP status predicts performance in an experimental performance-based WM task.

**Methods**: Emerging adults (*N* = 81, *M*_age_ = 18.9, 51.2% female, 78.6% Caucasian, 58.9% with CP) completed a computerized version of an auditory digit span task (WM capacity) and a pain history questionnaire as part of a larger study investigating relationships among cognition, emotion, and pain perception.

**Results**: A linear regression analysis revealed that approximately 11% of the variance in WM capacity was accounted for by CP status (*F*(1, 79) = 5.78, *p* =.02). There was a moderate, negative relationship between CP and WM capacity such that CP (current and/or past) was associated with lower WM capacity (*b* =.26, *t*(79) = 2.41, *p* =.02).

**Discussion/Conclusions**: Results suggest that CP may be associated with WM deficits in terms of both perceived deficits in daily functioning as well as performance on objective WM measures. The immediate and long-term influence of WM on treatment outcomes and functional disability in CP necessitate further investigation.

## Systematic Review of Non-pharmacological Management of Preterm Infant Procedural Pain

Ilana Shiff^a,*^, Oana Bucsea^a^, Rebecca Pillai Riddell^a^, Hannah Gennis^b^, Shaylea Badovinac^b^, Miranda DiLorenzo^b^, Nicole Racine^c^, Sara Ahola Kohut^d^, Diana Lisi^e^, Kara Turcotte^e^, Bonnie Stevens http://orcid.org/0000-0001-5387-2302^f^ and Lindsay Uman^g^

^a^Clinical-Developmental Psychology, York University, Toronto, Ontario, Canada; ^b^Psychology, York University, Toronto, Ontario, Canada; ^c^Psychology, University of Calgary, Calgary, Alberta, Canada; ^d^Gastroenterology, Hepatology and Nutrition, Hospital for Sick Children, Toronto, Ontario, Canada; ^e^Psychology, University of British Columbia | Okanagan, Kelowna, British Columbia, Canada; ^f^Nursing, University of Toronto, Toronto, Ontario, Canada; ^g^Complex Pain Team, IWK Health Centre, Halifax, Nova Scotia, Canada

**CONTACT** Ilana Shiff ishiff@yorku.ca

© 2020 The Author(s). Published with license by Taylor & Francis Group, LLC.

This is an Open Access article distributed under the terms of the Creative Commons Attribution License (http://creativecommons.org/licenses/by/4.0/), which permits unrestricted use, distribution, and reproduction in any medium, provided the original work is properly cited.

**Introduction/Aim**: Preterm infants (born less than 37 weeks gestation) undergo countless painful procedures over the course of hospitalization (Holsti & Grunau, 2007), and pain management is paramount (Pillai Riddell et al., 2015). The aim of this study is to assess the efficacy of non-pharmacological interventions for preterm procedural pain, excluding kangaroo care, music and breastfeeding.

**Methods**: We searched multiple databases for relevant articles published before April 2019. Participants included preterm infants that were less than one month old. Only randomized controlled trials (RCTs) or RCT cross-over designs that had a no-treatment comparison group were eligible for inclusion in the analyses. Studies examining additive effects of an intervention were also included. Analyses were run separately for thirteen different intervention categories and two pain response types (pain reactivity and immediate pain regulation). Each intervention category was comprised of at least two studies.

**Results**: Fourty-five studies were analyzed. The largest SMD for treatment improvement over control conditions on pain reactivity was for multi-sensory bundle interventions (SMD: −1.85; 95% CI: −3.04 to −0.65). For immediate pain regulation, the largest SMDs were: light reducing interventions (SMD: −1.17; 95% CI: −1.54 to −0.8) and swaddling (SMD: −1.58; 95% CI: −2.58 to −0.57). Interestingly, administering non-pharmacological interventions in addition to a sucrose solution had an additive effect for pain reactivity (SMD: −1.25, CI: −2.33 to −0.16) but not for immediate pain regulation.

**Discussion/Conclusions**: Non-pharmacological interventions can be effective for reducing pain reactivity and immediate pain regulation for hospitalized preterm infants, and should continue to be evaluated.

## Parent Version of the Sensitivity to Pain Traumatization Scale: Development, Preliminary Validation, and Longitudinal Examination

Jaimie K. Beveridge^a,*^, Maria Pavlova^a^, Joel Katz http://orcid.org/0000-0002-8686-447X^b^ and Melanie Noel http://orcid.org/0000-0003-3752-8055^a^

^a^Psychology, University of Calgary, Calgary, Alberta, Canada; ^b^Psychology, York University, Toronto, Ontario, Canada

**CONTACT** Jaimie K. Beveridge jaimie.beveridge@ucalgary.ca

© 2020 The Author(s). Published with license by Taylor & Francis Group, LLC.

This is an Open Access article distributed under the terms of the Creative Commons Attribution License (http://creativecommons.org/licenses/by/4.0/), which permits unrestricted use, distribution, and reproduction in any medium, provided the original work is properly cited.

**Introduction/Aim**: Sensitivity to pain traumatization (SPT) is a recently-developed construct measuring the propensity to develop responses to pain that resemble a traumatic stress reaction. SPT may be a risk factor for the co-occurrence of chronic pain and PTSD. Parents of youth with chronic pain report significant PTSD symptoms, with a proportion reporting their child’s chronic pain as their most traumatic event. The Sensitivity to Pain Traumatization Scale (SPTS-12) was recently developed for use in adults. This study aimed to develop and validate the parent version of the Sensitivity to Pain Traumatization Scale (SPTS-P) and examine its relation to future parenting behaviors and child pain-related outcomes.

**Methods**: 141 youth (72.3% girls) aged 10–18 years (*M* = 14.28) referred for tertiary-level treatment of chronic pain and one of their parents (92.2% mothers) completed online surveys at baseline and 3 months later. Parents completed the SPTS-P and measures of PTSD, depression, and parenting behaviors. Youth completed measures of pain and psychosocial functioning.

**Results**: Exploratory factor analysis revealed a one-factor structure for the SPTS-P, consistent with the original SPTS-12, that accounted for 37.2% of the variance. One item had poor fit. The SPTS-P demonstrated good reliability and validity. Parent SPT at baseline was positively related to maladaptive parenting behaviors (protectiveness and monitoring) at follow-up (*r*_s_ =.36-.48, all *p* <.001) and was unrelated to child outcomes at follow-up.

**Discussion/Conclusions**: The SPTS-P is a psychometrically-sound measure. Importantly, parents with higher SPT were more likely to engage in parenting behaviors that are associated with greater disability in youth with chronic pain.

## Paradoxical Thalamic Hypoactivation during Pressure Pain in Hyperalgesic Adolescents with Juvenile Fibromyalgia: A Preliminary fMRI Study

Han Tong^a,*^, Thomas Maloney^b^, Michael Payne^a^, Tracy Ting^c^, Robert Coghill^a^, Susmita Kashikar-Zuck^a^ and Marina Lopez-Sola^a,d^

^a^Division of Behavioral Medicine and Clinical Psychology, Cincinnati Children’s Hospital Medical Center, Cincinnati, Ohio, USA; ^b^Pediatric Neuroimaging Research Consortium, Cincinnati Children’s Hospital Medical Center, Cincinnati, Ohio, USA; ^c^Division of Rheumatology, Cincinnati Children’s Hospital Medical Center, Cincinnati, Ohio, USA; ^d^Serra Hunter Program, Unit of Psychological Medicine, Department of Medicine, School of Medicine and Clinical Sciences, University of Barcelona, Barcelona, Spain

**CONTACT** Han Tong Han.Tong@cchmc.org

© 2020 The Author(s). Published with license by Taylor & Francis Group, LLC.

This is an Open Access article distributed under the terms of the Creative Commons Attribution-NonCommercial License (http://creativecommons.org/licenses/by-nc/4.0/), which permits unrestricted non-commercial use, distribution, and reproduction in any medium, provided the original work is properly cited.

**Introduction**: Juvenile Fibromyalgia (JFM) is a chronic widespread pain condition affecting primarily adolescent girls. Although hypersensitivity to pressure pain has been observed in patients with JFM, its underlying brain mechanisms remain unknown.

**Methods**: We characterized brain responses to pressure pain in 18 adolescents with JFM and 24 healthy adolescents (female, ages 13–17) using fMRI. Participants received 12 noxious pressure stimuli (2.5 or 4 kg/cm2, each lasting 10 s, applied to the left thumbnail) and rated pain intensity and unpleasantness on a 0–100 visual analogue scale.

**Results**: Patients with JFM reported greater pain intensity (Patients: 40.6 ± 23.5, Controls: 26.3 ± 18.2, t = 7.4, *p* < .001) and unpleasantness (Patients: 40.8 ± 24.1, Controls: 26.5 ± 18.6, t = 7.3, *p* < .001). Pressure pain robustly activated all major pain processing regions in healthy adolescents. A similar but more restrictive pain-evoked pattern was found in JFM, without significant thalamic activation (p_FDR-corr_<0.05). Thalamic hypoactivation in patients was confirmed when statistically compared with controls (p_uncorr_<0.001). The neurologic pain signature (Wager TD et al., 2013) was strongly expressed but failed to differentiate the two groups (t = 0.2, *p* = .85). Nevertheless, patients showed a trend for greater expression (t = 1.4, *p* = .09) of a brain signature of adult fibromyalgia pain (Lopez-Sola M et al., 2017).

**Conclusions**: We found a paradoxical attenuation of thalamic responses to pressure pain in hyperalgesic adolescents with JFM, potentially compatible with reductions in peripheral nociceptive input. This finding was paradoxically accompanied by near-normal responses in a brain marker of nociceptive pain and a trend toward over-expression of a brain marker of fibromyalgia pain, suggesting potentially amplified responses in other pronociceptive neural circuits.

## Distinct Healthcare Utilization Trajectories among Chronic Pain Patients: An Application of Group-Based Trajectory Modelling

Hermine Lore Nguena Nguefack^a,*^, M. Gabrielle Pagé http://orcid.org/0000-0002-7742-2717^b,c^, Manon Choinière http://orcid.org/0000-0001-9593-8883^b,c^, Joel Katz^d^, Oumar Mallé Samb^a^ and Anaïs Lacasse http://orcid.org/0000-0002-3992-5145^a^

^a^Département des sciences de la santé, Université du Québec en Abitibi-Témiscamingue (UQAT), Rouyn-Noranda, Québec, Canada; ^b^Centre de recherche du Centre hospitalier de l’Université de Montréal (CRCHUM); ^c^Département d’anesthésiologie et de médecine de la douleur, Faculté de médecine, Université de Montréal, Montréal, Québec, Canada; ^d^Department of Psychology, Faculty of Health, York University, Toronto, Ontario, Canada

**CONTACT** Hermine Lore Nguena Nguefack HermineLore.NguenaNguefack@uqat.ca

© 2020 The Author(s). Published with license by Taylor & Francis Group, LLC.

This is an Open Access article distributed under the terms of the Creative Commons Attribution License (http://creativecommons.org/licenses/by/4.0/), which permits unrestricted use, distribution, and reproduction in any medium, provided the original work is properly cited.

**Introduction/Aim**: Better understanding of inter – and intra-individual differences regarding healthcare resources utilization is essential to grasp sociodemographic inequalities and predictors of poor health outcomes. This study aimed to describe 5-year healthcare utilization trajectories among individuals suffering from chronic pain (CP).

**Methods**: The TORSADE Cohort was used, a research infrastructure containing data about 61 083 individuals living in the province of Quebec and resulting from the linkage between the Canadian Community Health Survey (cross-sectional questionnaires 2007–2008, 2009–2010, 2011–2012) and provincial administrative databases. Among participants reporting suffering from CP in the survey, the annual number of healthcare contacts was computed during the 5 years preceding the questionnaire completion. Group-Based Trajectory Modeling (GBTM) was used to identify subgroups of individuals following similar patterns of healthcare utilization over time.

**Results**: 11 684 individuals with CP were included in this study (mean age: 55.80 ± 17.70; females: 61.74%). Most participants reported moderate pain intensity (53.43%) and activity limitations because of their pain (62.93%). Based on the lowest BIC value, adequate numbers of participants in each group and parsimony, three healthcare utilization trajectories were found: none-and-infrequent users group (48.28%, n = 5641), moderate users group (41.19%, n = 4813) and heavy users group (10.53%, n = 1230). Prevalence of patients who visited a pain clinic during the 5-year follow-up varied across subgroups (none-and-infrequent users: 4.90%, moderate users: 6.75%, heavy users:13.82%; Chi-square *p* < .0001).

**Discussion/Conclusions**: GBTM is an innovative approach to better define healthcare utilization profiles. Next steps will be to examine sociodemographic determinants of healthcare trajectories and their impact on clinical outcomes.

## Neonatal Pain and Maternal Parenting: Cortisol Patterns across Development and Internalizing Behaviors in Children Born Very Preterm

Mia A. McLean http://orcid.org/0000-0003-1186-3699^a,*^, Cecil M. Y. Chau^a^, Joanne Weinberg^b^, Anne Synnes^a^, Steven P. Miller^c^ and Ruth E. Grunau http://orcid.org/0000-0002-5428-9212^a^

^a^Pediatrics, University of British Columbia, Vancouver, BC, Canada; ^b^Cellular & Physiological Sciences, University of British Columbia, Vancouver, BC, Canada; ^c^Pediatrics, University of Toronto, Toronto, Ontario, Canada

**CONTACT** Mia A. McLean mia.mclean@bcchr.ca

© 2020 The Author(s). Published with license by Taylor & Francis Group, LLC.

This is an Open Access article distributed under the terms of the Creative Commons Attribution License (http://creativecommons.org/licenses/by/4.0/), which permits unrestricted use, distribution, and reproduction in any medium, provided the original work is properly cited.

**Introduction/Aim**: Pain of invasive procedures (~10 per day) in the neonatal intensive care unit is associated with altered stress hormone (cortisol) regulation, which may contribute to high internalizing (anxiety/depressive) symptoms in children born very preterm. It is unknown whether parenting behaviors ameliorate effects of neonatal pain on cortisol levels across childhood and later internalizing behaviors in these children. We aimed to examine how early pain is associated with patterns of cortisol expression across early childhood and subsequent internalizing problems at 4.5 years, and the role of mother interactive behaviors, to uncover pathways of resilience in this vulnerable population.

**Methods**: Longitudinal prospective cohort study of infants born very preterm (24–32 weeks gestation) seen at 1.5, 3, 4.5 years. Salivary cortisol was measured three times at each age, and mother-child behaviors filmed (1.5 and 3 years). Internalizing behavior at age 4 was assessed by mother-completed Child Behavior Checklist (CBCL 1.5– 5).

**Results**: Girls of less intrusive mothers were more likely to display stable low cortisol levels across development. Moreover, girls with persistent low cortisol across early childhood were reported by parents to show fewer anxiety and withdrawal behaviors, compared to children with high cortisol across childhood. In boys only, neonatal factors were more related to a pattern of high stable cortisol expression.

**Discussion/Conclusions**: Our findings inform understanding of the role of parenting behaviors in optimizing child stress regulation and internalizing behaviors in children born very preterm. This work advances knowledge of mechanisms of recovery following early life pain in very preterm children.

## Identifying Protective and Risk Factors of Suicidality among Those with Chronic Pain: A Canadian Nationally Representative Study

Rachel Roy^a,*^, Jordana L. Sommer^b^, Renée El-Gabalawy^c^

^a^Department of Anesthesiology, Perioperative and Pain Medicine, University of Manitoba, Winnipeg, Manitoba, Canada; ^a^Department of Psychology, University of Manitoba, Winnipeg, Manitoba, Canada; ^b^Department of Clinical Health Psychology, University of Manitoba, Winnipeg, Manitoba, Canada

**CONTACT** Rachel Roy rroy3@hsc.mb.ca

© 2020 The Author(s). Published with license by Taylor & Francis Group, LLC.

This is an Open Access article distributed under the terms of the Creative Commons Attribution License (http://creativecommons.org/licenses/by/4.0/), which permits unrestricted use, distribution, and reproduction in any medium, provided the original work is properly cited.

**Introduction/Aim**: Research has identified elevated rates of suicidality (ideation, plans, attempts) among those with chronic pain. However, previous research is lacking and primarily focuses on mechanisms increasing rather than decreasing risk. This study aimed to identify risk and protective correlates of suicidality among individuals with chronic pain in a nationally representative Canadian sample.

**Methods**: We analyzed data from the 2012 Canadian Community Health Survey-Mental Health supplement (CCHS-MH; *N *= 25,113). Multiple logistic regressions examined associations between risk (disability, activity limitation) and protective (social support, positive mental health, spirituality) correlates with suicidality among individuals endorsing usual pain/discomfort, while adjusting for sociodemographic characteristics and psychiatric conditions.

**Results**: After adjustment, among those with usual pain/discomfort, greater social support was associated with reduced odds of suicide ideation (AOR = 0.94, 95% CI[0.92–0.96], *p *<.001), plans (AOR = 0.99, 95% CI[0.97–0.99], *p *<.001), and attempts (AOR = 0.95, 95%CI[0.92–0.98], *p *<.05). Positive mental health was also associated with reduced odds of suicide ideation (AOR = 0.97, 95% CI[0.96–0.98], *p *<.05), plans (AOR = 0.99, 95% CI[0.97–0.99], *p *<.05) and attempts (AOR = 0.97, 95% CI[0.96–0.99], *p *=.001). In addition, compared to those whose pain did not prevent any activities, those whose pain prevented most activities had increased odds of suicide plans (AOR = 1.72, 95% CI[1.07–2.78], *p* <.05) and attempts (AOR = 1.94, 95% CI[1.08–3.48], *p* <.05). Additionally, greater disability was associated with increased odds of suicide ideation (AOR = 1.01, 95% CI[1.01–1.02], *p* <.001).

**Discussion/Conclusions**: Results outline important factors associated with suicidality among this population. These findings may inform targeted screening, prevention, and intervention strategies to promote resilience and mitigate risk, among those with chronic pain.

## The ‘Pawsitive’ Impacts of Therapy Dog Visits with Adult Emergency Department Pain Patients: Randomized Controlled Trial

Susan Tupper http://orcid.org/0000-0003-3736-357X^a,*^, Eloise Carr http://orcid.org/0000-0003-1870-4244^b^, Colleen Dell^c^, James Stempien^d^, Betty Rohr^c^, Ben Carey^c^, Maria Cruz^c^, Sharon Acoose^e^, Peter Butt^f^, Lindsey Broberg^g^, Lisa Collard^h^, Logan Fele-Slaferek^g^, Cathie Fornssler^g^, Donna Goodridge^i^, Janet Gunderson^g^, Holly McKenzie^c^, Joe Rubin^j^, Jason Shand^k^, Jane Smith^g^, Jason Trask^h^ and Kerry Ukrainetz^g^

^a^Quality, Safety & Standards, Saskatchewan Health Authority, Saskatoon, Saskatchewan, Canada; ^b^Nursing, University of Calgary, Calgary, Alberta, Canada; ^c^Sociology, University of Saskatchewan, Saskatoon, Saskatchewan, Canada; ^d^Emergency Medicine, University of Saskatchewan, Saskatoon, Saskatchewan, Canada; ^e^School of Indigenous Social Work, First Nations University of Canada, Saskatoon, Saskatchewan, Canada; ^f^Family Medicine, University of Saskatchewan, Saskatoon, Saskatchewan, Canada; ^g^Saskatchewan Centre for Patient Oriented Research, University of Saskatchewan, Saskatoon, Saskatchewan, Canada; ^h^Emergency Medicine, Saskatchewan Health Authority, Saskatoon, Saskatchewan, Canada; ^i^Respirology, Critical Care and Sleep Medicine, University of Saskatchewan, Saskatoon, Saskatchewan, Canada; ^j^Veterinary Microbiology, University of Saskatchewan, Saskatoon, Saskatchewan, Canada; ^k^Clinical Analyst, Saskatchewan Health Authority, Saskatoon, Saskatchewan, Canada

**CONTACT** Susan Tupper susan.tupper@usask.ca

© 2020 The Author(s). Published with license by Taylor & Francis Group, LLC.

This is an Open Access article distributed under the terms of the Creative Commons Attribution License (http://creativecommons.org/licenses/by/4.0/), which permits unrestricted use, distribution, and reproduction in any medium, provided the original work is properly cited.

**Introduction/Aim**: Pain is a primary reason individuals attend an Emergency Department (ED). Long wait times negatively impact patients’ experience of pain. Change in symptoms and physiologic variables were measured at 3 time points pre/post a 10 minute therapy dog visit in ED patients who experienced pain.

**Methods**: Using a randomized controlled design, pain, anxiety, depression and well-being were measured with an 11-point rating scale before, immediately after, and 20 minutes post-visit. Blood pressure and heart rate were recorded at the time points. Control data was gathered twice (30 minutes apart). A two-way ANOVA was conducted to compare effects of dog therapy condition on the dependent variables.

**Results**: Participants (n = 198) were 41% female, with a mean age of 58 years. Pain was shown to decrease significantly (mean change = 0.9, SD = 2.05) or almost one value lower on a 0 to 10 scale, in the intervention group compared to no change in control [F(1,193) = 8.54, *p* = .004, η_p_2 = .04]. Anxiety also lowered one numerical value with the intervention compared to control (mean change = 1.13, SD = 2.80) [F(1,193) = 7.95, *p* = .005, η_p_2 = .04]. Depression (mean change = 0.72, SD = 1.71) and well-being ratings (mean change = 0.87, SD = 1.84) similarly decreased for the intervention group. Blood pressure and heart rate had no significant differences in both groups.

**Discussion/Conclusions**: Clinically significant changes in pain were observed in the therapy dog intervention compared to control. The findings of this novel study contribute important knowledge toward the potential value of ED therapy dogs to affect patients’ experience of pain, and related measures of anxiety, depression and well-being.

## Pain and Affect in Multiple Sclerosis: Contributions of the Kappa Opioid System

Caylin I. Chadwick^a,*^, Zoë Dworsky-Fried http://orcid.org/0000-0001-5900-4442^b^, Bradley J. Kerr^c^ and Anna M. W. Taylor^b^

^a^Neuroscience and Mental Health Institute, University of Alberta, Edmonton, Alberta, Canada; ^b^Pharmacology, University of Alberta, Edmonton, Alberta, Canada; ^c^Anesthesiology and Pain Medicine, University of Alberta, Edmonton, Alberta, Canada

**CONTACT** Caylin I. Chadwick caylin@ualberta.ca

© 2020 The Author(s). Published with license by Taylor & Francis Group, LLC.

This is an Open Access article distributed under the terms of the Creative Commons Attribution-NonCommercial License (http://creativecommons.org/licenses/by-nc/4.0/), which permits unrestricted non-commercial use, distribution, and reproduction in any medium, provided the original work is properly cited.

**Introduction/Aim**: Multiple Sclerosis (MS) is a debilitating disease in which inflammation and autoimmune reactions result in demyelination within the central nervous system (CNS). Neuropathic pain and negative affect are two symptoms commonly experienced with MS. The kappa opioid receptor (KOR) is involved in modulating mood and pain sensation, and changes in KOR function have been described in several models of chronic pain. Here, we aim to characterize changes in KOR expression and function in a model of MS.

**Methods**: Male and female C57Bl/6J mice were induced with experimental autoimmune encephalomyelitis (EAE), which models the inflammation, autoimmunity, and demyelination that occurs in human MS. The analgesic efficacy of the KOR agonist U50,488H (1.6–30 mg/kg, i.p.) was measured using von Frey filaments. At first signs of clinical symptoms, brains and spinal cords were isolated and fluorescent *in situ* hybridization (FISH) used to quantify KOR and dynorphin mRNA levels. Western blots were used to measure changes in KOR protein expression.

**Results**: Mice with EAE showed significantly less U50,488H analgesia compared to control mice. This correlated with a reduction in KOR mRNA and total protein levels in the spinal cord. Dynorphin mRNA levels were not significantly changed.

**Discussion/Conclusions**: These data indicate that KOR agonists are less effective in EAE. This loss of analgesic activity correlated with a reduction in protein and mRNA expression in the spinal cord. These results are in contrast to what have been described in other models of neuropathic pain, and suggest unique disease mechanisms in this animal model of MS.

## Long-term Musculoskeletal Pain Outcomes after Intrathecal Baclofen Pump Implant for Spasticity Management in Children with Cerebral Palsy

Cole Hagen^a,*^, Lisa Lykken^a^, Alyssa Merbler^b^, Chantel C. Barney^a,b^ and Frank Symons^b^

^a^Department of Research Administration, Gillette Children’s Specialty Healthcare, Research, Saint Paul, Minnesota, USA; ^b^Department of Educational Psychology, University of Minnesota, Minneapolis, Minnesota, USA

**CONTACT** Cole Hagen colehagen@gillettechildrens.com

This work was supported by Eunice Kennedy Shriver NICHD Grant No. 73126

© 2020 The Author(s). Published with license by Taylor & Francis Group, LLC.

This is an Open Access article distributed under the terms of the Creative Commons Attribution License (http://creativecommons.org/licenses/by/4.0/), which permits unrestricted use, distribution, and reproduction in any medium, provided the original work is properly cited.

**Introduction/Aim**: Chronic muscle tightness (i.e., spasticity) is associated with musculoskeletal pain in individuals with cerebral palsy (CP). Intrathecal baclofen (ITB) pumps are used for spasticity management. Previous studies have reported reduced tone and associated pain outcomes within the first 18 months after implant; however, no evidence describes longer-term ITB related pain outcomes.

**Methods**: We prospectively assessed the long term pain outcomes following ITB pump implant in 16 individuals with CP (mean age = 11.93 years). The majority of participants had a CP diagnosis of quadriplegia (81.25%) and relied on wheeled mobility (87.5%; GMFCS level V). Assessments were completed immediately before and 3.91 years (SD = 1.40 years) after ITB implant. For all participants, pain (Brief Pain Inventory [BPI]; Dalhousie Pain Interview [DPI]) and spasticity burden (Multiple Sclerosis Spasticity Scale [MSSS-88] muscle stiffness and pain/discomfort subscales) assessments were completed by parent-report.

**Results**: A repeated measures t-test demonstrated a significant decrease in pain duration from before implant (M = 63.26 hours, SD = 83.78 hours) to long-term follow-up (M = 8.49 hours, SD = 29.10 hours; t(15) = 2.74 *p* = .015). There was also a significant decrease in spasticity burden from before implant (M = 57.13, SD = 4.06) to long-term follow-up (M = 43.73, SD = 3.91; t(14) = 4.02, *p* = .001). Pain interference and pain intensity displayed no significant change at long-term follow-up. In this pilot use of the MSSS-88 in CP, the scale demonstrated preliminary validity as scores correlated with pain interference (r = 0.48, *p* = .006) and intensity (r = 0.47, *p* = .007).

**Discussion/Conclusions**: These initial analyses suggest that the immediate reduction in pain duration and spasticity burden previously reported are still present nearly four years after ITB implant.

## Do Sleep Problems Contribute to Opioid Misuse Behaviours among Patients with Chronic Pain on Long-term Opioid Therapy?

Alberto Herrero Babiloni http://orcid.org/0000-0001-5588-5916^a,*^, Leah Frimerman^a^, Maria Verner^b^, Amanda Sirois^b^, Katherine Scott^c^, Jordi Perez^d^, Yoram Shir^e^, Gilles J. Lavigne^f^ and Marc O. Martel^g^

^a^Division of Experimental Medicine, McGill University, Montreal, Québec, Canada; ^b^Faculty of Dentistry, McGill University, Montreal, Québec, Canada; ^c^Department of Physiology, McGill University, Montreal, Québec, Canada; ^d^Department of Anesthesia, McGill University, Montreal, Québec, Canada; ^e^Department of Anesthesia, Faculty of Dentistry, McGill University, Montreal, Québec, Canada; ^f^CIUSSS Nord Ile Montreal-Sacré-Coeur Hospital, Faculty Dental Medicine, Universite de Montreal, Faculty of Dentistry, McGill University, Montreal, Québec, Canada; ^g^Department of Anesthesia, Faculty of Dentistry, Division of Experimental Medicine, McGill University, Montreal, Québec, Canada

**CONTACT** Alberto Herrero Babiloni alberto.herrerobabiloni@mail.mcgill.ca Division of Experimental Medicine, McGill University, Montreal, Québec, Canada

© 2020 The Author(s). Published with license by Taylor & Francis Group, LLC.

This is an Open Access article distributed under the terms of the Creative Commons Attribution License (http://creativecommons.org/licenses/by/4.0/), which permits unrestricted use, distribution, and reproduction in any medium, provided the original work is properly cited.

**Introduction/Aims:** Studies have revealed alarmingly high rates of opioid misuse among patients with chronic pain. To date, the contribution of sleep problems to opioid misuse in these patients has yet to be systematically investigated. The main objective of this study was to examine the associations between sleep quality and different types of opioid misuse behaviors among patients with chronic pain prescribed opioids. We also examined the factors that could mediate associations between sleep quality and opioid misuse behaviors.

**Methods:** In this longitudinal diary study, 103 patients with chronic pain prescribed opioids were recruited. Patients completed measures of sleep quality, pain intensity, negative affect, and opioid craving for 14 consecutive days. Saliva samples were also collected to assess cortisol (i.e., HPA axis) activity, and opioid misuse behaviors (i.e., opioid overuse, using opioids for other reasons than pain, borrowing opioids) were assessed using a validated self-report instrument.

**Results:** Poorer sleep quality was associated with opioid overuse (−.29, *p* < .01), but not with any other types of opioid misuse behaviors (both p’s > .05). Poorer sleep quality was associated with heightened pain intensity (*p* < .001), negative affect (*p* < .001), and opioid craving (*p* < .001), but the association between sleep quality and opioid overuse was neither mediated by these variables nor by HPA axis activity (all p’s > .05).

**Conclusions:** Our findings suggest that sleep problems might lead to the overuse of opioids. Interventions designed to improve sleep quality might contribute to reducing opioid misuse among patients with pain.

## Cerebrospinal Fluid Interleukin-8 and Self-reported Postoperative Pain Levels of Adolescents with or without Pain before Surgery

Shajenth Premachandran^a,*^, Kelsey Vickers^b^, Jean A. Ouellet^c^ and Catherine E. Ferland^d^

^a^Experimental Surgery, McGill University, Montreal, Quebec, Canada; ^b^Anatomy and Cell Biology, McGill University, Montreal, Quebec, Canada; ^c^Orthopedics, Shriners Hospital for Children-Canada, McGill University, Montreal, Quebec, Canada; ^d^Anesthesia, Shriners Hospital for Children-Canada, McGill University, Montreal, Quebec, Canada

**CONTACT** Shajenth Premachandran shajenth.premachandran@mail.mcgill.ca

© 2020 The Author(s). Published with license by Taylor & Francis Group, LLC.

This is an Open Access article distributed under the terms of the Creative Commons Attribution License (http://creativecommons.org/licenses/by/4.0/), which permits unrestricted use, distribution, and reproduction in any medium, provided the original work is properly cited.

**Introduction/Aim**: A preoperative pain biomarker would provide an objective measure of pain intensity to help identify patients at risk of poor pain management and provide better targeting pharmacological treatments before surgery. Interleukin-8 (IL-8) is studied as a pro-inflammatory biomarker of pain and is shown to be upregulated in the cerebrospinal fluid (CSF) in acute and chronic pain states. The aim of this study is to evaluate IL-8 as a proxy of pain between patients with and without pain prior to a major orthopedic surgery.

**Methods**: Seventy patients with adolescent idiopathic scoliosis scheduled to undergo corrective surgery were recruited in this study. CSF samples were collected before opioid injection on the day of surgery, processed and stored pending analysis. Patients self-reported their pain scores on the morning of surgery, 24 hours, 48 hours and 6 weeks after surgery using a numerical rating scale. Chi-squared tests were utilized to identify differences in patients reporting pain and no pain between low/high IL-8 concentration groups.

**Results**: Mean IL-8 concentration was 36.70 ± 15.98 pg/ml (Range: 11.25–106.7 pg/ml). Pain ratings increased 24 hours (mean = 4.09 ± 2.27, *p* < .0001) and 48 hours (mean = 4.23 ± 2.27, *p* < .0001) postoperatively. No differences were observed between patients reporting pain and no pain in low/high IL-8 groups both preoperatively (*p* = .2836) and postoperatively 24 hours(*p* = .7893), 48 hours(*p* = .2044) and 6 weeks(*p* = .7369) after surgery.

**Discussion/Conclusions**: These findings suggest that CSF IL-8 is not a reliable proxy of preoperative pain in pediatric patients undergoing orthopedic surgery. This may be due to the synergistic effect of multiple combinations of anesthetics given to subjects prior to CSF collection which can alter IL-8 transcription.

## Organizational Contextual Factors that Influence the Implementation of an Online, Multifaceted Intervention for Improving Evidence-based Pain Practices in the NICU

Shelly-Anne Li^a,b,*^, Melanie Barwick^b,c^, Lianne Jeffs^d^ and Bonnie Stevens^e,f^

^a^Lawrence S Bloomberg Faculty of Nursing, University of Toronto, Toronto, Ontario, Canada; ^b^The Hospital for Sick Children, Toronto, Ontario, Canada; ^c^Department of Psychiatry, Faculty of Medicine, Dalla Lana School of Public Health, University of Toronto, Toronto, Ontario, Canada; ^d^Lunenfeld-Tanenbaum Research Institute, Sinai Health, Institute of Health Policy Management and Evaluation, University of Toronto, Toronto, Ontario, Canada; ^e^Lawrence S Bloomberg Faculty of Nursing, Faculties of Medicine and Dentistry, University of Toronto, Toronto, Ontario, Canada; ^f^Research Institute, The Hospital for Sick Children, Toronto, Ontario, Canada

**CONTACT** Shelly-Anne Li shellyanne.li@mail.utoronto.ca

© 2020 The Author(s). Published with license by Taylor & Francis Group, LLC.

This is an Open Access article distributed under the terms of the Creative Commons Attribution License (http://creativecommons.org/licenses/by/4.0/), which permits unrestricted use, distribution, and reproduction in any medium, provided the original work is properly cited.

**Introduction/Aim**: The complex, high-stress neonatal intensive care unit (NICU) environment poses challenges for optimal pain management for healthcare providers (HCPs) including the demanding workload, unpredictable patient conditions, and frequent distractions and interruptions. The NICU environment manifests organizational contextual factors (e.g., organizational culture, resources, leadership) that can influence or modify the successful implementation and delivery of neonatal pain practices. We explored stakeholder perspectives (HCPs, unit managers, senior leaders) about how organizational contextual factors influenced the implementation of the Infant Pain Practice Change (ImPaC) Resource, a multifaceted web-based strategy targeted to change pain practices, in the NICU. The aim was to collect data from the first of four data collection sites to inform future data collection processes.

**Methods**: One-hour individual, semi-structured interviews were held with five stakeholders (2 HCPs, 1 NICU manager, 2 senior leaders) at a single Canadian, academically affiliated pediatric hospital. The interviews were guided by the Consolidated Framework for Implementation Research, and data were analyzed using an interpretive descriptive approach to qualitative analysis.

**Results**: Factors associated with effective implementation of the ImPaC Resource included the development of an interdisciplinary pain committee, a culture open to change and excellence in service delivery, available resources (dedicated time, funding, personnel), advocacy from senior leaders, buy-in from unit managers, and prioritization of pain prevention and treatment.

**Discussion/Conclusions**: Findings highlight key factors considered by HCPs, senior leaders and middle managers as associated with effective implementation of the ImPaC Resource in the NICU.

## Talk More, Repress Less? Parental Beliefs about Reminiscing with Young Children about Pain

Madison Kennedy^a,*^, Maria Pavlova^a^, Tatiana Lund^a^ and Melanie Noel http://orcid.org/0000-0003-3752-8055^b^

^a^Department of Psychology, University of Calgary, Calgary, Alberta, Canada; ^b^Department of Psychology, Alberta Children’s Hospital Research Institute (Behaviour and the Developing Brain Theme), Hotchkiss Brain Institute, Mathison Centre for Mental Health Research and Education, University of Calgary, Calgary, Alberta, Canada

**CONTACT** Madison Kennedy madison.kennedy@ucalgary.ca

© 2020 The Author(s). Published with license by Taylor & Francis Group, LLC.

This is an Open Access article distributed under the terms of the Creative Commons Attribution License (http://creativecommons.org/licenses/by/4.0/), which permits unrestricted use, distribution, and reproduction in any medium, provided the original work is properly cited.

**Introduction/Aim**: Painful events provide powerful social learning opportunities for children. For younger children, parents are the primary socialization agents of painful events. Previous research has shown that parents reminisce less adaptively about past painful versus sad events, raising questions about the socio-cultural attitudes about, and avoidance of, pain. Further, parent-child reminiscing about past pain has been linked to children’s behavioral expression of empathy for others’ pain. However, little is known about *why* parents avoid discussing pain with their children. The present study examined parental meta-beliefs about discussing pain and their links to parent-child reminiscing and socio-emotional skills.

**Methods**: Parental meta-beliefs were elicited through interviews with 113 parents of 4-year old children, analyzed using thematic analysis and coded for overall avoidance of discussing pain. Parents also reported on their children’s socio-emotional functioning and reminisced about a past painful event with their child. Relations between reminiscing elements and socio-emotional functioning were analyzed.

**Results**: Thematic analysis revealed common parental beliefs about risks and benefits of discussing pain (e.g., normalizing painful experiences, teaching empathy, or instilling fear). Parents who believed in the benefits of discussing pain used elements of adaptive reminiscing with their children. Further, their children had better developed theory of mind, and prosocial and emotion-regulation skills (*r*s ranged |.21-.26|, *p*s <.05).

**Discussion/Conclusions**: Parents hold diverse meta-beliefs about discussing pain, which are associated with their reminiscing styles and children’s socio-emotional functioning. Better understanding of parental meta-beliefs may elucidate the process of pain socialization in children and its role in children’s pain trajectories.

## Usual Pain and Discomfort is Differentially Associated with Suicidality across Pain Conditions: Results from a Nationally Representative Sample

Bronwen Grocott^a,*^, Jordana L. Sommer^b^ and Renée El-Gabalawy^c^

^a^University of Manitoba, Psychology, Winnipeg, Manitoba, Canada; ^b^University of Manitoba, Psychology and Anesthesiology, Perioperative and Pain Medicine, Winnipeg, Manitoba, Canada; ^c^University of Manitoba, Clinical Health Psychology and Anesthesiology, Perioperative and Pain Medicine, Winnipeg, Manitoba, Canada

**CONTACT** Bronwen Grocott grocottb@myumanitoba.ca

© 2020 The Author(s). Published with license by Taylor & Francis Group, LLC.

This is an Open Access article distributed under the terms of the Creative Commons Attribution License (http://creativecommons.org/licenses/by/4.0/), which permits unrestricted use, distribution, and reproduction in any medium, provided the original work is properly cited.

**Introduction/Aim**: Chronic pain is associated with suicidality (ideation, plans, attempts), a relationship not fully explained by psychiatric comorbidity. This study described characteristics of chronic pain (presence of usual pain/discomfort) and the prevalence of suicidality across pain conditions (arthritis, migraine, back pain). Associations between usual pain/discomfort and suicidality across pain conditions were also examined.

**Methods**: We analyzed data from the 2012 Canadian Community Health Survey-Mental Health supplement (*N* = 25,113). Weighted cross-tabulations described pain characteristics and the prevalence of suicidality according to pain condition. Multiple logistic regressions examined associations between the presence of usual pain/discomfort (reference = absence of usual pain/discomfort) and suicidality among each pain condition.

**Results**: Across pain conditions, the prevalence of usual pain/discomfort ranged from 40-55%. The prevalence of suicide ideation, plans and attempts was 15-24%, 6-10%, and 5-9%, respectively, among individuals with pain conditions. After adjusting for sociodemographics and psychiatric conditions, usual pain was associated with significantly increased odds of suicide ideation (AOR = 1.79, 95% CI [1.19–2.68], *p* <.05) and attempts (AOR = 2.49, 95% CI [1.25–4.98], *p* <.05) among those with migraines, and ideation (AOR = 1.59, 95% CI [1.23–2.05], *p* <.001), plans (AOR = 1.55, 95% CI [1.04–2.31], *p* <.05), and attempts (AOR = 1.68, 95% CI [1.19–2.58], *p* <.05) in those with back pain, compared to the absence of usual pain. Usual pain was not associated with suicidality among those with arthritis.

**Discussion/Conclusions**: By elucidating the complex nature of suicidality among those with chronic pain, our findings may facilitate a more targeted approach to screening in this population.

## Impact of Weight Terminology and Education on Parental Response to Pediatric Pain Treatment Information

Alison M. Stoner^a,*^, Kristen E. Jastrowski Mano^b^, Katrina R. Piangerelli^a^, Johanna R. Michlig^c^, Alexis Cleland^d^, Christina M. Hopkins^e^, Steven Weisman^f^, W. Hobart Davies^d^ and Keri R. Hainsworth^f^

^a^Psychology, Assumption College, Worcester, Massachusetts, USA; ^b^Psychology, University of Cincinnati, Cincinnati, Ohio, USA; ^c^Children’s Hospital of Wisconsin, Jane B. Pettit Pain and Headache Center, Milwaukee, Wisconsin, USA; ^d^Psychology, University of Wisconsin-Milwaukee, Milwaukee, Wisconsin, USA; ^e^Psychology, Duke University, Durham, North Carolina, USA; ^f^Anesthesiology, Medical College of Wisconsin, Milwaukee, Wisconsin, USA

**CONTACT** Alison M. Stoner am.stoner@assumption.edu

© 2020 The Author(s). Published with license by Taylor & Francis Group, LLC.

This is an Open Access article distributed under the terms of the Creative Commons Attribution License (http://creativecommons.org/licenses/by/4.0/), which permits unrestricted use, distribution, and reproduction in any medium, provided the original work is properly cited.

**Introduction/Aim:** It is important that pain providers speak with patients/parents about the link between obesity and chronic pain in youth. The specific weight term used when speaking with adults impacts treatment initiation. The aim of this study was to investigate parental response to pain physician’s use of weight terminology and education when conveying pain-related information.

**Methods:** Participants (*N* = 268 parents) read a vignette about a parent bringing his/her adolescent daughter to a pediatric pain provider, and completed a 14-item questionnaire about pain treatment. Vignettes varied according to a 2 × 3 design (pain/obesity education: presence/absence; weight term: obesity, excess body weight, BMI). An exploratory factor analysis (EFA) of questionnaire items was conducted to increase the reliability of the dependent variables. A two-group between-subjects multivariate analysis of variance (MANOVA) was conducted.

**Results:** EFA yielded a two-factor solution: acceptability of information and personal responsibility toward treatment plan. There was a significant main effect of weight term (*p* < .05) with term specifically affecting Acceptability (*p* < .05), but not Personal Responsibility (*p* > .05). Parents found the physician’s message more acceptable when the term “BMI” was used, compared to when “obesity” was used. Whether or not the physician provided education regarding the relationship between pediatric chronic pain and obesity did not impact parental perceptions.

**Discussion/Conclusions:** Results suggest that weight terminology significantly influences parents’ perception of the acceptability of the physician’s message. Use of a more clinical/stigmatizing term (i.e., obesity) results in reduced acceptability of the message, which may negatively impact adherence to pain treatment plans and continuity of care.

## Child in Pain? How Did You Decide? Respite Workers’ Responses to a Vignette Presenting a Child with a Developmental Disability

Hiba Nauman^a,*^, Lara Genik^a^ and C. Meghan McMurtry http://orcid.org/0000-0002-3278-1169^a,b^

^a^Department of Psychology, University of Guelph, Guelph, Ontario, Canada; ^b^Pediatric Chronic Pain Program, McMaster Children’s Hospital, Hamilton, Ontario, Canada

**CONTACT** Hiba Nauman hnauman@uoguelph.ca

© 2020 The Author(s). Published with license by Taylor & Francis Group, LLC.

This is an Open Access article distributed under the terms of the Creative Commons Attribution License (http://creativecommons.org/licenses/by/4.0/), which permits unrestricted use, distribution, and reproduction in any medium, provided the original work is properly cited.

**Introduction/Aim**: Due to the presence of cognitive and/or communication deficits, children with intellectual/developmental disabilities (I/DD) typically rely on caregivers to interpret and make decisions about their pain. Respite workers are common secondary caregivers of children with I/DD. Using vignette methodology, we sought to examine whether receiving a ~ 4-hour pain or control training impacted respite workers’ determination of the presence/absence of pain and the factors considered.

**Methods**: As part of a larger randomized controlled trial (#NCT03421795), 158 respite workers were randomly assigned to receive a pain specific (58 female, 8 male; *M_age_*: 29.78) or general care control training (“F-words in childhood disability”; 81 female, 10 male; *M_age_*: 31.30). Prior to the training and at follow up (4–6 weeks later), participants read a vignette involving a nonverbal child and indicated whether they believed the child was in pain (yes/no/maybe) and what factors influenced their decision. Frequency and Chi-square analyses were used to examine responses and compare them across groups, respectively.

**Results**: No significant differences existed between groups in decisions about the presence of pain at pre-training (X^2^ = 0.37, *p* =.83) or follow up (X^2^ = 0.65, *p* =.721). At follow up, both pain training (30.8%) and control groups (32.3%) most commonly reported considering knowledge of the child (i.e. likes/dislikes/temperament) whereas participants who received the pain training were more likely to report considering environmental/situational factors (X^2^ = 5.26, *p* <.05).

**Discussion/Conclusions**: Pain training led to increased consideration of environmental factors suggesting it may promote awareness of the role of social/contextual factors in pain.

## Examining Factors Related to the Uptake of a Parent-directed Knowledge Translation Resource on Vaccination Pain Management Strategies

Nicole E. MacKenzie^a^, Perri R. Tutelman^a^, Christine T. Chambers http://orcid.org/0000-0002-7138-916X^a,*^, Jennifer A. Parker http://orcid.org/0000-0001-9900-4703^b^, Melanie Barwick^c^, Kathryn A. Birnie^d^, Katelynn E. Boerner^e^, Vera Granikov^f^, Noni MacDonald^g^, C. Meghan McMurtry http://orcid.org/0000-0002-3278-1169^h,i^, Pierre Pluye^f^ and Anna Taddio^j^

^a^Department of Psychology and Neuroscience, Dalhousie University, Halifax, Nova Scotia, Canada; ^b^Centre for Pediatric Pain Research, IWK Health Centre, Halifax, Nova Scotia, Canada; ^c^Department of Psychiatry, Research Institute, Hospital for Sick Children and University of Toronto, Toronto, Ontario, Canada; ^d^Lawrence S. Bloomberg Faculty of Nursing, CPsych University of Toronto, Toronto, Ontario, Canada; ^e^Department of Psychiatry, BC Children’s Hospital & University of British Columbia, Vancouver, British Columbia, Canada; ^f^Department of Family Medicine, McGill University, Montreal, Quebec, Canada; ^g^Department of Pediatrics, Dalhousie University, Halifax, Nova Scotia, Canada; ^h^Psychology, University of Guelph, Guelph, Canada; ^i^Pediatric Chronic Pain Program, McMaster Children’s Hospital, Hamilton, Ontario, Canada; ^j^Leslie Dan Faculty of Pharmacy, University of Toronto, Toronto, Ontario, Canada

**CONTACT** Christine T. Chambers christine.chambers@dal.ca

© 2020 The Author(s). Published with license by Taylor & Francis Group, LLC.

This is an Open Access article distributed under the terms of the Creative Commons Attribution License (http://creativecommons.org/licenses/by/4.0/), which permits unrestricted use, distribution, and reproduction in any medium, provided the original work is properly cited.

**Introduction/Aim:** Parents’ use of evidence-based strategies for managing children’s vaccination pain can be influenced through knowledge translation (KT) interventions. This study assessed factors related to parents’ planned, actual, and future use of pain management strategies at a vaccination appointment following exposure to a vaccination pain management KT intervention.

**Methods:** Parents of children aged 0–17 reviewed an evidence-based information sheet on vaccination pain management strategies disseminated through an online survey. They reported their impression of the information sheet and plans to use the pain management strategies at an upcoming vaccination via an online survey based on the Information Assessment Method for Parents. Following their child’s vaccination, parents completed another survey and reported their actual strategy use, confidence, and plans for future use.

**Results:** 128 parents completed both surveys. Most parents who planned to use a pain management strategy reported actual use during their child’s vaccination (90%, *n* = 115). Parents who found the information relevant were 9.76 times more likely to use at least one recommendation. Parents who felt confident in their use of strategies during vaccination were 4.23 times more likely to plan to continue using strategies at future vaccinations.

**Discussion/Conclusions:** This study supports the effectiveness of parent-directed knowledge translation interventions to promote uptake of evidence-based pain management strategies, while also supporting parents’ confidence and plans to continue using strategies. These findings highlight the importance of creating KT interventions which are relevant to parents and easily accessible in order to promote uptake of evidence-based vaccination pain management strategies for children.

## Persistent Postmastectomy Sensory Disturbance: Association with Persistent Pain Severity, Psychosocial Functioning, and Temporal Summation of Pain

Kelsey Mikayla Flowers^a,*^, Valerie Hruschak^a^, Emily Schwartz^a^, Robert Edwards^a^ and Kristin Schreiber^a^

^a^Department of Anesthesiology, Perioperative and Pain Medicine at Harvard Medical School, Brigham and Women’s Hospital, Boston, Massachusetts, USA

**CONTACT** Kelsey Mikayla Flowers kmflowers@bwh.harvard.edu

© 2020 The Author(s). Published with license by Taylor & Francis Group, LLC.

This is an Open Access article distributed under the terms of the Creative Commons Attribution License (http://creativecommons.org/licenses/by/4.0/), which permits unrestricted use, distribution, and reproduction in any medium, provided the original work is properly cited.

**Introduction/Aim**: Chronic sensory disturbance following breast cancer surgery can have a significant functional impact on patients’ lives. Neuropathic symptoms ranging from hypoesthesia to spontaneous pain and hyperalgesia may result from surgical injury and are often associated with greater acute and chronic pain severity. Furthermore, somatization, which represents a heightened attention to physical symptoms, may also modulate the degree of impact sensory disturbances have.

**Methods**: Patients undergoing lumpectomy or mastectomy with or without axillary surgery (sentinel node or axillary dissection) completed electronic questionnaires preoperatively and at postoperatively at 2-weeks, 3-months, 6 – and 12-months. The Breast Cancer Pain Questionnaire (BCPQ) longitudinally assessed pain severity, frequency, and degree of sensory disturbance (questions similar to S-LANSS and DN4). The Brief Symptom Index 18 was used to assess somatization.

**Results**: Mean sensory disturbance was highest at 2-weeks post-op and decreased over time. Greater sensory disturbance was associated with axillary lymph node dissection (β = 1.07, *p* < .001), higher baseline pain burden index (β = .050, *p* < .001), higher somatization (β = .102, *p* = .05), and greater mean temporal summation on preoperative bedside QST (β = .102, *p* = .04). Interestingly, there was a negative association between painkiller effectiveness and sensory disturbance, suggesting that painkillers are less effective amongst those with more neuropathic symptomatology (*Rho *= − .322, *p* = .005).

**Discussion/Conclusions**: The degree of sensory disturbance experienced by patients was predicted by younger age, axillary surgery, preoperative pain, somatization, and temporal summation on QST. Future perioperative interventions to reduce nerve injury and treat neuropathic symptoms may well be targeted to these higher risk individuals to minimize or prevent chronic post-mastectomy sensory disturbance and pain.

## The Trajectory of Pain Catastrophizing in Breast Cancer Patients after Lumpectomy, Mastectomy, and Mastectomy with Reconstruction

Valerie Hruschak^a,*^, Kelsey Mikayla Flowers^a^, Emily Schwartz^a^, Robert Edwards^a^ and Kristin Schreiber^a^

^a^Department of Anesthesiology, Perioperative and Pain Medicine at Harvard Medical School, Brigham and Women’s Hospital, Boston, Massachusetts, USA

**CONTACT** Valerie Hruschak Vhruschak@bwh.harvard.edu

© 2020 The Author(s). Published with license by Taylor & Francis Group, LLC.

This is an Open Access article distributed under the terms of the Creative Commons Attribution License (http://creativecommons.org/licenses/by/4.0/), which permits unrestricted use, distribution, and reproduction in any medium, provided the original work is properly cited.

**Introduction/Aim**: Pain catastrophizing has been associated with greater pain severity, longevity, and impact in various types of persistent pain, including postsurgical pain. We investigated the trajectory of pain catastrophizing (rumination, magnification, and helplessness) regarding pain, and its relation to general somatization and pain severity in the first year after breast surgery.

**Methods**: Women (n = 259) undergoing lumpectomy, simple mastectomy, and mastectomy with reconstruction were recruited preoperatively to complete validated questionnaires capturing demographics, psychosocial characteristics, including catastrophizing (Pain Catastrophizing Scale), somatization (Brief Symptoms Inventory-18), and affect (Positive and Negative Affect Schedule). Additionally, a brief bedside sensory test of temporal summation of mechanical pinprick pain was conducted. Pain and psychosocial variables were assessed postoperatively out to 1 year after surgery.

**Results**: Participants were female, predominantly Caucasian (86.4%), and with a mean age of 55.5 years old. Pain catastrophizing was highest preoperatively and decreased significantly over time. Pain catastrophizing was significantly higher amongst patients who underwent mastectomy with reconstruction. Generalized Estimating Equations revealed that higher catastrophizing was independently associated with increased pain (β = 0.12, *p* = <0.001), somatization (β = 0.47, *p* = < 0.001), negative affect (β = 0.27, *p* = < 0.001), and temporal summation (β = 0.47, *p* = .001).

**Discussion/Conclusions**: These findings illustrate that pain catastrophizing varies substantially amongst individuals having surgery for breast cancer, however plays a more prominent role amongst women having reconstructive surgery. Higher preoperative pain catastrophizing scores in the preoperative period suggests that preoperative interventions directed toward mitigation of aspects of catastrophizing may have the potential to improve pain outcomes after this type of surgery.

## Prediction of Risk Factors to Sleep Disturbance after Breast Surgery: A Longitudinal Prospective Study

Emily Schwartz^a,*^, Megan Patton^a^, Valerie Hruschak^a^, Kelsey Mikayla Flowers^a^, Robert Edwards^a^ and Kristin Schreiber^a^

^a^Department of Anesthesiology, Perioperative and Pain Medicine at Harvard Medical School, Brigham and Women’s Hospital, Boston, Massachusetts, USA

**CONTACT** Emily Schwartz eschwartz6@bwh.harvard.edu

© 2020 The Author(s). Published with license by Taylor & Francis Group, LLC.

This is an Open Access article distributed under the terms of the Creative Commons Attribution License (http://creativecommons.org/licenses/by/4.0/), which permits unrestricted use, distribution, and reproduction in any medium, provided the original work is properly cited.

**Introduction/Aim:** Sleep disturbance is often associated with pain persistence and is an important negative sequela that may occur post-surgically. In this prospective longitudinal study of women undergoing breast cancer surgery, we investigated sleep disturbance preoperatively and periodically up to one year. We assessed the relationship of demographic, medical, and psychological factors with sleep disturbance, and its association with acute and persistent postsurgical pain.

**Methods:** Women ages 18–80 (n = 259) underwent breast-conserving surgery, mastectomy, or mastectomy with reconstruction. PROMIS Sleep Disturbance short form captured extent of sleep disturbance preoperatively and at 2 weeks, 6 and 12 months after surgery. Pain severity, frequency, and physical, cognitive and emotional impact of pain, as well as psychosocial traits, and sensitivity to pain using a brief bedside quantitative sensory test were also longitudinally assessed.

**Results:** Sleep disturbance was variable amongst individual patients but stable over the course of the study, and moderately correlated with pain at all timepoints (Rho = 0.14–0.51,p’s<0.001). Greater sleep disturbance was also associated with younger age (Rho = −0.214,*p* = .001), lower physical activity (Rho = −.0170,*p* = .007), opioid use (Rho = 0.132, *p* = .039), catastrophizing (Rho = 0.241, *p* < .001), depression (Rho = 0.353,*p* < .001) anxiety (Rho = 0.414,*p* < .001), and lower pressure pain thresholds and tolerance on quantitative sensory testing (Rho = −0.208,*p* = .001;Rho-0.168,*p* = .009). Multivariate analysis revealed preoperative age (β = −0.098), baseline pain (β = 0.229), opioid use (β = 3.004), anxiety (β = 0.391), and positive affect (β = −0.142) as independent predictors of sleep disturbance.

**Discussion/Conclusions**: Sleep disturbance varies amongst individuals in the first year after surgery, and is associated with postsurgical pain, younger age, general pain sensitivity on QST, and psychosocial functioning.

## An Exploratory Investigation of Nociceptive Flexion Reflex in Women with Cerebral Palsy

Cole Hagen^a,*^, Elizabeth Boyer^a^, Rebekah Summers^b^, Lisa Lykken^a^, Chantel Barney^a,c^ and Frank Symons^c^

^a^Research, Gillette Children’s Specialty Healthcare, Saint Paul, Minnesota, USA; ^b^Department of Neurology, University of Minnesota, Minneapolis, Minnesota, USA; ^c^Department of Educational Psychology, University of Minnesota, Minneapolis, Minnesota, USA

**CONTACT** Cole Hagen colehagen@gillettechildrens.com

© 2020 The Author(s). Published with license by Taylor & Francis Group, LLC.

This is an Open Access article distributed under the terms of the Creative Commons Attribution License (http://creativecommons.org/licenses/by/4.0/), which permits unrestricted use, distribution, and reproduction in any medium, provided the original work is properly cited.

**Introduction/Aim**: Nociceptive flexion reflex (NFR) is utilized as a biomarker of spinal nociceptive processes. A contact heat evoked potential stimulator (CHEPS) was used to elicit spinal reflex responses from A-delta afferents. While underpowered, this exploratory work aimed to 1) establish the feasibility of testing the NFR using a CHEPS in cerebral palsy (CP), and 2) examine any differences between CP and controls to generate future hypotheses.

**Methods**: A CHEPS was used to apply 60 stimulus trials to the forearm and lower leg in high functioning women with CP (n = 10; M age = 20.66 years, SD = 2.36) compared to typically developing women (TD; n = 10; M age = 20.51 years, SD = 2.22). Monopolar surface electromyography (EMG) electrodes were placed on the biceps femoris and biceps brachii muscles. The rectified mean and peak EMG were obtained from 90–150 ms after stimulation to quantify the NFR. Z-scores were calculated relative to the 5–65 ms pre-stimulus data. Data were averaged across trials. Participants reported pain intensity using the numeric pain rating scale post-stimulus.

**Results**: There was no significant difference in mean (t(18) = 1.02, *p* = .32) or peak (t(18) = 1.68, *p* = .11) EMG Z-scores between groups. However there was a medium effect size for mean EMG (*d* = 0.45) and large effect size for peak EMG (*d* = 0.75) indicating reduced physiologic response in CP. There was no significant difference in pain ratings between groups (t(12.51) = 0.20, *p* = .84; *d* = 0.09).

**Discussion/Conclusions**: Initial findings suggest that subjective pain experiences from CHEPS are similar between women with CP and controls while physiologic responses may be attenuated in CP.

## Chemogenetic Silencing of TRPV1-expressing Colonic Afferents Reduces Visceral Hypersensitivity in Colitis

Manon Defaye^a,*^, Nasser Abdullah^a^, Mircea Iftinca^a^ and Christophe Altier^a^

^a^Department of Physiology and Pharmacology, University of Calgary, Calgary, Alberta, Canada

**CONTACT** Manon Defaye manon.defaye1@ucalgary.ca

© 2020 The Author(s). Published with license by Taylor & Francis Group, LLC.

This is an Open Access article distributed under the terms of the Creative Commons Attribution License (http://creativecommons.org/licenses/by/4.0/), which permits unrestricted use, distribution, and reproduction in any medium, provided the original work is properly cited.

**Introduction/Aim**: Abdominal pain is one of the main symptoms of Inflammatory Bowel Diseases (IBD) and often persists beyond clinical remission. Although we have a good understanding of the mechanisms of peripheral sensitization during colitis, little is known about the central sensitization. Recent work from our lab showed that G-CSF released from activated microglia was able to sensitize gut-innervating TRPV1+ colonic nociceptors that converge in the spinal cord and cause visceral sensitization. Here, we investigated the role of TRPV1+ visceral afferents in central sensitization during colitis.

**Methods**: To assess the role of TRPV1+ colonic afferents in central sensitization, we used a chemogenetic approach to inhibit the activity of TRPV1+ sensory neurons selectively. Six-week-old mice expressing the Gi-coupled DREADD (Designer Receptors Exclusively Activated by Designer Drugs) in TRPV1+ neurons were subjected to DSS treatment (2.5%, 7 days). One group was injected twice daily with the DREADD ligand Clozapine N-oxide (CNO) and visceromotor responses were evaluated by colorectal distension.

**Results**: The vast majority of TRPV1- and CGRP-labeled neurons expressed the HA-tagged Gi-DREADD in dorsal root ganglia (DRG) demonstrating efﬁcient cre-recombinase activity in small peptidergic neurons. *In vitro* experiment in cultured DRG neurons from Gi-DREADD mice revealed that CNO exposure induced a significant decrease in capsaicin-evoked currents. CNO exposure, *in vivo*, produced a decrease in DSS-induced visceral hypersensitivity compared with vehicle-injected mice.

**Discussion/Conclusions**: Overall, these data provided novel insights in our understanding of the mechanisms of chronic abdominal pain conditions and could be harnessed for developing effective therapeutic approaches to relieve pain in IBD.

## Retention of Prescription Opioids after Discharge following Hip and Knee Arthroplasty: A Clinical Audit

Kayla Denness http://orcid.org/0000-0002-2552-3268^a,*^, Eloise Carr http://orcid.org/0000-0003-1870-4244^b^ and Rosa Reyes^a^

^a^Department of Anesthesia, Alberta Health Services, South Health Campus, Calgary, Alberta, Canada; ^b^Faculty of Nursing, University of Calgary, Calgary, Alberta, Canada

**CONTACT** Kayla Denness kayla.denness@ahs.ca

© 2020 The Author(s). Published with license by Taylor & Francis Group, LLC.

This is an Open Access article distributed under the terms of the Creative Commons Attribution License (http://creativecommons.org/licenses/by/4.0/), which permits unrestricted use, distribution, and reproduction in any medium, provided the original work is properly cited.

**Introduction/Aim**: A projected increase in osteoarthritis prevalence will result in a correlated increase in total joint arthroplasties (TJA). In many settings, fast-track surgery and ERAS-style perioperative protocols contribute to shorter hospital stays. Early discharge after TJA tasks patients with self-managing postoperative pain that may be severe. Prescribed opioids are necessary to help patients stay on post-TJA physiotherapy pathways, increasing the likelihood of regaining quality of life previously lost to osteoarthritis. The clinical audit reported here begins to explore opioid oversupply and retention following discharge after TJA.

**Methods**: A cross-sectional survey was developed based on available evidence and clinical experience of the authors. Eight questions concerning post-discharge opioid prescriptions were administered by telephone to 20 participants four weeks after hip or knee TJA at a major hospital in western Canada.

**Results**: All participants (n = 20) filled an opioid prescription after discharge. Four weeks postoperatively, 15 (75%) were no longer taking daily opioids. Four patients took all of their prescribed opioid; 4 intended to bring unused opioid to a pharmacy for disposal; and 12 intended to keep unused opioids for future needs. Seventeen (85%) were satisfied with the number of pills they received.

**Discussion/Conclusions**: Prescribed opioids contribute to the risk of overdose and diversion in the community. In the absence of consensus guidelines for acute pain opioid prescribing, clinicians must be vigilant in educating patients about safe use, storage, and disposal of unused opioids. Multidisciplinary exploration and surveillance of post-discharge opioid management may identify systemic gaps and deficiencies in patient and provider education.

## Evaluating the Impact of Psychological and Sensitization Risk Factors on Chronic Pain-related Outcomes: A Cross-section Study

Zakir Uddin^a,*^, Arthur Woznowski-Vu^a^, Daniel Flegg^a^, Andrea Aternali^b^ and Timothy H. Wideman^a^

^a^School of Physical and Occupational Therapy, McGill University, Montreal, Quebec, Canada; ^b^Psychology, McGill University, Montreal, Quebec, Canada

**CONTACT** Zakir Uddin zakir.uddin@mail.mcgill.ca

© 2020 The Author(s). Published with license by Taylor & Francis Group, LLC.

This is an Open Access article distributed under the terms of the Creative Commons Attribution License (http://creativecommons.org/licenses/by/4.0/), which permits unrestricted use, distribution, and reproduction in any medium, provided the original work is properly cited.

**Introduction/Aim**: The risk constructs based on psychological risk factor (e.g. pain catastrophizing, PC) and sensitization risk factor (e.g. pressure pain threshold, PPT) are important in research and clinical practice. While most research looks at individual constructs, but doesn’t consider how different constructs might interact within the same individual. A cumulative impact evaluation of psychological and sensitization risk factors on pain-related outcomes may help guide us in the risk assessment of patients with pain conditions. The aim of this study is to evaluate the cumulative impact of these psychological PC and sensitization PPT risk factors on pain-related outcomes (activity avoidance, pain severity and disability) considering covariates.

**Methods**: We included 109 participants (70.60% women; mean ± SD age 53.6 ± 12.3 years) with chronic musculoskeletal pain for data analysis who completed all measures of this study.

Participants completed a single testing session that included measures of risk factors (PC and PPT) and pain-related outcomes (self-reported avoidance, functional avoidance, disability and pain severity). Subgroups were constructed by dichotomizing (median split) of PC and PPT scores, resulting in 4 groups: 1. low catastrophizing and low sensitivity (N = 26), 2. high catastrophizing and low sensitivity (N = 27), 3. low catastrophizing and high sensitivity (N = 25) and 4. high catastrophizing and high sensitivity (N = 31).

**Results**: One-way ANOVA revealed significant group differences (*p* < .05, η2 = .08 to.14) on all outcomes of this study (except functional avoidance) and post hoc analysis indicated the significance differences are between group 1 and 4 (*p* < .05). A cumulative impact is reflected by large effect sizes between group 1 and 4 (d = .8 to 1).

**Discussion/Conclusions**: The study suggests both higher level of pain catastrophizing and pressure sensitivity has a cumulative impact in risk screening for pain-related outcomes, considering gender in functional avoidance (task related outcome). This finding has important clinical and theoretical implications.

## Evaluating the Role of Positive and Negative Psychological Factors in Back Pain Rehabilitation: A Longitudinal Study

Zakir Uddin^a,*^, Arthur Woznowski-Vu^a^, Daniel Flegg^a^, Andrea Aternali^b^ and Timothy H. Wideman^a^

^a^School of Physical and Occupational Therapy, McGill University, Montreal, Quebec, Canada; ^b^Psychology, McGill University, Montreal, Quebec, Canada

**CONTACT** Zakir Uddin zakir.uddin@mail.mcgill.ca

© 2020 The Author(s). Published with license by Taylor & Francis Group, LLC.

This is an Open Access article distributed under the terms of the Creative Commons Attribution License (http://creativecommons.org/licenses/by/4.0/), which permits unrestricted use, distribution, and reproduction in any medium, provided the original work is properly cited.

**Introduction/Aim**: To predict 3-months rehabilitation outcomes of positive and negative psychological factors after controlling for significant covariates.

**Methods**: We included 81 adult participants with subacute nonspecific back pain for data analysis who completed all measures for (1) 3-months follow-up outcomes (i.e. pain severity, affective interference, physical interference and disability); (2) baseline positive (i.e. optimism, resilience and self-compassion) and negative (i.e. pain catastrophizing and pain-related fear) psychological factors. Four multiple regression analyses (dependent variables: pain severity, affective interference, physical interference and disability) were used to determine the predictive capability of positive and negative psychological factors while controlling for significant individual characteristics’ covariates.

**Results**: Pain catastrophizing predicting significantly only affective interference (β = .292, t = 2.294, *p* < .05) and disability (β = .252, t = 2.170, *p* < .05) outcomes. Other psychological factors are non-significant for predicting any of the 4 outcomes. Baseline pain intensity is a significant predictor for 3 outcomes (except affective interference), whereas baseline pain medication has failed to predict any outcome. Gender shows a significant predictor for physical interference outcome only (β = .199, t = 2.027, *p* < .05).

**Discussion/Conclusions**: Among negative psychological factors, only pain catastrophizing predicting 2 outcomes out of 4. None of the positive psychological factors can predict any outcome in his study. Baseline pain catastrophizing and pain intensity assessment may have a clinical benefit for better back pain rehabilitation outcomes considering gender.

## Do You Know How Painful that Was? The Role of Attachment in the Accuracy of Pain Perceptions among Romantic Dyads

Erika Gentile^a,*^, Hasagani Tissera^a^, Michael J.L. Sullivan^a^, Mathieu Roy http://orcid.org/0000-0002-3335-445X^a^ and John E. Lydon^a^

^a^Psychology, McGill University, Montreal, Quebec, Canada

**CONTACT** Erika Gentile erika.gentile@mail.mcgill.ca

© 2020 The Author(s). Published with license by Taylor & Francis Group, LLC.

This is an Open Access article distributed under the terms of the Creative Commons Attribution License (http://creativecommons.org/licenses/by/4.0/), which permits unrestricted use, distribution, and reproduction in any medium, provided the original work is properly cited.

Insecure attachment has been associated with negative pain outcomes. One contributing factor may be that insecure attachment styles influence pain perceptions, which is an important precursor for adequate support provision. To examine this, 109 romantic couples underwent a cold pressor task. Targets undergoing the task provided pain ratings while perceivers estimated partners’ pain. The perception of partners’ pain was conceptualized as *distinctive accuracy*, which assesses one’s ability to recognize targets’ unique pain experience. Overall, perceivers demonstrated distinctive accuracy. Further, perceivers’ and targets’ attachment interacted to predict distinctive accuracy. More secure targets, presumably displaying more expressive and relevant pain cues, were judged with greater distinctive accuracy but only by less secure perceivers. This may be because less secure perceivers are highly motivated to attend to their partner. Altogether, this study highlights one important individual difference factor that may help explain why attachment styles influence pain experiences.

**Introduction/Aim**: In comparison to a *secure* attachment style (perceptions that care is available and one is worthy of that care), an *insecure* attachment style (negative perceptions of the self as being worthy of care and/or perceptions of others as being unavailable to provide care) has been consistently linked with pain variables indicative of pain-related distress. Yet, little is known as to why this is. In general, receiving support from close others helps people better cope with pain and is an important precursor for adequate support provision is to perceive one’s pain experience accurately. If one does not understand the extent to which their partner is in pain, they may not be able to help them cope with that pain. The present study examines whether attachment styles influence pain perceptions, measured here as *distinctive accuracy* – being able to recognize targets’ unique pain experience. This may further our understanding of the negative pain outcomes associated with insecure attachment. Understanding the influence of attachment on one’s ability to cope and manage pain may aid in informing preventions, early interventions, and treatment efforts

**Methods**: Since romantic partners have a significant impact on one’s pain experience, this research focused on romantic couples. One hundred and nine romantic couples participated in this study and each member of the couple was randomly assigned to be either the *target*, the individual undergoing a 60-second cold pressor task, or the perceiver, the individual observing the target undergo the cold pressor task. Following the assignment of roles, participants completed demographic questionnaires assessing personal and relationship well-being as well as attachment style. During the cold pressure task, both the target and the perceiver were simultaneously prompted to provide ratings of the target’s pain using an 11-point scale at every 15-second interval. The average pain intensity ratings across the 4 time points were recorded.

**Results**: Results from the current study replicated previous work demonstrating that perceivers underestimate their partner’s pain and that insecure attachment is associated with more negative pain outcomes. Despite underestimating partner’s pain, perceivers viewed their partners, on average, with considerable distinctive accuracy. Moreover, the association between the target’s attachment style and distinctive accuracy was dependent on the perceiver’s level of security (*b = − 0*.25, *z = − 2*.04, *p* = .04). More secure target’s pain was perceived with greater distinctive accuracy by less secure perceivers (*b = *0.29, *z = *2.10, *p* = .04) but not by more secure perceivers (*b = − 0*.11, *z = *0.78, *p* = .44). Less secure targets were perceived with similar levels of distinctive accuracy by all perceivers (*b = *0.08, *z = *0.68, *p* = .50). Overall, the accuracy of pain perceptions depends on both the target’s and the perceiver’s attachment style.

**Discussion/Conclusions**: Our findings propose an explanation as to why less securely attached people may experience worse pain outcomes. By examining the differential effects of attachment for targets and perceivers, this research provides evidence to help understand the importance of attachment on pain outcomes.

## The Relationship between Perceived Phantom Limb Shortening (Telescoping) and Post-amputation Pain

Andrea Aternali http://orcid.org/0000-0002-8629-5086^a,*^, Sander L. Hitzig http://orcid.org/0000-0002-9139-9250^b^, Amanda Mayo http://orcid.org/0000-0001-7061-2529^b^ and Joel Katz http://orcid.org/0000-0002-8686-447X^a^

^a^Clinical Psychology, York University, Toronto, Ontario, Canada; ^b^Department of Psychology, York University, Toronto, Ontario, Canada

**CONTACT** Joel Katz jkatz@yorku.ca

© 2020 The Author(s). Published with license by Taylor & Francis Group, LLC.

This is an Open Access article distributed under the terms of the Creative Commons Attribution License (http://creativecommons.org/licenses/by/4.0/), which permits unrestricted use, distribution, and reproduction in any medium, provided the original work is properly cited.

**Introduction/Aim**: Approximately 80% of individuals who have undergone limb amputation report post-amputation pain. Despite ongoing efforts, there are few effective treatments for phantom and/or residual limb pain. The development of interventions for reducing post-amputation pain requires more knowledge about the relationship of phantom limb sensations on recovery. One phenomenon that occurs after amputation is phantom limb “telescoping”. Telescoping is defined as the experience of one’s phantom hand or foot gradually approaching the residual limb over time and is suggested to impact the experience of phantom pain. The current study explores the relationship between telescoping and post-amputation pain.

**Methods**: Fourteen adults living with an amputation for at least three months completed questionnaires requesting demographic information, average phantom and residual limb pain (0–10 numeric scale), and telescoping (yes/no) using an online application (https://phantomlimbs.ca/demo.html). Bivariate point biserial correlation coefficients evaluated the relationship between telescoping and demographic/pain-related information.

**Results**: A preliminary analysis of 14 limb loss respondents, 2 of whom reported telescoping, showed that telescoping was not correlated with age, sex, ethnicity, or time since amputation, but it was significantly correlated with level of education, *r*(12) = 0.849, *p* < .000. Telescoping was not significantly correlated with average phantom limb pain intensity, *r*(12) = − 0.204, *p* = .485, or residual limb pain intensity, *r*(12) = − 0.037, *p* = .899. Of the 3 participants who were pain-free, one reported telescoping.

**Discussion/Conclusions**: The results suggest that post-amputation pain intensity is not related to telescoping. A larger sample is needed for more definitive results.

## Non-pharmacological Pain Management of Neonatal and Older Infant Acute Pain

Oana Bucsea^a,*^, Ilana Shiff^a^, Rebecca Pillai Riddell^a^, Hannah Gennis^a^, Shaylea Badovinac^a^, Miranda DiLorenzo^a^, Nicole Racine^b^, Sara Ahola Kohut^c^, Diana Lisi^d^, Kara Turcotte^d^, Bonnie Stevens^e^ and Lindsay Uman^f^

^a^Psychology, York University, Toronto, Ontario, Canada; ^b^Psychology, University of Calgary, Calgary, Alberta, Canada; ^c^Gastroenterology, Hepatology and Nutrition, Hospital for Sick Children, Toronto, Ontario, Canada; ^d^Psychology, University of British Columbia | Okanagan, Kelowna, British Columbia, Canada; ^e^Nursing, University of Toronto, Toronto, Ontario, Canada; ^f^Complex Pain Team, IWK Health Centre, Halifax, Nova Scotia, Canada

**CONTACT** Oana Bucsea obucsea@yorku.ca

© 2020 The Author(s). Published with license by Taylor & Francis Group, LLC.

This is an Open Access article distributed under the terms of the Creative Commons Attribution License (http://creativecommons.org/licenses/by/4.0/), which permits unrestricted use, distribution, and reproduction in any medium, provided the original work is properly cited.

**Introduction/Aim**: The objective of the current review is to assess the efficacy of non-pharmacological interventions (excluding kangaroo care, music, and breastfeeding) for managing acute pain in full-term neonates and older infants.

**Methods**: Analyzes were run separately for pain reactivity (within first 30 seconds post-stimulus) and immediate pain regulation (> 30 seconds post-stimulus) for 15 different non-pharmacological strategies. Each intervention category was based on at least two studies.

**Results**: Based on 31 studies examining neonatal pain responses, swaddling (SMD = − 1.02, 95%CI [−1.43, −0.62]) and non-nutritive sucking (SMD = − 1.20, 95%CI [−2.01, −0.38]) were found to be most efficacious at reducing pain reactivity, while non-nutritive sucking (SMD = − 1.19, 95%CI [−1.85, −0.52]) was also found to most improve immediate pain regulation. Based on 27 studies examining older infant pain responses, touch-related interventions were found to be most efficacious at both reducing pain reactivity (SMD = − 1.51, 95%CI [−2.17, −0.85]) and improving immediate pain regulation (SMD = − 1.37, 95%CI [−2.40, −0.34]). Interestingly, non-pharmacological interventions had an additive effect on sucrose for reducing pain reactivity (SMD = − 1.28, 95%CI [−2.47, −0.09]) and improving immediate pain regulation (SMD = − 0.56, 95%CI [−0.97, −0.14]) in neonates, but not older infants.

**Discussion/Conclusions**: This review seeks to inform the implementation of evidence-based interventions for managing acute pain in neonates and older infants.

## The Role of Rehabilitation in Opioid Tapering: A Scoping Review

Miranda Wiens^a,*^, Devon Jarrett^b^, Alissa Settimi^b^, Courtney White^b^, Zachary Hollingham^b^ and Tara Packham http://orcid.org/0000-0002-5593-1975^b^

^a^Hamilton Health Sciences, Acute Medicine, Hamilton, Ontario, Canada; ^b^School of Rehabilitation Science, McMaster University, Hamilton, Ontario, Canada

**CONTACT** Miranda Wiens wiensm@hhsc.ca

© 2020 The Author(s). Published with license by Taylor & Francis Group, LLC.

This is an Open Access article distributed under the terms of the Creative Commons Attribution License (http://creativecommons.org/licenses/by/4.0/), which permits unrestricted use, distribution, and reproduction in any medium, provided the original work is properly cited.

**Introduction/Aim**: One in five Canadians are affected by chronic non-cancer pain, with Canada having the highest rate of opioid prescribing in the world. Recent Canadian prescribing guidelines suggest non-pharmacological pain management approaches, like those provided by physiotherapists and occupational therapists, should be used in opioid tapering. We conducted a scoping review to locate and summarize the current evidence regarding the roles of physiotherapy and occupational therapy in opioid tapering for individuals with chronic non-cancer pain.

**Methods**: A systematic search of Medline, EMBASE, PubMed, and CINAHL databases was conducted. Articles were included if physiotherapy or occupational therapy were described or were part of an interdisciplinary team and if opioid tapering or reduction were mentioned or measured as an outcome.

**Results**: 22 articles were included – one systematic review, four narrative reviews, two case-reports, six retrospective studies, one cross-sectional study, one randomized controlled trial, one prospective longitudinal cohort, one evidence-based perspective, one program description, and four conference abstracts. Papers were categorized into 5 themes: multidisciplinary team care, exercise focused, single modalities, patient preferences, and patient experiences.

**Discussion/Conclusions**: The findings suggest there is currently limited evidence to guide PTs and OTs in their role in opioid tapering for patients with chronic non-cancer pain. It appears the general use of physiotherapy and occupational therapy interventions are helpful in supporting the opioid tapering process, however, further research is needed to establish effectiveness.

## The Pathway to Pain-Free: Pain Experiences in Adolescent Recipients of Hematopoietic Stem Cell Transplant to Cure Sickle Cell Disease

Caitlin Forbes^a,*^, Courtney Charnock^a^, Gregory M. T. Guilcher^b^, Melanie Noel http://orcid.org/0000-0003-3752-8055^c^ and Fiona Schulte^a^

^a^Cumming School of Medicine, University of Calgary, Oncology, Calgary, Alberta, Canada; ^b^Departments of Oncology and Pediatrics, Cumming School of Medicine, University of Calgary, Calgary, Alberta, Canada; ^c^Psychology, University of Calgary, Calgary, Alberta, Canada

**CONTACT** Caitlin Forbes caitlin.forbes@ucalgary.ca

© 2020 The Author(s). Published with license by Taylor & Francis Group, LLC.

This is an Open Access article distributed under the terms of the Creative Commons Attribution License (http://creativecommons.org/licenses/by/4.0/), which permits unrestricted use, distribution, and reproduction in any medium, provided the original work is properly cited.

**Introduction/Aim**: Sickle cell disease (SCD) is a chronic disease affecting over 5000 individuals in Canada. Acute and chronic pain is commonly experienced and may require hospitalization, medication, and blood transfusions. Hematopoietic stem cell transplant (HSCT) is the only established cure for SCD however, up to 40% of adult HSCT recipients continue to experience pain. Pain in adolescents and young adults (AYAs) following HSCT in childhood has not been investigated. This study aims to explore the pain experience of AYAs who received HSCT to cure SCD.

**Methods**: Four AYA recipients of HSCT (mean age = 19.75 years [*SD *= 3.30], 25% male) and their parents (25% fathers) completed separate, semi-structured interviews. Participants were asked to describe their SCD experience at multiple time points (i.e., prior to HSCT, during HSCT, following HSCT). Interviews were audio-recorded, transcribed verbatim, and coded by two independent coders using an inductive approach.

**Results**: Recruitment for the study is ongoing. Parents and AYAs described significant interference in their lives due to SCD and its treatment (e.g., missing school, work or social activities). Families describe HSCT as a life changing event that completely resolved SCD pain. Cultural stigma toward SCD influenced access to social support in all parent narratives.

**Discussion/Conclusions**: SCD is a painful chronic disease that severely impacts the lives of patients and their families. Enduring stigma surrounding the diagnosis impacts families’ ability to access social support. HSCT allowed adolescents to live without pain and pursue social and educational goals. Future research is required to address stigma and bolster social support among families living with SCD.

## Updating the Pediatric Fear-avoidance Model of Chronic Pain: The Transdiagnostic Role of Parent and Youth Intolerance of Uncertainty

Alexandra Neville^a,*^, Daniel Kopala-Sibley^b^, Sabine Soltani^a^, Gordon J. G. Asmundson^c^ and Melanie Noel http://orcid.org/0000-0003-3752-8055^a^

^a^Psychology Department, University of Calgary, Calgary, Alberta, Canada; ^b^Department of Psychiatry, University of Calgary, Calgary, Alberta, Canada; ^c^Department of Psychology, University of Regina, Regina, Saskatchewan, Canada

**CONTACT** Alexandra Neville alexandra.neville@ucalgary.ca; @neville_alex

© 2020 The Author(s). Published with license by Taylor & Francis Group, LLC.

This is an Open Access article distributed under the terms of the Creative Commons Attribution License (http://creativecommons.org/licenses/by/4.0/), which permits unrestricted use, distribution, and reproduction in any medium, provided the original work is properly cited.

**Introduction/Aim**: The experience of pediatric chronic pain is fraught with uncertainty. Individuals who are high in intolerance of uncertainty (IU), which is conceived as a transdiagnostic risk factor for fear and anxiety-related psychopathology, tend to interpret ambiguous situations as more threatening than those who are low in IU. The pediatric fear-avoidance model illustrates the bidirectional relationships between parent and youth psychological responses (e.g., catastrophizing) influencing youth fear of pain and pain disability. This study investigated the role of parent and youth IU as predisposing risk factors in the pediatric fear-avoidance model of chronic pain.

**Methods**: Participants included 138 youth with chronic pain (*M_age_ *= 14.29 years; 74% female) and their parents (93% female). At baseline, parents and youth reported on their IU and catastrophic thinking about youth pain; youth reported on their fear of pain and pain interference. Three months later, youth and parents reported on their IU; youth reported on their pain interference.

**Results**: Structural equation modeling supported the prediction that higher baseline youth IU directly exacerbates youth fear of pain, which in turn predicts worse pain interference at follow-up. IU indirectly predicted worse youth fear of pain through its influence on youth pain catastrophizing. Further, baseline youth IU predicted increased IU at follow-up. Parent IU predicted increased parent pain catastrophizing and youth IU.

**Discussion/Conclusions**: These findings suggest that parent and youth IU play important roles as predisposing risk factors in the pediatric fear-avoidance model of chronic pain. Future research is needed to determine how to target these psychological factors in treatment.

## Meaningfulness in Chronic Pain Rehabilitation: A Concept Analysis

Katrina Liddiard http://orcid.org/0000-0003-3826-2631^a,*^, Annette Raynor http://orcid.org/0000-0002-6517-3872^a^ and Cary Brown http://orcid.org/0000-0002-5282-4170^b^

^a^School of Medical & Health Science, Edith Cowan University, Joondalup, Western Australia, Australia; ^b^Department of Occupational Therapy, Faculty of Rehabilitation Medicine, University of Alberta, Edmonton, Alberta, Canada

**CONTACT** Katrina Liddiard k.liddiard@ecu.edu.au

© 2020 The Author(s). Published with license by Taylor & Francis Group, LLC.

This is an Open Access article distributed under the terms of the Creative Commons Attribution License (http://creativecommons.org/licenses/by/4.0/), which permits unrestricted use, distribution, and reproduction in any medium, provided the original work is properly cited.

**Introduction/Aim**: Chronic pain represents a considerable burden to the individual and society, and often disrupts meaningful aspects of life, however, clinicians use the term “meaningful” in various ways. This inconsistency may contribute to a lack of consensus on how to make rehabilitation personally meaningful for people with chronic pain. The aim of this study was to identify the structure, and function, of the concept “meaningfulness” as it is currently used in peer-reviewed, chronic pain rehabilitation literature; and to develop an operational definition of the concept “meaningfulness” for use in a larger, mixed methods study into meaningfulness in rehabilitation for people with chronic pain.

**Methods**: This research applied Walker and Avant’s Concept Analysis method. A systematic search of chronic pain rehabilitation literature was conducted through databases CINAHL, MEDLINE and PsycINFO, along with citation searching and hand searching. All uses of the concept “meaningful”, “meaningfulness” and “personally meaningful” were identified.

**Results**: After exclusion criteria were applied, 113 articles remained, however, only ten of these used “meaningful” as perceived by the person with chronic pain. From the findings, an operational definition of patient-defined meaningfulness was developed.

**Discussion/Conclusions**: The concept “meaningfulness” was used inconsistently, which suggests that rehabilitation clinicians and patients may not identify the same things to be meaningful. The definition derived from the concept analysis is: Patient-identified meaningfulness describes that which patients themselves select as being of value, and relates to their personal sense of identity. With this definition, further rigorous research will be conducted into the benefits of personally meaningful chronic pain rehabilitation.

## Early Life Trauma and Alterations to Brain Structure Underlying the Chronification of Headaches in Youth

Jillian Vinall Miller^a,*^, Quinn Andre^b^, Inge Timmers^c^, Laura Simons http://orcid.org/0000-0002-3395-9483^c^, Nivez Rasic^a^ and Catherine Lebel^d,*^, Melanie Noel http://orcid.org/0000-0003-3752-8055^e,*^

^a^Anesthesiology, Perioperative and Pain Medicine, University of Calgary, Calgary, Alberta, Canada; ^b^Medicine, University of Alberta, Edmonton, Alberta, Canada; ^c^Anesthesiology, Perioperative and Pain Medicine, Stanford University, Stanford, California, USA; ^d^Radiology, University of Calgary, Calgary, Alberta, Canada; ^e^Psychology, University of Calgary, Calgary, Alberta, Canada

**CONTACT** Jillian Vinall Miller jillian.miller1@ucalgary.ca

*Co-senior authors

© 2020 The Author(s). Published with license by Taylor & Francis Group, LLC.

This is an Open Access article distributed under the terms of the Creative Commons Attribution License (http://creativecommons.org/licenses/by/4.0/), which permits unrestricted use, distribution, and reproduction in any medium, provided the original work is properly cited.

**Introduction/Aim**: Chronic headaches are highly prevalent in youth, and often persist into adulthood. The present study explored the extent that alterations to brain structures involved in emotional processing (i.e. amygdala and associated connections [i.e. the uncinate fasciculus, which connects parts of the limbic system e.g. the amygdala with the frontal lobe]), as well as behavioral changes (i.e. posttraumatic stress symptoms [PTSS] and poor sleep quality) resulting from early life trauma were associated with persistent headaches in youth.

**Methods**: Thirty youth aged 10–18 years with chronic headaches underwent a 3T MRI scan. Left and right amygdala volumes were acquired and summed. Mean fractional anisotropy (FA) values of the left and right uncinate fasciculus were acquired and averaged. Youth tracked their daily headaches for one-month, and self-reported on their pubertal status, sleep quality and PTSS using validated measures. Linear regression was used to explore the relationships between average number of headaches, brain and behavior measures.

**Results**: Smaller amygdala volumes (*ß *= −0.64, *P *= .04), lower fractional anisotropy of the uncinate fasciculus (*ß *= −0.53, *P *= .02) and greater PTSS (*ß *= 0.73, *P *= .002) were associated with greater number of headaches per month in youth, after accounting for pubertal status, total subcortical gray matter volume and sleep quality (Adjusted R^2^ = 0.44).

**Discussion/Conclusions**: Alterations to regions of the brain involved in emotional processing and PTSS appears to underlie the chronification of headaches in youth. By addressing PTSS, clinicians may be able to reduce the frequency of headaches in patients, leading to associated brain changes and thereby preventing the persistence of pain into adulthood.

## Trigeminal Neuralgia and Radiologically Isolated Syndrome: A Case Series

Sarasa Tohyama^a,*^, Makenna Timm^b^, Aisha Halawani^b^, David J. Mikulis^c^ and Mojgan Hodaie^b^

^a^Institute of Medical Science, University of Toronto, Toronto, Ontario, Canada; ^b^Division of Brain, Imaging, and Behaviour, Systems Neuroscience, Krembil Brain Institute, Toronto, Ontario, Canada; ^c^Division of Neuroradiology, Joint Department of Medical Imaging, Toronto Western Hospital, Toronto, Ontario, Canada

**CONTACT** Sarasa Tohyama sarasa.tohyama@mail.utoronto.ca

© 2020 The Author(s). Published with license by Taylor & Francis Group, LLC.

This is an Open Access article distributed under the terms of the Creative Commons Attribution License (http://creativecommons.org/licenses/by/4.0/), which permits unrestricted use, distribution, and reproduction in any medium, provided the original work is properly cited.

**Introduction/Aim**: Trigeminal neuralgia (TN) is a chronic neuropathic facial pain disorder distinguished by recurrent attacks of severe, electrical pain. TN can be a symptom of multiple sclerosis (MS), where multiple lesions in the brain and spinal cord are often observed on conventional MRI. Here, we report a new group of TN patients that present with MRI findings strongly suggestive of MS without clinical manifestation of the disease, termed radiologically isolated syndrome (RIS).

**Methods**: We identified 4 patients (1 male and 3 females, mean age ± SD: 55.8 ± 15.5 years) via retrospective review of MRI and clinical records. The diagnostic criteria for TN and MS were rereviewed according to the latest International Classification of Headache Disorders (ICHD-3) and McDonald criteria for MS. T1- and T2-weighted MR images were examined to determine lesion characteristics. Clinical follow-up data were examined to assess treatment response.

**Results**: All 4 patients fulfilled criteria for the diagnosis of TN. While MRI findings were highly suggestive of MS, patients did not meet diagnostic criteria for MS, and were identified as having RIS. TN pain was not sufficiently controlled with medical treatment and all patients elected to undergo surgery.

**Discussion/Conclusions**: This report is the first description of TN associated with RIS. These patients present with TN and white matter lesions suggestive of MS, with an otherwise normal neurological examination. These findings point to the role of central demyelination as a potential pathogenesis of TN pain.

## A Preliminary Evaluation of Falls Risk for Indiviuals with Chronic Pain Attending an Interdisciplinary Pain Self-management Group

Andree Roy^a,*^ and Douglas Cane^a^

^a^Pain Management Unit, Nova Scotia Health Authority, Halifax, Nova Scotia, Canada

**CONTACT** Andree Roy Andree.roy@nshealth.ca

© 2020 The Author(s). Published with license by Taylor & Francis Group, LLC.

This is an Open Access article distributed under the terms of the Creative Commons Attribution License (http://creativecommons.org/licenses/by/4.0/), which permits unrestricted use, distribution, and reproduction in any medium, provided the original work is properly cited.

**Introduction/Aim**: Fear of movement associated with chronic pain often leads to deconditioning, postural compromises and subsequent risk of falls. Compared to the general population, individuals with chronic pain are at greater risk of falling. This study evaluated several measures used to assess falls risk in the elderly. These included Self-selected Walking speed (SSWS), Timed Up and Go (TUG), Sit-to-Stand (STS), and the Ability-Specific Balance Confidence Questionnaire (ABC). Measures were obtained at the start of a pain management group. Individuals with chronic pain were compared to existing norms, and relationships between measures were assessed.

**Methods**: Prior to treatment, 216 individuals with ongoing pain completed measures of pain intensity, the ABC, Self-selected Walking Speed, Timed Up and Go, and the Sit to Stand. Norms were determined based on sex and age. Correlations were calculated to examine the relationships among these measures.

**Results**: Overall, the performance of individuals with ongoing pain on the physical measures was similar to the elderly. Physical performance did not differ for men and women, was unrelated to age, and was negatively related to pain intensity. Scores on the ABC did not differ by sex, were unrelated to age, and negatively related to pain intensity. Poorer physical performance significantly correlated with decreased confidence with respect to balance.

**Discussion/Conclusions**: Ongoing pain is associated with increased risk of falling. This risk does not differ for men and women and is independent of age. Greater pain intensity is related to poorer physical performance and decreased confidence for balance. Risk of falling should be assessed for all individuals with ongoing pain regardless of age.

## Pediatric Pain: A New Frontier for Using Virtual Patient Technology to Assess Provider Decision Making

Megan M. Miller http://orcid.org/0000-0002-6222-2659^a,*^ and Adam T. Hirsh^a^

^a^Department of Psychology, Indiana University-Purdue University Indianapolis, Indianapolis, Indiana, USA

**CONTACT** Megan M. Miller mmm24@iupui.edu

© 2020 The Author(s). Published with license by Taylor & Francis Group, LLC.

This is an Open Access article distributed under the terms of the Creative Commons Attribution License (http://creativecommons.org/licenses/by/4.0/), which permits unrestricted use, distribution, and reproduction in any medium, provided the original work is properly cited.

**Introduction/Aim**: Virtual Patient (VP) technology has been used to elucidate disparities in adult pain care. A strength of VP technology is the ability to standardize/manipulate aspects of the clinical encounter – such as patient demographics (eg, race, gender), behaviors (eg, guarding, bracing), and expressions (ie, facial displays, vocalizations) – to test hypotheses about providers’ pain care decisions. Although disparities in pain care for youth have garnered recent attention, our understanding is limited by the lack of experimental investigations on this topic. VP technology is well-suited to fill this gap.

**Methods**: Virtual pediatric patients (VPPs) were developed using Autodesk Character Generator and Adobe Fuse. Initial VPPs were modeled on functional abdominal pain such that all VPPs demonstrate condition-specific, dynamic pain expressions (ie, grimacing, holding stomach). Several iterations were developed with a focus on patient (eg, race, gender, age) and caregiver factors (eg, behavior toward child), allowing systematic investigation of their impact on providers’ pain care decisions. In our initial study, VPPs varied on gender and race (ie, Black/White), with other characteristics standardized across VPPs.

**Results**: Providers (N = 129 [sample 1], 43 [sample 2]) made pain care decisions for 4 VPPs (one of each race/gender). Systematic differences across patient race/gender were identified. Moreover, results suggest that providers engaged with VPPs similarly to real patients, and responses were not substantially biased by social desirability.

**Discussion/Conclusions**: Identifying the individual and contextual factors that affect provider decision-making is an essential first step to reducing disparities in pediatric pain care. VP technology offers unique advantages to achieving this goal.

## Use of Nonpharmacological Treatments for Chronic Pain Management: A Sex- and Gender-based Analysis

Anaïs Lacasse http://orcid.org/0000-0002-3992-5145^a,*^, Véronique Gagnon^a^, Gabrielle Pagé http://orcid.org/0000-0002-7742-2717^b^, Line Guénette http://orcid.org/0000-0001-9769-7550^c^ and Lucie Blais^d^

^a^Département des Sciences de la Santé, Université du Québec en Abitibi-Témiscamingue (UQAT), Rouyn-Noranda, Québec, Canada; ^b^Département d’anesthésiologie et de Médecine de la Douleur, Faculté de Médecine, Centre de Recherche du Centre Hospitalier de l’Université de Montréal (CRCHUM), Université de Montréal, Montréal, Québec, Canada; ^c^Faculté de Pharmacie, Université Laval, Québec, Québec, Canada; ^d^Faculté de Pharmacie, Université de Montréal, Montréal, Québec, Canada

**CONTACT** Anaïs Lacasse anais.lacasse@uqat.ca

© 2020 The Author(s). Published with license by Taylor & Francis Group, LLC.

This is an Open Access article distributed under the terms of the Creative Commons Attribution License (http://creativecommons.org/licenses/by/4.0/), which permits unrestricted use, distribution, and reproduction in any medium, provided the original work is properly cited.

**Introduction/Aim**: Although many studies identified sex as a predictor of pain treatments utilization (women being more likely), the association between the use of nonpharmacological modalities and gender is less clear. This study aimed to explore the association between sex, gender and the use of nonpharmacological treatments for chronic pain (CP).

**Methods**: In 2019, a province-wide web-based cross-sectional study was conducted among adults suffering from CP (Quebec, Canada). Sex was measured as a dichotomous variable and gender roles (behavioral norms applied to men/women) were measured using the Bem Sex-Role Inventory (BSRI). The median split method was applied to masculine/feminine BSRI scores to form subgroups. Multivariate logistic regression allowed the identification of variables associated to nonpharmacological treatments use.

**Results**: 1372 participants completed the questionnaire about sex/gender (women:84.0%, mean age:49.4 ± 13.1) and a majority reported using nonpharmacological treatments for their pain (82.8%). Bivariate comparisons revealed that the prevalence of use varied across sex (women: 83.9%, men: 77.1%; *p* = .0165) and gender roles subgroups (feminine: 77.1%, masculine: 87.7%, androgynous: 86.7%, undifferentiated: 79.4%; *p* = .0003). Controlling for pain frequency, duration, intensity, catastrophizing, age, use of pain medications, work status, and education level, gender (but not sex) remained associated to nonpharmacological treatments use (Masculine vs Feminine OR: 1.781 95%CI: 1.083–2.927; Androgynous vs Feminine OR: 1.798, 95%CI: 1.175–2.752).

**Discussion/Conclusions**: Gender roles differences were found regarding the use of nonpharmacological treatments and may suggest a need for more personalized promotion of such treatment modalities. Further studies should explore if gender roles affect treatment preferences or are associated with barriers to treatment.

## Parent Surgical History: A Root of Children’s Post-Surgical Pain?

Cara Nania^a,*^ and Melanie Noel http://orcid.org/0000-0003-3752-8055^b^

^a^Department of Psychology, University of Calgary, Calgary, Alberta, Canada; ^b^Department of Psychology, University of Calgary, Alberta Children’s Hospital Research Institute (Behaviour and the Developing Brain Theme), Hotchkiss Brain Institute, Mathison Centre for Mental Health Research and Education, Calgary, Alberta, Canada

**CONTACT** Cara Nania cgnania@ucalgary.ca

© 2020 The Author(s). Published with license by Taylor & Francis Group, LLC.

This is an Open Access article distributed under the terms of the Creative Commons Attribution License (http://creativecommons.org/licenses/by/4.0/), which permits unrestricted use, distribution, and reproduction in any medium, provided the original work is properly cited.

**Introduction/Aim**: How children remember pain sets the stage for future pain experiences. Parents with higher trait anxiety prior to their child’s surgery have children who develop a negatively-biased recall of their post-surgical pain. Further, parental catastrophizing about child pain prior to surgery predicts greater pain memory biases, which is linked to future pain problems and fear-avoidance. Little is known about *why* parents demonstrate these behaviors. The present study examined the impact of parental surgical history on their catastrophic thinking about child pain, as well as children’s post-surgical pain and fear.

**Methods**: The sample included 85 children (aged 4–7) scheduled to undergo a tonsillectomy and one of their parents. Prior to surgery, parents reported on their past surgical experiences and completed a measure of catastrophic thinking about child pain. Children’s post-operative pain intensity and pain-related fear were reported on days 1–3 following surgery.

**Results**: Parents who reported being more afraid of their own post-surgical pain catastrophized more about their child’s pain (*p* =.03). Parental catastrophic thinking about child pain was also related to worse child pain intensity and fear post-surgery (*ps* <.05).

**Discussion/Conclusions**: These findings extend existing research by identifying potential factors that impact parental pre-operative anxiety and pain catastrophizing. Parents who recall being afraid of their own post-surgical pain also catastrophize about their children’s pain, putting their children at risk for worse pain outcomes after surgery. These findings underscore the importance of parental factors in the surgical experience of children and provide a target for pain management interventions.

## The Social Context of Parent and Child Pain: A Gender Analysis

Shanaya Fischer^a,*^, Jaimie Beveridge^a^ and Melanie Noel http://orcid.org/0000-0003-3752-8055^b^

^a^Department of Psychology, University of Calgary, Calgary, Alberta, Canada; ^b^Department of Psychology, University of Calgary, Alberta Children’s Hospital Research Institute (Behaviour and the Developing Brain Theme), Hotchkiss Brain Institute, Mathison Centre for Mental Health Research and Education, Calgary, Alberta, Canada

**CONTACT** Shanaya Fischer sdfische@ucalgary.ca

© 2020 The Author(s). Published with license by Taylor & Francis Group, LLC.

This is an Open Access article distributed under the terms of the Creative Commons Attribution License (http://creativecommons.org/licenses/by/4.0/), which permits unrestricted use, distribution, and reproduction in any medium, provided the original work is properly cited.

**Introduction/Aim**: Chronic pain (pain lasting > 3 months) afflicts 15-40% of youth. Youth with chronic pain experience greater peer victimization and report having less friends. Furthermore, among youth in this population, it is estimated that approximately 50% have a parent with chronic pain. Little research to date has examined the relationships between pain and social functioning in parents and youth. The present study examined the relationships between these variables.

**Methods**: 95 youth aged 8–18 and one of their parents were recruited from a tertiary-level pediatric chronic pain program at a children’s hospital in Western Canada. The dyad completed a questionnaire at intake and again 3 months later. Parent and youth rated their pain intensity on an 11 point NRS. Parents completed the PROMIS Ability to Participate in Social Roles subscale. Youth completed the PROMIS Peer Relationships Subscale.

**Results**: Parent and child pain had no significant affect on parent social functioning at 3 months. Parent and child pain were significantly associated with youth social functioning at 3 months. This relationship differed by gender. The influence of parent pain on youth social functioning was significant for girls (*ΔR*^2^ = .03, *ΔF *= 4.22, *p* <.05) but not boys, whereas the impact of youth pain on youth social functioning was significant for boys (*ΔR*^2^ = .12, *ΔF *= 5.49, *p* < .05) but not girls.

**Discussion/Conclusions**: This study adds to a growing body of literature surrounding the importance of parental experience of pain on youth functioning. The results suggest that girls may be especially impacted by their parents’ experience of pain.

## Integrated Psychosocial Group Treatment (IPGT): A Harm Reduction and Preventative Approach for Chronic Pain Patients at Risk for Opioid Misuse

Valerie Hruschak^a,*^, Daniel Rosen^b^, Megan Tierney^b^, Shaun Eack^b^, Ajay D. Wasan^c^ and Gerald Cochran^d^

^a^Department of Anesthesiology, Perioperative and Pain Medicine at Harvard Medical School, Brigham and Women’s Hospital, Boston, Massachusetts, USA; ^b^School of Social Work, University of Pittsburgh, Pittsburgh, Pennsylvania, USA; ^c^Department of Anesthesiology and Perioperative Medicine and Psychiatry, University of Pittsburgh/UPMC Pain Medicine, Pittsburgh, Pennsylvania, USA; ^d^Internal Medicine, Epidemiology,University of Utah,Salt Lake City, Utah, USA

**CONTACT** Valerie Hruschak Valerie.Hruschak@gmail.com

© 2020 The Author(s). Published with license by Taylor & Francis Group, LLC.

This is an Open Access article distributed under the terms of the Creative Commons Attribution License (http://creativecommons.org/licenses/by/4.0/), which permits unrestricted use, distribution, and reproduction in any medium, provided the original work is properly cited.

**Introduction/Aim**: North America is experiencing an interrelated public health crisis, involving the management of chronic pain and the risks associated with opioid misuse. A fundamental challenge is to achieve a balance between decreasing the misuse of opioids and associated harms while optimizing pain care.

**Methods**: Chronic pain patients at risk for opioid misuse (n = 30) were randomly assigned to Integrated Psychosocial Group Treatment (IPGT) or treatment as usual (TAU). IPGT consists of 6 group sessions involving psychoeducation, motivational interviewing, cognitive behavioral therapy, mindfulness, and peer support. Participants were assessed at baseline, 6 weeks, and 9 weeks. Outcomes were assessed using self-reported measures and included: (1) feasibility; (2) acceptability; and (3) pain severity, pain interference, pain catastrophizing, and opioid misuse. Data were analyzed using descriptive and multivariate analyzes.

**Results**: All intervention components were delivered to 87% (n = 13) of IPGT recipients who reported a high level of satisfaction. Findings suggested that the IPGT group experienced nonsignificant improvements in pain severity compared to the TAU control group (β = 0.22, *p* = .35). However, we observed significant treatment by time interactions on the outcome of pain interference (β = 3.32, *p* = .05) and pain catastrophizing (β = 2.74, *p* = .02). We detected no significant differences in opioid misuse (AOR = 069, *p* = .16).

**Discussion/Conclusions**: This study provides support for IPGT being acceptable and feasible for delivery in chronic pain patients at risk for opioid misuse in which efficacy was demonstrated in pain interference and catastrophizing. Future studies should expand on these findings by investigating IPGT within a fully powered framework.

## Comparative Efficacy of Pharmacologic Interventions for the Prevention of Chronic Postsurgical Pain. A Systematic Review and Network Meta-analysis

Claire Allen^a,*^, Andrew M. Walker^a^, Zahra A. Premji^b^, Marie-Eve Beauchemin-Turcotte^a^, Jenny Wong^a^, Sonya Soh^a^, Geoffrey Hawboldt^a^, Kelly Shinkaruk^a^ and David Archer^a^

^a^Cumming School of Medicine, University of Calgary, Calgary, Alberta, Canada; ^b^Haskayne School of Business, Libraries and Cultural Resources, University of Calgary, Calgary, Alberta, Canada

**CONTACT** Claire Allen claire.allen@ahs.ca

© 2020 The Author(s). Published with license by Taylor & Francis Group, LLC.

This is an Open Access article distributed under the terms of the Creative Commons Attribution License (http://creativecommons.org/licenses/by/4.0/), which permits unrestricted use, distribution, and reproduction in any medium, provided the original work is properly cited.

**Introduction/Aim**: Optimal perioperative pain management is an important public health goal with 10% to 50% of patients reporting chronic postsurgical pain (CPSP). With few head-to-head trials of CPSP preventive strategies, ranking is difficult. Through a systematic review, pairwise and network meta-analyses (NMA) we aimed to rank interventions for efficacy and adverse effects. Our goals are to inform the selection of preventative measures in perioperative practice and guide future investigations.

**Methods**: We searched Cochrane Central Registry of Controlled Trials, MEDLINE, Embase, ClinicalTrials.gov, and WHO ICTRP for double-blind, randomized controlled trials of CPSP prevention in adults. We assessed risk of bias and confidence level in the evidence. Using meta-regression, we evaluated potential effect modifiers. Primary outcomes were treatment effect and potential harm. We used group-level data and estimated risk ratio with random effects.

**Results**: We included 102 studies with 13,416 participants. CPSP incidence in placebo patients varied according to IASP coding. In high-risk patients (IASP codes 1–4), effective interventions were anti–inflammatories+neural block (NNT = 4), SNRI (NNT = 3), and systemic local anesthetic (NNT = 7). For low-risk patients (IASP codes 5–8), effective interventions were alpha-2 receptor agonists (NNT = 6), gabapentanoids + NMDA receptor blocker (NNT = 5), and neural block (NNT = 15). The highest ranked interventions were alpha-2-agonists, systemic local anesthetics, anti–inflammatories+neural block, SNRI, NMDA receptor blocker+neural block, and neural block.

**Discussion/Conclusions**: Interventions with “acute post-operative pain benefits” had the greatest effect in reducing CPSP risk. Our ranking suggests that “de-afferentation” with neural block may be important. A preplanned living NMA comparing top-ranking interventions may address under-powered studies with low precision in the estimates.

## Moderators of Mindfulness Therapy Vs Cognitive Behavioural Therapy for the Treatment of Provoked Vestibulodynia, a Chronic Genital Pain Disorder

Lauren Rietchel^a,*^, Bozena Zdaniuk^a^, Sophie Bergeron http://orcid.org/0000-0001-8601-761X^b^ and Lori A. Brotto^a^

^a^Obstetrics and Gynaeocology, University of British Columbia, Vancouver, British Columbia, Canada; ^b^Psychology, Université De Montréal, Montreal, Quebec, Canada

**CONTACT** Lauren Rietchel laurenrietchel@alumni.ubc.ca

© 2020 The Author(s). Published with license by Taylor & Francis Group, LLC.

This is an Open Access article distributed under the terms of the Creative Commons Attribution License (http://creativecommons.org/licenses/by/4.0/), which permits unrestricted use, distribution, and reproduction in any medium, provided the original work is properly cited.

**Introduction/Aim**: The goal of this study was to evaluate the moderators of mindfulness-based cognitive therapy (MBCT) and cognitive behavioral therapy (CBT) to improve the treatment of provoked vestibulodynia (PVD), a chronic genital pain disorder.^1^ These treatments are shown to reduce self-reported pain and pain catastrophizing in PVD.^2,3^

**Methods**: One hundred and thirty women with PVD were assigned to a CBT or MBCT group. The following potential moderators were examined at pre-treatment: 1) age 2) pain duration 3) pain intensity 4) PVD subtype (acquired or life-long) 5) treatment credibility 6) the Five Facet Mindfulness Questionnaire (FFMQ) 7) the Big 5 personality domains.^4^ The outcome measures of sexual dysfunction, the numerical rating of pain, and pain catastrophizing were evaluated at pre- and post-treatment, and at 6- and 12-months follow-up.

Moderation was tested using multilevel models, nesting four time points within a participant. The interaction of the moderator, time effect, and treatment assignment was evaluated for significance and a simple slope analysis of significant interactions was performed.

**Results**: Treatment credibility moderated the pain intensity of women (*B *= 0.305, *p *< .01), where those with higher treatment credibility ratings improved more when they were in the MBCT group than the CBT group. Moreover, PVD subtype moderated pain catastrophizing (*B *= 3.150, *p *< .05). Those with lifelong PVD improved more in the CBT condition, while women with acquired PVD improved more in the MBCT condition. No other tested variables moderated outcomes.

**Discussion/Conclusions**: These findings may assist clinicians in individualizing psychological treatment recommendations for women seeking pain relief from their PVD.

References1.Harlow
BL, Wise
LA, Stewart
EG. Prevalence and predictors of chronic lower genital tract discomfort. Am J Obstet Gynecol. 2001;185(3):545–50. doi:10.1067/mob.2001.116748.115687752.Boyer
SC, Goldfinger
C, Thibault-Gagnon
S, Pukall
CF. Management of female sexual pain disorders. Adv Psychosom Med. 2011. doi:10.1159/000328810.220052063.Brotto
LA, Bergeron
S, Zdaniuk
B, Driscoll
M, Grabovac
A, Sadownik
LA, Smith
KB, Basson
R. A comparison of mindfulness-based cognitive therapy vs cognitive behavioral therapy for the treatment of provoked vestibulodynia in a hospital clinic setting. J Sex Med. 2019;16(6):909–23. doi:10.1016/j.jsxm.2019.04.002.311034814.Costa
PT, McCrea
RR.
Revised NEO personality inventory (NEO-PI-R) and NEO five-factor inventory (NEO-FFI) professional manual. Odessa (FL): Psychological Assessment Resources; 1992.

## Predictors of Perinatal Back Pain Persistence up 5 to 14 Months after Childbirth in Canada: A Biopsychosocial Perspective

Oluwakemi Awe^a,*^, Brenna Bath^b^ and Marwa Farag^a^

^a^School of Public Health, University of Saskatchewan, Saskatoon, Saskatchewan, Canada; ^b^School of Rehabilitation Science, College of Medicine, University of Saskatchewan, Saskatoon, Saskatchewan, Canada

**CONTACT** Oluwakemi Awe oluwakemi.awe@usask.ca

© 2020 The Author(s). Published with license by Taylor & Francis Group, LLC.

This is an Open Access article distributed under the terms of the Creative Commons Attribution License (http://creativecommons.org/licenses/by/4.0/), which permits unrestricted use, distribution, and reproduction in any medium, provided the original work is properly cited.

**Introduction/Aim**: Pregnancy-related back pain symptoms persist for some women. The aim of this study was to determine the prevalence and associated biopsychosocial predictors of perinatal back pain persisting up to 5 to 14 months postpartum among Canadian mothers.

**Methods**: We analyzed data from the 2006 Maternity Experiences Survey (MES). Weighted prevalence of persistent perinatal back pain was estimated for the full sample of women (n = 5,798), and a subsample who reported problematic back pain during the first 3 months postpartum (n = 2,251). Multiple logistic regression models were employed to identify factors associated with persistent perinatal back pain (yes/no) pain in each group.

**Results**: Overall, 16.3% of Canadian mothers reported persistent back pain at 5 to 14 months post-delivery. Among women who reported problematic back pain during the first 3 months postpartum, 45.6% continued to have un-resolved symptoms lasting 5–14 months after childbirth. The biopsychosocial predictors of persistent back pain in the full sample were: maternal age <20 years; immigrant status; obesity; poorer perceived health; high perceived stress; higher stressful life events; inadequate social support post-delivery; history of violent abuse; residence in Quebec and Ontario; and lower household income. Significant predictors among those whose back problems persisted from 3 months to 5–14 months were: immigrant status; poorer perceived health; higher stressful life events; inadequate social support post-delivery; history of violent abuse; and rural dwelling.

**Discussion/Conclusions**: Maternal care services and policies should consider biopsychosocial factors that may influence delayed recovery of back pain in the postpartum period when designing and implementing interventions.

## Validation and Trajectory Analysis of the Sensitivity to Pain Traumatization Scale

Samantha Fashler^a,*^, David Flora^a^, Aliza Weinrib^b^, Hance Clarke http://orcid.org/0000-0003-4975-3823^b^ and Joel Katz http://orcid.org/0000-0002-8686-447X^a^

^a^Psychology, York University, Toronto, Ontario, Canada; ^b^Anesthesia and Pain Management, Toronto General Hospital, Toronto, Ontario, Canada

**CONTACT** Samantha Fashler sfashler@yorku.ca

© 2020 The Author(s). Published with license by Taylor & Francis Group, LLC.

This is an Open Access article distributed under the terms of the Creative Commons Attribution License (http://creativecommons.org/licenses/by/4.0/), which permits unrestricted use, distribution, and reproduction in any medium, provided the original work is properly cited.

**Introduction/Aim**: The Sensitivity to Pain Traumatization Scale (SPTS-12) was created to assess the propensity to develop a traumatic stress response to pain. Despite preliminary support, the SPTS-12 has yet to be examined over time and its predictive validity has not been evaluated. The aim of the present study is to investigate how SPTS-12 scores change over time in a clinical sample of patients receiving care after surgery from the Toronto General Hospital Transitional Pain Service.

**Methods**: A sample of 361 adults (55% male; *M*_age_ = 50.6 years, *SD*_age_ = 14.3) completed questionnaires assessing symptoms of pain, anxiety, depression, and trauma at multiple visits to the Transitional Pain Service after surgery. Latent-class mixed-model analysis was used to estimate latent trajectories of SPTS-12 scores across days after surgery. One-way ANOVAs were then used to determine how trajectory classes differed over time on measures of morphine use, average pain intensity, pain interference, and depressive symptoms.

**Results**: The final model consisted of five SPTS-12 trajectory groups, three of which were characterized by significantly decreasing scores over time. Analysis of pain-related outcomes predicted by SPTS-12 trajectories provided evidence of criterion validity of the SPTS-12. SPTS-12 trajectories did not significantly differ on morphine use at any time point. SPTS-12 trajectories for average pain, pain interference, and depression scores differed at two or more postsurgical visits (all *p* < .001).

**Discussion/Conclusions**: The present results support the long-term stability of SPTS-12 scores, as well as the ability of the SPTS-12 to predict important pain-related outcomes over time.

## Psychological Characteristics Associated with Treatment Benefit in a Digital Psychological Intervention for Pediatric Chronic Pain

Rocio de la Vega http://orcid.org/0000-0002-2517-6948^a,*^ and Tonya M. Palermo http://orcid.org/0000-0001-6036-6715^a^

^a^Center for Child Health, Behavior and Development, Seattle Children’s Research Institute, Seattle, Washington, USA

**CONTACT** Rocio de la Vega rocio.delavega@seattlechildrens.org

© 2020 The Author(s). Published with license by Taylor & Francis Group, LLC.

This is an Open Access article distributed under the terms of the Creative Commons Attribution License (http://creativecommons.org/licenses/by/4.0/), which permits unrestricted use, distribution, and reproduction in any medium, provided the original work is properly cited.

**Introduction/Aim:** Digital psychological interventions have shown efficacy for improving disability in adolescents with chronic pain. However, little is known about the psychological characteristics that contribute to positive changes during treatment. The aim of this study is to examine baseline psychological characteristics (diagnosis uncertainty, readiness to change [RtC]) as predictors of treatment perceptions (expectancies, helpfulness) and treatment outcomes.

**Methods:** Youth with chronic pain were recruited from a tertiary care pediatric pain clinic. Youth completed measures of: sociodemographics, diagnostic uncertainty, sleep, pain, interference, executive function, RtC, and treatment perceptions at baseline (T1), mid-treatment (T2), post-treatment (T3) and 3-month follow-up (T4). All youth received access to WebMAP: an 8-module online Cognitive Behavioral Treatment that teaches pain coping skills.

**Results:** Data collection is ongoing. Seventy-two adolescents have been recruited, from which 69 (Mage = 15.4, SD = 1.4; 74% female) completed T1, and N = 51 completed T2. As hypothesized, diagnosis uncertainty was negatively associated with RtC, that is, participants without a clear diagnosis scored higher in Precontemplation (t (66) = 2.95, *P* = .004) and Contemplation (t (66) = 2.45, *P* = .017) and lower in Action/Maintenance (t (66) = −3.40, *P* = .001). As expected, treatment expectancies were negatively associated with Precontemplation scores (r = −.33). Moving to perceptions about the treatment, baseline RtC was associated with perceptions of helpfulness of the treatment skills at T2, as predicted. Specifically, perceived helpfulness was negatively correlated with baseline Precontemplation (r = −.34) and positively correlated with Action/Maintenance (r = .37).

**Discussion/Conclusions:** These preliminary results show that diagnosis uncertainty is associated with pre-treatment RtC, which may impact perceptions and expectancies about treatment. Further research exploring these associations is warranted.

## Parent Worry and Stress: Critical Factors in Understanding Parent Pain Ratings during Vaccination in the Second Year of Life

Hannah Gennis^a,*^, Shaylea Badovinac^a^, Rebecca Pillai Riddell^a^, Daniel Flanders^b,c^, Eitan Weinberg^b,c^ and Hartley Garfield^c^

^a^Psychology, York University, Toronto, Ontario, Canada; ^b^Kindercare Pediatrics, Toronto, Ontario, Canada; ^c^Pediatrics, University of Toronto, Toronto, Ontario, Canada

**CONTACT** Hannah Gennis hgennis@yorku.ca

© 2020 The Author(s). Published with license by Taylor & Francis Group, LLC.

This is an Open Access article distributed under the terms of the Creative Commons Attribution License (http://creativecommons.org/licenses/by/4.0/), which permits unrestricted use, distribution, and reproduction in any medium, provided the original work is properly cited.

**Introduction/Aim**: To understand child and parent factors that predict parent pain ratings during vaccination in the second year of life.

**Methods**: This data is from an ongoing longitudinal study (OUCH Cardio Cohort) that follows parent-child dyads during vaccination at 12 months (*n* = 121), 18 months (*n* = 67), and 24 months (*n* = 39). Children’s pain was measured using the FLACC scale (Merkel et al., 1997) immediately, and at one- and two minutes post-needle. Parents rated their worry pre- and post-needle, as well as their child’s pain post-needle using Likert scales. Parent stress (PSI; Abidin, 2012) and psychological distress (BSI; Derogatis, 2001) were also measured. A hierarchical regression analysis was run at all three ages with parent pain ratings as the outcome variable. Child sex was entered in the model first, then children’s pain behaviors, then parent variables.

**Results**: At 12 months, parent worry pre-needle (*ß* = .21, *p* = .03) and parenting stress (*ß *= .21, *p* = .04) predicted parent pain beyond children’s pain behaviors (1 min post-needle *ß* = .23, *p* = .02). At 18 months, parent worry post-needle significantly predicted parent pain ratings (standardized *ß* = .32, *p* = .02), controlling for all other variables. The model at 24 months was not significant.

**Discussion/Conclusions**: Parent worry and stress are important factors contributing to parent pain ratings. Given the importance of accurate pain assessment for the proper management of children’s pain, efforts to address parent worry and stress should be considered during vaccination appointments.

## Healthcare Provider Barriers and Facilitators for Prescribing Opioids for Chronic Pain in North America: A Systematic Review of Qualitative Research

Louise V. Bell^a,*^, Michael Asamoah-Boaheng^b^, Oluwatosin A. Badejo^b^, Norman Buckley http://orcid.org/0000-0002-1031-6813^c^, Jason W. Busse http://orcid.org/0000-0002-0178-8712^d^, Tavis S. Campbell^e^, Kimberly Corace^f^, Lynn Cooper^g^, David Flusk^h^, David A. Garcia^b^, Mohammad A. Hossain^b^, Alfonso Iorio http://orcid.org/0000-0002-3331-8766^i^, Kim L. Lavoie^j^, Patricia A. Poulin^k^, Becky Skidmore^l^ and Joshua A. Rash http://orcid.org/0000-0003-0927-0712^a^

^a^Psychology, Memorial University of Newfoundland, St. John’s, Newfoundland, Canada; ^b^Clinical Epidemiology, Memorial University of Newfoundland, St. John’s, Newfoundland, Canada; ^c^Anesthesia, McMaster University, Hamilton, Ontario, Canada; ^d^Anesthesia, Health Research Methods, Evidence and Impact, The Michael G. DeGroote Institute for Pain Research and Care, The Michael G. DeGroote Centre for Medicinal Cannabis Research, McMaster University, Hamilton, Ontario, Canada; ^e^Psychology, University of Calgary, Calgary, Alberta, Canada; ^f^The Royal Ottawa Mental Health Centre, Psychiatry, University of Ottawa, Ottawa, Ontario, Canada; ^g^Canadian Injured Workers Alliance, Thunderbay, Ontario, Canada; ^h^Anesthesia, Memorial University of Newfoundland, St. John’s, Newfoundland, Canada; ^i^Health Research Methods, Evidence and Impact, McMaster University, Hamilton, Ontario, Canada; ^j^Hopital du Sacre-Coeur de Montreal, Psychology, Montreal Behavioural Medicine Centre, Centre intégrée universitaire de santé et services sociaux de Nord de l’Ile de Montreal (CIUSSS-NIM), Montreal, Quebec, Canada; ^k^The Ottawa Hospital Research Institute, The Ottawa Hospital Pain Clinic, School of Psychology & Anesthesiology and Pain Medicine, University of Ottawa, Ottawa, Ontario, Canada; ^l^Independent Information Specialist, Ottawa, Ontario, Canada

**CONTACT** Louise V. Bell lvb603@mun.ca

© 2020 The Author(s). Published with license by Taylor & Francis Group, LLC.

This is an Open Access article distributed under the terms of the Creative Commons Attribution License (http://creativecommons.org/licenses/by/4.0/), which permits unrestricted use, distribution, and reproduction in any medium, provided the original work is properly cited.

**Introduction/Aim**: The aim of this review was to identify empirically supported barriers and facilitators for prescribing opioids for Chronic Non-Cancer Pain (CNCP) through a systematic review of qualitative literature.

**Methods**: Six databases were searched from inception to June 3, 2019 for qualitative studies reporting on provider knowledge, attitudes, beliefs, or practices pertaining to prescribing opioids for CNCP in North America. Data were extracted, risk of bias rated, and confidence in evidence graded using Cochrane Confidence in the Evidence from Reviews of Qualitative research (CERQual). Constructs identified were coded using the Theoretical Domains Framework

**Results**: Twenty-six studies reporting on 599 healthcare providers were included. Themes were extracted from data and fourteen constructs were identified that contributed as barriers or facilitators to prescribing opioids for CNCP. Barriers to prescribing opioids included perceived inadequate education (moderate confidence), self-efficacy to prescribe opioids for complex cases (low confidence), regulatory scrutiny (moderate confidence), opioid diversion (moderate confidence), patient- related (e.g., overdose) salient events (high confidence), provider-related (e.g., threat) salient events (high confidence), insufficient time (high confidence), and concerns related to patient misuse (high confidence). Facilitators included a positive patient-provider relationship (moderate confidence), patient-provider communication (low confidence), education on opioid prescribing tools (low confidence), lack of available non-opioid alternatives (moderate confidence), setting goals with patients (moderate confidence), and institutional pressure (low confidence).

**Discussion/Conclusions**: Understanding the barriers and facilitators that influence opioid-prescribing offers important insight into modifiable targets for interventions. Such interventions can support providers in delivering care consistent with guidelines to manage CNCP, while minimizing risks.

## Mitochondrial Changes in Peripheral Sensory Neurons in Response to Inflammatory Stimuli

Aislinn Maguire^a,*^, Fajr Haq^b^ and Bradley Kerr^c^

^a^Neuroscience, University of Alberta, Edmonton, Alberta, Canada; ^b^Physiology, University of Alberta, Edmonton, Alberta, Canada; ^c^Anesthesiology and Pain Medicine, University of Alberta, Edmonton, Alberta, Canada

**CONTACT** Aislinn Maguire maguire@ualberta.ca

© 2020 The Author(s). Published with license by Taylor & Francis Group, LLC.

This is an Open Access article distributed under the terms of the Creative Commons Attribution License (http://creativecommons.org/licenses/by/4.0/), which permits unrestricted use, distribution, and reproduction in any medium, provided the original work is properly cited.

**Introduction/Aim**: Sensory neurons in the dorsal root ganglion (DRG) have been shown to become hyper-excitable/sensitized in response to chronic inflammatory stimuli as often occurs in autoimmune disorders like rheumatoid arthritis or Multiple Sclerosis (MS). This sensitization in response to inflammation has been postulated to be an underlying cause of the chronic pain associated with these disorders. The molecular pathways mediating sensitization, however, remain to be fully elucidated. We hypothesize that morphological and functional changes in the mitochondria of nociceptors (pain sensing sensory neurons) are involved in this process. Given the well-established sex bias of painful autoimmune disorders, we also predict that these changes are more prominent in female nociceptors.

**Methods**: We have begun to test our hypotheses by stimulating male and female DRG sensory neurons *in vitro* with physiologically relevant doses of the inflammatory cytokine TNFα and examining changes in mitochondrial morphology.

**Results**: We find that both 24 and 48 hours of TNFα stimulation lead to significant changes in mitochondrial morphology. Interestingly, these changes were only evident in female sensory neurons.

**Discussion/Conclusions**: Ongoing studies are evaluating functional readouts of male and female sensory neurons such as cellular respiration and reactive oxygen species production in response to TNFα stimulation. These pathways may represent novel targets to treat pain associated with chronic, neuroinflammatory disorders and also shed light on why pain in these conditions is biased toward females.

## Subgroups of Pain Patterns in Children and Adolescents with Cancer in China

Wen Zhang^a,*^, Jennifer Stinson^b^, Qingmei Huang^a^, Jiashu Wang^c^, Lei Cheng^a^ and Changrong Yuan^a^

^a^School of Nursing, Fudan University, Shanghai, China; ^b^Lawrence S. Bloomberg Faculty of Nursing, the Hospital for Sick Children, Child Health Evaluative Sciences and the University of Toronto, Toronto, ON, Canada; ^c^School of Nursing and Health Management, Shanghai University of Medicine and Health Sciences, Shanghai, China

**CONTACT** Wen Zhang zhangwenivy@aliyun.com

© 2020 The Author(s). Published with license by Taylor & Francis Group, LLC.

This is an Open Access article distributed under the terms of the Creative Commons Attribution License (http://creativecommons.org/licenses/by/4.0/), which permits unrestricted use, distribution, and reproduction in any medium, provided the original work is properly cited.

**Introduction/Aim**: Pain is a distressing symptom for children and adolescents with cancer; and has been shown to have individual variability. This study sought to determine subgroups of pain patterns (pain intensity, duration, interference and control over pain) using latent profile analysis (LPA), as well as examining how these subgroups differed on demographic and patient-reported outcomes.

**Methods**: A total of 187 children and adolescents (8 to 17) with cancer in China were prospectively recruited and asked to complete the following measures: pain intensity, pain duration, pain interference and pain control using the Chinese translation of validated questions from the Pain Squad Cancer App, as well as 7 short forms of symptoms and function using Pediatric PROMIS. LPA was used to identify latent subgroups of pain patterns.

**Results**: Three latent classes were identified: Low (68.3%), Continuous/Chronic (19.9%), and High/Acute (55.3%) class. Children in High/Acute class were more likely to be cared by unemployed guardians. Inpatient children were more likely to be in the Continuous/Chronic class. With the exception of Peer Relationships, the other patient-reported outcomes (depressive symptoms (*p *= .001), anger (*p *< .001), anxiety (*p *= .040), and fatigue (*p *= .039), mobility (*p *= .010)), were significantly higher in High/Acute than in Low class. There was no significant difference for upper extremity function in pairwise comparison, but significant as a whole (*p *= .038).

**Discussion/Conclusions**: Three distinct pain subgroups demonstrate the heterogeneity in pain patterns among Chinese children and adolescents with cancer. These subgroupings can assist clinicians to better identify and target treatments for patients at higher risk of pain.

## Dialectical Behaviour Therapy Skills Training for Chronic Pain: A Mixed-Method Analysis of Group Treatment Satisfaction

Kirsten M. Gullickson^a^, Brandon K. Ulmer^a^, Mark K. Simmonds^b^ and Bruce D. Dick^b,*^

^a^Multidisciplinary Pain Clinic, Alberta Health Services, Edmonton, Alberta, Canada; ^b^Anesthesiology and Pain Medicine, University of Alberta, Edmonton, Alberta, Canada

**CONTACT** Bruce D. Dick bruce.dick@ualberta.ca

© 2020 The Author(s). Published with license by Taylor & Francis Group, LLC.

This is an Open Access article distributed under the terms of the Creative Commons Attribution License (http://creativecommons.org/licenses/by/4.0/), which permits unrestricted use, distribution, and reproduction in any medium, provided the original work is properly cited.

**Introduction/Aim:** Individuals referred to chronic pain clinics often experience difficulties with emotion regulation, distress tolerance, mindfulness, and interpersonal effectiveness as a result of their pain. Thus, the University of Alberta Multidisciplinary Pain Clinic began offering Pain 401, a Dialectical Behavior Therapy (DBT) skills training group, to chronic pain patients with the goal of improving psychosocial functioning and quality of life. The aim of the current study was to assess patients’ satisfaction with this novel chronic pain treatment.

**Methods**: Pain 401 consists of twelve 90-minute sessions that cover four skill areas. At post-treatment, patients were asked to complete a treatment satisfaction questionnaire that included Likert-style rating items and open-ended questions. Quantitative feedback was analyzed using descriptive statistics and qualitative feedback was analyzed using content analysis.

**Results**: Fifteen patients returned their treatment satisfaction questionnaire. Overall, patients were extremely satisfied with Pain 401 (*M* = 4.73/5; *SD* =.46). Every patient said the group was worth their time and they would recommend it to others with chronic pain. Patients rated all the skills as helpful, but rated distress tolerance and emotion regulation most highly. Emotion regulation was rated the most difficult skill. Patients’ open-ended responses illustrated the most valued aspects of the treatment and included suggestions for group improvement.

**Discussion/Conclusions**: Patients with chronic pain reported high satisfaction with a 12-session DBT skills training group. Qualitative feedback provided by patients will be used to improve future offerings of Pain 401. Treatment satisfaction will be considered in combination with treatment effectiveness when evaluating group outcomes.

## Complementary and Alternative Medicine Recommendations are of Lower Quality than Overall Recommendations in Rheumatoid and Osteoarthritis Guidelines: A Systematic Review

Jeremy Y. Ng http://orcid.org/0000-0003-0031-5873^a,*^, Ashlee Azizudin^a^ and Jason W. Busse http://orcid.org/0000-0002-0178-8712^b,c,d^

^a^Department of Health Research Methods, Evidence, and Impact, McMaster University, Hamilton, Ontario, Canada; ^b^Departments of Anesthesia and Health Research Methods, Evidence and Impact (HEI), McMaster University, Hamilton, Ontario, Canada; ^c^Michael G. DeGroote Institute for Pain Research and Care, Hamilton, Ontario, Canada; ^d^Michael G. DeGroote Centre for Medicinal Cannabis Research, Hamilton, Ontario, Canada

**CONTACT** Jeremy Y. Ng ngjy2@mcmaster.ca

© 2020 The Author(s). Published with license by Taylor & Francis Group, LLC.

This is an Open Access article distributed under the terms of the Creative Commons Attribution License (http://creativecommons.org/licenses/by/4.0/), which permits unrestricted use, distribution, and reproduction in any medium, provided the original work is properly cited.

**Introduction/Aim**: Sixty percent of arthritis patients in the UK have used complementary and alternative medicine (CAM) approaches. Despite this, the quality of CAM recommendations in clinical practice guidelines (CPGs) for arthritis is uncertain. We reviewed published CPGs to identify and evaluate the quality of CAM recommendations for the treatment or management of arthritis.

**Methods**: We systematically searched MEDLINE, EMBASE, CINAHL and Guidelines International Network databases. We also searched the National Center for Complementary and Integrative Health website. Eligible articles were CPGs for the treatment or management of rheumatoid arthritis and osteoarthritis. Two independent reviewers evaluated the quality of reporting for each guideline that provided CAM recommendations, and the specific section providing CAM recommendations, using the AGREE II instrument.

**Results**: Scaled domain percentages from highest to lowest were (overall, CAM): clarity of presentation (92.2%, 94.1%), scope and purpose (90.4%, 87.4%), rigour of development (72.6%, 64.2%), stakeholder involvement (64.8%, 49.6%), editorial independence (61.1%, 60.6%), and applicability (51.4%, 33.3%).

**Discussion/Conclusion**: Approximately half of arthritis CPGs finclude CAM recommendations. In those that do, a gap exists in that the quality of CAM recommendations are of significantly lower quality than overall recommendations across the stakeholder involvement, rigor of development, and applicability domains.

## The Impact of Remifentanil on Post-operative Opioid Consumption and Pain in Adolescent Idiopathic Scoliosis Repair

Stephanie Schwindt^a,*^, Richa Sharma^a^, Rebecca Dubé^b^, Benjamin Steinberg^a^ and Stephen Brown^a^

^a^Department of Anesthesia and Pain Medicine, The Hospital for Sick Children, Toronto, Ontario, Canada; ^b^Centre hospitalier universitaire Sainte-Justine, Université de Montréal, Montreal, Quebec, Canada

**CONTACT** Stephanie Schwindt stephanie.schwindt@mail.utoronto.ca

© 2020 The Author(s). Published with license by Taylor & Francis Group, LLC.

This is an Open Access article distributed under the terms of the Creative Commons Attribution-NonCommercial License (http://creativecommons.org/licenses/by-nc/4.0/), which permits unrestricted non-commercial use, distribution, and reproduction in any medium, provided the original work is properly cited.

**Introduction/Aim**: Posterior spinal fusion surgery for adolescent idiopathic scoliosis (AIS) is associated with significant postoperative pain. Remifentanil, an opioid analgesic commonly infused during spinal fusion surgery, has been associated with increased postoperative opioid consumption. The primary objective of this study is to correlate intraoperative remifentanil dose with postoperative opioid consumption in a cohort of AIS patients.

**Methods**: Our study, conducted with SickKids REB approval, is a retrospective chart review of patients aged 12–18 who have undergone posterior spinal fusion for AIS repair by one surgeon at SickKids from 2011–2017. We collected data on baseline characteristics, surgical complexity, perioperative analgesic agents and postoperative pain.

**Results**: Data for 85 patients has been extracted. The sample population is 83.5% female with a mean (SD) age of 14.8 (1.51) years and weight of 56.8 (14.33) kg. The perioperative course of our study population is characterized by a mean (SD) OR duration of 427 (65.25) minutes, 12.1 (1.39) vertebrae instrumented, 1.07 (0.31) days to ambulation and 4.8 (0.89) days to discharge. An interim analysis of the correlation between total weight-adjusted remifentanil dose and total postoperative weight-adjusted opioid dose at 96 hours produced an R^2^ value of 0.0004.

**Discussion/Conclusions**: There is no evident correlation between remifentanil dosing and postoperative opioid consumption in our interim analysis. This lack of correlation requires corroboration with the complete data set. Our results are intended to help guide the development of multimodal analgesic strategies for posterior spinal fusion that optimize acute pain control, while minimizing postoperative opioid consumption.

## Complementary and Alternative Medicine Recommendations in Guidelines for Low Back Pain are Common, but of Lower Quality than Overall Recommendations

Jeremy Y. Ng http://orcid.org/0000-0003-0031-5873^a,*^, Uzair Mohiuddin^a^ and Jason W. Busse http://orcid.org/0000-0002-0178-8712^b,c,d^

^a^Department of Health Research Methods, Evidence, and Impact, McMaster University, Hamilton, Ontario, Canada; ^b^Departments of Anesthesia and Health Research Methods, Evidence and Impact (HEI), McMaster University, Hamilton, Canada; ^c^Michael G. DeGroote Institute for Pain Research and Care, Hamilton, Ontario, Canada; ^d^Michael G. DeGroote Centre for Medicinal Cannabis Research, Hamilton, Ontario, Canada

**CONTACT** Jeremy Y. Ng ngjy2@mcmaster.ca

© 2020 The Author(s). Published with license by Taylor & Francis Group, LLC.

This is an Open Access article distributed under the terms of the Creative Commons Attribution License (http://creativecommons.org/licenses/by/4.0/), which permits unrestricted use, distribution, and reproduction in any medium, provided the original work is properly cited.

**Introduction/Aim**: Approximately 40% of North American adults with low back pain (LBP) use complementary and alternative medicine (CAM). We explored the prevalence of CAM recommendations among clinical practice guidelines (CPGs) for low back pain (LBP), and assessed their reporting quality compared to overall quality of guideline recommendations.

**Methods**: We searched MEDLINE, EMBASE, CINAHL, and Guidelines International Network databases, along with the National Center for Complementary and Integrative Health website. Two reviewers evaluated the quality of reporting for each guideline that provided CAM recommendations, and the specific section providing CAM recommendations, using the AGREE II instrument. We used the Wilcoxon signed rank test to explore for differences.

**Results**: Of 181 unique search results, 22 LBP CPGs were found, 17 of which made CAM recommendations. With regards to scaled domain percentages, this overall guideline scored significantly higher than the CAM section for 4 of 6 domains (overall, CAM): (1) scope and purpose (89%, 87%, *p* = .07), (2) clarity of presentation (83%, 73%, *p* = .001), (3) stakeholder involvement (57%, 42%, *p* = .005), (4) rigor of development (47%, 45%, *p* = .003), (5) editorial independence (35%, 35%, *p* = 1.00), and (6) applicability (32%, 22%, *p* = .001).

**Discussion/Conclusions**: The majority of LBP guidelines provide CAM recommendations; however, the quality of CAM recommendations are significantly lower than overall recommendations across a number of domains.

## Psychological and Social Consequences of Dysmenorrhea in Adolescents

Kayla M. Wall^a,*^ and Michelle M. Gagnon^a^

^a^Psychology, University of Saskatchewan, Saskatoon, Saskatchewan, Canada

**CONTACT** Kayla M. Wall kayla.wall@usask.ca

© 2020 The Author(s). Published with license by Taylor & Francis Group, LLC.

This is an Open Access article distributed under the terms of the Creative Commons Attribution License (http://creativecommons.org/licenses/by/4.0/), which permits unrestricted use, distribution, and reproduction in any medium, provided the original work is properly cited.

**Introduction/Aim**: Dysmenorrhea is the most prevalent gynecological concern among adolescents. Although previous research has suggested that adolescents with dysmenorrhea are at risk for poor psychological and social functioning, the impact of dysmenorrhea on psychological and social functioning in North American adolescents has been neglected. We examined pain-related menstrual characteristics along with the relationship between dysmenorrhea and psychological and social functioning, among North American adolescents. We hypothesized that more severe dysmenorrhea symptoms would be associated with more symptoms of depression and anxiety, and that adolescents would report that menstrual pain negatively impacts their social functioning.

**Methods**: This study is a preliminary investigation using data being collected as part of a larger investigation on menstrual pain and adolescent health. We recruited adolescents aged 14 to 18 living in North America to complete an online survey. Following receipt of parental consent and adolescent assent, adolescents completed measures of menstrual characteristics, social functioning, anxiety and depression.

**Results**: Thirty-eight adolescents girls completed the survey (*M*age = 15.92). The majority of participants (74%) reported physical pain with menstruation. There was a significant positive relationship between menstrual symptom severity and symptoms of anxiety, *r* =.63, *p* <.001 and symptoms of depression, *r* =.61, *p* <.001. Further, 43% of teens indicated that their social activities were limited by menstrual pain.

**Discussion/Conclusions**: Consistent with research in other regions, North American adolescents are reporting that dysmenorrhea negatively impacts their psychological and social functioning. Implications are discussed.

## “I’m Not Sure She Even Knows What a Pain-free Life Looks Like”: Parent Perspectives of Their Child’s Arthritis Pain

Yvonne N. Brandelli^a,*^, Christine T. Chambers http://orcid.org/0000-0002-7138-916X^b^, Perri R. Tutelman^a^, Jennifer Stinson http://orcid.org/0000-0002-9969-8052^c^, Adam M. Huber^d^, Jennifer Wilson^e^ and Emma S. Cameron^f^

^a^Psychology and Neuroscience, Dalhousie University, Halifax, Nova Scotia, Canada; ^b^Psychology and Neuroscience & Pediatrics, Dalhousie University & IWK Health Centre, Halifax, Nova Scotia, Canada; ^c^Lawrence S. Bloomberg Faculty of Nursing, University of Toronto and Research Institute at the Hospital for Sick Children, Toronto, Ontario, Canada; ^d^Pediatrics & Rheumatology, Dalhousie University & IWK Health Centre, Halifax, Nova Scotia, Canada; ^e^Cassie and Friends, Vancouver, British Columbia, Canada; ^f^School of Health and Human Performance, Dalhousie University, Halifax, Nova Scotia, Canada

**CONTACT** Yvonne N. Brandelli Yvonne.Brandelli@dal.ca

© 2020 The Author(s). Published with license by Taylor & Francis Group, LLC.

This is an Open Access article distributed under the terms of the Creative Commons Attribution License (http://creativecommons.org/licenses/by/4.0/), which permits unrestricted use, distribution, and reproduction in any medium, provided the original work is properly cited.

**Introduction/Aim**: Over 70% of children with arthritis experience some degree of pain, demonstrating the commonality of this experience. Despite the wealth of research exploring arthritis pain, little is known about the parent perspective, which is important given the impact of pain on the broader family unit. The purpose of this study was to explore parents’ perspectives of their child’s arthritis pain.

**Methods**: In partnership with Cassie and Friends, 169 parents of children with juvenile idiopathic arthritis were recruited online and through social media to participate in an online survey. Parents (95% mothers) provided responses to an open-ended question describing what resonated with them about their child’s arthritis pain. Data were analyzed using descriptive content analysis.

**Results**: Parent perspectives clustered into five main categories: their child’s pain presentation (e.g., the invisibility of pain), their child’s pain communication (e.g., difficulties indicating when they are in pain), the pain journey (e.g., the chronicity and rollercoaster-like experiences), the impact of pain on their child and themselves (e.g., affecting mental health and relationships), and their child’s strength and resilience (e.g., perseverance).

**Discussion/Conclusions**: Parents described various aspects of their child’s pain as salient, illuminating the complex experiences of arthritis pain from their unique perspectives. While some reflected on the barriers that pain had created, others shared ways their family had adjusted. Understanding the diversity of experiences may allow healthcare professionals to tailor their care, thereby more effectively supporting families. This knowledge may also guide the dissemination of evidence-based pain management strategies to the childhood arthritis community.

## Quantifying Robustness of the SUCRA-Based Opioids Ranks in a Network Meta-Analysis (NMA) in Chronic Non-Cancer Pain

Atefeh Noori^a,*^, Jason W. Busse http://orcid.org/0000-0002-0178-8712^a,b,c^, Reed A. Siemieniuk^a^, Behnam Sadeghirad^a^, Li Wang^a^, Rachel Couban^a^, Luis Montoya^d^, Patrick Jiho Hong^e^, Edward Zhou^f^, David Juurlink^g^, Lehana Thabane^a^ and Gordon H. Guyatt^a^

^a^Department of Health Research Methods, Evidence, and Impact (HEI), McMaster University, Hamilton, Ontario, Canada; ^b^Michael G. DeGroote Institute for Pain Research and Care, McMaster University, Hamilton, Ontario, Canada; ^c^Michael G. DeGroote Centre for Medicinal Cannabis Research, McMaster University, Hamilton, Ontario, Canada; ^d^Orthopaedic Surgery Arthritis Program, Krembil Research Institute, University Health Network, Toronto, Ontario, Canada; ^e^Department of Anesthesia, University of Toronto, Toronto, Ontario, Canada; ^f^Department of Family Medicine, McMaster University, Hamilton, Ontario, Canada; ^g^Department of Medicine, University of Toronto, Toronto, Ontario, Canada

**CONTACT** Atefeh Noori nooria3@mcmaster.ca

© 2020 The Author(s). Published with license by Taylor & Francis Group, LLC.

This is an Open Access article distributed under the terms of the Creative Commons Attribution License (http://creativecommons.org/licenses/by/4.0/), which permits unrestricted use, distribution, and reproduction in any medium, provided the original work is properly cited.

**Introduction**/**Aim**: An inherent appeal of network meta-analysis (NMA) is the ability to rank competing treatments using the surface under the cumulative ranking curve (SUCRA) approach; however, the validity of these rankings is uncertain. We evaluated the concordance between the SUCRA approach and a minimally-contextualized GRADE assessment.

**Methods**: We searched MEDLINE/PubMed, EMBASE, CINAHL, PsycInfo, and CENTRAL, up to March 2019 for randomized trials exploring opioids vs. another opioid or placebo for chronic noncancer pain. We constructed a frequentist NMA that explored the relative effect of each opioid on pain relief. We then estimated the SUCRA for each opioid from 0% (worst) to 100% (best). Concurrently, we assessed the certainty of evidence using the GRADE approach (dichotomized as “moderate-to-high” or “low-to-very-low” certainty of evidence) and categorized opioids first based on their effectiveness vs. placebo and then vs. other competing opioids, and finally according to GRADE ratings.

**Results**: We included 76 studies with 21,752 patients that evaluated 15 individual opioids. The SUCRA method suggested codeine-extended release (94%) and oxymorphone-ER (88%) as the best opioids for pain relief. The certainty of evidence for both these drugs relative to placebo was, however, low. All comparisons supported by moderate–to-high certainty-evidence demonstrated that opioids were more effective than placebo, but that none were superior to others.

**Discussion/Conclusion**: Our findings suggest that apparent differences in effectiveness between opioids, when ranked according to SUCRA, result from the failure to consider the certainty of evidence.

## Inhibition of the Pain-related Pupil Dilation Response during Pain Inhibition by Distraction

Alice Wagenaar-Tison^a^, Antoine Bergeron^b^, Zoha Deldar^a^, Stéphane Northon^a^, Nabi Rustamov^a^, Sylvain Sirois^b^ and Mathieu Piché^a,*^

^a^Department of Anatomy, Université Du Québec À Trois-Rivières, Trois-Rivières, Québec, Canada; ^b^Department of Psychology, Université Du Québec À Trois-Rivières, Trois-Rivières, Québec, Canada

**CONTACT** Mathieu Piché mathieu.piche@uqtr.ca

© 2020 The Author(s). Published with license by Taylor & Francis Group, LLC.

This is an Open Access article distributed under the terms of the Creative Commons Attribution License (http://creativecommons.org/licenses/by/4.0/), which permits unrestricted use, distribution, and reproduction in any medium, provided the original work is properly cited.

**Introduction/Aim**: It has been reported that pain inhibition is associated with facilitation of the nociceptive flexion reflex (NFR) during a mental arithmetic task (distraction). This sensorimotor dissociation may reflect the regulation of stimulus saliency to facilitate protective motor behaviors. Besides, pain evokes a pupil dilation response (PRD), which reflects stimulus saliency. The aim of this study was to clarify whether NFR facilitation during pain inhibition by distraction reflects changes in saliency, as indexed by the PDR.

**Methods**: Twenty healthy volunteers were recruited in the study. All participants received a series of 90 painful stimuli at the right ankle, distributed equally in three conditions: control, anticipation and distraction. Anticipation was produced by a visual cue presented 1 s before painful stimuli. Distraction was produced by a mental arithmetic task performed during painful stimulation. Pain was measured using a visual analogue scale. NFR and PDR were measured using electromyographic and pupillometric methods.

**Results**: Pain ratings were significantly decreased by distraction (*p* < .001), but not by anticipation (*p* = .7). NFR amplitude tended to increase during distraction, but was not significantly different between conditions (*p* = .3). Pupil diameter increased between 500 and 1000 ms post-stimulus in the control condition, but this response was abolished by distraction (*p* < .05). Moreover, anticipation decreased pupil diameter compared with control, between −500 and −200 ms pre-stimulus (*p* < .05).

**Discussion/Conclusions**: These results indicate that the saliency of pain stimuli is decreased during pain inhibition by distraction. The dissociation between pain and NFR amplitude is suggestive of a motor facilitation independent of saliency.

## The Quality of, and Discrepancies Among, Network Meta-Analyses for Pharmacologic Management of Knee Osteoarthritis: A Systematic Survey

Mark Phillips^a,*^, Herman Johal^a^, Raman Mundi^a^, Ashaka Patel^b^, Jason W. Busse http://orcid.org/0000-0002-0178-8712^c,d,e^, Lehana Thabane^f,g^ and Mohit Bhandari http://orcid.org/0000-0003-3556-9179^a^

^a^Department of Health Research Methods, Evidence, and Impact and Division of Orthopaedic Surgery, McMaster University, Hamilton, Ontario, Canada; ^b^Department of Health Sciences, McMaster University, Hamilton, Ontario, Canada; ^c^Departments of Anesthesia and Health Research Methods, Evidence and Impact (HEI), McMaster University, Hamilton, Ontario, Canada; ^d^Michael G. DeGroote Institute for Pain Research and Care, McMaster University, Hamilton, Ontario, Canada; ^e^Michael G. DeGroote Centre for Medicinal Cannabis Research, McMaster University, Hamilton, Ontario, Canada; ^f^Department of Health Research Methods, Evidence, and Impact, McMaster University, Hamilton, Ontario, Canada; ^g^Biostatistics Unit, St Joseph’s Healthcare Hamilton, Hamilton, Ontario, Canada

**CONTACT** Mark Phillips phillimr@mcmaster.ca

© 2020 The Author(s). Published with license by Taylor & Francis Group, LLC.

This is an Open Access article distributed under the terms of the Creative Commons Attribution License (http://creativecommons.org/licenses/by/4.0/), which permits unrestricted use, distribution, and reproduction in any medium, provided the original work is properly cited.

**Introduction/Aim**: The pharmacological management of pain secondary to knee osteoarthritis (OA) has been the focus of numerous network meta-analyses (NMAs); however, they have reported different conclusions. We systematically evaluated this literature.

**Methods**: We conducted a systematic literature search for all published NMAs addressing non-surgical management of painful knee OA. We evaluated the quality of reporting with the 30-item PRISMA checklist for NMAs, methodological differences between reviews, and how these differences contributed to inconsistencies in findings.

**Results**: Eight NMAs were eligible for review. Adherence to the PRISMA reporting checklist was variable, with scores ranging from 7/30 to 29/30 items reported. Studies most frequently reported the study rationale and objectives in their introduction (8/8 studies), and the conclusions clearly at the end of the study (8/8 studies). The least frequently reported item was registration of a systematic review protocol (1/8 studies). The length of follow-up ranged from 4 weeks to beyond 52 weeks. Among NMAs that assessed both oral and injectable treatments, corticosteroids were identified as optimal at 1-month follow-up in one review, whereas another review exploring 6-months follow-up identified intra-articular hyaluronic acid as the best option. Another NMA using a 6-month follow-up identified etoricoxib as the optimal treatment option for pain relief, however, this review did not include injectable therapies. Finally, the NMA that assessed results beyond 1 year found that glucosamine provided optimal pain relief.

**Discussion/Conclusions**: Varying results across NMAs for management of painful knee OA may be attributed to the differences in timeframe assessed, and the treatments considered.

## Pain as Embodied Risk: A Qualitative Thematic Analysis in Childhood Cancer Survivors

Lauren Heathcote http://orcid.org/0000-0003-2515-3102^a,*^, Nele Loecher^a^, Sheri Spunt^b^, Pamela Simon^b^, Sarah Cunningham^a^, Perri Tutelman^c^, Lidia Schapira^d^ and Laura Simons http://orcid.org/0000-0002-3395-9483^a^

^a^Anesthesiology, Perioperative, and Pain Medicine, Stanford University School of Medicine, Palo Alto, California, USA; ^b^Pediatric Oncology, Stanford University School of Medicine, Stanford, California, USA; ^c^Department of Pediatrics, Dalhousie University, Halifax, Nova Scotia, Canada; ^d^Department of Medical Oncology, Stanford University School of Medicine, Stanford, California, USA

**CONTACT** Lauren Heathcote lcheath@stanford.edu

© 2020 The Author(s). Published with license by Taylor & Francis Group, LLC.

This is an Open Access article distributed under the terms of the Creative Commons Attribution License (http://creativecommons.org/licenses/by/4.0/), which permits unrestricted use, distribution, and reproduction in any medium, provided the original work is properly cited.

**Introduction/Aim**: When a child or adolescent lives beyond cancer, they face a lifetime of uncertainty about new or changing physical symptoms. Symptoms such as pain could indicate a normal health event (e.g. muscle ache), a consequence of toxic treatment, or cancer recurrence. The challenge for every survivor is knowing how to monitor, attend to, and interpret everyday experiences of pain. Yet, very little is known about how cancer survivors experience and respond to pain in their everyday lives.

**Methods**: We conducted semi-structured interviews with 25 Adolescent and Young Adult (AYA) cancer survivors (15–25 years old; 45% female, 45% male, 10% non-binary) on their lived experience of perceiving, interpreting, and responding to pain during survivorship. A reflexive thematic analysis was performed.

**Results**: Two overarching themes were generated. The first theme highlighted how pain experiences related to young survivors’ varying sense of **connection with their body**. Some survivors reported feeling in tune with their bodies, while others described a deep sense of disconnection or that their body felt like an adversary. The second theme represented how young survivors **interpreted pain and other signals from their body**, particularly the need to play detective with every new feeling of pain and how this contributed to an overall sense of embodied risk.

**Discussion/Conclusions**: Pain is a complex and often threatening experience after cancer. Assessment and intervention for post-cancer pain must be considered within a biopsychosocial framework, including fears and anxieties that patients hold about post-cancer pain as a signal of bodily threat.

## Adult Observer Evaluations of Children’s Pain: Influence of Child’s Age and Gender

Randa Elgendy^a,*^ and Michelle M. Gagnon^a^

^a^Psychology, University of Saskatchewan, Saskatoon, Saskatchewan, Canada

**CONTACT** Randa Elgendy randa.elgendy@usask.ca

© 2020 The Author(s). Published with license by Taylor & Francis Group, LLC.

This is an Open Access article distributed under the terms of the Creative Commons Attribution-NonCommercial License (http://creativecommons.org/licenses/by-nc/4.0/), which permits unrestricted non-commercial use, distribution, and reproduction in any medium, provided the original work is properly cited.

**Introduction/Aim**: In studies of observer ratings of pain, pain in females and younger children is often perceived as less severe than that of males and older children (Goodenough et al., 1998; Igler et al., 2017). Yet, this research relies on written vignettes, which may not accurately reflect observers’ responses when seeing children experience pain. We examined whether observers’ ratings of children’s pain were influenced by the child’s age and gender when shown videorecordings of children in pain.

**Methods**: Adult participants (*N* = 101, *M*_age_ = 22.69) watched videorecordings of children completing the cold pressor task. Sixteen stimulus videos were shown and represented children across four age and gender groups: girls aged 6–8 and 9–12, boys aged 6–8 and 9–12. Participants rated their perception of the child’s pain severity using a 10-point rating scale. A two-factor, repeated measures ANOVA was conducted to examine the effects of child’s age and gender on observer ratings of pain severity.

**Results**: There was a significant interaction between child’s age and gender (*F*(1, 100) = 179.20, *p* <.000) on perception of pain severity. Pain severity was rated higher for older boys compared to younger boys, but no difference in ratings was observed between older and younger girls. The main effect of child’s age on perception of pain severity was significant (*F*(1, 100) = 309.31, *p* <.000). The main effect for gender was not significant.

**Discussion/Conclusions**: Findings of contextual influences on observer ratings of children’s pain shown through videorecordings are inconsistent with previous research relying on written vignettes. Implications for pain assessment and healthcare are discussed.

## The Voice of the Patient on Subjective Experiences of Diabetic Neuropathic Pain in a Poor Environment

Vincent Adzika^a,*^, John Appiah Poku^b^, Osei Sarfo Kanatanka^c^ and Athena Pedro^a^

^a^Department of Psychology, Faculty of Community and Health Sciences, University of the Western Cape, Cape Town, South Africa; ^b^Department of Behavioral Sciences, School of Medical Sciences, Kwame Nkrumah University of Science and Technology, Kumasi, Ghana; ^c^Department of Medicine, Komfo Anokye Teaching Hospital, Kumasi, Ghana

**CONTACT** Vincent Adzika vin.adzika@gmail.com Department of Psychology, Faculty of Community and Health Sciences, University of the Western Cape, Cape Town, South Africa

© 2020 The Author(s). Published with license by Taylor & Francis Group, LLC.

This is an Open Access article distributed under the terms of the Creative Commons Attribution License (http://creativecommons.org/licenses/by/4.0/), which permits unrestricted use, distribution, and reproduction in any medium, provided the original work is properly cited.

**Introduction/Aim**: Diabetic neuropathy is a major clinical manifestation of diabetes that damages the nerves and affects the peripheral nervous system. The ability to be aware of a patient’s quality of pain and characteristic is instrumental to good diagnosis leading to proper management. However, in most poor environments, patient’s knowledge of neuropathic pain characteristic and experiences are usually silent during diagnosis and management

The study assessed the subjective pain experiences and impact of diabetic neuropathy in an environment where patients lack adequate information on PDN.

**Method**: A qualitative methodological framework focusing on participant’s pain experiences and personal beliefs concerning PDN was used to purposely recruit 30 diabetic patients diagnosed to have neuropathic pain. After ascertaining neuropathy a semi-structured interview guide was used to elicit each response regarding PDN experiences. The data was transcribed and analyzed using a thematic approach to qualitative analysis.

**Results**: The main complaint was sensitivity to touch and intense pain in the foot or fingers. The patients experienced numerous pain characteristics such as burning numbness, tingling, and stabbing in various parts of the body. Other daily symptoms included frequent urination, tummy aches, kneel pain, lower back pain, and chest pain. Patients claimed that these pain characteristics had resulted in a poor quality of health and financial burden.

**Discussion/Conclusions**: The voice of patients in poor environments regarding diabetic neuropathic pain is usually silent during diagnoses but forms the baseline for management. Patient’s claimed that clinicians don’t allow them to discuss pain characteristics and subjective experiences during diagnosis.

## Examining Return-to-Work Outcomes of Workers with or without Comorbid Painful Physical Injuries Admitted to WCB-Alberta’s Traumatic Psychological Injury Programs

Geoffrey S. Rachor http://orcid.org/0000-0002-2850-7220^a,*^, Cary A. Brown^b^, Sebastian Straube^c^, Charl Els^d^, Bruce Dick http://orcid.org/0000-0003-0404-4927^e^, Shelby Yamamoto^f^, Don Voaklander^f^, Tanya Jackson^g^, Sentil Senthilselvan http://orcid.org/0000-0002-6631-1376^f^, Suzette Bremault- Phillips^b^, Theodore Berry^h^, Jarett Stastny^h^, Manuela Joannou^i^ and Douglas P. Gross^j^

^a^Psychology, Faculty of Rehabilitation Medicine, University of Alberta, Edmonton, Alberta, Canada; ^b^Department of Occupational Therapy, University of Alberta, Edmonton, Alberta, Canada; ^c^Division of Preventive Medicine, Department of Medicine, University of Alberta, Edmonton, Alberta, Canada; ^d^Department of Psychiatry, University of Alberta, Edmonton, Alberta, Canada; ^e^Department of Anesthesiology & Pain Medicine, University of Alberta, Edmonton, Alberta, Canada; ^f^University of Alberta, School of Public Health, Edmonton, Alberta, Canada; ^g^Department of Medicine, University of Alberta, Edmonton, Alberta, Canada; ^h^Workers’ Compensation Board of Alberta, Millard Health, Edmonton, Alberta, Canada; ^i^Project Trauma Support, Perth, Ontario, Canada; ^j^Department of Physical Therapy, University of Alberta, Edmonton, Alberta, Canada

**CONTACT** Geoffrey S. Rachor geoffrey.rachor@ualberta.ca

© 2020 The Author(s). Published with license by Taylor & Francis Group, LLC.

This is an Open Access article distributed under the terms of the Creative Commons Attribution License (http://creativecommons.org/licenses/by/4.0/), which permits unrestricted use, distribution, and reproduction in any medium, provided the original work is properly cited.

**Introduction/Aim**: The Workers’ Compensation Board of Alberta (WCB-Alberta) offers rehabilitation for claimants with Traumatic Psychological Injuries (TPI) aimed at facilitating return-to-work. This study examines the effects of having comorbid painful physical injuries on return-to-work of claimants undergoing TPI rehabilitation.

**Methods**: We conducted a secondary analysis of administrative data collected on injured claimants with TPI admitted to WCB-Alberta’s TPI programs between the years 2014– 2016. Claimants were categorized into two sub-groups. Group 1 (*n* = 313) included claimants with TPI only, and Group 2 (*n* = 180) included claimants diagnosed with comorbid painful physical injuries. Variables included age, sex, occupation, duration of injury, and return-to- work status at discharge. Chi-squared tests examined differences in return-to-work outcomes.

**Results**: Claimants were primarily male (44%) with a mean age of 41 (± 11) years in Group 1 and primarily male (52%) with a mean age of 40 (± 12) years in Group 2. Group 1 were primarily public safety personnel (32%) exposed to a psychologically traumatic event in the workplace, while Group 2 were primarily workers in the trades (51%) involved in transport accidents. Mean duration of injury in Groups 1 and 2 were 198 (± 558) and 149 (± 299) days, respectively. Successful return-to-work across Groups 1 and 2 was achieved for 52% and 25% of claimants, respectively (*p* < .001).

**Discussion/Conclusions**: Findings demonstrate that return-to-work is significantly lower for workers with TPI and comorbid painful physical injuries. Identifying comorbidities and best practices in rehabilitation for claimants undergoing TPI rehabilitation is needed to optimize return-to-work.

## Interventional Pain Management for Chronic Pain: A Survey of Canadian Physicians

Harsha Shanthanna^a,*^, Anuj Bhatia^b^, Mohan Radhakrishna^c^, Emilie Belley-Cote^d^, Thuva Vanniyasingam^a^, Lehana Thabane^d^ and Jason W. Busse http://orcid.org/0000-0002-0178-8712^e,f^

^a^Department of Anesthesia, McMaster University, Hamilton, Ontario, Canada; ^b^Department of Anesthesia and Pain Medicine, Toronto Western Hospital, Toronto, Ontario, Canada; ^c^Department of Physical Medicine and Rehabilitation, McGill University, Montreal, Quebec, Canada; ^d^Department of Health Research Methods, Evidence and Impact, McMaster University, Hamilton, Ontario, Canada; ^e^Departments of Anesthesia and Health Research Methods, Evidence and Impact (HEI), McMaster University, Hamilton, Ontario, Canada; ^f^Michael G. DeGroote Centre for Medicinal Cannabis Research, Michael G. DeGroote Institute for Pain Research and Care, Hamilton, Ontario, Canada

**CONTACT** Harsha Shanthanna harshamd@gmail.com

© 2020 The Author(s). Published with license by Taylor & Francis Group, LLC.

This is an Open Access article distributed under the terms of the Creative Commons Attribution License (http://creativecommons.org/licenses/by/4.0/), which permits unrestricted use, distribution, and reproduction in any medium, provided the original work is properly cited.

**Introduction/Aim**: The use of interventional pain management (IPM) modalities to alleviate chronic pain is increasing despite the lack of high-quality evidence. We undertook a survey to explore practice patterns, training, and attributes of IPM practice in Canada.

**Methods**: We administered a 32-item survey via seven Canadian physician member organizations, whose members were engaged in the management of chronic pain.

**Results**: Of 777 physicians contacted, 256 (33%) responded. One hundred and sixty-nine of 194 (87%) practiced IPM and 103 of 194 (53%) managed only non-cancer pain. Pain management training of ≥6 months was associated with higher odds of IPM training (odds ratio [OR] 2.98, 95% confidence interval [CI] 1.32 to 6.7), but not necessarily ongoing IPM practice (OR 1.97, 95%CI 0.74 to 5.3). A substantial percentage of physicians (108 of 168; 64%) practiced IPM based only on training received either during their base residency program or courses. Only 48 of 186 (26%) felt that there were adequate opportunities for IPM training, and 69 of 186 (37%) believed that their colleagues practiced IPM in accordance with the best current evidence.

**Discussion/Conclusions**: Our survey indicates that IPM practice and training was not uniform, and that interventional therapies for chronic pain may not be performed in accordance with the best available evidence. Our survey highlights a lack of IPM training opportunities, which may result in substandard training. Concerted efforts involving physician organizations and regulators are needed to standardize IPM training and develop clinical guidelines to optimize evidence-based practice.

## Patterns of Recreational and Medical Cannabis Use in a Large Community Sample of Cannabis Users

Jasmine Turna http://orcid.org/0000-0003-0118-9039^a,b,*^, Iris Balodis^a,b^, Catharine Munn^a^, Michael Van Ameringen^a^, Jason Busse http://orcid.org/0000-0002-0178-8712^a,c,d^ and James MacKillop^a,b^

^a^Michael G. DeGroote Centre for Medicinal Cannabis Research, McMaster University, Hamilton, Ontario, Canada; ^b^Peter Boris Centre for Addictions Research, McMaster University, Hamilton, Ontario, Canada; ^c^Departments of Anesthesia and Health Research Methods, Evidence and Impact (HEI), McMaster University, Hamilton, Ontario, Canada; ^d^Michael G. DeGroote Institute for Pain Research and Care, Hamilton, Ontario, Canada

**CONTACT** Jasmine Turna turnaj@mcmaster.ca

© 2020 The Author(s). Published with license by Taylor & Francis Group, LLC.

This is an Open Access article distributed under the terms of the Creative Commons Attribution License (http://creativecommons.org/licenses/by/4.0/), which permits unrestricted use, distribution, and reproduction in any medium, provided the original work is properly cited.

**Introduction/Aim**: Regulatory changes are increasing access to medical cannabis and cannabis in general. As such, understanding patterns of recreational and medical cannabis use is a high public health priority. We characterized patterns of cannabis use (recreational and medical), other substance use, and psychiatric symptoms in a large sample of adult cannabis users in Canada, prior to federal cannabis legalization.

**Methods**: This was a self-report assessment of 709 cannabis users (mean age = 30.19 (11.82) years; 55.01% female). Patterns of overall substance use and psychiatric symptomatology was compared based on recreational/medical cannabis status were examined.

**Results**: Overall, 61% of participants endorsed recreational use while 39% reported medical use. The most common therapeutic reason for use was pain relief, and a third of medical users reported reduced use of opioids as a result. Of medical users, only 23% reported authorization from a health professional. Recreational cannabis users typically reported infrequent use (less than weekly), whereas medical users mostly reported daily use. Compared to recreational users, medical users reported more problematic cannabis use in addition to greater psychiatric symptomatology (anxiety, depression and trauma). Interestingly, a large majority of medical users also reported using recreationally (81%), while exclusive medical use was less common (19%). This dual motives group reported more daily cannabis use and more alcohol and tobacco use.

**Discussion/Conclusions**: These findings reveal the differences in cannabis use patterns and preferences between recreational and medical users, and suggest that many medical users of cannabis may reduce use of opioids as a result.

## The Possible Role of Pelvic Pain in Hystero-epilepsy

John Jarrell^a,*^ and Frank Stahnisch^b^

^a^Department of Obstetrics and Gynecology, University of Calgary, Calgary, Alberta, Canada; ^b^Hannah Professor, History of Medicine and Health Care, University of Calgary, Calgary, Alberta, Canada

**CONTACT** John Jarrell john.jarrell@ahs.ca

© 2020 The Author(s). Published with license by Taylor & Francis Group, LLC.

This is an Open Access article distributed under the terms of the Creative Commons Attribution License (http://creativecommons.org/licenses/by/4.0/), which permits unrestricted use, distribution, and reproduction in any medium, provided the original work is properly cited.

**Introduction/Aim**: In 1881, 218 cases of removing “normal ovaries” for menstrual distress was reported by 42 surgeons where hystero-epilepsy was the indication in 12%. At the same time Jean-Martin Charcot (1825–1893) thought this term represented a neurological disease where hysterical attacks could be initiated or terminated by pressure on ovarian zones. The aim of this study is to clarify hystero-epilepsy.

**Methods**: An extensive literature review was conducted classifying “hystero-epilepsy” and “ovarian epilepsy” from Pubmed and Pubmed Central as: Charcot – related phenomena no ovarian symptoms; Charcot – related phenomena with ovarian symptoms, gynecological – related pain and tenderness, nervous system disease and inability to categorize. Males and children were excluded.

**Results**: A total of 206 citations were hand searched. There were 28 citations associated with gynecological pain (1877–1910), 38 citations with Charcot related phenomena with no ovarian symptoms (1877–1903) and 16 with ovarian symptoms (1877–1898), 29 associated with nervous system disease (1877–1908), 57 unable to categorize, 25 exclusions and 13 repeat reports.

**Discussion/Conclusions**: Gynecological interpretation of hystero-epilepsy was consistently reported in relation to menstrual-related disorders. Charcot – related phenomena were complicated by association with and without ovarian zones. The possibility that women with Charcot – related ovarian zones were suffering from chronic pelvic pain is supported by the development of two devices worn to eliminate the attacks by compressing the ovarian regions of the lower abdomen. Charcot’s understanding was eventually challenged as either fakery or a psychological disorder. This report re-opens the late nineteenth century theory suggesting pelvic disease as an alternative explanation.

## Persistent Neuropathic Pain after Breast Cancer Surgery: A Systematic Review and Meta-analysis

Li Wang http://orcid.org/0000-0003-1585-8846^a,*^, Allen Li^b^, Jared Cohen^b^, Daniel Lu^b^, Niveditha Devasenapathy^c^, Sasha Kheyson^d^, Yvgeniy Oparin^b^, Kate Jackson^b^, Giuliana Guarna^b^, Rachel Couban^a^ and Jason W. Busse http://orcid.org/0000-0002-0178-8712^a,e^

^a^Department of Anesthesia, McMaster University, Hamilton, ON, Canada; ^b^Michael G. DeGroote School of Medicine, McMaster University, Hamilton, ON, Canada; ^c^Department of Family Medicine, Cumming School of Medicine, University of Calgary, Calgary, Alberta, Canada; ^d^Indian Institute of Public Health -delhi, Public Health Foundation of India, Gurgaon, Haryana, India; ^e^Departments of Health Research Methods, Evidence and Impact (HEI), McMaster University, Hamilton, Ontario, Canada

**CONTACT** Li Wang lwang246@gmail.com

© 2020 The Author(s). Published with license by Taylor & Francis Group, LLC.

This is an Open Access article distributed under the terms of the Creative Commons Attribution License (http://creativecommons.org/licenses/by/4.0/), which permits unrestricted use, distribution, and reproduction in any medium, provided the original work is properly cited.

**Introduction/Aim:** Persistent neuropathic pain is a common complication after breast cancer surgery, ranging from 10% to 70%. We conducted a systematic review to address this uncertainty.

**Methods:** We searched MEDLINE, EMBASE, CINAHL and PsycINFO from inception to November 2018, to identify observational studies that reported persistent neuropathic pain after breast cancer surgery. We performed random effects meta-analysis with Freeman-Tukey transformation for prevalence, and pooled pain intensity after converting all pain scales to a 10 cm visual analogue scale (VAS). The GRADE approach was used to rate the quality of evidence.

**Results:** We included 31 observational studies with 9,263 patients. Moderate quality evidence showed the prevalence of persistent neuropathic pain was 29% (95%CI 23% to 35%). No subgroup effects were found whether neuropathic pain was measured by validated instruments (27%, 95% CI 21% to 34%) or by clinical assessment (29%, 95% CI 21% to 37%; test of interaction *p* = .47).

Ten studies (901 patients) reported persistent neuropathic pain intensity among patients with the pooled score of 3.8 cm (95%CI 3.1 to 4.5 cm on a 10 cm VAS), indicating 49% of these patients suffering moderate-to-severe pain (≥4 cm on a 10 cm VAS). We did not find significant subgroup effects between validated instruments (3.4 cm, 95%CI 1.8 to 4.9 cm) vs. clinical assessment (4.0 cm, 95%CI 3.2 to 4.9 cm, test of interaction *p* = .38)

**Discussion/Conclusions:** Persistent neuropathic after breast surgery is common, affecting approximately 1 in 3 women undergoing this procedure, and half who develop persistent complaints experience moderate to severe pain.

**Figure:**
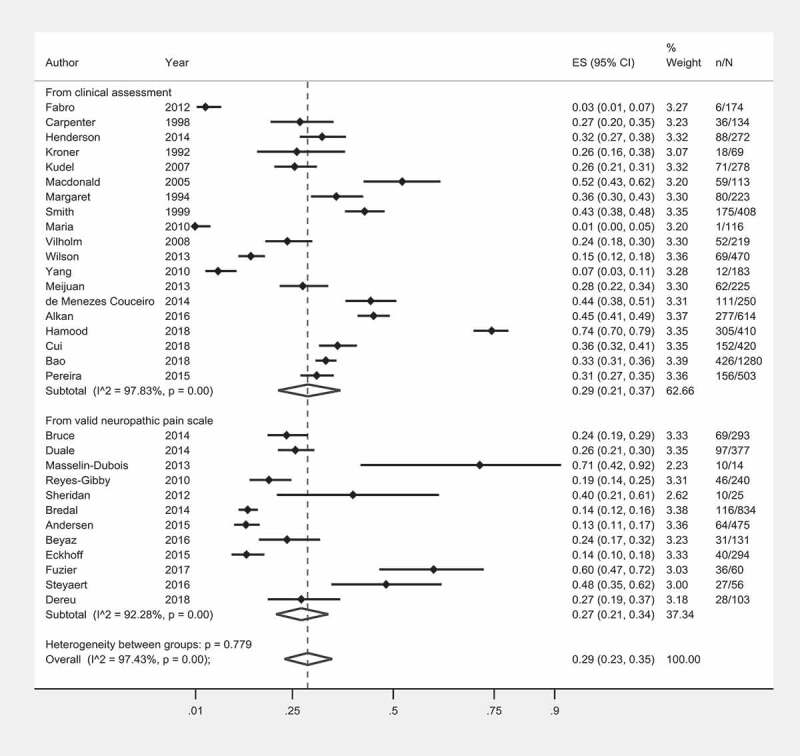

**Figure:**
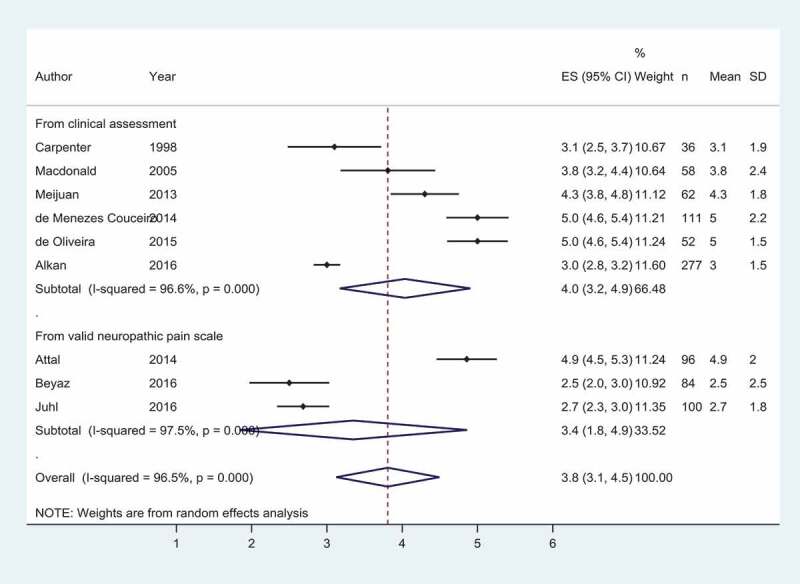

Prevalence of persistent neuropathic pain after breast cancer surgery

Intensity of persistent neuropathic pain after breast cancer surgery

## Pain in Inflammatory Bowel Disease

Valentina Mihajlovic http://orcid.org/0000-0002-8831-4316^a,*^ and Dean Tripp^b^

^a^Psychology, Queen’s University, Kingston, Ontario, Canada; ^b^Psychology, Anesthesiology, and Urology, Queen’s University, Kingston, Ontario, Canada

**CONTACT** Valentina Mihajlovic valentina.mihajlovic@queensu.ca

© 2020 The Author(s). Published with license by Taylor & Francis Group, LLC.

This is an Open Access article distributed under the terms of the Creative Commons Attribution License (http://creativecommons.org/licenses/by/4.0/), which permits unrestricted use, distribution, and reproduction in any medium, provided the original work is properly cited.

**Introduction/Aim**: Pain is a common experience and primary concern for individuals with Inflammatory Bowel Disease (IBD). Abdominal pain and extra-intestinal symptoms contribute to the overall pain experience of individuals with IBD and negatively affect quality of life. Our aim was to outline pain characteristics and the impact of pain on quality of life in a worldwide sample of individuals with IBD.

**Methods**: 375 adults (74% female, *M*_age_ = 40 years) with IBD completed a series of validated questionnaires online to assess pain course (painDETECT), pain localization and severity (pain body diagram), IBD activity (IBD Symptom Inventory Short Form), shame (Chronic Illness-Related Shame Scale), and depressive symptoms (Patient Health Questionnaire Nine).

**Results**: A majority of the sample reported experiencing some pain over the course of one month: 15.7% of participants reported pain attacks with pain in-between, 25.1% reported pain attacks without pain in-between, 21.8% reported persistent pain with pain attacks, and 26% reported persistent pain with slight fluctuations. 11.5% of participants reported no pain. Abdominal (52%) and back (32%) pain were the main pain localizations. Pain was significantly associated with greater disease activity, *b* = 4.8403, SE = .4989, *p* < .001, shame, *b* = 1.0438, SE = .2477, *p* < .001, and depressive symptoms, *b* = 1.1852, SE = .1847, *p* < .001.

**Discussion/Conclusions**: Pain is highly prevalent in IBD and has a substantial impact on quality of life. Results from this study underscore the importance of pain management in IBD.

## Examining the Utility of the Parent Risk and Impact Screening Measure (PRISM) in Parents of Youth with Acute Musculoskeletal Pain

Wendy Gaultney^a,*^, Hayley Turner^a^, Anna Wilson^a^ and Amy Holley^a^

^a^Department of Pediatrics, Oregon Health & Science University, Portland, Oregon, USA

**CONTACT** Wendy Gaultney gaultnew@ohsu.edu

© 2020 The Author(s). Published with license by Taylor & Francis Group, LLC.

This is an Open Access article distributed under the terms of the Creative Commons Attribution License (http://creativecommons.org/licenses/by/4.0/), which permits unrestricted use, distribution, and reproduction in any medium, provided the original work is properly cited.

**Introduction/Aim**: The Pediatric Risk and Impact Screening Measure (PRISM) screens parents of youth with chronic pain for emotional, cognitive and behavioral responses that may affect child pain and function. The current study examines the utility of the PRISM for screening parents of youth with acute musculoskeletal (MSK) pain, examining associations among PRISM scores, parent factors, youth pain and activity limitations.

**Methods**: Participants are 66 youth ages 11–-17 (*M* = 14.51, 41% female) with acute MSK pain, and their parent (77% female), from a longitudinal study examining youth pain outcomes. Dyads were recruited from emergency departments and outpatient clinics. Parents completed the PRISM which yields low (scores 0–-5) and moderate/high (scores 6–-12) risk groups. Parents also reported on protective behaviors, distress tolerance, and catastrophizing about their child’s pain. Youth reported on pain intensity, frequency and activity limitations. T-tests and chi-squares tested differences between PRISM risk groups.

**Results**: PRISM scores classified 9.1% of parents (n = 6) as moderate/high-risk and 90.9% low-risk (n = 60). PRISM risk group did not differ by youth or parent demographic factors. The moderate/high-risk group reported higher protectiveness (*t* = −4.39, *p* < .001), less tolerance of and higher catastrophizing about child pain (*t* = 2.43, *p* = .02; *t* = −5.23, *p* < .001). Youth activity limitations and pain were not different by risk group.

**Discussion/Conclusions**: The PRISM can rapidly screen parents of youth with acute MSK pain to identify those who may need additional supports. Scores may inform interventions targeting parent behaviors and distress. Longitudinal data will examine if youth of parents identified as high risk have poorer outcomes including development of chronic pain.

## Metacognitions about Health May Moderate the Relationship between Pain Catastrophizing and Pain-Related Disability

Geoffrey S. Rachor^a,*^ and Alexander M. Penney^b^

^a^Faculty of Rehabilitation Medicine, University of Alberta, Edmonton, Alberta, Canada; ^b^Department of Psychology, MacEwan University, Edmonton, Alberta, Canada

**CONTACT** Geoffrey S. Rachor grachor@ualberta.ca

© 2020 The Author(s). Published with license by Taylor & Francis Group, LLC.

This is an Open Access article distributed under the terms of the Creative Commons Attribution License (http://creativecommons.org/licenses/by/4.0/), which permits unrestricted use, distribution, and reproduction in any medium, provided the original work is properly cited.

**Introduction/Aim**: Metacognitions have been associated with chronic pain, including pain intensity and pain-related disability, and with pain catastrophizing. Researchers have hypothesized that metacognitions may moderate the relationship between pain catastrophizing and pain-related outcomes, however, this has not yet been tested. The current study aimed to examine whether metacognitions about health moderate the relationship between pain catastrophizing and pain-related disability, controlling for pain severity.

**Methods**: University students with self-reported chronic pain (*N* = 280) completed the Brief Pain Inventory – Short Form, measuring pain severity and pain interference, the Pain Catastrophizing Scale (PCS), and the Metacognitions about Health Questionnaire (MCQ-HA), containing three subscales: 1) Metacognitive Beliefs about Biased Thinking (MCQ-HAB), 2) That Thoughts can Cause Illness, and 3) That Thoughts are Uncontrollable. Preliminary regression analyses were conducted to determine the appropriateness of variables for inclusion in the moderation model. Moderation analysis was then conducted using the PROCESS macro for SPSS.

**Results**: Preliminary regression analyses indicated that, while controlling for pain severity, PCS scores predicted pain interference, and that MCQ-HAB was the only MCQ-HA subscale to predict pain interference. Moderation analysis was then conducted with pain interference as the dependent variable, PCS as the independent variable, and MCQ-HAB as the moderator, controlling for pain severity. Results indicated significant effects of both PCS (β=.08, *p* =.003) and MCQ-HAB (β=.31, *p* =.012) on pain interference, as well as a significant interaction effect of PCS and MCQ-HAB (β= −.01, *p* =.048), where higher PCS scores were associated with greater pain interference at low, but not high, levels of MCQ-HAB.

**Discussion/Conclusions**: Findings suggest that metacognitive beliefs about biased thinking may moderate the relationship between pain catastrophizing and pain-related disability.

## The Ehlers-Danlos Syndrome (EDS) Expert Panel’s Criteria for Neurosurgical Intervention: A Critical Appraisal Using the AGREE II

Lindsay Wilson^a,*^ and Adena Gutstein^b^

^a^Department of Brain and Spinal Cord Injury Rehabilitation, Toronto Rehabilitation Institute, University Health Network, Toronto, ON, Canada; ^b^Department of Family Medicine, North York Family Health Team, Toronto, ON, Canada

**CONTACT** Lindsay Wilson lwilson6@lakeheadu.ca Department of Brain and Spinal Cord Injury Rehabilitation, Toronto Rehabilitation InstituteUniversity Health Network, Toronto, ON, Canada

© 2020 The Author(s). Published with license by Taylor & Francis Group, LLC.

This is an Open Access article distributed under the terms of the Creative Commons Attribution License (http://creativecommons.org/licenses/by/4.0/), which permits unrestricted use, distribution, and reproduction in any medium, provided the original work is properly cited.

**Introduction/Aims**: In 2015, the EDS Expert Panel proposed criteria for the neurosurgical management of defects involving the spine and craniovertebral junction; examples include cervical instability, Chiari Malformation, Basilar Impression, and Syringomyelia. A critical appraisal using the AGREE II was conducted to determine whether criteria were sufficient to establish best practice guidelines.

**Methods**: The AGREE II includes 23 questions scored on a 7-point scale and divided into the following six domains: purpose, stakeholders, rigor, clarity, applicability, and editorial independence. Three independent reviewers appraised criteria, and individual scores were averaged across each domain.

**Results**: Overall, criteria achieved a score of 43.5%, which is insufficient to establish guidelines. Criteria had three main limitations: first, recommendations were not based on a systematic review of the literature, and methods of expert consensus were not described. Second, the Panel did not address the main reasons why existing criteria require clarification, which are largely to do with the lack of consensus surrounding the utility of dynamic imaging, choice in craniometrics and pathological thresholds, and the role of neurophysiological testing in evaluating clinical manifestations. Third, the risks/benefits of the procedure were not discussed, and the final recommendation included vague qualifiers such as “possible brainstem compression” and “significant instability … or deficits to warrant a fusion.”

**Discussion/Conclusion**: Objective indications for the management of complications involving the hypermobile spine remain to be determined. Progress hinges on a more rigorous approach to synthesizing the literature, and future criteria must include explicit detail about the radiological and clinical modalities/metrics used in the diagnostic workup.

## Transitioning to Practice and Managing Pain: An Exploratory Study of New Graduate Nurses Experiences

Monakshi Sawhney http://orcid.org/0000-0001-5399-1715^a,*^, Eloise Carr^b^ and Elina Gama Fila^c^

^a^School of Nursing, Queens University, Kingston, Ontario, Canada; ^b^Faculty of Nursing, University of Calgary, Calgary, Alberta, Canada; ^c^Department of Psychology, York University, Toronto, Ontario, Canada

**CONTACT** Monakshi Sawhney mona.sawhney@queensu.ca

© 2020 The Author(s). Published with license by Taylor & Francis Group, LLC.

This is an Open Access article distributed under the terms of the Creative Commons Attribution License (http://creativecommons.org/licenses/by/4.0/), which permits unrestricted use, distribution, and reproduction in any medium, provided the original work is properly cited.

**Introduction/Aim**: Nurses learn to assess and manage pain during their undergraduate education and they refine their pain management skills after graduation. However, little is known about nurse’s experience in managing pain as they transition to practice. The aim of this study was to explore the experiences of new graduate registered nurses (NG-RNs) managing pain as they transition to practice from the educational setting to the workplace.

**Methods**: A qualitative descriptive design was used to gain an understanding of the NG-RNs experience. NG-RNs (n = 15) completed a telephone interview up to 12 weeks after graduation. They were asked the following questions: What aspects of managing pain do undergraduate nursing students find stressful?; How confident do you feel managing patients’ pain now?

**Results**: Participants reported three salient issues that made managing pain as a nursing student stressful: dependence on other healthcare providers, lack of experience, and fear of harm. The experiences described reflected a sense of helplessness when trying to manage pain as a student. To reduce this stress, they identified consulting with clinical faculty, using their theoretical knowledge, and non-pharmacological methods to manage pain. Eleven participants reported a moderate level of confidence in managing pain post-graduation. They identified needing more clinical experience, and not being confident in managing chronic pain, or acute on chronic pain.

**Discussion/Conclusions**: The findings of this study provide some insight into the experience of NG-RNs managing pain. Providing nursing students with clinical and theoretical experiences to improve their confidence in managing pain may ease the transition to independent nursing practice.

## Systematic Review of Physical Illnesses and Psychological Features as Risk Factors for Back Pain from Childhood to Young Adulthood

Amber Beynon^a,*^, Jeffrey Hebert^b^, Christopher Hodgetts^a^, Leah Boulos^c^ and Bruce Walker^a^

^a^College of Science, Health, Engineering and Education, Murdoch University, Murdoch, Australia; ^b^Faculty of Kinesiology, University of New Brunswick, Fredericton, Canada; ^c^Research Services, Maritime SPOR SUPPORT Unit, Halifax, Nova Scotia, Canada

**CONTACT** Amber Beynon amber.beynon@murdoch.edu.au

© 2020 The Author(s). Published with license by Taylor & Francis Group, LLC.

This is an Open Access article distributed under the terms of the Creative Commons Attribution License (http://creativecommons.org/licenses/by/4.0/), which permits unrestricted use, distribution, and reproduction in any medium, provided the original work is properly cited.

**Introduction/Aim:** Low back pain etiology is complex and has many contributing variables. The purpose of this systematic review was to report evidence of chronic physical illnesses, mental health disorders, and psychological features as potential risk factors for back pain in young people.

**Methods:** This systematic review and meta-analysis included cohort and inception cohort studies that investigated potential risk factors for back pain in children, adolescents, and young adults. Potential risk factors of interest were chronic physical illnesses, mental health disorders, or other psychological features. Searches were conducted in MEDLINE, Embase, CINAHL, and Scopus from inception to June 2018.

**Results:** Nineteen of 2653 screened articles were included in the qualitative synthesis, and data from 12 articles were included in the meta-analysis. Evidence from inception cohort studies demonstrated the most likely risk factors for back pain are psychological distress, as well as psychological features including emotional coping problems and somatosensory amplification. Evidence from non-inception cohort studies cannot distinguish between potential risk factors or triggers for back pain. However, we identified several additional factors that are associated with back pain. Specifically, asthma, headaches, abdominal pain, depression, anxiety, conduct problems, somatization, and “feeling tense” are potential risk factors/triggers for back pain.

**Discussion/Conclusions**: The most likely risk factors for back pain are psychological features. Due to the limitations of the literature we still cannot be certain if physical illnesses, mental health disorders, and psychological features are comorbidities, triggers, or risk factors for back pain. More high-quality research is needed to better elucidate these relationships.

## Opiate Prescribing in Canada Following the Legalization of Cannabis: A Clinical and Economic Time Series Analysis

George Dranitsaris^a,*^, Carlo DeAngelis^b^, Blake Pearson^c^, Laura McDermott^d^ and Bernd Pohlmann-Eden^d^

^a^Augmentium Pharma Consulting Inc, Toronto, Ontario, Canada; ^b^Odette Cancer Centre, Toronto, Ontario, Canada; ^c^Opioid Reduction Strategy, Erie St. Clair Local Health Integration Network, Chatham, Ontario, Canada; ^d^Scientus Pharma Inc., Toronto, Ontario, Canada

**CONTACT** George Dranitsaris george@augmentium.com

© 2020 The Author(s). Published with license by Taylor & Francis Group, LLC.

This is an Open Access article distributed under the terms of the Creative Commons Attribution License (http://creativecommons.org/licenses/by/4.0/), which permits unrestricted use, distribution, and reproduction in any medium, provided the original work is properly cited.

**Background:** The purpose of this study was to assess time trends in the amount and total cost of opiate prescribing in Canada prior to and following the legalization of cannabis.

**Methods:** Canada wide monthly claims data for public and private payers were obtained from IQVIA PharmaStat for January 2016 – June 2019. The drug products evaluated consisted of morphine, codeine, fentanyl, hydrocodone, hydromorphone, meperidine, oxycodone and tramadol. All opiate volumes were converted to a mean morphine equivalent dose (MED) per claim. Time series regression modeling was undertaken with dependent variables being mean MED per claim and total monthly spending. The slopes of the time series curves were compared post vs. pre cannabis legalization.

**Results:** Over the 42-month time horizon, there was a steady and statistically significant decline in the mean MED per claim within public drug plans. However, when comparing post vs. pre legalization, the decline in MED was 5.4 times greater in the former time period (22.3 vs. 4.1 mg per claim every month). In addition, total monthly opiate spending reductions averaged $95,000 per month before October 2018 compared to $267,000 per month following the legalization of cannabis. Similar findings were also observed within private payer plans.

**Conclusions**: It appears that the legalization of cannabis coincided with a marked drop in opiate usage in Canada. Consistent with reports from other studies, our findings support the hypothesis that easier access to cannabis for pain may reduce opiate usage and save drug costs for both public and private drug plans.

## Appropriateness of Imaging for Axial Pain: A Systematic Review

Mostafa Alabousi http://orcid.org/0000-0003-4023-0559^a,*^, John J. Riva^b^, Eden Liu^c^, Sheniz Eryuzlu^d^, Allison Chan^e^, Leen Naji^f^, Ronelle Calver^e^, Senthujan Gunaseelan^a^, John You^g^, Rachel Couban^h^, Gordon H. Guyatt^i^, Paul E. Alexander^h^, Amane Abdul-Razzak^j^, John A Dufton^k^, Regina Li^l^, Yoan K. Kagoma^m^, Madison Y. Zhang^h^, Yoga Raja Rampersaud^n^, Michael J. Goytan^o^, Nancy S. Lloyd^l^, Brie DeMone^p^, Thomas E. Feasby^q^, Martin Reed^r^ and Jason W. Busse http://orcid.org/0000-0002-0178-8712^s^

^a^Department of Radiology, McMaster University, Hamilton, Ontario, Canada; ^b^Departments of Health Research Methods and Family Medicine, McMaster University, Hamilton, Ontario, Canada; ^c^Department of Medicine, Western University, London, Ontario, Canada; ^d^Department of Family Medicine, University of Toronto, Toronto, Ontario, Canada; ^e^Department of Physical Medicine and Rehabilitation, McMaster University, Hamilton, Ontario, Canada; ^f^Department of Family Medicine, McMaster University, Hamilton, Ontario, Canada; ^g^Departments of Medicine and Health Research Methods, Evidence, and Impact, McMaster University, Hamilton, Ontario, Canada; ^h^Department of Health Research Methods, Evidence, and Impact, McMaster University, Hamilton, Ontario, Canada; ^i^Department of Clinical Epidemiology and Biostatistics, McMaster University, Hamilton, Ontario, Canada; ^j^Department of Oncology, University of Calgary, Calgary, Alberta, Canada; ^k^Department of Radiology, University of British Columbia, Vancouver, British Columbia, Canada; ^l^Department of Medicine, McMaster University, Hamilton, Ontario, Canada; ^m^Department of Radiology, McMaster University and Juravinski Hospital, Hamilton, Ontario, Canada; ^n^Department of Orthopedic Surgery, University of Toronto, Toronto, Ontario, Canada; ^o^Department of Surgery, University of Manitoba, Winnipeg, Manitoba, Canada; ^p^Department of Seniors and Active Living, Manitoba Health, Winnipeg, Manitoba, Canada; ^q^Department of Neurology, University of Calgary, Calgary, Alberta, Canada; ^r^Department of Radiology, University of Manitoba, Winnipeg, Manitoba, Canada; ^s^Departments of Anesthesia and Health Research Methods, Evidence, and Impact, McMaster University, Hamilton, Ontario, Canada

**CONTACT** Mostafa Alabousi mostafa.alabousi@medportal.ca Department of Radiology, McMaster University, Hamilton, Ontario, Canada

© 2020 The Author(s). Published with license by Taylor & Francis Group, LLC.

This is an Open Access article distributed under the terms of the Creative Commons Attribution License (http://creativecommons.org/licenses/by/4.0/), which permits unrestricted use, distribution, and reproduction in any medium, provided the original work is properly cited.

**Introduction/Aim**: Up to 80% of adults will experience spine related complaints, which may result in diagnostic imaging, including plain x-rays, computed tomography, and magnetic resonance imaging. We performed a systematic review to explore the rates of inappropriate spine imaging, as well as the effectiveness of interventions to decrease inappropriate imaging.

**Methods**: We searched MEDLINE, HealthStar, EMBASE, CINAHL, Index to Chiropractic Literature, and The International Guideline Library from inception until July 8, 2018. Eligible studies reported on the proportion of inappropriate spine-related imaging or tested a strategy to improve the appropriateness of spine-related imaging. Articles underwent title, abstract, and full-text screening, followed by data extraction in duplicate.

**Results**: Of 17,527 unique citations, we reviewed 483 full text studies. We identified 62 eligible studies: 35 studies explored rates of inappropriate spine-related imaging, while 27 studies explored interventions to reduce inappropriate imaging of axial complaints. Inappropriate rates of cervical spine imaging ranged from 3%–54%, most frequently based on the National Emergency XRadiography Utilization Study and American College of Radiology criteria. Inappropriate rates of lumbar spine imaging ranged from 13%–80%, most frequently based on the U.S. Agency for Health Care Policy and Research guidelines. Interventions to improve appropriate imaging included guideline dissemination, educational interventions, and decision support systems, reducing imaging rates between 0%–80%, with active strategies implemented at the point-of-care showing larger effects.

**Discussion/Conclusions**: Inappropriate spine imaging is an ongoing issue. Active decision aids reduced inappropriate imaging rates more than passive dissemination of educational material.

## Parent Sleep Quality as a Potential Mechanism in the Relationship between Parent PTSD Symptoms and Child Chronic Pain Outcomes

Tessa Wihak^a,*^, Maria Pavlova^a^ and Melanie Noel http://orcid.org/0000-0003-3752-8055^a^

^a^Psychology, University of Calgary, Calgary, Alberta, Canada

**CONTACT** Tessa Wihak tessa.wihak@ucalgary.ca

© 2020 The Author(s). Published with license by Taylor & Francis Group, LLC.

This is an Open Access article distributed under the terms of the Creative Commons Attribution License (http://creativecommons.org/licenses/by/4.0/), which permits unrestricted use, distribution, and reproduction in any medium, provided the original work is properly cited.

**Introduction/Aim:** Clinically elevated levels of post-traumatic stress disorder (PTSD) symptoms have been documented in parents of youth with chronic pain and are related to worse child pain intensity. Child factors including catastrophic thinking about pain have been shown to mediate this relationship. However, less is known about the role of PTSD-related parent factors, such as a poor sleep quality, in this pathway. The current study is the first to examine parent sleep quality as a potential mediator in the parent PTSD-child pain relationship among youth with chronic pain.

**Methods:** Fifty-one youth with chronic pain and one of their parents (*M* = 45 years; 91% mothers) were recruited from a tertiary-level pain management program. Parents completed questionnaires assessing sleep quality and PTSD symptoms and youth reported on their pain intensity at baseline. Correlation and mediation analyses were conducted to examine the relationship between parent variables and youth pain outcomes.

**Results:** Poor parent sleep quality was associated with higher levels of PTSD symptoms (*r* =.43, *p* <.01) and higher child pain intensity (*r* =.54, *p* <.001). Higher PTSD was correlated with higher child pain intensity (*r* =.37, *p* <.001). Parent sleep quality mediated the relationship between parent PTSD and child pain intensity (*n* = 51, *ab* = 0.05, CI_BCa _= 0.0047 to 0.13).

**Discussion/Conclusions:** Findings suggest that parent reported sleep quality may explain how parent PTSD symptoms influence child pain outcomes. Prospective research using objective measures to measure parent sleep quality is needed.

## Injustice Experience, a Yellow Flag in Chronic Pain Management

Hadi Shojaei^a,*^, Delaram Shojaei (Presenting Author)^b,c^ and Arman Shojaei (Presenting Author)^c,d^

^a^Northern Ontario School of Medicine, Lakehead University, Thunder Bay, Ontario, Canada; ^b^Faculty of Medical Science, Western University, London, Ontario, Canada; ^c^Faculty of Science, University of British Colombia, Vancouver, British Columbia, Canada; ^d^Faculty of Science, York University, Toronto, Ontario, Canada

**CONTACT** Hadi Shojaei shojaeih@tbh.net

© 2020 The Author(s). Published with license by Taylor & Francis Group, LLC.

This is an Open Access article distributed under the terms of the Creative Commons Attribution License (http://creativecommons.org/licenses/by/4.0/), which permits unrestricted use, distribution, and reproduction in any medium, provided the original work is properly cited.

Chronic pain is defined as pain without apparent biological value that has persisted beyond the normal tissue healing time (IASP, 2012). One of four Canadians or 25% of the population in Canada are suffering from chronic pain. Approaching these patients has always been a major challenge and of course individualized, particularly if they have sustained an injury like a car accident, work-related injury, etc. Many experts believe that chronic pain is sometimes a physical manifestation of psychological issues. Could experiencing injustice be one of them? Among the different questionnaires available for assessing chronic pain patients, including the Brief Pain Inventory (BPI), Pain stages of change (PSCQ), McGill pain, Pain Catastrophe Scale, etc, Injustice Questionnaire questionnaire (IEQ) could also be very useful. Particularly if injury has occurred as a result of another’s error or negligence. Therefore, pain patients might experience post-injury life with a sense of injustice as well as chronic pain disease. A study of 300 patients referred to Chronic Pain Management Program (CPMP) by community physicians in 2018 and 2019 showed that 160 of the patients sustained an injury as their inciting event, and 79% of them had IEQ scores of 30 or higher, indicating high level of perceived injustice. Furthermore, 92% had multiple investigations, and specialists’ visits, and polypharmacy without any clear diagnosis. Only 19% of them were suffering from severe and 38% from moderately depression but 36% of them had severe and 29% moderate anxiety.

**Introduction/Aim**: Although injustice has a long history in philosophy and psychosocial, only recently the topic of perceived injustice has been used among pain specialist. In many chronic pain patients, life following injury is associated with persistent psychosocioemotional issues. Current research suggest that perceived injustice consequent to injury might represent one of the strongest predictors of problematic outcomes. Injured individuals who report high levels of perceived injustice also experience more intense pain, more severe depression and are less likely to return to work. Individuals with high levels of perceived injustice display more pain behavior, seek more medical attention, and rate themselves as more severely disabled. Perceptions of injustice are also associated with the persistence of post-traumatic stress symptoms consequent to injury.

**Methods**: After having read extensive literature review about bioethics, injustice experience, and chronic pain disease; A retrospective cross-sectional study of 300 consecutive patients referred to CPMP, Thunder Bay, ON, in 2018 and 2019 was carried out. In this study, 160 patients were found to have an injury as their inciting event. They all were provided with Injustice Experience Questionnaire (IEQ) which is a 12-item scale that asks respondents to indicate the frequency with which they experience different thoughts concerning the sense of unfairness in relation to their injury. 153 of them completed and scored the IEQ. Those with the IEQ score of 30 or higher, representing clinically relevant level of perceived injustice, were included and their demographics data and result of other questionnaires (BPI, GAD-7, PHQ-9 and McGill Pain questionnaire) were collected and analyzed.

**Results**: Among 153 injury patients, 120 patients (79%) had the IEQ score of ≽30. Sixty two percent of them were female. Mean age = 52.25 and Range = 24–80 years old. Mean years of having pain was 12 years. 68% had diffuse or widespread body pain. 41% reported motor vehicle accident, 26% work-related injury, 10% fall, 9% sport-related injury, 11% reported fight and direct trauma, 2% from medical interventions like surgery, 1% others. Mean BPI score was 60/70, indicating high levels of pain interference. PHQ-9 showed 19% severe and 38% moderately depression. GAD-7, showed 36% severe and 29% moderate anxiety. 88% had sleep issues, and 64% complained of constant Fatigue. Only 21% had returned back to work. 92% had multiple investigations and without any clear diagnosis.

**Discussion/Conclusions**: Very high percentage of chronic pain patients in Northern Ontario are perceiving high levels of injustice, based on their IEQ scores. It is certainly a yellow flag in their pain management. Failure to pay attention to it may result in unsuccessful pain management. Prior knowledge of a patient’s level of perceived injustice, in addition to other pain-related variables, enable treatment plans to be more individually tailored.

## Designing and Evaluating a Virtual Reality Game for Facilitating Empathy towards Chronic Pain Patients

Xin Tong http://orcid.org/0000-0002-8037-6301^a,*^, Diane Gromala http://orcid.org/0000-0002-7737-9173^a^, Pegah Kiaei^a^, Chris D. Shaw http://orcid.org/0000-0002-6940-7971^a^ and Owen Williamson^b^

^a^School of Interactive Arts and Technology, Simon Fraser University, Surrey, British Columbia, Canada; ^b^Department of Epidemiology and Preventive Medicine, Monash University, Melbourne, Australia

**CONTACT** Xin Tong tongxint@sfu.ca

© 2020 The Author(s). Published with license by Taylor & Francis Group, LLC.

This is an Open Access article distributed under the terms of the Creative Commons Attribution License (http://creativecommons.org/licenses/by/4.0/), which permits unrestricted use, distribution, and reproduction in any medium, provided the original work is properly cited.

**Introduction/Aim**: Pain is a basic and necessary experience that alerts us to physical harm or infection. Yet pain is notoriously difficult to describe and harder still for one person to “stand in the shoes” or understand what another person’s pain is like. Empathy from other people is essential for treating or communicating with these patients, despite the barriers to feel patients’ pain. Therefore, we designed a VR game in which participants inhabit an avatar – a character who suffers from chronic pain.

**Methods**: To understand user experiences of our AS IF VR game, we designed a usability test. The study was a posttest, pretest study, proposed to assess participants’ empathy levels toward CP patients before and after playing AS-IF. Additionally, we analyzed the sense of embodiment (SoE) levels of our participants.

**Results**: Overall, after playing the VR game, participants scored significantly higher on the *Willingness to Help Scale and the Kindness subscale –* an adaptation of the Empathy questionnaire. Further, from the semi-structured interviews, we were able to gather essential feedback about the strengths and limitations of the current VR design, such as the effectiveness of pain representations.

**Discussion/Conclusions**: The visual-motor synchronicity of a player’s full-body movements mirrored by the avatar appears to elicit identification with the avatar. Results revealed that the game was effective in improving implicit and explicit empathy. Furthermore, findings showed that the game raised the emotional and perspective-taking aspects of players’ empathy.

## Exploring the Relationships between Post-Traumatic Stress Symptoms, Pain Catastrophizing, and Pain Outcomes in Survivors of Childhood Cancer and their Parents

Courtney Charnock^a,b,*^, Michaela Patton^c^, Caitlin Forbes^a,b^, Brooke Russell^c^, Melanie Noel http://orcid.org/0000-0003-3752-8055^c^, Melanie Khu^b^, Alexandra Neville^c^, Kathleen Reynolds^d,e^ and Fiona Schulte^a,b,c^

^a^Department of Oncology, Cumming School of Medicine, University of Calgary, Calgary, Alberta, Canada; ^b^Hematology, Oncology, and Transplant Program, Alberta Children’s Hospital, Calgary, Alberta, Canada; ^c^Department of Psychology, University of Calgary, Calgary, Alberta, Canada; ^d^Long Term Survivor’s Clinic, Alberta Children’s Hospital, Calgary, Alberta, Canada; ^e^Department of Family Medicine, Cumming School of Medicine, University of Calgary, Calgary, Alberta, Canada

**CONTACT** Courtney Charnock cncharno@ucalgary.ca

© 2020 The Author(s). Published with license by Taylor & Francis Group, LLC.

This is an Open Access article distributed under the terms of the Creative Commons Attribution License (http://creativecommons.org/licenses/by/4.0/), which permits unrestricted use, distribution, and reproduction in any medium, provided the original work is properly cited.

**Introduction**: Ongoing pain and post-traumatic stress symptoms (PTSS) are late effects among survivors of childhood cancer (SCCs). Previous research in non-cancer pediatric chronic pain populations has established robust relationships between parent and child PTSS and pain outcomes. Parent and child pain catastrophizing has been shown to mediate this relationship. These relationships have not been explored in SCCs. We investigated the role of parent and child PTSS and pain catastrophizing on pain outcomes in SCCs.

**Methods**: Canadian survivors [*n* = 94, 54.6% female, mean age = 17.0 years (*SD *= 4.74)] and their parents were recruited. Survivors self-reported PTSS, as well as pain catastrophizing, severity, and interference. Parents self-reported PTSS and catastrophizing about their survivor’s pain.

**Results**: Higher survivor pain catastrophizing was associated with higher pain severity (*r* =.49, *p* < .001) and interference (*r* =.43, *p* < .001). Higher parent pain catastrophizing was associated with higher survivor pain severity (*r* =.50, *p* = .004) but not interference (*r* =.14, *p* > .05). Neither parent nor child PTSS was associated with survivor pain outcomes (*p*’s >.05). Controlling for age and gender, linear regressions revealed parent and survivor pain catastrophizing each predicted survivor pain severity, and cumulatively accounted for 54% of the variance in pain severity scores.

**Conclusions**: Contrary to previous findings in the chronic pain literature, PTSS was unrelated to pain outcomes in SCCs. However, similar to chronic pain populations, parent and child pain catastrophizing impacted child pain interference and severity. Family-based interventions targeting pain catastrophizing should be evaluated for this population.

## Usability Testing of a mHealth Application for Managing Chronic Arthritis and Joint Health

Pegah Kiaei http://orcid.org/0000-0002-6416-1044^a,*^, Weina Jin^a^, Ruoyu Li^a^ and Diane Gromala http://orcid.org/0000-0002-7737-9173^a^

^a^The School of Interactive Arts and Technology, Simon Fraser University, Surrey, British Columbia, Canada

**CONTACT** Pegah Kiaei skiaeizi@sfu.ca

© 2020 The Author(s). Published with license by Taylor & Francis Group, LLC.

This is an Open Access article distributed under the terms of the Creative Commons Attribution License (http://creativecommons.org/licenses/by/4.0/), which permits unrestricted use, distribution, and reproduction in any medium, provided the original work is properly cited.

**Introduction/Aim:** Arthritis is projected to exacerbate with the increase of the aged population in Canada ^[1]^. Live With Arthritis Plus (LWAP) is an app designed by eTreatMD to diagnose joint arthritis symptoms and measure their impact factors and treatment effectiveness. Under industrial guidelines from the Food and Drug Administration (FDA), the researchers conducted a Human Factor Usability (HFU) study on LWAP, evaluating its intended Use of Device (UoD), Instructions for Use (IFU), and the Ability to Support Safe and Effective Use (ASSEU).

**Methods:** Aging adults (45+) diagnosed with Osteoarthritis (OA) or Rheumatoid Arthritis (RA) (“patients”) were recruited through a Snowball sampling for this study.

There were two main stages: a Training Stage (TrS) watching LWAP’s video tutorials, and a Testing Stage (TeS) of actually interacting with the app under-designed scenarios. The patients were then asked to fill out HFU questionnaires, followed by semi-structured interviews with the researchers.

**Results:** In-experiment observations and interviews showed that the patients were unable to use LWAP’s hand-image functionality due to their constant tremors of hands. Additionally, while a majority (64%) of the patients reported positive experiences with LWAP’s User Interface, those preferring outward methods (distraction) to confront pain denied future use of this app since daily pain tracking prevents distraction.

**Discussion/Conclusions**: Patients recording their pain symptoms and treatments in the past expressed satisfaction with LWAP. However, patients’ physical and age conditions should be expressly considered while polishing features including:

● Image-taking

● Icon size and consistency

● Error/Warning-raising

● Bulk selection accessibility

● Question frequency

## A Systematic Review of Virtual Reality and Augmented Reality Studies for Phantom Limb Pain Alleviation

Xin Tong http://orcid.org/0000-0002-8037-6301^a,*^, Xinxing Wang^b^, Diane Gromala http://orcid.org/0000-0002-7737-9173^a^, Bi-Fa Fan^b^, Owen Williamson^c^ and Kunlin Wei http://orcid.org/0000-0001-5098-3808^d^

^a^School of Interactive Arts and Technology, Simon Fraser University, Surrey, Canada; ^b^China-Japan Friendship Hospital, Beijing, China; ^c^Department of Epidemiology and Preventive Medicine, Monash University, Melbourne, Australia; ^d^Motor Control Lab, School of Psychological and Cognitive Sciences, Peking University, Beijing, China

**CONTACT** Xin Tong tongxint@sfu.ca

© 2020 The Author(s). Published with license by Taylor & Francis Group, LLC.

This is an Open Access article distributed under the terms of the Creative Commons Attribution License (http://creativecommons.org/licenses/by/4.0/), which permits unrestricted use, distribution, and reproduction in any medium, provided the original work is properly cited.

**Introduction/Aim:** Phantom Limb Pain (PLP) is a type of neuropathic pain perceived in a part of the body after a limb is “missing” through amputation or is nonfunctioning as a result of a severe injury such as a Brachial Plexus Avulsion (BPA). Examples of non-pharmacological interventions include mirror therapy (MT), motor imagery, the use of immersive virtual reality (VR) and augmented reality (AR) technologies. Among these interventions, VR and AR technology have been shown to reduce PLP during and temporarily after the intervention; in some cases, the pain alleviation lasted for extended periods of time, up to six months.

**Methods:** In March 2019, a search was conducted in the databases of Cochrane, PubMed, IEEE, ACM, and Google Scholar. Using the primary keywords “Phantom Limb Pain,” “Virtual Reality,” and “Augmented Reality,” twenty research studies in total were selected. All these studied were reviewed and their reported analgesic effects analyzed.

**Results:** Researchers using VR/AR interventions reported approximately 34–55% decreases in perceived pain as indicated by the McGill Pain Questionnaire with varying intervention durations and frequencies. Notably, prior research showed majority PLP patients reported a decreased awareness of their phantom limb’s kinesthesia. Some researchers used VR/AR interventions to restore the patients’ sense or control of phantom limb movement, which in turn leads to pain reduction.

**Discussion/Conclusions:** The findings and implications of this review may shed light on the analgesic effects of VR/AR interventions, on future designs of VR/AR environments and tasks that are critical for the effectiveness of alleviating PLP.

## Designing and Implementing a Patient-Driven Pain Assessment Tool in the Pediatric Perioperative Setting

Catherine Stratton^a,*^, Jennifer Tyrrell^a,b^ and Lisa Isaac^a,c^

^a^Department of Anesthesiology and Pain Medicine, The Hospital for Sick Children, Toronto, Ontario, Canada; ^b^Lawrence S. Bloomberg Faculty of Nursing, University of Toronto, Toronto, Ontario, Canada; ^c^Faculty of Medicine, Department of Anesthesia, University of Toronto, Toronto, Ontario, Canada.

**CONTACT** Catherine Stratton catherine.stratton@sickkids.ca

© 2020 The Hospital for Sick Children and The University of Toronto. Published with license by Taylor & Francis Group, LLC.

This is an Open Access article distributed under the terms of the Creative Commons Attribution License (http://creativecommons.org/licenses/by/4.0/), which permits unrestricted use, distribution, and reproduction in any medium, provided the original work is properly cited.

**Introduction/Aim**: This study aims to limit surgically instigated chronic pain risk by adapting the Ontario Pediatric Chronic Pain Network (OPCN) registry to create a patient-driven perioperative pain assessment questionnaire for the pediatric Transitional Pain Service (pTPS).^1,2^

**Methods**: Patients and parents seen in the pTPS from July-December 2019 are entered in this Quality Improvement project. We followed the Plan-Do-Study-Act Cycle.^3^

*Plan*: Investigators identified relevant Patient-Reported Outcomes Measurement Information System (PROMIS®) pain-related scores^4^ and developed qualitative questions on patients’ pain goals. Lastly, investigators added a satisfaction survey to inform questionnaire improvements.

*Do*: Questionnaires are e-sent to participants the week before their pTPS appointment in Research Electronic Data Capture (REDCap^TM^).

*Study*: Investigators review questionnaire completion, satisfaction and recommendations.

*Act*: Recommendations applicable to all participants are implemented during the study to observe satisfaction changes over time. A pTPS’ staff focus group will finalize the questionnaire by assessing recommendations’ feasibility.

**Results**: 56 questionnaires were sent to 23 patient-parent dyads. Participants completed the questionnaire in 3–20 minutes. 53.57% questionnaires were completed which is consistent with other pediatric psychological surveys.^5^ 14.29% patients “strongly agreed” and 64.29% “agreed” the questionnaire captured important information about their pain. 13.33% parents “strongly agreed” and 53.33% “agreed” with this. 7.14% “strongly disagreed” with this statement. 13.33% “disagreed,” but changed their responses to “agree” or “strongly agreed” after the questionnaire’s revisions. 92.86% patients and 93.34% parents felt the language in questionnaire was clear.

**Discussion/Conclusions**: Results from the satisfaction survey support a qualitative and quantitative mixed-methods approach since it allows patient goals to drive their perioperative care.

References1.Katz
J, Weinrib
A, Fashler
S, Katznelson
R, Shah
B, Ladak
S, … Clarke
H. The Toronto general hospital transitional pain service: development and implementation of a multidisciplinary program to prevent chronic postsurgical pain. J Pain Res.
2015:695. doi:10.2147/jpr.s91924.26508886PMC46108882.Lavand’homme
P. Transition from acute to chronic pain after surgery. Pain. 2017;158:S50–S54. doi:10.1097/j.pain.0000000000000809.281346533.Agency for Healthcare Research and Quality, Plan-Do-Study-Act (PDSA) Cycle; 2008
11. https://innovations.ahrq.gov/qualitytools/plan-do-study-act-pdsa-cycle.4.Mcgrath
PJ, Walco
GA, Turk
DC, Dworkin
RH, Brown
MT, Davidson
K, … Zeltzer
L. Core outcome domains and measures for pediatric acute and chronic/recurrent pain clinical trials: pedIMMPACT recommendations. J Pain. 2008;9(9):771–83. doi:10.1016/j.jpain.2008.04.007.185622515.Johnston
D, Gerbing
R, Alonzo
T, Aplenc
R, Nagarajan
R, Schulte
F, Cullen
P, Sung
L. Patient-reported outcome coordinator did not improve quality of life assessment response rates: a report from the childrens oncology group. PLoS One. 2015;10(4):4. doi:10.1371/journal.pone.0125290.PMC441113625915772

## Neurophysiological Markers of Conditioned Pain Modulation (CPM) Analgesia in Chronic Pain Patients

Hyerang Jin^a,*^, Bart Witjes^b^, Mathieu Roy http://orcid.org/0000-0002-3335-445X^c^, Sylvain Baillet^a^ and Cecile de Vos^b^

^a^Department of Neurology and Neurosurgery, McGill University, Montreal, QC, Canada; ^b^Department of Anesthesiology, Erasmus University Medical Centre, Rotterdam, The Netherlands; ^c^Department of Psychology, McGill University, Montreal, QC, Canada

**CONTACT** Hyerang Jin hyerang.jin@mail.mcgill.ca

© 2020 The Author(s). Published with license by Taylor & Francis Group, LLC.

This is an Open Access article distributed under the terms of the Creative Commons Attribution License (http://creativecommons.org/licenses/by/4.0/), which permits unrestricted use, distribution, and reproduction in any medium, provided the original work is properly cited.

**Introduction/Aim**: Conditioned pain modulation (CPM) is currently used to evaluate the efficiency of the endogenous modulatory controls which are generally dysfunctional in chronic pain patients. It is a “pain inhibits pain” phenomenon, where noxious pain perception is reduced by a spatially distant conditioning pain. Our study aimed to identify brain-signal biomarkers of CPM efficiency to validate its use in the clinic and to facilitate objective measurement of impaired pain modulatory systems in chronic pain patients.

**Methods**: In 17 chronic low back pain patients and 19 age- and sex-matched healthy volunteers scanned with magnetoencephalography (MEG), we evoked test pain sensation using transcutaneous electrical stimulations on the right tibial nerve and delivered conditioning pain in the form of an ice pack on the left arm. The three conditions of the experiment were baseline (test pain), CPM (test pain and conditioning pain) and recovery (test pain).

**Results**: As expected, chronic pain patients generally showed impaired CPM. In healthy subjects, the suppression in alpha frequencies in response to test pain was attenuated during CPM (compared to the baseline condition). We also found that the identified MEG correlate of CPM was significantly reduced in chronic pain patients.

**Discussion/Conclusions**: These findings support the known compromised function of the descending pain inhibitory system in chronic pain patients. If robust neural correlates of impaired CPM analgesia in chronic pain patients are found, they could be used to study pain pathophysiology and assist in optimizing and personalizing treatments.

## Chronic Pain Treatment Approaches and Associated Pain Relief by Pain Type in Individuals with Cerebral Palsy

Abagail M. Raiter^a,*^, Lisa A. Lykken^a^, Alyssa Merbler^b^, Chantel Barney^a^ and Frank Symons^b^

^a^Research, Gillette Children’s Specialty Healthcare, St. Paul, Minnesota, USA; ^b^Educational Psychology, University of Minnesota, Minneapolis, Minnesota, USA

**CONTACT** Abagail M. Raiter AbagailRaiter@gillettechildrens.com

© 2020 The Author(s). Published with license by Taylor & Francis Group, LLC.

This is an Open Access article distributed under the terms of the Creative Commons Attribution License (http://creativecommons.org/licenses/by/4.0/), which permits unrestricted use, distribution, and reproduction in any medium, provided the original work is properly cited.

**Introduction/Aim**: Although pain is readily acknowledged as a persistent problem affecting quality of life among individuals living with cerebral palsy (CP), there is relatively limited research specific to what caregivers do to help. We aimed to investigate types of pain experienced by individuals with CP and common pain-relieving approaches utilized by caregivers.

**Methods**: Eighty-six participants (58.1% male; mean age = 17.2 years, range 6–38 years) with CP were enrolled. A majority of participants were quadriplegic (77%) and relied on wheeled mobility (Gross Motor Function Classification System [GMFCS] levels IV–V; 86%). Pain type, pain intensity (0–10 numeric rating scale; 0 = no pain, 10 = worst pain ever), treatment approach, and pain relief (0–10 numeric rating scale; 0 = didn’t help at all, 10 = completely relieved pain) were assessed by caregiver report. Mean pain intensity (MPI) was calculated for each type of pain reported before treatment. Mean pain relief (MPR) was calculated for each treatment approach.

**Results**: Fifty-one (61.45%) participants were living with chronic pain. MPI was 7.9 when rated for a bad day compared to 2.5 when rated for a good day. The two most common pains included musculoskeletal pain (including spasms, positioning, and stretching pain; n = 68) and gastrointestinal pain (n = 11). The most frequent treatment to relieve musculoskeletal pain was medication (n = 25, MPI = 7.36, MPR = 5.32), changing positions (n = 24, MPI = 5.13, MPR = 6.58), and massage (n = 19, MPI = 6.74, MPR = 5.16); in some cases no treatment was provided (n = 9, MPI = 4.89).

**Discussion/Conclusions**: Musculoskeletal pain is prevalent in individuals with CP, and additional research is needed to investigate treatment approaches in this vulnerable population.

## Companion Dogs and Their Impact on the Sleep of Patients with Chronic Pain

Cary A. Brown http://orcid.org/0000-0002-5282-4170^a,*^, Yuluan Wang^a^, Katrina Liddiard^b^ and Eloise Carr http://orcid.org/0000-0003-1870-4244^c^

^a^Department of Occupational Therapy, University of Alberta, Edmonton, Canada; ^b^School of Exercise and Health Sciences, Edith Cowan University, Perth, Australia; ^c^Faculty of Nursing, University of Calgary, Calgary, Canada

**CONTACT** Cary A. Brown cary.brown@ualberta.ca

© 2020 The Author(s). Published with license by Taylor & Francis Group, LLC.

This is an Open Access article distributed under the terms of the Creative Commons Attribution License (http://creativecommons.org/licenses/by/4.0/), which permits unrestricted use, distribution, and reproduction in any medium, provided the original work is properly cited.

**Introduction**: Chronic pain is prevalent in many industrialized nations. Pain takes a significant toll on personal physical and mental wellbeing, and exerts very high costs to families, employers and society. Encouragingly, research shows that pain and sleep have a reciprocal nature, thus suggesting that interventions to improve sleep may decrease pain symptoms. To-date, we know little about how companion dog ownership may influence the pain/sleep relationship. Typical advice to remove pets from the bedroom negates the possible positive benefit of human-animal co-sleeping; a more nuanced examination is warranted. The objective of this study was to investigate pain patients’ perception about the impact of their pet dog on sleep.

**Methodology**: A content analysis of interview data exploring patients’ perception about the impact of the pet dog on sleep. The qualitative dataset was extracted from a subgroup of participants in a larger study focused on the pain patient/pet dog relationship. The subgroup was asked, “Does your dog have a positive or negative impact on your sleep?” Using an iterative approach, the data were thematically coded.

**Main Findings**: Theme codes included: companionship; physical presence/’cuddles’; routine/schedule; distraction from anxiety/worry at night; reassuring/protective presence; active intervention to keep participant safe; daytime activity to promote sleeping at night; and reciprocal concern for the sleep of the pet dog.

**Principle Conclusions and Implications**: Companion dogs may play important roles in helping some chronic pain patients achieve better quality sleep. Routine advice to remove the dog to improved sleep could be counter-productive and more nuanced and contextualized recommendations should be developed.^1^

Reference1.Brown
C, Wang
Y, Carr
E. Undercover dogs: pet dogs in the sleep environment of patients with chronic pain. Soc Sci.
2018;7(9):157. doi:10.3390/socsci7090157.

## Non-Pharmacological Interventions for Managing Chronic Non-Cancer Pain: What Does the Evidence Say?

Colleen Donder^a,*^

^a^Knowledge Mobilization, CADTH, Kingston, Nova Scotia, Canada

**CONTACT** Colleen Donder colleend@cadth.ca

© 2020 CADTH. Published with license by Taylor & Francis Group, LLC.

This is an Open Access article distributed under the terms of the Creative Commons Attribution License (http://creativecommons.org/licenses/by/4.0/), which permits unrestricted use, distribution, and reproduction in any medium, provided the original work is properly cited.

**Introduction/Aim**: The opioid crisis has taken the lives of thousands of Canadians over the past few years. While there are no simple solutions, the crisis demands action and that policy and practice decisions be informed by credible evidence. This includes understanding and applying the available evidence on non-pharmacological interventions for managing pain. This poster will present the evidence found on various non-pharmacological interventions for chronic, non-cancer pain management.

**Methods**: Rapid reviews of the evidence were conducted on various non-pharmacological interventions for chronic non-cancer pain. Information specialists performed limited literature searches of key databases and resources; titles and abstracts of the retrieved publications were screened by one reviewer for possible inclusion. Full-text publications were evaluated for final article selection according to predetermined selection criteria (population, intervention, comparator, outcomes, and study designs).

**Results**: CADTH has produced over 40 Rapid Response reports on non-pharmacological interventions for chronic pain management since January 2016. The evidence identified for, but not limited to, the following interventions for chronic pain will be presented: acupuncture, mindfulness, yoga, cognitive behavioral therapy, multidisciplinary treatment programs, manual therapy for chronic neck and back pain, and exercise for knee osteoarthritis. The amount and level of evidence varied by intervention. Gaps in the literature were also identified suggesting areas where more research is needed.

**Discussion/Conclusions**: Many non-pharmacological interventions have evidence to support their use in chronic-pain management. Understanding the evidence on non-pharmacological interventions and putting it into practice and policy can change the way chronic pain is managed.

## Activation of NK1 Receptors is Required for the Reversal of Mechanical Hyperalgesia via Non-ionotropic NMDA Signaling

David Rodriguez^a,*^, Abigail D’Souza^a^ and Robert Bonin^a^

^a^Pharmaceutical Sciences, University of Toronto, Toronto, Ontario, Canada

**CONTACT** David Rodriguez luisdavid.rodriguez@mail.utoronto.ca

© 2020 The Author(s). Published with license by Taylor & Francis Group, LLC.

This is an Open Access article distributed under the terms of the Creative Commons Attribution License (http://creativecommons.org/licenses/by/4.0/), which permits unrestricted use, distribution, and reproduction in any medium, provided the original work is properly cited.

**Introduction/Aim**: Pathological pain is associated with changes to the synaptic strength of nociceptive pathways in the spinal cord dorsal horn. Our lab has previously demonstrated that reactivation of potentiated pathways induces a labile state, which renders relevant synapses susceptible to disruption. Synaptic plasticity is highly dependent on NMDA receptor activity. Within the brain, NMDA receptors can signal via ionotropic (calcium-dependent; I-NMDA) or non-ionotropic (NI-NMDA) mechanisms, which are associated with synaptic potentiation and depotentiation, respectively. In this study, we test the hypothesis that NI-NMDA signaling mediates synaptic depotentiation in sensitized spinal pathways and contributes to the reversal of hyperalgesia.

**Methods**: Postsynaptic field potentials (fPSPs) in spinal cord explants to study long-term potentiation (LTP) within the dorsal horn. Von Frey assay to measure withdrawal response to mechanical stimuli.

**Results**: Administration of APV (glutamate-site antagonist) had no effect on sensitized pathways. However, pharmacological isolation of NI-NMDA using 7CK (glycine-site antagonist) caused the reversal of dorsal horn LTP, in both male and female mice. Behaviorally, intrathecal administration of 7CK+NMDA significantly increased withdrawal thresholds following capsaicin-induced sensitization in an NK1-depedent manner.

**Discussion/Conclusions**: NI-NMDA activity reverses LTP in sensitized nociceptive pathways, and it ameliorates mechanical hyperalgesia. Inhibition of I-NMDA, without the activation of NI-NMDA signaling, does not reverse spinal LTP. NI-NMDA reversal of hyperalgesia appears to require the engagement of NK1 receptors. Our results identify NI-NMDA receptor activity as a novel target for the treatment of pathological pain; we will continue to explore how the NK1 receptor is able to regulate or “gate” the reversal of hyperalgesia by NI-NMDA.

## Amygdala Resting-State Functional Connectivity in Youth with Chronic Pain and Its Relation with Pain-related Distress and Fear Learning

Inge Timmers http://orcid.org/0000-0001-7162-6623^a,*^, Marina López-Solà http://orcid.org/0000-0002-5517-2665^b^, Lauren Heathcote http://orcid.org/0000-0003-2515-3102^a^, Marissa Heirich^a^, Gillian Rush^a^, Deborah Shear^a^, David Borsook^c^ and Laura Simons http://orcid.org/0000-0002-3395-9483^a^

^a^Department of Anesthesiology, Perioperative, and Pain Medicine, Stanford University School of Medicine, Stanford, California, USA; ^b^University of Cincinnati, Cincinnati Children’s Hospital, Cincinnati, Ohio, USA; ^c^Department of Anesthesiology, Critical Care and Pain Medicine, Boston Children’s Hospital, Boston, Massachusetts, USA

**CONTACT** Inge Timmers itimmers@stanford.edu

© 2020 The Author(s). Published with license by Taylor & Francis Group, LLC.

This is an Open Access article distributed under the terms of the Creative Commons Attribution License (http://creativecommons.org/licenses/by/4.0/), which permits unrestricted use, distribution, and reproduction in any medium, provided the original work is properly cited.

**Introduction/Aim**: Brain imaging studies have indicated altered amygdala resting-state functional connectivity (rsFC) in youth with chronic pain, but whether and how the amygdala network manifests in the context of threat learning is unclear. Here, we study rsFC following fear acquisition in youth with chronic pain, aiming to investigate alterations compared to healthy peers, and its relation to pain-related distress and fear learning.

**Methods**: 75 youth were included in analyses (age M = 15.8, SD = 2.9; 64 females; 46 patients). Resting-state functional MRI was collected after participants underwent a differential fear conditioning (Screaming Lady) paradigm. Seed-based functional connectivity analyses were performed, focused on group comparisons, followed by whole-brain mediation analyses investigating whether amygdala rsFC mediates the relation between pain-catastrophizing and differential fear-learning (fear to CS+>CS-) in patients.

**Results**: Post-acquisition fear did not differ across groups, but was significantly related to pain-related distress in patients (CS+>CS- with pain-catastrophizing: *r* = .41, *p* = .005). Patients showed increased coupling between left amygdala and two clusters (right supramarginal gyrus/SMG and right inferior frontal gyrus/IFG) compared to controls. In patients, amygdala-SMG coupling correlated with pain-related distress (*r* = −.33, *p* = .03). Analyses further revealed that amygdala-SMG coupling mediated the relation between pain-catastrophizing and differential fear in patients.

**Discussion/Conclusions**: In youth with chronic pain, elevated pain-related distress was associated with facilitated fear acquisition. This association was significantly mediated by amygdala-SMG coupling at rest, which was increased in patients compared to healthy peers. The results strengthen a link between pain-related distress, augmented neural integration of bodily signals and core emotional processing in youth with chronic pain.

## Pain Management Program Outcomes in Veterans with Chronic Pain & Comparison with Non-Veterans

Eleni G. Hapidou^a,*^ and Jane Jomy^b^

^a^Michael G. DeGroote Pain Clinic, Hamilton Health Sciences and McMaster University, Hamilton, Ontario, Canada; ^b^Bachelor of Health Sciences Honor’s Program, McMaster University, Hamilton, Ontario, Canada

**CONTACT** Eleni G. Hapidou hapidou@hhsc.ca

© 2020 The Author(s). Published with license by Taylor & Francis Group, LLC.

This is an Open Access article distributed under the terms of the Creative Commons Attribution License (http://creativecommons.org/licenses/by/4.0/), which permits unrestricted use, distribution, and reproduction in any medium, provided the original work is properly cited.

**Introduction/Aim**: Among veterans in Canada, twice as many (41%) experience chronic pain compared to the general population (20%). The aim of this study was to compare chronic pain management outcomes in 68 veterans and 68 non-veterans attending an intensive, four-week chronic pain management program at the Michael G. DeGroote Pain Clinic. The two groups were matched for age and gender.

**Methods**: Data were obtained from psychometric measures completed by patients at admission and discharge to examine program effectiveness and differences in pain experience. These included bothersome symptoms, pain intensity, pain-related disability, depression, anxiety, catastrophizing, kinesiophobia, sensitivity to pain traumatization, stages of change, acceptance of pain, and satisfaction measures.

**Results**: MANOVA was used to examine session (admission – discharge) by group (veteran – non-veteran) differences on psychometric measures using SPSS. Data analysis yielded significant differences from admission to discharge and between veterans and nonveterans on catastrophizing, kinesiophobia, sensitivity to pain traumatization, pain acceptance, stages of change, and pain coping, all favoring veterans.

**Discussion/Conclusions**: Evidence supports the effectiveness of the interdisciplinary pain management program in addressing chronic pain and comorbidity in veterans and non-veterans and provides insight into how pain is experienced differently by veterans. Further research on veteran chronic pain would provide more insight into the unique pain experience within this group.

## What Happens to Adolescents with Low Back Pain? A Study of Pain Trajectories from 14 to 22 Years of Age

Amber Beynon^a,*^, Jeffrey Hebert^b^, Charlotte Lebouef-Yde^c^, Darren Beales^d^, Angela Jacques^d^ and Bruce Walker^a^

^a^College of Science, Health, Engineering and Education, Murdoch University, Murdoch, Australia; ^b^Faculty of Kinesiology, University of New Brunswick, Fredericton, New Brunswick, Canada; ^c^Clinical Biomechanics, Institute for Regional Sundhedsforskning, Funen, Odense, Denmark; ^d^School of Physiotherapy and Exercise Science, Curtin University, Bentley, Australia

**CONTACT** Amber Beynon amber.beynon@murdoch.edu.au

© 2020 The Author(s). Published with license by Taylor & Francis Group, LLC.

This is an Open Access article distributed under the terms of the Creative Commons Attribution License (http://creativecommons.org/licenses/by/4.0/), which permits unrestricted use, distribution, and reproduction in any medium, provided the original work is properly cited.

**Introduction/Aim**: Low back pain (LBP) often starts during childhood or adolescence. Therefore, it is important to understand the course of LBP in a young population. Understanding the different pain trajectories experienced by young people may help to inform prevention and treatment strategies in this population. The study aim was to identify LBP trajectories from early adolescence to early adulthood.

**Methods**: We analyzed longitudinal data from 942 participants who were enrolled in the Western Australian Raine Study cohort. Data on LBP with impact on daily living were collected at the ages of 14, 17, 20, and 22. We constructed group-based trajectory models to identify discrete trajectories of LBP with impact.

**Results**: Three LBP trajectory subgroups were identified from 14 to 22 years of age. One group included almost half the participants (46%) who had a high and increasingly high probability of LBP. Another subgroup with 46% of the participants had a consistently low probability of LBP, and the third group comprising less than 10% of the participants had a decreasing probability of LBP.

**Discussion/Conclusions**: Since LBP in adolescence has a strong tendency to continue into early adulthood, two important issues arise: 1. Is it possible to identify the determinants of LBP within this population? and 2. Can LBP be prevented within this population? It is likely that factors other than those usually reported in the literature (psycho-social and biomechanical) need to be taken into account in such studies.

## Inhibition of Acute Inflammation Leads to Chronification of Inflammatory Pain

Lucas V. Lima^a,*^, Gabrielle G. Dutra^a^, Marc Parisien^a^, Luda Diatchenko^a^ and Jeffrey S. Mogil^a^

^a^Alan Edwards Centre for Research on Pain, McGill University, Montreal, Quebec, Canada

**CONTACT** Lucas V. Lima lucas.vasconceloslima@mail.mcgill.ca

© 2020 The Author(s). Published with license by Taylor & Francis Group, LLC.

This is an Open Access article distributed under the terms of the Creative Commons Attribution License (http://creativecommons.org/licenses/by/4.0/), which permits unrestricted use, distribution, and reproduction in any medium, provided the original work is properly cited.

**Introduction/Aim**: Recent evidence suggests that tissue healing is coordinated by inflammatory cells right from the onset of the inflammatory response, suggesting that anti-inflammatory treatments might delay recovery. We investigated the effect of inhibiting the inflammatory response on pain chronification.

**Methods**: CD-1 male and female mice were treated with dexamethasone (a corticosteroid) for seven consecutive days, either via micropumps or subcutaneous injections (0.5 mg/kg/day). Mice treated with vehicle were used as control (n = 4–5 per group). Complete Freud’s Adjuvant (CFA) was injected into the left hindpaw of mice on the second day of treatment in all mice, as a model of inflammatory pain. Mechanical paw withdrawal threshold (PWT) was measured prior to treatment (baseline) and at regular intervals until both groups recovered to their baseline PWT values.

**Results**: Mice treated with dexamethasone either via micropumps or daily injections showed an acute anti-allodynic effect observed at day 3 to day 6 post-CFA (micropumps) and day 6 to day 11 (daily injection). Allodynia in saline-treated mice recovered according to the usual time course, reaching baseline PWT levels by day 11 to day 15 post-CFA. However, dexamethasone-treated mice displayed persistent allodynia lasting up to 100 days post-CFA.

**Discussion/Conclusions**: Current data from our experiments suggest that interfering with the inflammatory response taking place immediately after injury with a corticosteroid (dexamethasone), although promoting acute pain relief, might lead to pain chronification. If this phenomenon is also seen in humans, it could have dramatic implications for the treatment of acute pain.

## Can We Change Patients’ Health Outcomes Related to Chronic Pain by Educating Their Healthcare Providers?

Jane Zhao http://orcid.org/0000-0002-4143-9031^a,*^, Dominika Bhatia http://orcid.org/0000-0002-9621-0672^b^, Leslie Carlin^c^, Paul Taenzer^d^ and Andrea Furlan http://orcid.org/0000-0001-6138-8510^a,e^

^a^Toronto Rehabilitation Institute, Project ECHO at UHN, Toronto, Ontario, Canada; ^b^Institute of Health Policy, Management and Evaluation, University of Toronto, Toronto, Ontario, Canada; ^c^Department of Anthropology, University of Toronto, Toronto, Ontario, Canada; ^d^Department of Physical Medicine and Rehabilitation, Faculty of Health Sciences, Queen’s University, Kingston, Ontario, Canada; ^e^Department of Medicine, University of Toronto, Toronto, Ontario, Canada

**CONTACT** Jane Zhao jane.zhao@uhn.ca

© 2020 The Author(s). Published with license by Taylor & Francis Group, LLC.

This is an Open Access article distributed under the terms of the Creative Commons Attribution License (http://creativecommons.org/licenses/by/4.0/), which permits unrestricted use, distribution, and reproduction in any medium, provided the original work is properly cited.

**Introduction/Aim**: Project ECHO (Extension for Community Healthcare Outcomes) is a tele-education model that uses weekly videoconferencing sessions and case-based learning to connect specialists with HCPs in rural, remote, and underserved areas. Launched in 2014, ECHO Ontario Chronic Pain and Opioid Stewardship (“ECHO”) aims to educate, support, and improve chronic pain management in the province of Ontario, Canada.

In this study, we examined the outcomes, attitudes, and experiences of patients with chronic pain whose cases were presented by their HCPs during ECHO sessions.

**Methods**: We conducted a mixed-methods study with patients who were presented during ECHO sessions from December 2015 to January 2019. For the quantitative component, we used seven validated questionnaires assessed at two time points. For the qualitative component, a sample of patients was selected for in-depth interviews.

**Results**: From December 2015 to January 2019, 195 patients were presented during the weekly ECHO sessions. We obtained consent from 42 patients. The majority of self-reported questionnaires did not show significant changes before and after their case presentation.

Nineteen interviews were conducted. Thematic analysis revealed patients’ perceptions about ECHO: patients appreciated the opportunity for their HCP to present their case to a group of interprofessional specialists.

**Discussion/Conclusions**: This study highlights the difficulties in managing patients with chronic pain in rural, remote, and underserved areas. Qualitative investigation offers insight to how people with chronic pain value specialist care of their condition, even when their numeric pain scores remain unchanged, and thus sheds light on the nature of ‘care”.

## Prolonged Hyperalgesia Induced by Optogenetic Activation of TRPV1+ Nociceptors Using Direct Spinal Light Delivery

Maham Zain^a,*^, Laura A. Bennett^a^, Jerry Li^a^ and Robert P. Bonin^a^

^a^Leslie Dan Faculty of Pharmacy, University of Toronto, Toronto, Ontario, Canada

**CONTACT** Maham Zain maham.zain@mail.utoronto.ca

© 2020 The Author(s). Published with license by Taylor & Francis Group, LLC.

This is an Open Access article distributed under the terms of the Creative Commons Attribution License (http://creativecommons.org/licenses/by/4.0/), which permits unrestricted use, distribution, and reproduction in any medium, provided the original work is properly cited.

**Introduction/Aim**: The recent strides in optogenetics has spurred research into the neural basis of pain *in vivo* and in freely moving animals. We have developed and characterized a method for the delivery of light to the spinal dorsal horn that is suitable for optogenetic studies in freely behaving mice. Using this approach, we activated TRPV1 + nociceptors expressing channelrhodopsin (ChR2) to investigate how the activation of these sensory fibers can both acutely modulate behavior and induce hyperalgesia.

**Methods**: A ceramic ferrule was surgically implanted in the lumbar vertebrae of wildtype (WT) mice and mice expressing ChR2 in TRPV1 + afferents (TrpV1-ChR2). A fiber optic patch cable connected the implanted lens to a blue LED light source for stimulation of the central terminals of the TRPV1 + primary afferents. Possible adverse effects of this surgery were studied using immunohistochemical assessment of spinal cord glial activation, open field and rotarod behavioral assays. Mechanical sensitivity was assessed using von Frey filaments and thermal sensitivity using a thermal gradient assay to measure thermal preference.

**Results**: We observed that the surgical technique did not result in any changes in evoked motor function, spontaneous locomotion or mechanosensitivity. In addition, there was no evidence of astrocyte or microglia activation in the spinal cord after surgery. Delivery of blue light through the implant was found to result in robust nocifensive behaviors in the TRPV1-ChR2 mouse line. After suprathreshold stimulation of TRPV1-ChR2 at 2-Hz for 10 minutes, mice exhibited robust mechanical hypersensitivity which lasted approximately 2 weeks. Similar stimulation in implanted WT mice did not result in mechanical hypersensitivity. Notably, thermal preferences of the mice were not affected by either suprathreshold 2-Hz stimulation or acutely delivered subthreshold stimulation. Finally, delivery of light at a subthreshold intensity that did not acutely produce nocifensive behaviors produced progressively more pronounced nocifensive behaviors after prolonged stimulation.

**Discussion/Conclusions**: Optogenetic activation of TRPV1 + afferents produced a surprisingly long-lasting mechanical hypersensitivity. This suggests that optogenetically-induced hyperalgesia may mechanistically differ from the shorter duration hypersensitivity induced by capsaicin activation of peripheral TRPV1+afferents. In addition, persistent subthreshold stimulation eventually resulted in an emergence of nocifensive behaviors over the time course of the stimulation, alluding to potential wind-up. Further work will investigate the mechanisms of these behavioral changes.

## Lower Resting Vagal Tone in Patients with Primary Headache: Preliminary Results from a Randomized-controlled Trial

Blake Boehme http://orcid.org/0000-0002-6972-8695^a,*^, Abid Azam http://orcid.org/0000-0002-6016-1274^a^, Joel Katz http://orcid.org/0000-0002-8686-447X^a^, Helia Ghazinejad^a^, Fatma Al-Rubeye^a^, Myra Massey^a^ and Aviel Oppenheimer^a^

^a^Psychology, York University, Toronto, Ontario, Canada

**CONTACT** Blake Boehme blakeaboehme@gmail.com

© 2020 The Author(s). Published with license by Taylor & Francis Group, LLC.

This is an Open Access article distributed under the terms of the Creative Commons Attribution License (http://creativecommons.org/licenses/by/4.0/), which permits unrestricted use, distribution, and reproduction in any medium, provided the original work is properly cited.

**Introduction/Aim**: Heart rate variability (HRV) is used to measure the activity of the parasympathetic branch of the autonomic nervous system (ANS), referred to as vagal tone. Vagal activity in individuals with primary headache conditions including migraine and tension-type headache have been explored with equivocal results. This study is based on data from an ongoing randomized-controlled trial (NCT#: NCT03296007). The purpose of this secondary study was to examine the resting vagal tone of patients with primary headache conditions compared to a control group.

**Methods**: Undergraduate participants with a self-reported primary headache diagnosis (n = 31) were compared with control participants (n = 23) without headache. HRV data was collected using an electrocardiogram (ECG) system (MindWare) in a rest phase of 5 minutes while participants sat comfortably in a chair with their eyes closed. HRV was computed as the power in the high-frequency bandwidth (HF-HRV) and the root mean squared of the successive differences (RMSSD) in the ECG recordings. HRV was analyzed using separate one-way ANOVAs with group (headache, controls) as the independent factor and HF-HRV and RMSSD as dependent variables.

**Results**: Preliminary results showed significantly lower HF-HRV power (*F*_1,53_ = 9.3, *p* =.004) and RMSSD (*F*_1.53_ = 5.9, *p* =.018) at rest in the headache group compared to controls. The 2 groups did not differ on age or sex.

**Discussion/Conclusions**: These findings further our understanding of the ANS in headache disorders showing a hypofunction of the parasympathetic nervous system in people with migraine and tension-type headache.

## Chronic Pain in LGBTQ2S+ Youth: A Scoping Review

Vanessa Swanson^a,*^, Jillian Vinall Miller^b^ and Kelsey Barrie^c^

^a^Medicine, University of Calgary, Calgary, Alberta, Canada; ^b^Anesthesia, Perioperative and Pain Medicine, University of Calgary, Calgary, Alberta, Canada; ^c^Alberta Children’s Hospital Complex Pain Clinic, Vi Riddell Children’s Pain & Rehabilitation Centre, Calgary, Alberta, Canada

**CONTACT** Vanessa Swanson vanessa.swanson@ucalgary.ca

© 2020 The Author(s). Published with license by Taylor & Francis Group, LLC.

This is an Open Access article distributed under the terms of the Creative Commons Attribution License (http://creativecommons.org/licenses/by/4.0/), which permits unrestricted use, distribution, and reproduction in any medium, provided the original work is properly cited.

**Introduction/Aim**: Chronic pain, defined as ongoing or recurrent pain lasting more than three months is a growing epidemic. The burden of chronic pain disproportionately impacts populations affected by social inequities and discrimination. Therefore, we examined whether the prevalence of chronic pain is greater among LGBTQ2S+ youth as compared to heterosexual cis-gendered youth, and what factors may underlie this disparity.

**Methods**: A scoping review of all publications that reported on chronic pain in LGBTQ2S+ youth was conducted using PubMed and PsycINFO, using the key words: bisexual, chronic pain, gay, gender identity, lesbian, pain, persistent pain, queer, sexual identity, sexual orientation, transgender, transsexual.

**Results**: A total of 10 publications were included in the final analysis. Chronic pain impacts 10–40% of youth, and pain prevalence rates are generally higher among females. Two population-based studies found that sexual minorities (i.e. gay, lesbian, bisexual) reported higher rates of chronic pain compared to same-gender heterosexuals. Several stress factors were identified that in part explained this discrepancy. Sexual minority groups reported poorer sleep quality, lower socioeconomic status, and greater internalizing symptoms (anxiety/depression), suicide ideation and childhood maltreatment. There is a paucity of pain research in youth that are questioning their sexual and/or gender identities, are transgender, or are transitioning.

**Discussion/Conclusions**: The role of sexual and gender identity in the development of chronic pain among youth remains relatively unexplored. However, the evidence-to-date would suggest that these youth are at risk for developing persistent pain problems. Future research is required to guide the care of this vulnerable population.

## Beliefs and Attitudes towards Medicinal Cannabis for Chronic Pain Among Pain Physicians: A Qualitative Study

Mark Phillips^a,b,*^, Ramesh Zacharias^c,d^ and Jason W. Busse http://orcid.org/0000-0002-0178-8712^a,d,e,f^

^a^Department of Health Research Methods, Evidence, and Impact, McMaster University, Hamilton, Ontario, Canada; ^b^Division of Orthopaedic Surgery, McMaster University, Hamilton, Ontario, Canada; ^c^The Michael G. DeGroote Pain Clinic, McMaster University, Hamilton, Ontario, Canada; ^d^The Michael G. DeGroote Centre for Medicinal Cannabis Research, McMaster University, Hamilton, Ontario, Canada; ^e^The Michael G. DeGroote Institute for Pain Research and Care, McMaster University, Hamilton, Ontario, Canada; ^f^Department of Anesthesia, McMaster University, Hamilton, Ontario, Canada

**CONTACT** Mark Phillips phillimr@mcmaster.ca; @M_Phillips44

© 2020 The Author(s). Published with license by Taylor & Francis Group, LLC.

This is an Open Access article distributed under the terms of the Creative Commons Attribution License (http://creativecommons.org/licenses/by/4.0/), which permits unrestricted use, distribution, and reproduction in any medium, provided the original work is properly cited.

**Introduction/Aim**: This study aimed to provide insights into pain physicians opinions, values, and beliefs regarding the use of medical cannabis for chronic pain management.

**Methods**: This study utilized a focused ethnography approach. Pain management clinicians within the Hamilton and Greater Toronto Area were recruited through snowball sampling methods to identify a purposeful sample of individuals with a deep knowledge regarding medical cannabis use for chronic non-cancer pain. Thematic analysis was conducted to identify opinions, values and beliefs that pain management physicians held regarding therapeutic cannabis.

**Results**: A total of 7 participants were included within this study. There were 4 (57%) males and 3 (43%) females included, with a mean length of clinical practice of 19.7 (SD 16.9) years. The five key themes identified within this evaluation of pain management clinicians’ beliefs were: (1) lack of evidence published on benefits and harms of medical cannabis, (2) lack of education on medical cannabis use for chronic pain, (3) discrepancy between clinician’s and patient’s views of medical cannabis’ role in pain management, (4) difficulty with determining the appropriate dose of cannabis for patients, and (5) prohibitive costs of medical cannabis for patients.

**Discussion/Conclusions**: Our study-in-progress suggests that a number of pain physicians and their patients are interested in the potential role of cannabis for chronic pain, but barriers to use include lack of evidence and education, uncertainty around dosing, cost, and discrepancies between physician and patient beliefs.

## Is Sitting Causing the Pain in Your Butt? The Influence of Posture on Laser Evoked Potentials

Lukas D. Linde http://orcid.org/0000-0001-5180-4904^a,*^, Carey M. Ogryzlo^a^, Jessica McDougall^b^ and John L. K. Kramer^a^

^a^School of Kinesiology, University of British Columbia, Vancouver, British Columbia, Canada; ^b^School of Rehabilitation Sciences, University of British Columbia, Vancouver, British Columbia, Canada

**CONTACT** Lukas D. Linde lukas.linde@ubc.ca

© 2020 The Author(s). Published with license by Taylor & Francis Group, LLC.

This is an Open Access article distributed under the terms of the Creative Commons Attribution License (http://creativecommons.org/licenses/by/4.0/), which permits unrestricted use, distribution, and reproduction in any medium, provided the original work is properly cited.

**Introduction/Aim**: Laser evoked potentials (LEP) are used to objectively quantify human nociception, typically assessed in a lying posture. The effect of altered postures on LEP outcomes remains unknown. Our aim was to investigate the influence of altering posture (lying, sitting, standing) on LEP outcomes and pain perception.

**Methods**: Twenty (20) healthy participants attended a single testing session. Noxious radiant heat stimuli were delivered by an infrared (Nd:YAP) laser, at participants’ pain threshold, with a pulse diameter of 5 mm (Electronic Engineering, Florence, Italy). Laser stimuli were delivered to the left forearm and thigh in three postural conditions (lying, sitting, standing). We delivered 20 stimuli at each location in all three postures, in a randomized order. Brain activity was recorded through 32 electroencephalography (EEG) electrodes, according to 10–20 international positioning. LEP parameters (N2 latency, N2 amplitude, P2 latency, P2 amplitude, and N2P2 amplitude) were calculated from averaged EEG epochs (100 ms pre-laser to +1000 ms post-laser).

**Results**: Preliminary results (N = 4) revealed a significant increase in N2P2 amplitude in the sitting posture (Mean±SD: 47.6 ± 14.3 [uV], *p* < .05) compared to standing (38.4 ± 10.8) during arm stimulation. N2 amplitude was significantly reduced in the sitting posture (−30.5 ± 12.4 [uV], *p* < .05) compared to both lying (−23.0 ± 10.3) and standing (−19.6 ± 10.6) postures during arm stimulations. No significant differences were observed for remaining LEP outcomes or pain perception.

**Discussion/Conclusions**: Preliminary data suggest sitting produces larger LEP N2P2 amplitudes and N2 amplitudes without increasing pain perception. These preliminary findings may indicate a neural mechanism for why standing tends to be associated with pain relief.

## Correlations of Hypertension and Hypoalgesia among Complex Chronic Disease Patients in Ontario

Meaghan Ferguson^a,*^, Maxwell Slepian^b^ and Joel Katz http://orcid.org/0000-0002-8686-447X^a^

^a^Psychology, York University, Toronto, Ontario, Canada; ^b^GoodHope Ehlers-Danlos Syndrome Clinic, Toronto General Hospital, Toronto, Ontario, Canada

**CONTACT** Meaghan Ferguson mferg@Yorku.ca

© 2020 The Author(s). Published with license by Taylor & Francis Group, LLC.

This is an Open Access article distributed under the terms of the Creative Commons Attribution License (http://creativecommons.org/licenses/by/4.0/), which permits unrestricted use, distribution, and reproduction in any medium, provided the original work is properly cited.

**Introduction/Aim**: Hypertension-induced hypoalgesia, also known as decreased pain sensitivity due to high blood pressure has been documented in small-scale studies but has yet to be examined in a large Canadian database of complex care residents.

**Methods**: The Continuing Care Reporting System database was used, containing health information on residents of Canadian complex chronic care facilities (Ontario only) who were assessed using the Resident Assessment Instrument Minimum Data Set, V2.0. Hypertension was reported among 77,323 residents (65.5%, original *n = *118,122). Propensity score matching, with a 1:1 ratio, was used to identify a control record without hypertension for each case. McNemar’s test was used to evaluate dichotomous pain outcomes and Wilcoxon Signed Ranks test for ordinal pain levels (4-level and 6-level pain variables). Multinomial logistic regression was used to quantify the effect of hypertension on 4-level ordinal pain variables (no pain, mild, moderate, severe), controlling for potential confounders.

**Results**: The matched dataset included *n = *40,799 patients with and *n = *40,799 without hypertension. We observed a lower proportion of hypertension patients with pain (71.7%) compared to controls (72.6%), *OR *= 0.96 (95% CI 0.97–0.99), *p* = .007. Patients with hypertension were less likely to report mild (*OR* = 0.96 (95% CI 0.93–1.00), *p* = .051), moderate (*OR *= 0.98 (95% CI 0.95–1.01), *p* = .210) and severe pain (*OR *= 0.75 (95% CI 0.71–0.89), *p* < .001) after controlling for confounding variables.

**Discussion/Conclusions**: The results support the relationship between hypertension and reduced pain sensitivity on a population level. More studies are needed to understand the causes of this relationship and potential consequences of hypertension-related hypoalgesia.

## Spinal Dorsal Horn Neuronal Populations and Synaptic Proteins Underlying Central Sensitization and Pain Reconsolidation

Laura A. Bennett^a,*^, Hantao Zhang^a,*^, Robert P. Bonin^a^

^a^Pharmaceutical Sciences, University of Toronto, Toronto, Ontario, Canada

**CONTACT** Laura A. Bennett laura.bennett@mail.utoronto.ca; @lauraabennett

*indicates co-first authors

© 2020 The Author(s). Published with license by Taylor & Francis Group, LLC.

This is an Open Access article distributed under the terms of the Creative Commons Attribution License (http://creativecommons.org/licenses/by/4.0/), which permits unrestricted use, distribution, and reproduction in any medium, provided the original work is properly cited.

**Introduction/Aim**: Central sensitization is a critical process contributing to pathological pain, whereby spinal dorsal horn neurons undergo plastic changes in excitability. Our lab has previously found that central sensitization can be attenuated or reversed by a process similar to memory reconsolidation: an activity-dependent process of protein degradation and de-novo synthesis that can alter previously formed memories. Currently, the mechanisms that enable pain reconsolidation are poorly understood. Here, we investigated changes in protein expression as well as differential activation of neuronal populations in an animal model of sensitization to determine what molecular mechanisms may facilitate pain reconsolidation.

**Methods**: Central sensitization was induced by intraplantar injections of 1% capsaicin (w/v) in the hindpaw of adult mice. A second injection of 1% capsaicin in the same hindpaw initiated pain reconsolidation as previously described. Mechanical sensitivity was measured by Von Frey filaments. Immunohistochemistry was utilized for determination of cFos positive dorsal spinal neurons, indicative of neuronal activation. Western blots were conducted to quantify changes in synaptic protein expression.

**Results**: Central sensitization was assessed as a significant decrease in withdrawal responses to Von Frey filaments after peripheral capsaicin injection. Synaptic protein expression of ERK, GKAP, SHANK, phospho-ERK, CAMKII, GluA1, GluA2, and GluN1 were investigated using western blot. Dorsal spinal neuron activation was analyzed by cFos positive neurons and found to be 2-fold higher on the ipsilateral side of capsaicin injection compared to the contralateral side.

**Discussion/Conclusions**: Taken together, our results indicate a protein and neuronal-population specific process underlying the development of central sensitization after capsaicin injection. The changes in protein expression associated with sensitization may enable the induction of reconsolidation after a second capsaicin injection, and therefore represent novel targets for manipulating and reversing central sensitization.

## Father and Mother Reminiscing Style about past Pain is Differentially Associated with Young Children’s Cognitive Abilities

Tatiana Lund^a,*^, Maria Pavlova^a^, Madison Kennedy^a^, Cara Nania^a^ and Melanie Noel http://orcid.org/0000-0003-3752-8055^a^

^a^Psychology, University of Calgary, Calgary, Alberta, Canada

**CONTACT** Tatiana Lund tclund@ucalgary.ca

© 2020 The Author(s). Published with license by Taylor & Francis Group, LLC.

This is an Open Access article distributed under the terms of the Creative Commons Attribution License (http://creativecommons.org/licenses/by/4.0/), which permits unrestricted use, distribution, and reproduction in any medium, provided the original work is properly cited.

**Introduction/Aim**: Parent-child reminiscing has been shown to shape children’s cognitive, social, and emotional outcomes. Two distinct parent reminiscing styles have been identified: repetitive (maladaptive) and elaborative (adaptive). Recent research demonstrated differences in reminiscing about past pain between mothers and fathers and this was linked to children’s pain-related cognitions (pain memory biases). The current study examined whether parents’ reminiscing style about past pain differed as a function of children’s broader cognitive abilities, and the moderating role of child sex and parent role.

**Methods**: 116 four-year-old children (54% girls) and one of their parents (49% mothers) participated. Children completed four *NIH Toolbox* measures of cognition. Parent-child dyads reminisced about a past autobiographical event when their child experienced pain. Narratives were coded using an established coding scheme for elements of style/content.

**Results**: Mothers’ but not fathers’ statement repetitions were positively associated with receptive vocabulary (*r* = .271, *p* = .046). Fathers’ but not mothers’ more frequent use of yes-no repetition questions was negatively associated with children’s attention (*r* = −.271, *p* = .050). Greater episodic memory was associated with more frequent paternal but not maternal explanations (*r* =.377, *p* = .006). Parent statement repetitions were positively associated with receptive vocabulary in boys, but not girls (*r* =.425, *p* = .002). More frequent use of yes-no repetition questions was linked to worse attention and episodic memory in boys, but not girls (*rs *= −.397;-415, *ps *< .010).

**Discussion/Conclusions**: Children’s cognitive skills were differentially related to elements of repetitive reminiscing style depending on parent role and children’s sex, which may be explained by differences in gender socialization. Future research should longitudinally examine the directionality of this relationship.

## Impaired Opioid Responsiveness is Associated with Changes in Amygdala Function in an Animal Model of Multiple Sclerosis

Zoë Dworsky-Fried^a,*^, Bradley J. Kerr http://orcid.org/0000-0001-5900-4442^b,c^ and Anna M.W. Taylor http://orcid.org/0000-0001-7881-8783^a,b,c^

^a^Department of Pharmacology, University of Alberta, Edmonton, Alberta, Canada; ^b^Department of Anesthesiology and Pain Medicine, University of Alberta, Edmonton, AB, Canada; ^c^Neuroscience and Mental Health Institute, University of Alberta, Edmonton, Alberta, Canada

**CONTACT** Zoë Dworsky-Fried dworskyf@ualberta.ca

© 2020 The Author(s). Published with license by Taylor & Francis Group, LLC.

This is an Open Access article distributed under the terms of the Creative Commons Attribution License (http://creativecommons.org/licenses/by/4.0/), which permits unrestricted use, distribution, and reproduction in any medium, provided the original work is properly cited.

**Introduction/Aim**: Chronic pain is a frequent and disabling symptom of multiple sclerosis (MS). Opioids are highly effective in managing acute pain but are ineffective for treating MS-related pain. The amygdala plays a key role in modulating chronic pain and opioid analgesia. Here, we describe how experimental autoimmune encephalomyelitis (EAE), the most frequently used animal model of MS, alters behavioral responses and cellular activation in the amygdala following systemic opioid administration.

**Methods**: We employed the myelin oligodendrocyte glycoprotein (MOG)-induced experimental autoimmune encephalomyelitis (EAE) model in male and female C57BL/6 mice.

The reinforcing properties of morphine sulfate were assessed with the conditioned place preference paradigm. Morphine antinociception was measured using the tail withdrawal assay and the formalin test. Microglial activation, neuronal activity and μ-opioid receptor gene (OPMR1) expression in the amygdala were analyzed with immunohistochemistry and fluorescence *in situ* hybridization.

**Results**: Mice with EAE exhibited impaired reward and analgesia in response to systemic morphine. This loss of morphine efficacy correlated with robust microglial activation and reduced c-Fos expression in the amygdala during a noxious stimulus while OPRM1 expression remained unchanged compared to controls.

**Discussion/Conclusions**: Our experiments provide an intriguing mechanism for why opioids are less effective in treating MS-related pain. Our data also identifies microglial activation within the amygdala as a potential target to improve the efficacy of opioids in this patient population. Because EAE induces glial activation in regions involved in mood and affect, our findings may translate to other disorders, including anxiety and depression, that demonstrate high comorbidity with MS.

## Treatment Modality for the Postoperative Muscular Pain Management in Pylorus Preserving Pancreaticoduodenectomy

Hari Prasad Dulal^a,*^ and Bidur Khatiwoda^b^

^a^Asunta Medicare Pvt Ltd Clinical, Bhaktapur, Nepal; ^b^Nepal Army Institute of Health Science, Medical College Kathmandu, Kathmandu, Nepal

**CONTACT** Hari Prasad Dulal dulalhari75@gmail.com

© 2020 The Author(s). Published with license by Taylor & Francis Group, LLC.

This is an Open Access article distributed under the terms of the Creative Commons Attribution License (http://creativecommons.org/licenses/by/4.0/), which permits unrestricted use, distribution, and reproduction in any medium, provided the original work is properly cited.

**Introduction/Aim**: Pylorus preserving pancreaticoduodenectomy (PPPD) is treatment for resectable tumor in pancreaticoduodenal system. In open surgery, incision and suture of the abdominal muscles cause the inflammatory changes, strain, muscular pain which deteriorates the postoperative quality of life.

**Methods**: The 15 patients who underwent PPPD in Department of Surgery were randomly assigned to two groups. Group 1 was assigned as control group while group 2 received Electrical twitch obtaining intramuscular stimulation (ETOIMS) after surgery under the general anesthesia. ETOIMS was conducted on patient using Clavis (Alpine Biomed ApS, Denmark). Aseptic monopolar needle electrode was inserted into transverse abdominis muscle under ultrasound guidance. Pain score peak cough flow (PCF), gait speed was compared to preoperative values. Results were analyzed by linear mixed model or repeated measures ANOVA using SPSS 23.0

**Results**: The pain score was highest on the day of surgery (Group 1: 6.5 ± 2.2, Group 2: 5.5 ± 1.9, *P* = .17) and decreased gradually. Throughout the course, the pain scores were significantly lower in Group 2 after PPPD (*P* = .01). Although the PCF at each measuring time points didn’t show the group difference (*P* = .20), the improved PCF from the second day of surgery to discharge was greater in Group 2 (*P* = .02). Although gait speeds at each time point were not significantly different, time course of improving gait speed was significantly faster in Group 2 (*P* < .01).

**Discussion/Conclusions**: ETOIMS can be used as new treatment modality for pain control after PPPD with high beneficiary effects.

## Neurological symptoms in Ehlers Danlos Syndrome (EDS): a cross sectional study using population-powered data

Adena Gutstein^a,*^, Lindsay Wilson^b^, Susan Carleton^c^, Sandy Smeenk^d^ and Susan Edwards^d^

^a^Family Medicine, Bio Health Center, Toronto, Ontario, Canada; ^b^Brain Injury and Spinal Cord Rehabilitation, Toronto Rehabilitation Institute, Toronto, Ontario, Canada; ^c^The ILC Charitable Foundation, Coldwater, Ontario, Canada; ^d^The ILC Charitable Foundation, Oakville, Ontario, Canada

**CONTACT** Adena Gutstein adenagutstein@gmail.com

© 2020 The Author(s). Published with license by Taylor & Francis Group, LLC.

This is an Open Access article distributed under the terms of the Creative Commons Attribution License (http://creativecommons.org/licenses/by/4.0/), which permits unrestricted use, distribution, and reproduction in any medium, provided the original work is properly cited.

**Introduction**: Emerging reports indicate that patients with EDS suffer from disabling neurological symptoms. The impact of these symptoms on the patient population and healthcare system remain unclear.

**Aims**: Document and describe the neurological manifestations of EDS, and estimate toll of symptoms on patients and health service usage.

**Methods**: Data was collected in a population-based survey administered electronically via social media and community organizations. The survey included 30 questions looking at type, frequency and severity of neurological symptoms, as well as level of disability associated with symptoms. Respondents selected from a list of 20 symptoms, each of which were graded on a 5-pt scale (1 = absent, 5 = severe). Disability was measured by degree of functional impairment (0 = no impairment, 5 = life threatening), and health service usage was assessed using a composite metric that combined frequency of hospital visits and number of practitioners seen.

**Results**: A total of 373 respondents completed the survey, 260 of which had a diagnosis of EDS confirmed by a specialist. Of these, 84% (202/241) reported ≥ 1 neurological symptom; 95% (192/202) were disabled by these symptoms; and 39% (79/202) had seriously considered suicide. Headache/migraine and fatigue were the most prevalent symptoms, with the highest severity ratings. Lastly, neurological disability was associated with increased health service usage (R = 0.3, *p* < .0001).

**Conclusions**: The prevalence and severity of neurological symptoms in the EDS population suggest they are an important part of the emerging picture of this syndrome, and have implications on patient health and the healthcare system.

This study involved the collection of population-based, patient-powered data, and took place in partnership with the community organization “The ILC Charitable Foundation”. The study authors confirm that all research was conducted in an ethical fashion and in line with the principles of the Declaration of Helsinki.

## Sigma-1 Receptor Antagonist Haloperidol Reduces Tolerance Induced by Morphine in Rats with Neuropathic Pain

Licet Caridad Mena-Valdés^a,*^, Yisel Blanco Hernández^a^ and Francisco Javier López-Muñoz^a^

^a^Pharmacobiology Department, Cinvestav-Coapa, Mexico City, Mexico

**CONTACT** Licet Caridad Mena-Valdés licet.mena90@gmail.com

© 2020 The Author(s). Published with license by Taylor & Francis Group, LLC.

This is an Open Access article distributed under the terms of the Creative Commons Attribution-NonCommercial License (http://creativecommons.org/licenses/by-nc/4.0/), which permits unrestricted non-commercial use, distribution, and reproduction in any medium, provided the original work is properly cited.

**Introduction/Aim**: Haloperidol (Hal) is a prototype of butyrophenone widely used as antipsychotic agent. Recently, some authors described the anti-nociceptive effects of Hal mediated by sigma-1 receptors (S1R) antagonism. Morphine (Mor) is currently used in neuropathic pain (NP) therapy, however there is accumulating evidence that opioid therapy might not only be associated with the development of tolerance but also with an increased sensitivity to pain. The aim of this work was to evaluate the interaction between haloperidol and morphine on tolerance induced by morphine in rats with neuropathic pain.

**Methods**: Male Wistar rats (220–250 g) from Cinvestav-Coapa bioterium were used for experiments. Chronic constriction injury (CCI) model was developed to induce NP. The effects of Hal as S1R antagonist on Mor-induced tolerance, were evaluate for 7 days. The anti-hyperalgesic and anti-allodynic effects of repeated administrations (twice/day) of Hal (0.0178 mg/kg), Mor (0.0316 mg/kg) and their combination were evaluated by von Frey test and could allodynia test, respectively.

**Results**: Animals administered in repeated doses of Mor 0.0316 mg/kg presented tolerance after 5 day of treatment. However, animals administered with Mor 0.0316 mg/kg plus Hal 0.0178 mg/kg, showed antinociception during the 7 days of evaluation, achieving a maximum anti-hyperalgesic and anti-allodynic effects at day 4, which was maintained until the last day of evaluation.

**Discussion/Conclusions**: The interaction between S1R and the opioid system opens a window in neuropathic pain treatment. This study showed that haloperidol potentiates the antinociceptive effects of morphine and additionally modulates the tolerance induced by the opioid.

## Antinociceptive Effects of the Combination Tramadol and N -palmitoylethanolamide in Rats with Chronic Constriction of the Sciatic Nerve

Yisel Blanco-Hernández^a,*^, Licet Caridad Mena-Valdés^a^, Myrna Déciga-Campos^b^ and Francisco Javier López-Muñoz^a^

^a^Pharmacobiology Department, Cinvestav-Coapa, Mexico City, Mexico; ^a^Higher School of Medicine, IPN, Mexico City, Mexico

**CONTACT** Yisel Blanco-Hernández yisel.blanco90@gmail.com

© 2020 The Author(s). Published with license by Taylor & Francis Group, LLC.

This is an Open Access article distributed under the terms of the Creative Commons Attribution License (http://creativecommons.org/licenses/by/4.0/), which permits unrestricted use, distribution, and reproduction in any medium, provided the original work is properly cited.

**Introduction/Aim**: Neuropathic pain (NP) is a chronic disorder with a complex and multifactorial etiology. The NP prevalence constitute a significant health problem worldwide. Among drugs used in the NP treatment is tramadol (TRAM), a prototype of analgesic opioid drug. Moreover, it has recently described the antinociceptive effects of the cannabinoids for example N-palmitoylethanolamide (PEA).

**Methods**: Single doses of TRAM (1.0–31.6 mg/Kg, p.o), PEA (0.03–10.00 mg/Kg, p.o.) and the corresponding combinations were administered, from which the dose-response curves (DRC) were constructed. The anti-allodynic and anti-hyperalgesic effects were determined using the cold allodynia and von Frey tests in the chronic constriction of the sciatic nerve (CCI) model. The evaluations were made up to 10 days after the surgery at 30, 60, 90, 120 and 180 minutes after the administration of the drugs.

**Results**: The DRC analysis for anti-allodynic effects of drugs displayed similar efficacies, though in the DRC analysis for anti-hyperalgesic effects TRAM showed greater efficacy that PEA. It was found that TRAM, PEA and the combination showed antinociceptive effects significative respect as the control group (*p* < .05). Different combinations have been evaluated which have shown additive and potentiative effects. Of them, the combination of TRAM 3.16 mg/Kg + PEA 0.316 mg/Kg presents effect of potentiation which does not present significant differences (*p* > .05) with higher doses evaluated of the individual drugs.

**Discussion/Conclusions**: These results demonstrate that TRAM, PEA and the combination have antinociceptive effect in rats with NP induced by CCl, and the combination of TRAM 3.16 mg/Kg + PEA 0.316 mg/Kg evidence effects of potentiation type.

## OnabotulinumtoxinA Treatment Improved Health-Related Quality of Life in Adults with Chronic Migraine: Results from a Canadian, Prospective, Observational Study (PREDICT)

Guy Boudreau^a,*^, Ian Finkelstein^b^, Corrie Graboski^c^, May Ong^d^, Suzanne Christie^e^, Katherine Sommer^f^, Meetu Bhogal^g^, Goran Davidovic^g^ and Werner J. Becker^h^

^a^Neurological Treatment Center, Centre Hospitalier Universitaire de Montréal (CHUM), Montréal, QC, Canada; ^b^Toronto Headache & Pain Clinic, Toronto, ON, Canada; ^c^Island Health, Brentwood Bay, BC, Canada; ^d^Pain Centre, St Paul Hospital, Vancouver, BC, Canada; ^e^Neurology, University of Ottawa (Neurology), Ottawa, ON, Canada; ^f^Medical Affairs, Allegan Plc, Marlow, UK; ^g^Global Medical Affairs & CMO Strategic Planning, Allergan Plc, Markham, ON, Canada; ^h^Hotchkiss Brain Institute, University of Calgary, Calgary, Alberta, Canada

**CONTACT** Guy Boudreau guypboudreau@gmail.com

© 2020 The Author(s). Published with license by Taylor & Francis Group, LLC.

This is an Open Access article distributed under the terms of the Creative Commons Attribution License (http://creativecommons.org/licenses/by/4.0/), which permits unrestricted use, distribution, and reproduction in any medium, provided the original work is properly cited.

**Introduction/Aim**: PREDICT aimed to assess long-term health-related quality of life (HRQOL) in Canadian patients with chronic migraine (CM) treated with onabotulinumtoxinA.

**Methods**: Canadian, multicentre, prospective, observational study (NCT02502123) in adults naïve to botulinum toxin(s) for CM. OnabotulinumtoxinA administered ≤7 treatment cycles per the Canadian product monograph. Primary endpoint: mean change in Migraine-Specific Quality of Life (MSQ) Tx4 vs. baseline. Secondary endpoints: onabotulinumtoxinA treatment utilization (each session), headache days (daily headache diary), and physician (baseline, Tx4, and final visit)/patient (each session) satisfaction. Unless noted, data presented as mean(SD); number of patients (n).

**Results**: 184 participants (average 45 years, predominantly female [84.8%] and Caucasian [94.6%]) received ≥1 treatment with onabotulinumtoxinA. Mean dose of onabotulinumtoxinA/treatment cycle was 171(18) U; treatment interval 13.2(1.8) weeks. At baseline, patients reported 20.9(6.7) headache days/month, which decreased over time (range: −3.5[6.3] Tx1[n = 184] to −6.5[6.6] Tx4[n = 150]; all timepoints versus baseline, *p* < .0001). Significant increases in MSQ post-Tx4(n = 150) (restrictive: 21.5, preventive: 19.5, emotional: 22.9) were observed versus baseline, exceeding minimal clinically important differences (all, *p* < .0001). Following onabotulinumtoxinA treatment, most physicians rated their patients as improved (Tx4[n = 150]: 96.6%, final visit[n = 160]: 86.9%) and majority of patients were satisfied (range: 55.1% [Tx2;n = 174] to 85.8% [Tx7;n = 127]). 77 patients (41.8%) reported 168 treatment emergent adverse events (TEAEs), with 38 TEAEs in 22 patients (12.0%) considered treatment-related. 4 patients (2.2%) reported 6 serious TEAEs, none were considered treatment-related. No new safety signals were identified.

**Discussion/Conclusions**: Real-world data from PREDICT demonstrate that onabotulinumtoxinA treatment for CM reduced headache days and improved HRQOL, with high physician and patient satisfaction.

## Understanding Parent Behaviours in Response to Child Acute Pain: Parent Heart Rate Variability, Anxiety, and Catastrophizing

Kaytlin Constantin^a,*^, Rachel Moline^a^, Lindsay Labonte^a^ and C. Meghan McMurtry http://orcid.org/0000-0002-3278-1169^a^

^a^Department of Psychology, University of Guelph, Guelph, ON, Canada

**CONTACT** Kaytlin Constantin kaytlin@uoguelph.ca

© 2020 The Author(s). Published with license by Taylor & Francis Group, LLC.

This is an Open Access article distributed under the terms of the Creative Commons Attribution License (http://creativecommons.org/licenses/by/4.0/), which permits unrestricted use, distribution, and reproduction in any medium, provided the original work is properly cited.

**Introduction/Aim**: Parent behaviors strongly predict children’s acute pain response; less clear are the factors shaping parent behaviors. Heart rate variability (HRV) is considered a physiological correlate of current emotion regulatory efforts (moment-to-moment changes in HRV; “state HRV”) and the capacity for emotion regulation (resting HRV; “trait HRV”). We examined how parent state HRV relates to parent behaviors before and during their child’s acute pain, and trait HRV as a moderator between parental state anxiety, catastrophizing and behaviors.

**Methods**: Fifty-six children between 7 and 12 years of age completed the cold pressor task (CPT) in the presence of a primary caregiver. Parents completed the State-Trait Anxiety Inventory-State and the Pain Catastrophizing Scale-Parents-State. Parent HRV was examined at 30-second epochs at rest (“trait HRV”), before (i.e., “state HRV-warm”), and during their child’s CPT (“state HRV-cold”). HRV was calculated using a frequency-domain measure. Parent behaviors during the CPT were video recorded and coded as “coping-promoting” (CP) or “distress-promoting” (DP) using the Child-Adult Medical Procedure Interaction Scale-Revised.

**Results**: A small to moderate nonsignificant negative correlation was observed between state HRV-cold and CP behaviors during CPT. Parental trait HRV moderated the association between catastrophizing and DP behaviors.

**Discussion/Conclusions**: Parents with low state HRV during child pain engaged in greater CP behaviors, suggesting that modest decreases in parent HRV in the moment may be adaptive in the immediate pain context. In contrast, parents who generally have a low (vs. high) HRV may be at risk of engaging in DP behaviors when catastrophizing about their child’s pain.

## Determinants of Pain Assessment Documentation in Intensive Care Units

Jenna L. Morris^a,*^, Francis Bernard^b,c^, Mélanie Bérubé^d,e^, Jean-Nicolas Dubé^f,g^, Julie Houle^g,h^, Denny Laporta^i,j^, Suzanne N. Morin^k^, Marc Perreault^l,m^, David Williamson^c,l^ and Céline Gélinas http://orcid.org/0000-0001-7948-5570^a,i^

^a^Ingram School of Nursing, McGill University, Montréal, Québec, Canada; ^b^Faculty of Medecine, Université de Montréal, Montréal, Québec, Canada; ^c^Hôpital du Sacré-Coeur de Montréal, CIUSSS Nord-Ile-Montréal, Montréal, Québec, Canada; ^d^Faculty of Nursing, Université Laval, Québec City, Québec, Canada; ^e^CHU de Québec, Université Laval Research Center (Enfant-Jésus Hospital), Québec City, Québec, Canada; ^f^Centre Hospitalier Affilié Universitaire Régional, CIUSSS Mauricie-Centre-du-Québec, Trois-Rivières, Québec, Canada; ^g^Department of Nursing, Université du Québec à Trois-Rivières, Trois-Rivières, Québec, Canada; ^h^Centre Hospitalier Affilié Universitaire Régional, CIUSSS Mauricie et Centre-du-Québec, Trois-Rivières, Québec, Canada; ^i^Jewish General Hospital, CIUSSS Centre-West-Montréal, Montréal, Québec, Canada; ^j^Faculty of Medicine, Respiratory Division, McGill University, Montréal, Québec, Canada; ^k^Center for Outcomes Research and Evaluation, McGill University, Montréal, Québec, Canada; ^l^Faculty of Pharmacy, Université de Montreal, Montréal, Quebec, Canada; ^m^McGill University Health Center, Montreal, Québec, Canada

**CONTACT** Jenna L. Morris jenna.morris@mail.mcgill.ca

© 2020 The Author(s). Published with license by Taylor & Francis Group, LLC.

This is an Open Access article distributed under the terms of the Creative Commons Attribution License (http://creativecommons.org/licenses/by/4.0/), which permits unrestricted use, distribution, and reproduction in any medium, provided the original work is properly cited.

**Introduction/Aim**: Adequate pain assessment with appropriate self-report and behavioral tools depending on communication abilities is necessary to ensure optimal patient outcomes. This study aimed to 1) describe pain assessment documentation (0–10 numerical rating scale or 0–8 Critical-Care Pain Observation Tool [CPOT]) by nurses over a 24-hour period on the second day post-ICU admission, and 2) identify the sociodemographic and clinical determinants of pain assessment documentation.

**Methods**: A descriptive-correlational retrospective design was used. Sociodemographic (i.e., age, sex) and clinical data (i.e., diagnosis, level of consciousness, opioids, pain assessments) were extracted from the charts of 303 ICU patient admissions to five teaching hospitals between 2017 and 2019. Analyses consisted of descriptive statistics and multiple linear regression.

**Results**: 67.7% were male and mean age was 60.4 years (SD 18.1). 39.6% had a medical diagnosis, followed by trauma (33.7%) and surgical (26.7%). The median number of pain assessments ranged from 1 to 6 across sites, and two sites had fewer assessments compared to others (*p* <.001). Overall, pain assessment was present in 69.6% of charts, but only 20.5% of opioid doses were followed by documented pain reassessment within one hour. Higher level of consciousness (β = 0.365), using only breakthrough doses (β = 0.236), and lower opioid dose (β = −0.207) were significant determinants of pain assessment documentation (R^2^ = 0.252).

**Discussion/Conclusions**: Current pain assessment documentation is suboptimal in ICUs, especially for patients receiving higher opioid doses or who cannot self-report. Study findings highlight the need to implement tools and protocols to optimize pain assessment and documentation.

## Engaging the Patients: Experience from a Pharmacist Intervention Targeting High Dose High Risk Prescription Opioids in the Community Setting

Feng Chang http://orcid.org/0000-0003-4661-6911^a,*^, Mo Chen^a^ and Agnes Kluz^b^

^a^School of Pharmacy, University of Waterloo, Waterloo, Ontario, Canada; ^b^Huron Community Family Health Team, CCFP(PC), Seaforth, Ontario, Canada

**CONTACT** Feng Chang feng.chang@uwaterloo.ca School of Pharmacy, University of Waterloo, Waterloo, Ontario, Canada

© 2020 The Author(s). Published with license by Taylor & Francis Group, LLC.

This is an Open Access article distributed under the terms of the Creative Commons Attribution License (http://creativecommons.org/licenses/by/4.0/), which permits unrestricted use, distribution, and reproduction in any medium, provided the original work is properly cited.

**Introduction/Aim**: High-dose opioid use is associated with significant risks and limited benefit. The 2017 Canadian opioid guidelines emphasized caution against doses at or above 90 mg morphine equivalent daily (MED).

**Methods**: A naturalistic mixed-method study in community pharmacies was conducted. Participating pharmacists received training and tools on screening for opioid doses at or above 90 mg MED for consultation. At 8 months post-training, a retrospective chart review of pharmacist documentation and patient medication history was conducted to determine patient and prescriber reception. Mean MED was compared using paired t test.

**Results**: Seven pharmacists participated with 35 patients taking high dose opioids identified. Average patient age was 55 yrs, 60% male, average 268.6 mg MED. About 40% (14/35) were initially receptive to pharmacist consultation; 42.8% (15/35) were reluctant or not interested; 11.4% (4/35) transferred pharmacy; and 2 patients’ initial responses were unclear. At 8 months, 16/35 (45.7%) had MED dose reduced, with 1 completely tapered off and 1 put on opioid replacement therapy; 11 (31.4%) made no changes; 4 (11.4%) increased dose, and 4 (11.4%) were lost to follow up due to transferring pharmacy. All patients with dose reductions were stable at the time of data collection with some continuing to taper. Most of the medication changes happened within 3 months after pharmacist consultation (range 2 weeks – 4 months). Average MED decreased from 260.70 mg to 215.43 mg with a 17.4% reduction (*p* = .01).

**Discussion/Conclusions**: Pharmacist screening can be a useful strategy in engaging with patients and prescribers to proactively identify and manage high dose opioid use.

## Pain Response Variability in Infants Born Preterm during Routine Immunizations

Amos Hundert http://orcid.org/0000-0001-8257-2202^a,*^, Adele Orovec^b^, Tim Disher^c^ and Marsha Campbell-Yeo^d^

^a^Department of Community Health and Epidemiology, Dalhousie University and IWK Health Centre, Halifax, Nova Scotia, Canada; ^b^Department of Medicine, Dalhousie University and IWK Health Centre, Halifax, Nova Scotia, Canada; ^c^School of Nursing, Dalhousie University and IWK Health Centre, Halifax, Nova Scotia, Canada; ^d^School of Nursing, Faculty of Health and Departments of Pediatrics, Psychology and Neuroscience, Dalhousie University and IWK Health Centre, Halifax, Nova Scotia, Canada

**CONTACT** Amos Hundert amos.hundert@dal.ca Department of Community Health and Epidemiology, Dalhousie University and IWK Health Centre, Halifax, Nova Scotia, Canada

© 2020 The Author(s). Published with license by Taylor & Francis Group, LLC.

This is an Open Access article distributed under the terms of the Creative Commons Attribution License (http://creativecommons.org/licenses/by/4.0/), which permits unrestricted use, distribution, and reproduction in any medium, provided the original work is properly cited.

**Introduction/Aim**: A multitude of contextual factors contribute to individual differences in pain response. Existing research has begun to characterize individual variability in pain response in full term infants. We aimed to classify individual pain response trajectories during 2, 6, 12, and 18-month immunizations appointments in infants born preterm.

**Methods**: A cohort of preterm infants was followed during routine immunizations. Video was recorded during appointments to capture pain response with the Modified Behavioral Pain Scale (MBPS) assessed at baseline, injection, and recovery (2-minute post needle period). Latent class analysis was used to group infants in classes of pain response trajectories.

**Results**: A total of sixty-four infants (average gestational age 32.5 weeks) participated. Stable classes were identified at each age. At the 2-month immunization, infants demonstrated an increased response to pain, with 17% showing no regulation. From ages 6 to 18-months, fewer infants displayed a pain response. Those who responded were able to regulate pain during the recovery period, with varying time to regulation driving different classes. At every age, during injection, average pain response in at least one class was meaningfully different (>1-point on MBPS) to the sample average.

**Discussion/Conclusions**: There is substantial variation in preterm infants’ pain response trajectories. Infants at older ages demonstrated improved ability to regulate pain. In full term infants mean pain scores within classes differed from the sample mean during recovery, compared to during injection as found in this preterm sample. Further research in larger samples is needed to identify factors associated with pain trajectory class.

## Early Identification of Opioid-Induced Sedation: Taking a Step beyond Looking at Arousability

Danielle Dunwoody http://orcid.org/0000-0002-4326-7405^a,*^

^a^School of Nursing, York University, Toronto, Ontario, Canada

**CONTACT** Danielle Dunwoody ddunwood@yorku.ca School of Nursing, York University, Toronto, Ontario, Canada

© 2020 The Author(s). Published with license by Taylor & Francis Group, LLC.

This is an Open Access article distributed under the terms of the Creative Commons Attribution License (http://creativecommons.org/licenses/by/4.0/), which permits unrestricted use, distribution, and reproduction in any medium, provided the original work is properly cited.

**Introduction/Aim**: Managing acute pain within the acute care setting is challenging for all levels of practitioners. Clinical decision making regarding opioids has the limited supports of linear pain and sedation scales, which can conflict at times with clinical judgment. This study examined the common meanings of opioid-induced sedation and shared practices in the context of post-operative pain management in expert Post-Anesthesia Care Unit (PACU) nurses.

**Methods**: An interpretive phenomenology approach was utilized for this study. Twenty expert PACU nurses participated in qualitative interviews regarding their lived experiences. An interpretive team was utilized for the analysis of data, as well as validating themes and the pattern with a subset of participants.

**Results**: Four themes identified through the participants stories were recognizing that every patient is different, engaging in iterative knowing, walking a fine line, and looking beyond and anticipating. Participants were able to identify the picture of sedation within their clinical practice experiences which supported further dimension to the concept of opioid-induced sedation to include: arousability, hemodynamic and respiratory stability, mobility/motor function, cognition/consciousness, and safety.

**Discussion/Conclusions**: The results of this study suggest further investigation into the role cognition and consciousness play in relationship to the assessment opioid-induced sedation in the context of pain management with opioids specifically, in the acute pain phase. Further exploration into the roles of consciousness and cognition in relationship to opioid-induced sedation is warranted. Balancing pain control with sedation is an iterative, patient specific, and dynamic process that involves complex assessment components of nursing care.

## Mothers Knowledge about Newborn Pain Management: Preliminary Analysis of Maritime Mothers’ Pain-related Knowledge in the First Six Months after Birth

Brianna Richardson^a,b,*^, Justine Dol^c^, Marsha Campbell-Yeo^a,b^, Megan Aston^a^, Gail Tomblin Murphy^d^ and Douglas McMillan^e^

^a^School of Nursing, Dalhousie University, Halifax, NS, Canada; ^b^Centre for Pediatric Pain Research, IWK Health Centre, Halifax, NS, Canada; ^c^Faculty of Health, Dalhousie University, Halifax, NS, Canada; ^d^VP Research, Innovation and Discovery & Chief Nurse Executive, Nova Scotia Health Authority, Halifax, NS, Canada; ^e^Division of Neonatal Perinatal Medicine, Department of Pediatrics, Faculty of Medicine, Dalhousie University and IWK Health Centre, Halifax, NS, Canada

**CONTACT** Brianna Richardson brianna.richardson@dal.ca

© 2020 The Author(s). Published with license by Taylor & Francis Group, LLC.

This is an Open Access article distributed under the terms of the Creative Commons Attribution License (http://creativecommons.org/licenses/by/4.0/), which permits unrestricted use, distribution, and reproduction in any medium, provided the original work is properly cited.

**Introduction/Aim**: All newborns endure painful procedures (e.g. vaccinations) and parents can effectively reduce pain (e.g. by breastfeeding), parents may not be aware of effective strategies. With no evaluations of newborn pain management knowledge in parents from the Maritimes, we aimed to understand the current knowledge base for these mothers during the first six-months after birth.

**Methods**: In 2019, mothers (18 years+) with a newborn less than 6 months of age and from Nova Scotia (NS), New Brunswick (NB), or Prince Edward Island (PEI) completed an online survey that included a 10-item true/false questionnaire about newborn pain management knowledge. Pearson’s chi-square test was used to determine if difference in knowledge was associated with provinces and frequency of correct scores.

**Results**: In total, 578 mothers from NS (46.0%), NB (30.3%) and PEI (23.7%) completed the survey. Maritime mothers demonstrated knowledge of key effective strategies, such as breastfeeding (89.8% correct), holding (91.9% correct), and distraction (81.5% correct), however 55.5% reported incorrect responses regarding sucrose. For participants from NS and PEI, a significant association was found regarding breastfeeding (χ^2^ = 18.35, *p* <.001) and holding (χ^2^ = 13.43, *p* =.001). Within each question, NS had the greatest proportion of correct responses for all questions; PEI had the least proportion of correct responses for 8 questions and NB had the least for 2 questions.

**Discussion/Conclusions**: Preliminary analysis suggests that participants from NS may have more knowledge about effective pain management strategies than those from NS or PEI.Further analyses to determine specific gaps in newborn procedural pain-related knowledge are warranted.

## Pulsed Radiofrequency of Stellate Ganglion for Pain Control in Patient’s with Upper Limb Complex Regional Pain Syndrome: Retrospective Observational Study

Imrat Sohanpal^a,*^, Ramin Safakish^a^, Shadi Babazadeh^a^

^a^Allevio Pain Management, Toronto, Ontario, Canada

**CONTACT** Imrat Sohanpal Imrat.Sohanpal@AllevioClinic.com

© 2020 The Author(s). Published with license by Taylor & Francis Group, LLC.

This is an Open Access article distributed under the terms of the Creative Commons Attribution License (http://creativecommons.org/licenses/by/4.0/), which permits unrestricted use, distribution, and reproduction in any medium, provided the original work is properly cited.

**Introduction/Aim**: Chronic regional pain syndrome (CRPS) is a wide spectrum of painful conditions which are usually occur after an initiating event and diagnosed clinically.

**Methods**: This retrospective observational study of patients who were admitted to Allevio Pain Management during the period January 2013 to December 2018 underwent stellate ganglion Pulsed Radiofrequency (P-RF) for condition Complex Regional Pain Syndrome (CRPS). A total number of 19 patients were identified who underwent an ultrasound guided stellate ganglion block for upper limbs CRPS. This project has been conditionally approved by Veritas IRB Inc.

**Results**: All the 19 charts had been reviewed. The median age of study participants was 48 years, the baseline median scores for pain intensity and interference were 7.0. Over half of the patients experienced clinically meaningful improvements in BPI. The median pain-free period was 2 months. Eight out of 17 patients returned to work after the intervention. Before intervention, nine out of 19 patients were using narcotic analgetics. (dosage was 13.5–200 mg). After the intervention only one patient, remain on narcotic medication but with lower dose.

**Discussion**: Based on the above retrospective results, over half of the patients had greater than 50% reduction in pain with a median duration of approximately 2 months after pulsed radiofrequency of the stellate ganglion, and a significant Improvement in quality of life based on post treatment BPI results. Ultrasound guided technique of Pulsed Radiofrequency of Stellate ganglion offers a peripheral neuromodulation with the potential of extended pain relief to allow continuous rehabilitation and functional restoration.

## The Effects of Persisting with an Ineffective Pain Coping Strategy: A Pilot Study

Lindsey Yessick^a,*^, Kathleen Hoy^a^ and Tim Salomons^a^

^a^Psychology, Queen’s University, Kingston, Ontario, Canada

**CONTACT** Lindsey Yessick lindsey.yessick@queensu.ca

© 2020 The Author(s). Published with license by Taylor & Francis Group, LLC.

This is an Open Access article distributed under the terms of the Creative Commons Attribution License (http://creativecommons.org/licenses/by/4.0/), which permits unrestricted use, distribution, and reproduction in any medium, provided the original work is properly cited.

**Introduction/Aim**: Those who perceive a behavioral solution for removing pain will expend more effort taking action (i.e., problem-focused strategy). In contrast, those who perceive low behavioral control might adapt a more passive, emotion-focused coping strategy. The strategy that is most effective in a given situation depends on the amount of behavioral control that is available, and the individual’s ability to accurately gauge that control. As such, we would predict that persistence in an ineffective problem-focused coping strategy, would increase the aversive effects of nociceptive stimulation.

**Methods**: Twelve participants received 45 painful thermal stimuli and were offered two possible coping strategies: one problem-focused strategy (a puzzle that could result in complete cessation of pain stimulation if solved) and one emotion-focused strategy (comforting sounds that would run concurrent with subsequent stimuli but reduce distress). Participants were told they could switch to the emotion-focused strategy at any point. The problem-focused strategy was manipulated to be unsolvable, such that the emotion-focused strategy was the more adaptive option.

**Results**: Individuals that persisted with the ineffective active coping strategy (*n* = 6) experienced greater pain intensity (*d = *0.25) and unpleasantness (*d = *0.20) than those who switched to the emotion-focused strategy (*n* = 6). Similarly, individuals who persisted with the active strategy experienced a stronger increase in state anxiety (*d* = 1.09).

**Discussion/Conclusions**: Individuals who persisted with an ineffective strategy for coping with experimental pain in this pilot study experienced more pain and a greater increase in anxiety. Additional data are currently being collected.

## Perceptions of the 2017 Canadian Guideline for Opioid Therapy and Chronic Noncancer Pain: A Cross-Sectional Study of Canadian Physicians

Jason W. Busse http://orcid.org/0000-0002-0178-8712^a,b,c,*^, Joyce Douglas^d^, Tara S. Chauhan^d^, Bilal Kobeissi^d^ and Jeff Blackmer^d^

^a^Departments of Anesthesia and Health, Evidence and Impact (HEI), McMaster University, Hamilton, Ontario, Canada; ^b^Michael G. DeGroote Institute for Pain Research and Care, McMaster University, Hamilton, Ontario, Canada; ^c^Michael G. DeGroote Centre for Medicinal Cannabis Research, McMaster University, Hamilton, Ontario, Canada; ^d^Canadian Medical Association, Ottawa, Ontario, Canada

**CONTACT** Jason W. Busse bussejw@mcmaster.ca

© 2020 The Author(s). Published with license by Taylor & Francis Group, LLC.

This is an Open Access article distributed under the terms of the Creative Commons Attribution License (http://creativecommons.org/licenses/by/4.0/), which permits unrestricted use, distribution, and reproduction in any medium, provided the original work is properly cited.

**Introduction/Aim**: Physician adherence to guideline recommendations for use of opioids to manage chronic pain is often limited

**Methods**: In February 2018, we administered a 28-item, online survey to explore perceptions of the 2017 Canadian guideline for opioid therapy and chronic noncancer pain, and if physicians had altered practices in response to recommendations.

**Results**: We invited 34,322 Canadian physicians to complete our survey, and 1,128 responded for a response rate of 3%. Almost all were aware of the guideline, 94% had read the document, and 89% endorsed the clarity as good or excellent. The majority (86%) felt the guideline was feasible to implement, but 66% highlighted resistance by patients, and 63% the lack of access to effective non-opioid treatment, as barriers. Thirty-seven percent of respondents mistakenly believed the guideline recommended mandatory tapering for patients using high-dose opioid therapy (≥90 mg morphine equivalent per day), and 58% felt they would benefit from support for opioid tapering. Seventy percent of respondents had changed practices to align with guideline recommendations, with 51% engaging some high-dose patients in opioid tapering and 43% reducing the number of new opioid starts.

**Discussion/Conclusions**: There was high awareness of the 2017 Canadian opioid guideline among respondents, and preliminary evidence that recommendations have changed practice to better align with the evidence. Ongoing education is required to avoid the misunderstanding that opioid tapering is mandatory, and research to identify effective strategies to manage chronic noncancer pain is urgently needed.

## Exploring Discrepancies in Recommendations among Recent Clinical Practice Guidelines for Opioids and Chronic Non-cancer Pain

Jason W. Busse http://orcid.org/0000-0002-0178-8712^a,b,c,*^ and Gordon H. Guyatt http://orcid.org/0000-0003-2352-5718^d^

^a^Departments of Anesthesia and Health, Evidence & Impact (HEI), McMaster University, Hamilton, Ontario, Canada; ^a^Michael G. DeGroote Institute for Pain Research and Care, McMaster University, Hamilton, Ontario, Canada; ^b^Michael G. DeGroote Centre for Medicinal Cannabis Research, McMaster University, Hamilton, Ontario, Canada; ^c^Department of Health, Evidence, & Impact (HEI), McMaster University, Hamilton, Ontario, Canada

**CONTACT** Jason W. Busse bussejw@mcmaster.ca

© 2020 The Author(s). Published with license by Taylor & Francis Group, LLC.

This is an Open Access article distributed under the terms of the Creative Commons Attribution License (http://creativecommons.org/licenses/by/4.0/), which permits unrestricted use, distribution, and reproduction in any medium, provided the original work is properly cited.

**Introduction**/**Aim**: From 2016–2017, three clinical practice guidelines were published that made recommendations for opioids and chronic noncancer pain. We explored discrepancies between these guidelines, and reasons for these differences.

**Methods**: We reviewed the methodology and recommendations in 3 guidelines: (1) the 2016 CDC Guideline for Prescribing Opioids for Chronic Pain, (2) the 2017 Canadian Guideline for opioid therapy and chronic noncancer pain, and (3) the VA/DoD Clinical Practice Guideline for Opioid Therapy for Chronic Pain.

**Results**: In brief, both the CDC and VA/DoD guidelines made almost all strong recommendations (only 1 weak in each), whereas the Canadian guideline made mostly weak recommendations (6 of 10), despite all claiming use of the GRADE approach. The CDC guideline claimed there was no evidence to make a recommendation for use of opioids to manage chronic noncancer pain, the Canadian guideline made a weak recommendation for use of opioids to manage chronic noncancer pain (among selected patients), and the VA/DoD guideline made a strong recommendation against the use of opioids for chronic noncancer pain. These discrepancies were due, in part, to variability in how guidelines considered and synthesized evidence and because both the CDC and VA/DoD made a number of strong recommendations without supporting empirical evidence (ie. based on expert consensus, which is contrary to the GRADE approach).

**Discussion/Conclusion**: Clinical practice guidelines for opioids and chronic pain should adopt common methodology based on best practices to avoid sending competing messages to clinicians, patients, and policy-makers.

## Prevalence and Predictors of Opioid Use Disorder following Prescription for Chronic Noncancer Pain: A Systematic Review of Observational Studies

Ngai Chow http://orcid.org/0000-0002-1800-0362^a^, Li Wang^b^, Elena Kum http://orcid.org/0000-0002-6548-2632^c^, Elie Isenberg-Grzeda^d^, Gwendolyn Lovsted^e^, Atefeh Noori^a^, Patrick Jiho Hong^f^, Yasir Rehman^a^, Mahmood AminiLari^a^, Kayli Culig^g^, Nooralhuda Bakaa^a^, Alexandra Nieuwesteeg^h^, James MacKillop^i^, Jennifer Brasch^i^, Rachel Couban^b^ and Jason W. Busse http://orcid.org/0000-0002-0178-8712^j,k,l,*^

^a^Department of Health Research Methods, Evidence, and Impact, McMaster University, Hamilton, Ontario, Canada; ^b^Department of Anesthesia, McMaster University, Hamilton, Ontario, Canada; ^c^Faculty of Science, Western University, London, Ontario, Canada; ^d^Department of Psychiatry, Sunnybrook Health Sciences Centre and University of Toronto, Toronto, Ontario, Canada; ^e^Department of Medicine, McMaster University, Hamilton, Ontario, Canada; ^a^Department of Anesthesia, University of Toronto, Toronto, Ontario, Canada; ^f^Department of Medicine, University of Toronto, Toronto, Ontario, Canada; ^g^School of Medicine, Royal College of Surgeons in Ireland, Dublin, Ireland; ^h^Department of Psychiatry and Behavioural Neurosciences, McMaster University, Hamilton, Ontario, Canada; ^i^Departments of Anesthesia and Health Research Methods, Evidence and Impact (HEI), McMaster University, Hamilton, Ontario, Canada; ^j^Michael G. DeGroote Institute for Pain Research and Care, McMaster University, Hamilton, Ontario, Canada; ^k^Michael G. DeGroote Centre for Medicinal Cannabis Research, McMaster University, Hamilton, Ontario, Canada

**CONTACT** Jason W. Busse bussejw@mcmaster.ca

© 2020 The Author(s). Published with license by Taylor & Francis Group, LLC.

This is an Open Access article distributed under the terms of the Creative Commons Attribution License (http://creativecommons.org/licenses/by/4.0/), which permits unrestricted use, distribution, and reproduction in any medium, provided the original work is properly cited.

**Introduction/Aim**: We systematically reviewed observational studies to establish the prevalence of opioid use disorder (OUD) among patients prescribed this class of medication for CNCP, and to explore factors associated with the development of OUD.

**Methods**: We searched MEDLINE, EMBASE, CINAHL, Cochrane Library, and PsycINFO from inception to December 2018. Two specialists in addiction medicine reviewed each potentially eligible study, blinded to results, to ensure their outcome met current criteria for OUD. We pooled estimates of OUD across eligible studies using random-effects models. When possible, we pooled estimates of association with OUD for all independent variables reported by more than one study.

**Results**: Twenty-two studies were eligible for our review. Rates of OUD across studies ranged considerably, and the pooled prevalence of OUD was 20% (95% CI: 15% to 24%). We found moderate certainty evidence for a significant association between OUD and age (odds ratio [OR] for every 10-year increment, 0.63; 95% CI: 0.43 to 0.90) and current smokers (OR 1.63; 95% CI: 1.25 to 2.12); high certainty evidence for a significant association between OUD and male sex (OR 1.50; 95% CI: 1.05 to 2.14); and low certainty evidence for a significant association between OUD and a history of mental health disorders (OR 1.49; 95% CI: 1.17 to 1.89). Low certainty evidence showed no significant association between OUD and history of alcohol abuse/dependence or history of drug abuse.

**Discussion/Conclusions**: Limited evidence suggests that one in five CNCP patients prescribed opioids will develop OUD.

## A Novel Chemo-/Opto-Genetic Tool for the Bidirectional Control of Neuronal Activity in Freely Moving Mice

Haoyi Qiu^a,*^, Hugues Petitjean^a^, Albena Davidova^a^ and Reza Sharif-Naeini http://orcid.org/0000-0001-8896-5306^a^

^a^Department of Physiology and Cell Information Systems, McGill University, Montreal, Quebec, Canada

**CONTACT** Reza Sharif Naeini reza.sharif@mcgill.ca Department of Physiology and Cell Information Systems, McGill University, Montreal, Quebec, Canada

© 2020 The Author(s). Published with license by Taylor & Francis Group, LLC.

This is an Open Access article distributed under the terms of the Creative Commons Attribution License (http://creativecommons.org/licenses/by/4.0/), which permits unrestricted use, distribution, and reproduction in any medium, provided the original work is properly cited.

**Introduction/Aim**: The dorsal horn of the spinal cord is the first site of somatosensory input integration from the periphery. In its superficial layers, nociceptive inputs are processed by a complex network of excitatory and inhibitory interneurons whose function and connectivity remain poorly understood. Here, we introduce an innovative tool that allows for the precise control of the activity of a specific interneuron population.

**Methods**: To evaluate the efficacy of this tool, we used knock-in mice expressing Cre recombinase under the control of the calretinin (CR; CR:Cre mice) gene, a marker of excitatory spinal cord interneurons. The tool consisted of an inhibitory pharmacogenetic tool (hM4D) and an excitatory opsin (oChIEF) inserted in a Cre-dependent manner in an AAV2/9 viral vector. The virus was injected unilaterally in the spinal cord of Cr:Cre mice. Injections were followed by surgical implantation of a cannula through which a software-controlled blue laser modulated oChIEF activity.

**Results**: Upon transient photostimulation, virus-injected mice displayed spontaneous pain behaviors. Our results further indicate that the intensity of the spontaneous pain and nocifensive responses increased with an increase in laser power. After intraperitoneal injection of CNO, photostimuli were unable to produce nocifensive responses. These photostimulation-elicited nocifensive responses were evident once the drug was eliminated from the system.

**Discussion/Conclusions**: We present functional evidence that this vector can be used to inhibit or activate CR neurons *in vivo*. Our results indicate that this vector enables the bidirectional control of neuronal activity in defined subsets of interneurons to elucidate their physiological role *in vivo*.

## Views Towards a Novel Conflict of Interest Management Approach for the Canadian Opioid Guideline: A Qualitative Study

Gladys Honein-Abouhaidar^a,*^, Caroline El Rayes^b^, Jason W. Busse http://orcid.org/0000-0002-0178-8712^c^, Samantha Craigie^d^, Gordon H. Guyatt http://orcid.org/0000-0003-2352-5718^e^ and Elie Akl^f^

^a^Hariri School of Nursing, American University of Beirut, Beirut, Lebanon; ^b^Faculty of Health Sciences, American University of Beirut, Beirut, Lebanon; ^c^Departments of Anesthesia and Health Research Methods, Evidence and Impact (HEI), Michael G. DeGroote Institute for Pain Research and Care; Michael G. DeGroote Centre for Medicinal Cannabis Research, McMaster University, Hamilton, Ontario, Canada; ^d^Anesthesia, McMaster University, Hamilton, Ontario, Canada; ^e^Health Research Methods, Evidence and Impact, McMaster University, Hamilton, Ontario, Canada; ^f^Internal Medicine, American University of Beirut, Beirut, Lebanon

**CONTACT** Gladys Honein-Abouhaidar gh30@aub.edu.lb

© 2020 The Author(s). Published with license by Taylor & Francis Group, LLC.

This is an Open Access article distributed under the terms of the Creative Commons Attribution License (http://creativecommons.org/licenses/by/4.0/), which permits unrestricted use, distribution, and reproduction in any medium, provided the original work is properly cited.

**Introduction/Aim**: The 2017 Canadian opioid guideline pursued a voting panel without financial or intellectual conflicts of interest that was advised by conflicted content experts who were not present when clinical practice recommendations were voted on. The objective of this study was to assess the views of the participants regarding this approach toward managing conflict of interest (COI).

**Methods**: We conducted in-depth telephone semi-structured interviews with 16 content experts and panelists involved in the guideline. We used a thematic inductive analytical approach to analyze the data.

**Results**: We identified the following themes: (1) justification of the approach, (2) perceived strengths, (3) perceived limitations, and (4) suggestions for improvement. The main justification was balancing the need for expert input with avoiding the potential for financial and intellectual conflicts of interest to affect guideline recommendations. Perceived strengths included: selection of participants representing a range of views toward opioids for chronic non-cancer pain, content experts’ ability to fully express their opinions, and a dynamic and balanced discussion based on evidence. Perceived limitations included loss of clinical expertise from the panel (primarily from the experts), reservations regarding the COI vetting process, and the indirect influence that experts had on the final recommendations (primarily from the experts). Suggestions included improving the vetting for COI, verifying COI, and recruiting clinical experts with minimal COIs into the voting panel.

**Discussion/Conclusions**: Restricting panel participants to those without conflict while recruiting conflicted experts for thorough discussion to inform the panel represents an innovative approach to addressing COI on guideline panels.

## The Role of Task Difficulty in the Cognitive Modulation of Pain

Sophie Desjardins^a,*^, Todd A. Vogel^a^ and Mathieu Roy^a^

^a^Department of Psychology, McGill University, Montreal, Quebec, Canada

**CONTACT** Sophie Desjardins sophie.desjardins4@mail.mcgill.ca

© 2020 The Author(s). Published with license by Taylor & Francis Group, LLC.

This is an Open Access article distributed under the terms of the Creative Commons Attribution License (http://creativecommons.org/licenses/by/4.0/), which permits unrestricted use, distribution, and reproduction in any medium, provided the original work is properly cited.

Increasingly, non-pharmacological avenues of chronic pain treatment have been investigated in response to the inadequacy of pharmacological treatments. For instance, the analgesic effect of cognitive effort on pain is well demonstrated. Performing a difficult cognitive task is associated with a substantial reduction in pain. Though ample research has examined the analgesic effect of cognitive effort, none so far has assessed how difficult the task performed must be in order to produce a reduction in pain. Our study sought to investigate the role of task difficulty in the modulation of pain through cognitive effort.

**Introduction/Aim**: Examining the effect of task difficulty on the cognitive modulation of pain.

**Methods**: Seventeen healthy young adults were recruited to participate in the study. First, we performed a sensory calibration to assess pain tolerance. The participant was presented with 28 thermal stimulations and asked to rate them. These ratings were fitted into a logistic regression model to determine what temperature corresponded to 150/200 on VAS scale. Second, we performed a cognitive calibration to assess task performance. The participant performed a difficult cognitive task, the 2-back, and a staircase method was used to identify three levels of difficulty calibrated to the participant’s ability to perform on the task. The three levels were low, medium and high. Finally, the participant performed the main behavioral task which combined pain and cognitive effort, both of which were calibrated to their pain tolerance and task performance ability. The participant performed the 2-back, varying at three levels of difficulty, while receiving painful stimulations and subsequently rating them.

**Results**: We performed a one-way repeated measures ANOVA to assess mean differences in pain ratings across the three levels of task difficulty. Mauchly’s test of sphericity indicated that the assumption of sphericity had been violated, χ^2^(2) = 15.270, *p* < .001, therefore a Greenhouse-Geisser correction was used (Ɛ = 0.610). We found a significant effect of task difficulty on pain ratings *F*(2, 16) = 4.78, *p* < .05. Post-hoc comparisons using the Tukey HSD test showed that while low and high difficulty levels did not significantly differ in terms of pain ratings, the medium level of difficulty differed from both the low and the high levels in terms of pain ratings at *p* < .05.

**Discussion/Conclusions**: Overall, our findings suggest that inhibition of pain through cognitive effort depends largely on task difficulty. Although pain can be reduced through cognitive effort, the task at hand must be adjusted to a moderate level of difficulty. Low and high levels of difficulty may be inadequate for pain inhibition to occur. This study has large implications for the development of non-pharmacological forms of treatment for chronic pain like distraction.

## Electronic Versus Traditional Data Collection: A Multicenter Randomized Controlled Perioperative Pain Trial

James S. Khan^a,*^, Lindsay A. Jibb^b^, Jason W. Busse http://orcid.org/0000-0002-0178-8712^c,d,e^, Ian Gilron^f^, Stephen Choi^a^, James E. Paul^g^, Michael McGillion^h^, Sean Mackey^i^, D. Norman Buckley http://orcid.org/0000-0002-1031-6813^g^, Shun Fu Lee^j^ and P. J. Devereaux^k^

^a^Anesthesia, University of Toronto, Toronto, Ontario, Canada; ^b^School of Nursing, University of Ottawa, Ottawa, Ontario, Canada; ^c^Departments of Anesthesia and Health Research Methods, Evidence and Impact (HEI), McMaster University, Hamilton, Ontario, Canada; ^d^Michael G. DeGroote Institute for Pain Research and Care, McMaster University, Hamilton, Ontario, Canada; ^e^Michael G. DeGroote Centre for Medicinal Cannabis Research, McMaster University, Hamilton, Ontario, Canada; ^f^Anesthesiology & Perioperative Medicine, Queen’s University, Kingston, Ontario, Canada; ^g^Anesthesia, McMaster University, Hamilton, Ontario, Canada; ^h^School of Nursing, McMaster University, Hamilton, Ontario, Canada; ^i^Pain Medicine, Stanford University, Palo Alto, California, USA; ^j^Population Health Research Institute, McMaster University, Hamilton, Ontario, Canada; ^k^Health Research Methods, Evidence and Impact, McMaster University, Hamilton, Ontario, Canada

**CONTACT** James S. Khan james.khan@medportal.ca

© 2020 The Author(s). Published with license by Taylor & Francis Group, LLC.

This is an Open Access article distributed under the terms of the Creative Commons Attribution License (http://creativecommons.org/licenses/by/4.0/), which permits unrestricted use, distribution, and reproduction in any medium, provided the original work is properly cited.

**Introduction/Aim**: Electronic data collection is increasingly available as a means to collect pain related clinical trial data; however, effectiveness and costs relative to traditional data collection are uncertain. The aim of this study was to evaluate data quality, protocol adherence, satisfaction, and resource requirements of electronic data collection compared to traditional data collection methods in a perioperative factorial randomized controlled trial.

**Methods**: This study was an open-label two-arm parallel randomized controlled trial. Women undergoing breast cancer surgery were allocated to either electronic or traditional data collection and completed pain-related questionnaires at baseline, postop, and 3-month follow-up (NCT02240199).

**Results**: We acquired outcome data at all time points from 38 patients in the electronic group and 40 in the traditional group. The number of data queries (e.g., erroneously entered data) per patient was higher in the electronic data group (4.92 [SD = 4.67] vs. 1.88 [SD = 1.51]; *P* < .001). No between-group differences were observed for compliance with medications, data completeness, loss to follow-up, or patient or research assistant satisfaction. More research assistant time per patient was spent collecting data in the traditional group (42.6 min [SD = 12.8] vs. 9.92 min [SD = 7.6]; *P* < .001); however, costs per patient were higher in the electronic group ($176.85 [SD = 2.90] vs. $16.33 [SD = 4.90]; *P* < .001).

**Discussion/Conclusions**: Electronic data collection is feasible for perioperative pain clinical trials. Additional trials, including different surgical populations, are needed to confirm our findings and optimize use of electronic data capture methods.

## Fear of Movement or (Re)injury in Children and Adolescents Undergoing Major Surgery: Validation of the Tampa Scale for Kinesiophobia

Brittany N. Rosenbloom^a,*^, M. Gabrielle Pagé^b^, Lisa Isaac^c^, Fiona Campbell^c^, Jennifer Stinson^d^, Robert Cribbie http://orcid.org/0000-0002-9247-497X^a^ and Joel Katz^a^

^a^Psychology, York University, Toronto, ON, Canada; ^b^Department of Anesthesiology and Pain Medicine, Centre de recherche du Centre hospitalier de l’Université de Montréal, Montreal, QC, Canada; ^c^Anesthesia, The Hospital for Sick Children, Toronto, ON, Canada; ^d^Nursing, The Hospital for Sick Children, Toronto, ON, Canada

**CONTACT** Brittany N. Rosenbloom bnrosen@yorku.ca

© 2020 The Author(s). Published with license by Taylor & Francis Group, LLC.

This is an Open Access article distributed under the terms of the Creative Commons Attribution License (http://creativecommons.org/licenses/by/4.0/), which permits unrestricted use, distribution, and reproduction in any medium, provided the original work is properly cited.

**Introduction/Aim**: The Tampa Scale for Kinesiophobia (TSK) was designed to measure fear of movement or (re)injury in adults. The aim of this study was to evaluate the psychometric properties of the TSK in children and adolescents undergoing major surgery.

**Methods**: Participants included 264 children and adolescents (58.7% female, M_age_ = 14.1 years, SD_age_ = 2.51) scheduled for major surgery. Participants completed questionnaires before surgery, while in hospital postoperatively, and at 6 and 12 months after surgery. Exploratory factor analyses (EFA) were conducted to determine the factor structure of the TSK. Reliability, and convergent, divergent, and predictive validity were examined.

**Results**: EFA on the 17-item TSK revealed a two-factor model dividing 13 positively-worded items from 4 negatively-worded items. Given these results, a second EFA was conducted on only the 13 positively-worded items (TSK-13) revealing a three-factor model: Fear of injury, bodily vulnerability, and activity avoidance. The TSK-13 showed adequate internal consistency (α = 0.81, Ω = 0.82) and moderate convergent validity with measures of pain intensity (*r* = 0.25, *p* < .001), pain unpleasantness (*r* = 0.41, *p < *.001), and pain avoidance (*r* = 0.53, *p* < .001). The TSK-13 was not correlated with actual postoperative movement as measured by continuous physical activity monitoring (actigraphy; *r* = −0.10, *p* = .18) and showed adequate divergent validity from measures of depression (*r* = 0.41, *p* < .001) and general anxiety (*r* = 0.35, *p* < .001). Predictive validity for pain-related disability at 12 months (*r* = 0.34, *p* < .001) was adequate.

**Discussion/Conclusions**: The original TSK-17 does not appear to be a meaningful measure for youth after surgery. The TSK-13 revealed a 3-factor structure that is reliable and demonstrates adequate convergent, divergent, and predictive validity.

## The Sociocultural Context of Pediatric Pain: A Multi-Method Examination of Portrayal of Pain in Children’s Popular Media

Kendra Mueri^a,*^, Madison Kennedy^a^, Maria Pavlova^a^, Alexandra Neville^a^, Tatiana Lund^a^ and Melanie Noel http://orcid.org/0000-0003-3752-8055^a^

^a^Psychology, University of Calgary, Calgary, Alberta, Canada

**CONTACT** Kendra Mueri kendra.mueri@ucalgary.ca

© 2020 The Author(s). Published with license by Taylor & Francis Group, LLC.

This is an Open Access article distributed under the terms of the Creative Commons Attribution License (http://creativecommons.org/licenses/by/4.0/), which permits unrestricted use, distribution, and reproduction in any medium, provided the original work is properly cited.

**Introduction/Aim**: Early childhood pain experiencs set the stage for how individuals experience pain across the lifespan. Given that media is introduced during critical developmental periods, it is imperative to understand how pain is portrayed in children’s media. Aims: **1)** To explore how pain is portrayed and gendered in popular children’s media. **2)** To explore how parents and children perceive and discuss painful instances depicted in media.

**Methods**: Five children’s television shows and ten movies were selected from a list of seventy-five. Pain instances were coded using established coding schemes characterizing pain expression, pain experience, and behaviors of sufferers and observers. Sixty mother- and father-child dyads (children 4–6 years) will be recruited from the community and asked to view clips of painful instances to assess beliefs and perceptions toward pain portrayals.

**Results**: Of 381 identified pain instances, pain in boy characters accounted for 71.65% of all instances. The majority of pain instances were acute, short in duration, and seldom highlighted pain as a main story arc. Observers in the sufferer’s environment engaged in minimal prosocial behaviors, and ordeals of pain were often mitigated through humor or avoidance. Participant recruitment is ongoing and will include analysis of parent-child perceptions.

**Discussion/Conclusions**: Findings reveal an underrepresentation of girl characters experiencing pain, which could contribute to gender stereotypes of pain. Common social responses to pain were not empathic/prosocial and often included avoidance. By understanding how negative attitudes and behaviors toward pain are socialized at a young age, we can help educate parents and children how to best approach pain.
